# Genome-wide identification of Wig-1 mRNA targets by RIP-Seq analysis

**DOI:** 10.18632/oncotarget.6557

**Published:** 2015-12-11

**Authors:** Cinzia Bersani, Mikael Huss, Stefania Giacomello, Li-Di Xu, Julie Bianchi, Sofi Eriksson, Fredrik Jerhammar, Andrey Alexeyenko, Anna Vilborg, Joakim Lundeberg, Weng-Onn Lui, Klas G. Wiman

**Affiliations:** ^1^ Department of Oncology-Pathology, Karolinska Institutet, Cancer Center Karolinska, Stockholm, Sweden; ^2^ Science for Life Laboratory, School of Biotechnology, Royal Institute of Technology, Solna, Sweden; ^3^ Department of Microbiology, Tumour and Cell biology, Bioinformatics Infrastructure for Life Sciences, Science for Life Laboratory, Karolinska Institutet, Stockholm, Sweden; ^4^ Department of Molecular Biophysics and Biochemistry, Yale University, New Haven, CT, USA

**Keywords:** Wig-1, AREs, RIP-Seq, cell cycle, p53

## Abstract

RNA-binding proteins (RBPs) play important roles in the regulation of gene expression through a variety of post-transcriptional mechanisms. The p53-induced RBP Wig-1 (Zmat3) binds RNA through its zinc finger domains and enhances stability of p53 and N-Myc mRNAs and decreases stability of FAS mRNA. To identify novel Wig-1-bound RNAs, we performed RNA-immunoprecipitation followed by high-throughput sequencing (RIP-Seq) in HCT116 and Saos-2 cells. We identified 286 Wig-1-bound mRNAs common between the two cell lines. Sequence analysis revealed that AU-rich elements (AREs) are highly enriched in the 3′UTR of these Wig-1-bound mRNAs. Network enrichment analysis showed that Wig-1 preferentially binds mRNAs involved in cell cycle regulation. Moreover, we identified a 2D Wig-1 binding motif in HIF1A mRNA. Our findings confirm that Wig-1 is an ARE-BP that regulates cell cycle-related processes and provide a novel view of how Wig-1 may bind mRNA through a putative structural motif. We also significantly extend the repertoire of Wig-1 target mRNAs. Since Wig-1 is a transcriptional target of the tumor suppressor p53, these results have implications for our understanding of p53-dependent stress responses and tumor suppression.

## INTRODUCTION

Wig-1, also known as Zmat3 and PAG608, is an RNA-binding protein (RBP) that can bind and regulate expression of multiple mRNAs. Wig-1 contains three highly conserved C2H2-type zinc-finger domains that mediate RNA-protein interactions [1–3], preferentially binding double-stranded RNA (dsRNA). The first and second Wig-1 zinc fingers are required for dsRNA binding [4]. The effects of Wig-1 knockdown as well as the repertoire of known Wig-1 target mRNAs [5–7] indicate that Wig-1 acts as a prosurvival factor. Wig-1 stabilizes the mRNA of the N-Myc oncogene [6], a potent driver of cell cycle proliferation, while also promoting the decay of the pro-apoptotic FAS receptor mRNA [5] leading to decreased cell death. Moreover, Wig-1 can prevent cellular senescence by enhancing mRNA degradation of the cell cycle arrest gene p21 [8]. Interestingly, Wig-1 also stabilizes mRNA of the p53 tumor suppressor – which itself activates Wig-1 transcription [7] – thus enhancing the cell's ability to respond to stress and damage, providing a safeguard for maintained genomic stability. In line with the notion that Wig-1 is an mRNA regulator, Wig-1 has recently been identified in the repertoire of RBPs in the mouse embryonic stem cells [9] and HeLa cells [10] mRNA interactome by a method called “interactome capture”, which combines UV cross-linking of RBPs to RNA in living cells, oligo(dT) capture and mass spectrometry.

We have previously shown that Wig-1 binds to mRNAs through AU-rich element motifs (AREs) [5–7]. AREs are cis-acting regulatory elements present in 8% of the human transcriptome, primarily in the 3′UTR of mRNAs encoding proteins involved in inflammatory responses, cell cycle regulation, and transcription, but also other classes of proteins involved in a wide range of cellular processes [11]. The AU-rich element core is a pentanucleotide sequence element (AUUUA), embedded in a uracil-rich region in the 3′UTR of mRNAs. AREs have been classified as Class I AREs (1–3 scattered AUUUA motifs), Class II AREs (multiple overlapping AUUUA motifs), and Class III AREs (less well defined and lacking an AUUUA motif, often including longer stretches of Us) [12]. The modulation of gene expression via ARE-mediated decay (AMD) is crucial for the regulation of homeostasis and normal physiology as demonstrated by the various reports indicating that the knockout of well-studied ARE binding proteins (ARE-BPs), such as tristetraprolin (TTP), butyrate response factor 1 (BRF1) and Human antigen R (HuR), leads to severe pathologies, defective development or embryonic lethality [13–15]. The AMD pathway is complex; both cooperation and antagonism between different ARE-BPs has been observed in regulation of mRNA expression of a common target [16, 17]. Large-scale sequencing studies have indicated that ARE-BPs may have multiple mRNA targets and can form regulatory complexes with other RBPs [18, 19]. In addition, local secondary RNA structures can strongly affect protein/RNA interactions and will therefore have an impact on mRNA stability [20], alternative splicing [21] and localization [22]. Hence, a cross-comparison of sequence and structural conserved motifs should generate useful biological insights.

In this study we have performed a systematic analysis of Wig-1-associated mRNAs. Through RNA-immunoprecipitation followed by high-throughput sequencing (RIP-Seq) in HCT116 and Saos-2 cells, we found 286 Wig-1-bound mRNAs that are common in the two cell lines. Network enrichment analysis revealed that Wig-1 target mRNAs are highly connected to Cell Cycle regulation pathways. Furthermore, integration of the data from the RIP-Seq in HCT116 with our previously published gene expression data from the same cells upon Wig-1 knockdown [5] resulted in a list of 209 mRNAs that are bound and regulated by Wig-1, the majority of which are destabilized. Bioinformatics analysis confirmed that Wig-1 preferentially binds to mRNAs containing AREs and/or 3′UTRs with high A and U content. Together, our data provide major novel insights into Wig-1-mediated regulation of cell proliferation, the RNA-binding properties of Wig-1, and the Wig-1-regulated transcriptome.

## RESULTS

### Identification of Wig-1-bound mRNAs by RIP-Seq

**Figure 1:**
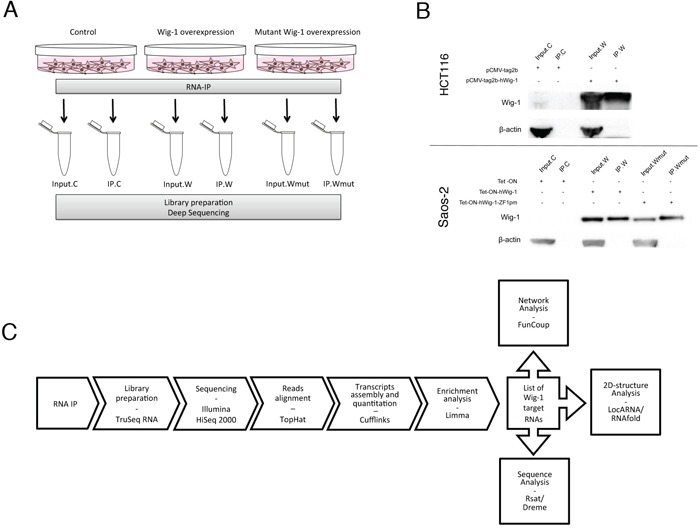
RIP strategy for isolation of Wig-1-associated RNAs **A.** Overview of the RIP-Seq experimental setup using HCT116 and Saos-2 cells. C: control (empty vector); W: Flag-Wig-1; Wmut: Flag-Wig-1-point mutant; IP: immunoprecipitated. **B.** Representative Western blot to confirm Wig-1 pulldown in the RIP experiments. Wig-1 was precipitated with anti-Flag beads in HCT116 cells transiently transfected with pCMV-tag2b (Flag empty vector) or pCMV-tag2b-hWig-1 (Flag-tagged Wig-1) and in Saos-2 TetON cells stably expressing either Flag-tagged wt Wig-1 (Wig-1) or a Flag-tagged Wig-1 zinc-finger 1 point mutant that cannot bind to RNA (Wig-1ZF1pm), or not expressing any exogenous Wig-1 (control, C). Wig-1 protein detection was performed with an antibody recognizing full length Wig-1, see materials and methods. **C.** Outline of the RIP-Seq data analysis (see Materials and Methods for further details).

To isolate Wig-1-bound mRNAs, we performed RIP assays followed by high-throughput sequencing in HCT116 and Saos-2 cells expressing Flag-tagged Wig-1 (W), as well as Saos-2 cells expressing Flag-tagged Wig-1 with a point mutation in the first zinc-finger (Wmut), which is therefore unable to bind to RNA [3] (Figure 1A–1B). We performed triplicate experiments for both cell lines and compared Wig-1-overexpressing samples to control samples (from cells transfected with empty vector) and to samples overexpressing mutant Wig-1. The outline of the experiment is summarized in Figure 1C. Input samples for each condition were also collected and sequenced. We obtained an average of 15.4 and 15.8 million reads for the libraries from RIP of control cells and Wig-1-transfected HCT116 cells, respectively. For Saos-2 cells, we obtained an average of 20.6, 17.3 and 21.2 million reads for RIP from control, wt Wig-1-overexpressing, and mutant Wig-1-overexpressing cells, respectively. RIP control samples contained a high number of duplicate sequences, indicating significant over-sequencing due to low RNA material in the sample (Supplementary Table S1). Notably, the IPs from Wig-1-overexpressing cells consistently yielded more mapped reads after duplicate removal than the RIP from control cells (14% more in HCT116 and 16% more in Saos-2) and from the mutant Wig-1 RIPs performed in Saos-2 cells (20% more).

**Figure 2:**
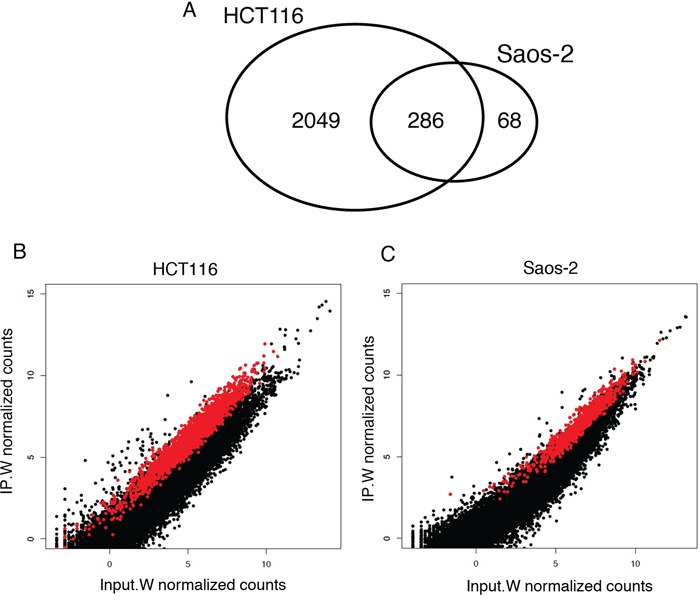
RIP-Seq enrichment analysis identified 286 Wig-1-bound mRNAs common between HCT116 and Saos-2 cells **A.** Venn diagram illustrating the numbers of mRNA identified as Wig-1 RNA targets in both HCT116 and Saos-2 cells, or in only one of the two cell lines. **B.** and **C.** Scatterplot of RIP-Seq data from HCT116 (panel B) and Saos-2 (panel C) cells, showing the normalized mean read counts for each transcript detected in the Wig-1 RIP sample (IP.W) plotted against the read count for the same transcript in the Wig-1 input sample (Input.W) (log2 scale). Black dots represent background RNAs (below the 2-fold cutoff, defined as not bound by Wig-1), while the red dots represent enriched RNAs (above the 2-fold cutoff, defined as bound by Wig-1). See materials and methods for details.

Enrichment analysis (see Methods) gave a total of 2335 and 354 RNA targets in HCT116 and Saos-2 cells, respectively (Figure 2A; Supplementary Tables S2 and S3), all enriched at least 2-fold after Wig-1 pulldown (log2 fold change (log_2_FC) > 1). We plotted the mean counts of each sequenced transcript in the input sample versus the RIP sample in HCT116 and Saos-2 cells and observed that Wig-1-bound RNAs (shown in red in Figure 2B–2C) are distributed over the entire expression level range. Thus, the binding of Wig-1 to its associated mRNAs does not correlate with the expression level of the target mRNA. Despite differences in the variety and copy number of transcripts expressed between these two cell types, we found 286 Wig-1-bound mRNAs that were common for both cell lines, and these were used for further analysis (Supplementary Table S4).

Moreover, we integrated the data from our previous study including a microarray analysis in HCT116 cells after Wig-1 knockdown by siRNA [5], and combined it with the RIP-seq data from the same cells presented here. We found 209 Wig-1 bound transcripts that are also affected by Wig-1 knockdown with a more than 4-fold difference in at least two out of the three replicates (Supplementary Table S5). Interestingly, 25 transcripts were downregulated (12%) while 184 transcripts were upregulated (88%) upon Wig-1 knockdown, suggesting that Wig-1 primarily acts as a destabilizing RNA-binding protein.

### Gene ontology and network analysis

To determine the functions of the Wig-1-associated RNAs we grouped them into molecular function (MF), biological process (BP) and cellular compartment (CC) by Gene Ontology (GO) term using the gene set enrichment analysis of the DAVID bioinformatics resource tool [23]. Two hundred sixty-one genes (out of 286) were annotated. The significant GO annotations are reported in Supplementary Table S6 (cutoff at *p*-value < 0.05 by Fisher Exact test). As for MF, a total of 90 and 30 genes from our list corresponded to proteins with catalytic or transferase activity respectively. Among the BP categories, “cellular metabolic process” (corresponding to 45% of the analyzed genes), “protein localization” (9%) and “cell cycle” (5%) were enriched. In terms of CC, components of the membrane-bounded organelle (59%), endoplasmic reticulum (14%), and mitochondrion (11%) are over-represented in our list of Wig-1-bound mRNAs (Supplementary Table S6).

To further characterize the list of Wig-1 associated transcripts we performed network enrichment analysis (NEA) and identified as many as 300 pathways as enriched at significance level NEA false discovery rate (FDR) < 0.01 (Supplementary Table S7). We decided to focus on the pathways from the Reactome database [24], as this database provides more precise molecular details of every event in a pathway and links to references that contain experimental verification of those events. At the specified significance threshold, 133 of the Reactome pathways were enriched. Table 1 presents the top 20 enriched pathways with respective *p*-values, while the complete list is included in Supplementary Table S8. Interestingly, the top five pathways were all associated with the cell cycle (Table 1). For example, 188 genes from our Wig-1-associated list were connected in the network to the “Cell Cycle Mitotic” pathway (which consisted a total of 268 genes). The software also grouped individual pathways into clusters, and the “Cell Cycle” cluster was indeed the top cluster detected in our analysis. The other enriched pathways belonging to the “Cell Cycle” cluster are “Mitotic M-M/G1 Phases”, “Mitotic Prometaphase”, “G1/S Transition”, “S Phase” and “Cell Cycle Checkpoints”. Other top pathways for Wig-1-bound targets are “HIV Infection”, “Synthesis of DNA” and “Metabolism of RNA” (Table 1).

**Table 1:** Pathways of the Reactome database that were highly enriched after network enrichment analysis (NEA) of the whole list of 286 Wig-1 bound targets and relative network enrichment *p*-value

	Pathway	Network enrichment *p*-value
1	Cell Cycle Mitotic	0.00E+00
2	Mitotic M-M/G1 Phases	0.00E+00
3	Mitotic Prometaphase	1.39E-80
4	G1/S Transition	2.30E-63
5	S Phase	4.00E-63
6	HIV Infection	8.04E-63
7	Cell Cycle Checkpoints	4.08E-60
8	Host Interactions Of HIV Factors	1.06E-53
9	Synthesis Of DNA	1.17E-51
10	Metabolism Of RNA	2.83E-48
11	Regulation Of APC Activators Between G1/S And Early	5.29E-48
12	NEP/NS2 Interacts With The Cellular Export Machinery	3.67E-45
13	Rev Mediated Nuclear Export Of HIV1 RNA	1.65E-44
14	Transport Of Mature mRNA Derived From An Intron Containing	2.51E-43
15	Transport Of Ribonucleoproteins Into The Host Nucleus	2.95E-43
16	Transport Of The SLBP Independent Mature mRNA	3.46E-43
17	Nuclear Import Of REV Protein	7.60E-43
18	Late Phase Of HIV Life Cycle	2.26E-41
19	Dna Replication Pre Initiation	2.03E-40
20	HIV Life Cycle	2.24E-40

We also performed NEA on the list of genes whose mRNA was bound and regulated by Wig-1 in HCT116 cells (Supplementary Table S9). Table 2 shows that Wig-1 bound transcripts whose expression levels were downregulated after Wig-1 knockdown (25 out of 209 genes) were also enriched in pathways included in the “Cell Cycle” cluster. In contrast, those whose expression levels are upregulated after Wig-1 knockdown (184 out of 209) are enriched in pathways associated with transcription, DNA repair and cellular immune response.

**Table 2:** Network enrichment analysis (NEA) was performed on bound and regulated Wig-1 targets as assessed by RIP-Seq and microarray analysis upon Wig-1 knockdown, respectively The top 10 enriched pathways from the downregulated (green) and upregulated (red) Wig-1 targets and the relative *p-values* from the Reactome database are shown

		Pathway	Network enrichment *p*-value
Downregulated genes after Wig-1 knockdown	1	Cell Cycle Mitotic	2.21E-31
	2	Mitotic M-M/G1 Phases	9.67E-31
	3	Mitotic Prometaphase	1.27E-20
	4	Cell Cycle Checkpoints	9.00E-17
	5	G1/S Transition	2.83E-15
	6	Regulation Of APC Activators Between G1/S And Early Anaphase	1.46E-13
	7	Cdc20:Phospho-APC/C mediated degradation of Cyclin A	1.60E-13
	8	DNA Replication Pre-Initiation	1.65E-12
	9	S Phase	2.81E-12
	10	Host Interactions Of HIV Factors	3.26E-12
Upregulated genes after Wig-1 knockdown	1	Generic Transcription Pathway	7.39E-17
	2	TAK1 activates NFkB by phosphorylation and activation of IKKs complex	3.12E-13
	3	Viral dsRNA:TLR3:TRIF Complex Activates RIP1	3.83E-13
	4	DNA Repair	5.08E-13
	5	Global Genomic Nucleotide Excision Repair	5.98E-13
	6	Cell Cycle Mitotic	1.67E-12
	7	Nucleotide Excision Repair	1.74E-12
	8	Transcription Coupled Nucleotide Excision Repair	9.23E-12
	9	Toll Like Receptor 3 Cascade	3.45E-11
	10	TRAF6 Mediated Induction Of The Antiviral Cytokine IFN Alpha Beta Cascade	3.82E-10

### Validation of Wig-1-bound mRNAs identified by the RIP-Seq analysis

In order to validate Wig-1-bound mRNAs, we selected nine Wig-1-associated mRNAs enriched in both HCT116 and Saos-2 RIP-Seq experiments: MAD2L1 (Mad2 Mitotic Arrest Deficient-Like 1), MTHFD2 (Methylenetetrahydrofolate Dehydrogenase (NAPD+ Dependent) 2), CCNG1 (Cyclin G1), EIF4E (Eukaryotic Translation Initiation Factor 4E), CHEK1 (Checkpoint Kinase 1), RMI1 (RecQ Mediated Genome Instability 1), HIF1A (Hypoxia Inducible Factor 1, Alpha), AMD1 (Adenosylmethionine Decarboxylase 1) and CAV1 (Caveolin 1) (Table 3). The selection was based on 1) inclusion in at least one of the top three enriched pathways as determined by network enrichment analysis, 2) the RIP-Seq enrichment score: with log_2_FC value ranging from 1.3 to 2.6, these targets were neither among the most enriched targets or the bottom group just above our cutoff of log_2_FC > 1 (corresponding to a twofold enrichment) and 3) presence of AREs in their 3′UTRs as determined by RSAT (Regulatory Sequence Analysis Tools).

**Table 3:** List of Wig-1-bound RNAs chosen for validation with information regarding their enrichment values in the RIP experiment shown as the log value of the fold-change (logFC) in both HCT116 and Saos2 cells, and the number of AUUUA pentamers found in the 3′UTRs by RSAT software

Target	Description	LogFC HCT116	LogFC Saos2	AUUUA count
MAD2L1	MAD2 mitotic arrest deficient-like 1	1.92	2.15	3
MTHFD2	Methylenetetra-hydrofolate dehydrogenase (NADP+ dependent) 2	1.36	1.29	5
CCNG1	Cyclin G1	2.59	2.14	3
EIF4E	Eukaryotic translation initiation factor 4E	2.06	1.82	3
CHEK1	Checkpoint kinase 1	1.8	1.4	2
RMI1	RecQ mediated genome instability 1	2.43	1.89	9
HIF1A	Hypoxia inducible factor 1, alpha subunit	2.03	1.57	7
AMD1	Adenosylmethionine decarboxylase 1	2.16	2.17	6
CAV1	Caveolin 1	1.55	1.56	3

**Figure 3:**
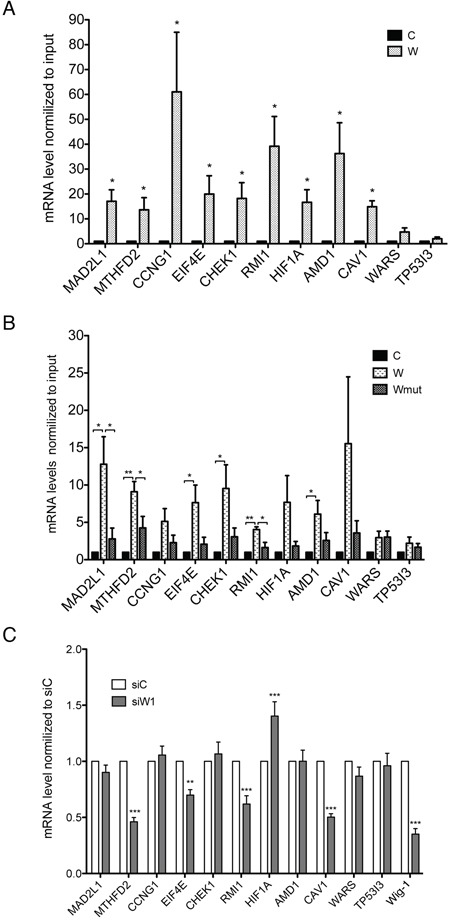
RIP-Seq target validation RIP was performed in HCT116 cells transiently transfected with Flag (C) or Flag-tagged Wig-1 (W) **A.** or in Saos-2 TetON cells (control, C) or Saos-2 TetON cells stably expressing either Flag-tagged wt Wig-1 (W) or a Flag-tagged zinc-finger 1 point mutant Wig-1 (Wmut) **B.** Wig-1 was precipitated with anti-Flag beads and the bound RNA was extracted. mRNA levels of the indicated targets were then determined by qRT-PCR and normalized to GAPDH mRNA and input. Data shown are mean ± SEM (*n* = 4 for HCT116 and *n* = 3 for Saos-2). *, *p*-value < 0.05 and **, *p*-value < 0.01 by Dunnett's multiple-comparison test. **C.** Wig-1 knockdown (siW1) leads to decreased levels of MTHFD2, EIF4E, RMI1 and CAV1 mRNAs and increased levels of HIF1A mRNA in HCT16 cells as assessed by qRT-PCR. Target mRNA levels were normalized to GAPDH mRNA, and control transfected cells (siC) are set to 1. Bars indicate mean ± SEM, *n* = 4. **, *p*-value < 0.01 and ***, *p*-value < 0.001 by Dunnett's multiple-comparison test.

We performed RIP and assessed the levels of these mRNA targets by qRT-PCR. We also included two negative controls for the validation: the tumor protein p53 inducible protein 3, TP53I3, and the tryptophanyl-tRNA synthetase, WARS, both of which were detected but not enriched after Wig-1 RIP in both HCT116 and Saos-2 cells. Supplementary Figures S1 and S2 show the FPKM values calculated from the triplicates RIP-Seq experiments per sample for the selected mRNAs in HCT116 and Saos-2 cells. As shown in Figure 3A, all nine targets were validated in HCT116 cells. For the validation in Saos-2 cells, the results for MAD2L1, MTHFD2 and RMI1 confirm significant binding to Wig-1 as compared both to empty vector control and Wig-1 mutant negative control, while EIF4E, CHEK1, and AMD1 shows significant binding to Wig-1 vs control, but not vs mutant Wig-1. For the remaining three targets (CCNG1, HIF1A and CAV1), Wig-1 binding was not statistically significant in Saos-2 cells (Figure 3B). The two negative controls TP53I3 and WARS were not pulled down by Wig-1 in either cell line.

To verify whether these targets are also regulated by Wig-1, we knocked down Wig-1 using siRNA (siW1). Our data showed that Wig-1 knockdown leads to decrease levels of MTHFD2, EIF4E, RMI1, and CAV1, while we observed an increase in the levels of HIF1A mRNA. The remaining targets showed little or no regulation, including the negative control WARS and TP53I3 (Figure 3C). Interestingly, MTHFD2 was also detected as downregulated in our previous microarray study conducted in HCT116 cells upon Wig-1 knockdown [5]. In contrast, Wig-1 knockdown resulted in increased expression of HIF1A mRNA (Figure 3C). However, Wig-1 knockdown did not lead to any significant changes in the amounts of HIF1A protein but rather a slight decrease (Supplementary Figure S6), presumably because HIF1A is tightly regulated at the protein level (see Discussion).

### 3′UTR sequence analysis of Wig-1 target mRNAs

**Figure 4:**
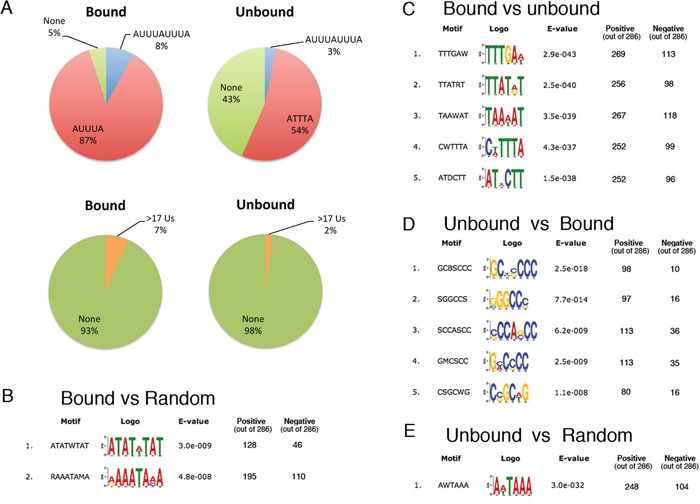
AU-rich elements in the 3′UTR of Wig-1 target mRNAs **A.** Left panel: Circle diagrams showing the percentages of the 3′UTR sequences of the 286 enriched Wig-1 RNA targets (defined as bound, see main text) that contain ARES (determined using RSAT, see materials and methods for details), upper panel, or have stretches of 17 continuous Us or more (lower panel). Right panel: As negative controls, The same analyses were performed on 3′UTRs from 286 RNAs present but not enriched after Wig-1 pulldown (defined as unbound and used as negative controls, see main text). **B.** RNA motifs enriched in the 286 Wig-1-associated RNAs versus a random control group generated by shuffling the nucleotide sequence of each 3′UTR, determined using Discriminative Motif analysis by DREME (see materials and methods for details). **C.** Same as in B, but instead of the random group, the group of 286 unbound RNAs (defined in A) was used as control. **D.** and **E.** represent controls for the motif analysis: D. show the reverse analysis of C: the Unbound group is compared to the bound group E. The unbound control group is compared to their corresponding shuffled, “random” sequences (similar to B for the bound RNAs).

Next, we set out to investigate if the mRNAs bound by Wig-1 share any particular sequence elements. The interaction of Wig-1 and its target mRNAs has been shown to be mediated predominantly by ARE motifs (p53, N-Myc and FAS [5–7]) located in the 3′UTR of the transcripts, with the exception of p21, where Wig-1 interacts with a different sequence in the p21 3′UTR [8]. These observations are in accordance with the notion of 3′UTRs as hubs for regulatory events [25]. Therefore, we restricted our analysis of enriched elements to the 3′UTR of the bound targets. We started by scanning our list of 286 targets for the occurrence of ARE motifs using the RSAT tool (http://www.rsat.eu/) [26]. As negative control group we included the 3′UTRs of 286 mRNAs that were detected in our sequencing data (and thus clearly expressed in our cell lines) but were not enriched after Wig-1 RIP (Supplementary Table S10). As shown in Figure 4A, 8% of the Wig-1-bound mRNAs contains 2 consecutive pentamers (AUUUAUUUA) as compared to 3% of the unbound control mRNAs (*p*-value = 0.018, two-sided proportionality test function in R), while the canonical pentamer (AUUUA) is present at least once in 87% of the Wig-1-bound mRNAs and in 54% of the unbound control group (*p*-value = 2.2e-16). Moreover, 43% of the unbound control group does not contain any pentamer, while the corresponding number for the Wig-1-bound group is 5%. Another sub-group of AREs is classified as stretches of uridines (Us). Using the same program, we found that 7% of the Wig-1-bound and 2% of the unbound control mRNAs contain a U-stretch (defined as a polyU stretch equal to or longer than 17 nucleotides) (*p*-value= 0.014). We conclude that the ARE motifs are significantly enriched in the 3′UTR of the Wig-1-associated RNAs, thus supporting previous results demonstrating that Wig-1 is an ARE-BP.

To confirm our results and to allow identification of previously unknown motifs, we used the DREME algorithm from the MEME suite of sequence analysis tools for a more comprehensive and unbiased approach [27]. We compared the 3′UTRs from the positive group of 286 Wig-1-bound mRNAs with the 3′UTRs from the shuffled sequences of these 3′UTRs (“random set”) or the group of 3′UTRs from 286 unbound mRNAs described above (“unbound set”) (Supplementary Table S10). Consistent with a preference of Wig-1 for ARE-containing mRNAs, the consensus motifs found in the Wig-1-bound mRNAs are highly enriched in A and U compared either to the random set (Figure 4B) or to the negative unbound set (Figure 4C) (cutoff at *e*-value < 10^−5^). In contrast, the opposite analysis and comparison of 3′UTRs from the unbound mRNAs to the bound mRNAs revealed that 3′UTRs from unbound mRNAs are rich in G and C (Figure 4D) and show no enrichment for AU-rich elements. In addition, when looking at the GC content of the analyzed sequences, we found that mRNAs bound by Wig-1 had 3′UTRs with an average of 33% GC-content, while the 3′UTRs from the unbound sets contain 46% GC. In accordance, the average GC content in all human 3′UTRs has been estimated to 45% [28]. Moreover, the AUUUA pentamer is found as the first top motif in the Wig-1-bound group compared to its random set (motif 1, panel B; *e*-value = 3.0e^−09^) and among the top motives (motif 4, panel C, *e*-value = 4.3e^−37^) when compared to the unbound set. As expected, we note that both the Wig-1-bound and unbound sets were enriched for the cleavage and poly-A signal (AAUAAA) when compared to the random control (Figure 4B, 4E). Altogether these data clearly confirm that Wig-1 preferentially binds to mRNAs that contain AREs and/or are generally AU-rich.

### Secondary structure analysis of Wig-1-bound mRNAs

**Figure 5:**
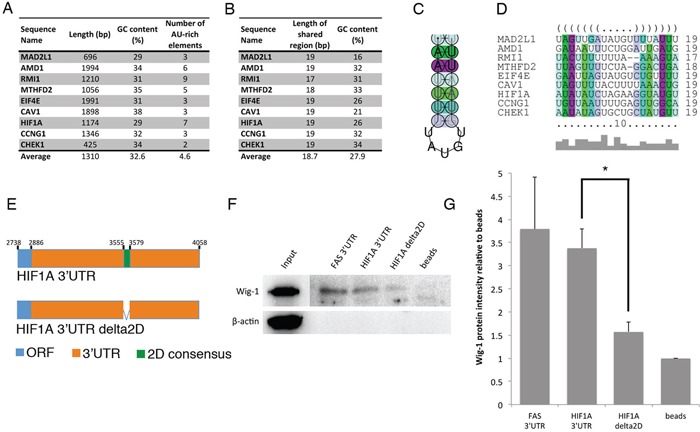
Identification of a predicted stem-loop structure within Wig-1-bound mRNAs **A.** The 3′UTR sequences of the nine validated Wig-1-bound mRNAs were compared to identify common features. Length, GC content and ARE element contents of the 3′UTRs are indicated. **B.** LocARNA software was used to identify a shared structural motif (referred to as the consensus 2D motif) (see materials and methods for details). Length and GC content for the consensus 2D motif from each of the 9 3′UTRs included in the analysis are listed. **C.** Consensus structure of the consensus 2D motif, predicted by RNAalifold. **D.** Alignment of the consensus 2D motifs from the 9 3′UTRs included in the analysis shown in dot-bracket notation, where base pairs in the stem loop are indicated by corresponding opening and closing brackets and unpaired nucleotides with dots. Base pairs in **C.** and **D.** are colored according to the Vienna RNA conservation coloring scheme [70] where each color represent the number of different base pairs supporting that pair. Red marks pairs have no sequence variation, therefore 100% sequence conservation; yellow, green, turquoise, blue, and violet mark pairs have 2, 3, 4, 5 or 6 different types of pairs, respectively (out of the six possible base pairs: C-G, G-C, A-U, U-A, G-U or U-G). Color saturation indicates structural conservation of the base pair and it decreases with the number of incompatible base pairs. **E.** Model of the HIF1A constructs used for biotin pulldown (see materials and methods for details). Constructs are not drawn to scale. **F.** Biotin pull-down assay using the above mentioned probes or a FAS full-length 3′UTR probe as positive control [5] followed by Western blotting for Wig-1 shows that Wig-1 binds to the FAS construct and the HIF1A construct containing the consensus 2D motif, but not to the HIF1A construct lacking the consensus 2D motif. A representative image from one of three independent experiments is shown. **G.** Wig-1 protein level quantification from three independent experiments using ImageJ densitometry software is shown as mean ± SEM; *n* = 3; * *p*-value < 0.05.

We next asked whether the Wig-1-bound mRNAs share any particular structural motifs. To this end we characterized the 3′UTR sequences of the nine validated targets (CCNG1, RMI1, CHEK1, MTHFD2, CAV1, AMD1, HIF1A, MAD2L1 and EIF4E) in terms of sequence content and secondary structure. These sequences range in length between 425 and 1994 nts, have an average GC content of 32.6%, which is lower than the average GC content of human 3′UTR sequences (calculated to be 45% [28]), and a variable number of putative AREs (between two and nine) (Figure 5A). By simultaneous folding and alignment of the sequences using the software LocARNA, we predicted 9 regions, one in each sequence, sharing both sequence and 2D structure similarity. These regions, on average, are 18.7 nts long and have a GC content of 27.9% (Figure 5B), almost one fourth lower than the average GC content of the whole 3′UTR sequences. The consensus sequence, UAAUUUUUAUGUGUAAUUU, derived from their multi-alignment has a GC content of 11% and folds to form a hairpin, which we will refer to as the consensus 2D motif (Figure 5C, 5D).

To further characterize the nine bound 3′UTR sequences we predicted their complete 2D sequence structures using RNAfold software (Supplementary Figure S3A–S3I). We assessed the spatial arrangement of their putative AREs and consensus 2D motifs in their primary sequences and in their 2D structures. In the primary sequence, the shared regions are close to an ARE (less than 165 nts away) in eight cases out of nine (Supplementary Figure S4 and Supplementary Figure S5). The exception is CCNG1 where the consensus 2D motif is 668 nts away from the closest ARE. However, the CCNG1 3′UTR 2D structure predicts that its consensus 2D motif is spatially close to one of its AREs (Supplementary Figure S3A).

For validation purposes, we tested the consensus 2D motif in HIF1A and MTHFD2 3′UTRs, which correspond to the sequence AUAUAUCUAGAAGGUAUGU and UUAGUUUUCUAGGACUGA, respectively (Figure 5D). In both cases, we performed *in vitro* pull-down assays using biotinylated RNA probes containing the whole candidate 3′UTR or the candidate 3′UTR with the consensus 2D motif deleted (Figure 5E). Additionally, we used the full length FAS 3′UTR as a positive control [5]. The biotinylated probes were incubated with a lysate from HCT116 cells overexpressing Flag-tagged Wig-1. After pull-down of RNA with streptavidin-coated beads (Figure 5F, 5G), Flag-Wig-1 was detected by Western blotting. In the case of HIF1A, Wig-1 was pulled down with the full length HIF1A 3′UTR probe and with the full length FAS 3′UTR, but not with the HIF1A 3′UTR probe with consensus 2D motif deletion (HIF1A delta2D). This result indicate that the consensus 2D motif found in the HIF1A 3′UTR predicted by LocARNA analysis is crucial for Wig-1 binding to the HIF1A 3′UTR. However, in the case of MTHFD2, Wig-1 was detected with the same intensity in the pull-down with the full length FAS 3′UTR, the full length MTHFD2 3′UTR probe, and the MTHFD2 3′UTR probe with consensus 2D motif deletion (MTHFD2 delta2D). (Supplementary Figure S7A–S7B). These results indicate that the consensus 2D motif found on MTHFD2 3′UTR predicted by LocARNA analysis is not critical for Wig-1 binding to the MTHFD2 3′UTR. It is possible that other regulatory elements are important for the binding of Wig-1 to MTHFD2 mRNA, including one or several of the 5 AREs in the 3′UTR.

## DISCUSSION

Modern large-scale technologies allow us to probe the entire target mRNA repertoire of RNA-binding proteins in one experiment. The challenge is now to integrate all this information and build accurate models of cellular RNA-RBP networks, which has been done for a number of known RBPs [29–31]. For Wig-1, global analysis of mRNA targets has not been performed previously. Here we show the results of a genome-wide study performed in HCT116 and Saos-2 cells aiming at characterizing the Wig-1-interacting transcriptome. We identified 2335 and 354 enriched mRNA targets in HCT116 and Saos-2 cells, respectively. In agreement with our previous study [5], FAS mRNA was found enriched in HCT116 cells (Supplementary Table S2). The reason for the larger number of targets in HCT116 cells is most likely due to higher reproducibility between the three HCT116 experimental replicates. This higher reproducibility was evident in terms of the amount of sequenced material as compared to Saos-2 (Supplementary Table S1). In addition, the Saos-2 experiment was more stringent because in this experiment, mRNAs were only considered bound by Wig-1 if they were enriched both compared to empty control (no Wig-1) and to an RNA-binding deficient mutant Wig-1, which most likely diminished unspecific background.

We found that 286 RNAs were shared between the two lists of targets bound in the two cell lines and we used this common list for further analysis. The network enrichment analysis indicated that Wig-1 targets are strongly linked to the Cell Cycle pathway. This is not surprising, as we have previously shown that Wig-1 regulates cell cycle progression and cellular survival [5, 6]. We found that Wig-1 silencing enhances apoptosis and reduces cell cycle arrest in response to cellular stress in HCT116 cells by regulation of the proapoptotic FAS and cell cycle arrest 14–3-3sigma mRNAs. Moreover, Wig-1 might facilitate cell cycle progression and survival post-stress by sustaining levels of growth-promoting mRNAs such as N-Myc [6]. Interestingly, the “HIV infection” pathway was also enriched in the NEA analysis. A recent publication showed that INFβ decreased Wig-1 levels in 4 different cancer cell lines as well as in mouse B-cells [32]. This suggests an involvement of Wig-1 in the cellular response to viral infection and should be investigated further.

Moreover, integration of the data from the RIP-Seq in HCT116 cells with our gene expression data on HCT116 cells upon Wig-1 knockdown [5] resulted in a list of 209 mRNAs that are both bound and regulated by Wig-1. Of these, 88% are upregulated upon Wig-1 knockdown and 12% are downregulated. The NEA on this group of targets indicated that Wig-1 increases the stability of mRNAs associated with the cell cycle pathway, while promoting the degradation of mRNAs associated with transcription, DNA repair and cellular immune response. Thus, these data suggest that Wig-1 is predominantly a destabilizing RNA-binding protein.

We chose nine candidates from the list of bound mRNAs common for both HCT116 and Saos-2 (CCNG1, RMI1, CHEK1, MTHFD2, CAV1, AMD1, HIF1A, MAD2L1 and EIF4E) for further validation, along with two additional mRNAs that were detected in the RNA-Seq but not enriched in the Wig-1 RIP sample as negative controls (TP53I3 and WARS). The validation confirmed the RIP-Seq data for all the targets in HCT116 cell line. For Saos-2 we confirmed six targets out of nine as compared to negative control: MAD2L1, MTHFD2, RMI1, EIF4E, CHEK1 and AMD1. For CCNG1, HIF1A and CAV1 the enrichment compared to Wig-1 mutant control was not statistically significant. This validation rates of 100% in HCT116 cells and 67% in Saos-2 cells compare well to results reported by others [29–31]. The fact that validation compared to the mutant Wig-1 was poorer could be due to the greater variation (larger standard deviation, hence lower statistical significance) between replicates observed for Saos-2. By knocking down endogenous Wig-1 in HCT116 cells and analyzing the levels of bound targets, we demonstrated that Wig-1 regulates the mRNA levels of five out of the nine validated candidates. This number of targets regulated at the mRNA level is particularly striking since ARE-BPs are known to affect also many other aspects of their target mRNAs other than RNA stability. Examples include regulation of splicing, maturation, transport, storage and translation, as shown for the mammalian Hu/elav family of ARE-BPs [33]. Therefore, not all *bona fide* Wig-1 targets are expected to be regulated at the level of mRNA stability, and other regulatory processes should be considered and further investigated.

Wig-1 knockdown by siRNA led to decreased levels of MTHFD2 (confirming our previous data from the microarray analysis on HCT116 cells [5]), EIF4E, RMI1, and CAV1 mRNA, and increased levels of HIF1A mRNA. However, we did not observe any significant changes in HIF1A protein levels after Wig-1 knockdown. This could be explained by the fact that while HIF1A mRNA is constitutively and ubiquitously expressed, regardless of the level of oxygen tension, HIF1A protein has a very fast turnover under normoxia [34]. Moreover, a large number of factors are involved in the tight regulation of HIF1A protein stability, particularly the ubiquitin ligases Cul2 and VHL [35], whose RNAs are among the Wig-1 bound targets found in this study (Supplementary Table S2).

The fact that these five genes (MTHFD2, EIF4E, RMI1, CAV1 and HIF1A) are all very relevant in tumor biology further emphasizes a role of Wig-1 in the regulation of cell cycle and cell proliferation, as well as tumor onset, progression and metastasis. For example, MTHFD2 is a mitochondrial enzyme fundamental in one-carbon metabolism, a metabolic system recently implicated in rapid cancer cell proliferation [36]. Indeed, MTHFD2 is consistently overexpressed in tumors as compared to normal adult cells [37]. MTHFD2 knockdown in HCT116 cells was associated with reduced cancer cell proliferation and marked cell death [37], consistent with the effect that we have previously observed after Wig-1 knockdown in these cells [5].

Caveolin-1 (CAV1) is a membrane protein involved in signal transduction, as well as in numerous other cellular processes including cell cycle, vesicular transport, cholesterol homeostasis and cell migration [38]. During the early stages of tumor progression, CAV1 negatively controls cell cycle progression and restrains cell proliferation, whereas accumulating evidence suggests that CAV1 has an opposite role in advanced stages of cancer, promoting cell growth and metastasis [39]. Similarly to MTHFD2, knockdown studies in HCT116 cells showed that CAV1 is anti-apoptotic, inhibiting Bax-dependent cell death [40].

The eukaryotic translation initiation factor 4E (eIF4E) is a key player in translational control. Silencing of eIF4E slows down proliferation, and causes arrest of the cell cycle in the G0/G1 phase, as well as increased apoptosis in many cancer types [41–43]. An additional study reported that EIF4E knockdown in HCT116 cells leads to increased p53-mediated apoptosis due to decreased translation of MDM2 by EIF4E and consequent p53 stabilization [44]. Interestingly, HuR has been reported to bind and stabilize EIF4E mRNA by an ARE in the 3′UTR [45].

RMI1 is an essential member of the RecQ-topoisomerase III complex that has a role in DNA replication and the replication stress response, and is involved in suppressing sister chromatid exchange and tumorigenesis [46]. Thus, it is conceivable that the p53 target Wig-1 might promote genomic stability through the stabilization of RMI1, analogous to the observed Wig-1-mediated stabilization of p53 mRNA.

Lastly, HIF-1 alpha (HIF1A), a transcription factor that regulates key genes involved in the glycolysis pathway [47], is upregulated at the mRNA level after Wig-1 knockdown, suggesting that Wig-1 enhances its degradation. Similarly, two other well studied ARE-BPs, HuR and TTP, have been reported to downregulate HIF1A expression [48, 49] thus preventing excessive HIF1A protein accumulation during prolonged hypoxia [49]. Interestingly, a study performed in rats showed that the rat Wig-1 homolog (PAG608) is transcriptionally activated by p53 after brain ischemia [50]. In a situation such as ischemia (which causes acute and strong hypoxia), HIF1A activity has been associated with cell death by direct interaction with the p53 protein [51], whereas in solid tumors (mild hypoxia), it is associated with cell survival and proliferation [52]. Thus, we speculate that Wig-1 modulation of HIF1A levels depends on the physiopathological context and may be tissue specific.

In line with previous reports on Wig-1 bound mRNAs, our current data demonstrate that Wig-1 can regulate targets both positively and negatively, and affect both pro- and anti-growth factors. This is in agreement with what has been reported for other ARE-BPs such as AUF1 and HuR [53–56]. Nonetheless, taken together, our findings support a pro-survival role of Wig-1, in agreement with the literature [5, 57]. We previously showed that Wig-1 modulates the p53 response to stress through the regulation of p53 itself, FAS and, indirectly, 14–3-3sigma mRNAs [5, 7]. We suggest that Wig-1 could also be involved in genome stability maintenance through the positive regulation of p53 and RMI1. In addition, our results suggest that Wig-1 takes part in regulation of cell cycle and cell proliferation through important oncogenes such as N-Myc [6], as well as MTHFD2, EIF4E, CAV1 and HIF1A (this study). In summary, the outcome of the regulation of these targets is in agreement with the phenotype that results from Wig-1-mediated regulation of previously identified targets. In fact, a positive regulation of MTHFD2 and EIF4E would support cell proliferation, in accordance with the effect of Wig-1 regulation on N-Myc [6]. Additionally, the stabilization of CAV1, MTHFD2 and EIF4E would also lead to decreased apoptosis, consistent with the effect on the destabilization of FAS mRNA by Wig-1 [5].

The RNA-immunoprecipitation method used here results in the pull-down of the entire target transcripts, not allowing the identification of the specific RNA sequence required for the RNA-protein interaction. Alternatively, the global RNA interaction partners of a protein can be assayed by CLIP-seq (crosslinking immuno-precipitation and sequencing), which utilizes *in vivo* UV crosslinking in order to create covalent and irreversible bonds between RNA and protein. The bound RNA is then fragmented, resulting in recovery and sequencing of only the portion of the RNA that is bound by the protein, which allows for exact determination of the RNA sequence bound by the protein. The crosslinking also enables precipitation of transient interactions. However, because CLIP-Seq allows identification of also transient and potentially unspecific interactions, while RIP-Seq generally identifies stronger and more stable interacting RNA partners that remain bound without crosslinking through purification and washes, we chose to use RIP-Seq rather than CLIP-Seq, favoring the identification of potentially stronger interactions with Wig-1 at the cost of less information on the exact motives bound by Wig-1. Analyzing these strong Wig-1 bound candidates for common sequence elements that may explain their interaction with Wig-1, we show that Wig-1 preferentially binds to mRNAs that contain AREs in their 3′UTR and 95% of the mRNAs bound by Wig-1 contain at least one AUUUA pentamer. Moreover, de novo motif enrichment analysis revealed that apart from classical AU-rich elements, Wig-1 also favors the binding of motifs that are generally rich in A and Us compared to an unbound control groups. Since protein/RNA interaction may occur over a wide range of affinities, depending on the involvement of both primary and secondary structure elements, we analyzed predicted secondary structures generated from the whole 3′UTR sequences from Wig-1-bound RNAs using the LocARNA software. Our data show that shared consensus secondary structures (the consensus 2D motif) for Wig-1-interacting RNAs have a very low GC content (11%). Interestingly, this consensus 2D motif is localized, in the majority of the cases (eight out of nine), in close proximity to an AU-rich element in the primary sequence of the 3′UTR or – in the ninth case - in the secondary folded structure on the 3′UTR. Moreover, we could experimentally validate that the consensus 2D motif on HIF1A 3′UTR is critical for Wig-1 binding. However, the relevance of the *in silico* predicted structural elements for binding to other Wig-1 targets need to be tested carefully. As shown in this study, the same 2D consensus element was not required for binding of Wig-1 to MTHFD2 3′UTR. It is not fully understood to what degree RNA secondary structures, as opposed to sequence motifs such as AREs, are important for Wig-1 binding to RNA. It is conceivable that Wig-1 RNA binding is affected by the recognition of a secondary RNA structure, while the effect of Wig-1 on mRNA stability could be conferred by a specific RNA sequence motif.

In conclusion, our study provides a novel and comprehensive view of the RNA-binding properties of Wig-1 and defines more precisely the Wig-1-RNA interaction network. Our data confirm that Wig-1 is an ARE-BP involved in regulation of cell cycle progression and cell proliferation. We also show that Wig-1 is primarily a destabilizing RNA-binding protein. We significantly expand the list of known Wig-1 targets, and provide novel insights into preferred Wig-1 RNA binding motifs. Importantly, we identify a putative 2D motif for Wig-1 mRNA binding to HIF1A mRNA. Since Wig-1 is a target of the p53 transcription factor, our results should provide a better understanding of p53-mediated tumor suppression through its target Wig-1, extending the frontiers of gene expression control from the transcriptional to the post-transcriptional level.

## MATERIALS AND METHODS

### Cell culture and transfection

HCT116 and Saos-2 cells were grown in IMDM supplemented with 10% fetal bovine serum (GIBCO, Grand Island, NY, USA) or fetal bovine serum with reduced tetracycline (Clontech, Mountain View, CA, USA) for the tetracycline-regulated Wig-1-expressing Saos-2 cells [4], 2 mM L-glutamine (Life technologies, Stockholm, Sweden) and 2.5 μg/ml Plasmocin (InvivoGen, San Diego, CA, USA) in 5% CO_2_ at 37°C. Cell were transfected with 10 nM all star negative control (siC) or Wig-1_1 (siW1) siRNAs using HiPerFect (Qiagen) according to manufacturers protocol. Transient plasmid transfections were performed using PEI reagent according to standard protocol.

### RIP, qRT-PCR and western blotting

HCT116 or Saos-2 cells were harvested 24 h after transfection or 48 h after induction with 1 μg/ml doxycycline, respectively. RIP and qRT-PCR was performed as described in [7]. Briefly, RIP was performed in HCT116 cells transiently transfected with pCMVtag2b or pCMVtag2bhWig-1 and in Saos-2 TetON cells without insert or Saos-2 TetON cells stably transfected with either Flag-tagged wt Wig-1 or a Flag-tagged Wig-1 zinc-finger 1 point mutant that cannot bind to RNA [3]. Flag-tagged Wig-1 was precipitated from cell lysates with anti-Flag beads (Sigma-Aldrich), and bound RNA was purified as described [7] and used for library preparation or quantified by qRT-PCR. List of the TaqMan probes are included in Supplementary Table S11. Cell lysate aliquots from the RIP experiments from input and RIP samples were collected and used for Western Blot analysis, loaded on SDS/10% polyacrylamide gels (Invitrogen) in loading buffer and reducing agent (Invitrogen) and run in Mops buffer (Invitrogen). Proteins were blotted to nitrocellulose membranes using the iBlot Dry Blotting System (Invitrogen) and probed with antibodies against Wig-1 (1:1,000) [4] HIF1A (1:1,000; Novus Biologicals), β-actin (1:5,000; Sigma–Aldrich) and GAPDH (1:8000, Santa Cruz). Primary antibodies were detected by using HRP-conjugated secondary antibodies (GE Healthcare) and Super Signal West Femto Maximum Sensitivity Substrate (Thermo Scientific) with a CCD camera (Fujifilm). For HIF1A protein detection by Western blotting, 48 hours post siRNA transfection, HCT116 cells were treated with 20 μM MG132 proteasome inhibitor (Sigma-Aldrich) for 8 h, trypsinized, washed in PBS, and re-suspended in hypotonic buffer (10 mM Tris-Cl pH 7.5, 2.5 mM MgCl2 and 1.5 mM KCl). Samples were placed on ice for 10 minutes. Detergent was added to a final concentration of 0.5% Triton-X. Samples were vortexed and centrifuged at 10,000 x g for 2 min. Supernatants (cytosolic fraction) were removed and pellets containing nuclear fraction were re-suspended in lysis buffer containing 50 mM Tris-Cl pH 7.5, 5 mM EDTA, 0.15M NaCl, 1% Triton-X, 0.5% Deoxycholate, 1 mM DTT and protease inhibitor cocktail (Sigma-Aldrich). Samples were vortexed thoroughly and spun at 14,000 × g for 30 min to remove insoluble fraction. Sample proteins concentrations were determined using Bradford assay and blotted to nitrocellulose membranes as above.

### Biotin pulldown assay

The pCRII-FAS-3′UTR, pCRII-HIF1A-3′UTR, pCRII-HIF1A-3′UTR-delta2D, pCRII-MTHFD2–3′UTR and pCRII-MTHFD2–3′UTR-delta2D vectors were linearized with Not1 or XhoI and used as templates for *in vitro* transcription using Sp6 polymerase (Roche, Indianapolis, IN, USA) in the presence of biotin labeling mix containing biotinylated UTPs (Roche) according to the manufacturer's protocol. 7 nM of probes were then incubated with approximately 500 μg of cell lysate of HCT116 cells transiently transfected with pCMVtag2bhWig-1 and used for the pulldown as described in [7].

### Preparation of RNA libraries and sequencing

RNA libraries for sequencing were prepared using TruSeq RNA Sample Prep (Illumina, CA, USA) (non-strand-specific) according to the manufacturer's instructions with the following changes. The protocols were automated using an MBS 1200 pipetting station (Nordiag AB, Sweden) and all purification steps and gel-cuts were replaced by the magnetic bead clean-up methods as previously described [58]. The clustering was performed on a cBot cluster generation system using a HiSeq paired-end read cluster generation kit according to the manufacturer's instructions (Illumina). The 30 samples were sequenced on an Illumina HiSeq 2000 as paired-end reads of 100 bp according to the manufacturer's instructions. Base conversion was done using Illumina's OLB v1.9.

### Transcriptomics analysis

We used TopHat [59] (v. 1.3.3, default settings) for alignment and Cufflinks [60] (v. 1.2.1, default settings) to quantitate transcript expression levels into FPKM (Fragments Per Kilobase of exon per Million fragments mapped) values. HTSeq (v. 0.5.1, default settings) (http://www.huber.embl.de/users/anders/HTSeq) was used to calculate gene-level read counts. These read counts were used as input to limma (Linear Models for Microarray Data) [61] to perform enrichment analysis. The raw counts were normalized using the voom function in limma which variance stabilizes the counts and adjusts them to sequencing library size. In the limma analysis, we defined the enriched RNAs in Wig-1 RIPs versus empty and mutant controls as the RNAs showing at least 2-fold enrichment after Wig-1 pulldown (log2 fold change (log_2_FC) > 1) and an adjusted *p*-value less than 0.05 for the difference (IP.W-IP.C)-(input.W-input.C) or (IP.W-IP.C)-(input.W-input.C) and (IP.W-IP.Wmut)-(input.W-input.Wmut) for HCT116 and Saos-2 cell line experiments respectively. C: control (empty vector); W: Flag-Wig-1; Wmut: Flag-Wig-1-point mutant; IP: immunoprecipitated.

### Gene ontology and network enrichment analysis

We used DAVID (Database for Annotation, Visualization and Integrated Discovery) to perform gene ontology (GO) enrichment analysis [23]. Wig-1-associated RNAs were categorized into cellular compartment, molecular function, and biological process terms and significantly enriched GO annotation were calculated by Fisher's exact test.

For the network enrichment analysis (NEA), we probabilistically estimated putative functional relations between gene sets as described in [62]. The advantage of this approach is that it considers the whole list of enriched genes, including those that are not members of any already known functional category but can be connected to such category in a network. Connectivity in a global interaction network between Wig-1 bound mRNAs and genes of known pathways were quantified as total numbers of links (edges) found in the global interaction network.

The global interaction network was based on the one from FunCoup [63], a database that provides interactomes computationally inferred from published articles and high-throughput data for a variety of species by combining different types of data, such as protein-protein interactions, mRNA co-expression, sub-cellular co-localization, phylogenetic profile similarity, co-targeting by either miRNA or transcription factors, protein co-expression, and domain-domain interactions. It also considered all known links derived from KEGG [64], PhosphoSite [65], CORUM [66], MSigDB [67], and HTRIdb [68] databases. It further transfers data from other eukaryotic species via orthologs. The resource uses Bayesian statistics to probabilistically estimate functional coupling between genes and proteins. We included in our analysis all links with the FunCoup confidence cutoff of 0.5 or higher. The whole network included 19031 unique genes with 974427 links between them. In a recent benchmark [69], we demonstrated that this is the most sensitive and specific network for NEA in cancer-related studies.

### 3′UTR sequence analysis

The occurrences of the ARE motifs in the 3′UTR sequences of the Wig-1 associated mRNAs were determined using RSAT [26], a regulatory sequence analysis suite that integrates a wide collection of modular tools for the detection of cis-regulatory elements in genome sequences. The 3′UTR sequences of the bound mRNAs were also used to scan for conserved motifs using DREME [27]. DREME performs discriminative discovery of short motifs (up to 8 bases) and uses a Fisher Exact Test to determine significance of each motif found in the positive set (the 3′UTRs of the 286 bound mRNAs) as compared with its representation in the random group (the nucleotide sequence of each 3′UTR was shuffled to generate a new sequence with the same nucleotide composition and length) and to a negative group of 3′UTRs from 286 unbound mRNAs (unbound mRNAs are defined as mRNAs detected by sequencing but not enriched by Flag-Wig-1 RIP). Since the software used for motif analysis in this study are set for DNA analysis, for simplicity, we will use T instead of U in describing Wig-1 preferential motifs, with exception for the 2D structural analysis figures.

### 2D structure analysis

Prediction of 2D sequence structure was performed using the program RNAfold (default parameter settings) contained in the ViennaRNA Package 2 [70]. Shared sequences and structures in the 3′UTR sequences were predicted performing a multiple alignment using the software LocARNA (local alignment with default parameter settings) [71]. Consensus sequences of shared regions are part of LocARNA output.

### GC content calculation

GC content was calculated using the software geecee (http://emboss.bioinformatics.nl/cgi-bin/emboss/geecee).

### Data access

RIP-Seq data are available in the ArrayExpress database (http://www.ebi.ac.uk/arrayexpress) under accession number E-MTAB-2840.

## SUPPLEMENTARY FIGURES AND TABLES























## References

[1] Varmeh-Ziaie S, Okan I, Wang Y, Magnusson KP, Warthoe P, Strauss M, Wiman KG (1997). Wig-1, a new p53-induced gene encoding a zinc finger protein. Oncogene.

[2] Hellborg F, Wiman KG (2004). The p53-induced Wig-1 zinc finger protein is highly conserved from fish to man. Int J Oncol.

[3] Mendez-Vidal C, Wilhelm MT, Hellborg F, Qian W, Wiman KG (2002). The p53-induced mouse zinc finger protein wig-1 binds double-stranded RNA with high affinity. Nucleic Acids Res.

[4] Mendez Vidal C, Prahl M, Wiman KG (2006). The p53-induced Wig-1 protein binds double-stranded RNAs with structural characteristics of siRNAs and miRNAs. FEBS Lett.

[5] Bersani C, Xu LD, Vilborg A, Lui WO, Wiman KG (2014). Wig-1 regulates cell cycle arrest and cell death through the p53 targets FAS and 14–3-3sigma. Oncogene.

[6] Vilborg A, Bersani C, Wickstrom M, Segerstrom L, Kogner P, Wiman KG (2012). Wig-1, a novel regulator of N-Myc mRNA and N-Myc-driven tumor growth. Cell Death Dis.

[7] Vilborg A, Glahder JA, Wilhelm MT, Bersani C, Corcoran M, Mahmoudi S, Rosenstierne M, Grander D, Farnebo M, Norrild B, Wiman KG (2009). The p53 target Wig-1 regulates p53 mRNA stability through an AU-rich element. Proc Natl Acad Sci U S A.

[8] Kim BC, Lee HC, Lee JJ, Choi CM, Kim DK, Lee JC, Ko YG, Lee JS (2012). Wig1 prevents cellular senescence by regulating p21 mRNA decay through control of RISC recruitment. The EMBO journal.

[9] Kwon SC, Yi H, Eichelbaum K, Fohr S, Fischer B, You KT, Castello A, Krijgsveld J, Hentze MW, Kim VN (2013). The RNA-binding protein repertoire of embryonic stem cells. Nature structural & molecular biology.

[10] Castello A, Fischer B, Eichelbaum K, Horos R, Beckmann BM, Strein C, Davey NE, Humphreys DT, Preiss T, Steinmetz LM, Krijgsveld J, Hentze MW (2012). Insights into RNA biology from an atlas of mammalian mRNA-binding proteins. Cell.

[11] Bakheet T, Williams BR, Khabar KS (2006). ARED 3. 0: the large and diverse AU-rich transcriptome. Nucleic Acids Res.

[12] Chen CY, Shyu AB (1995). AU-rich elements: characterization and importance in mRNA degradation. Trends Biochem Sci.

[13] Taylor GA, Carballo E, Lee DM, Lai WS, Thompson MJ, Patel DD, Schenkman DI, Gilkeson GS, Broxmeyer HE, Haynes BF, Blackshear PJ (1996). A pathogenetic role for TNF alpha in the syndrome of cachexia, arthritis, and autoimmunity resulting from tristetraprolin (TTP) deficiency. Immunity.

[14] Stumpo DJ, Byrd NA, Phillips RS, Ghosh S, Maronpot RR, Castranio T, Meyers EN, Mishina Y, Blackshear PJ (2004). Chorioallantoic fusion defects and embryonic lethality resulting from disruption of Zfp36L1, a gene encoding a CCCH tandem zinc finger protein of the Tristetraprolin family. Molecular and cellular biology.

[15] Katsanou V, Milatos S, Yiakouvaki A, Sgantzis N, Kotsoni A, Alexiou M, Harokopos V, Aidinis V, Hemberger M, Kontoyiannis DL (2009). The RNA-binding protein Elavl1/HuR is essential for placental branching morphogenesis and embryonic development. Molecular and cellular biology.

[16] Chang N, Yi J, Guo G, Liu X, Shang Y, Tong T, Cui Q, Zhan M, Gorospe M, Wang W (2010). HuR uses AUF1 as a cofactor to promote p16INK4 mRNA decay. Molecular and cellular biology.

[17] Lal A, Mazan-Mamczarz K, Kawai T, Yang X, Martindale JL, Gorospe M (2004). Concurrent versus individual binding of HuR and AUF1 to common labile target mRNAs. The EMBO journal.

[18] Linker K, Pautz A, Fechir M, Hubrich T, Greeve J, Kleinert H (2005). Involvement of KSRP in the post-transcriptional regulation of human iNOS expression-complex interplay of KSRP with TTP and HuR. Nucleic acids research.

[19] Pullmann R, Kim HH, Abdelmohsen K, Lal A, Martindale JL, Yang X, Gorospe M (2007). Analysis of turnover and translation regulatory RNA-binding protein expression through binding to cognate mRNAs. Molecular and cellular biology.

[20] Rabani M, Kertesz M, Segal E (2008). Computational prediction of RNA structural motifs involved in posttranscriptional regulatory processes. Proceedings of the National Academy of Sciences of the United States of America.

[21] Barash Y, Calarco JA, Gao W, Pan Q, Wang X, Shai O, Blencowe BJ, Frey BJ (2010). Deciphering the splicing code. Nature.

[22] Wan Y, Kertesz M, Spitale RC, Segal E, Chang HY (2011). Understanding the transcriptome through RNA structure. Nat Rev Genet.

[23] Huang da W, Sherman BT, Tan Q, Kir J, Liu D, Bryant D, Guo Y, Stephens R, Baseler MW, Lane HC, Lempicki RA (2007). DAVID Bioinformatics Resources: expanded annotation database and novel algorithms to better extract biology from large gene lists. Nucleic acids research.

[24] Croft D, O'Kelly G, Wu G, Haw R, Gillespie M, Matthews L, Caudy M, Garapati P, Gopinath G, Jassal B, Jupe S, Kalatskaya I, Mahajan S, May B, Ndegwa N, Schmidt E (2011). Reactome: a database of reactions, pathways and biological processes. Nucleic acids research.

[25] Matoulkova E, Michalova E, Vojtesek B, Hrstka R (2012). The role of the 3′ untranslated region in post-transcriptional regulation of protein expression in mammalian cells. RNA Biol.

[26] Thomas-Chollier M, Sand O, Turatsinze JV, Janky R, Defrance M, Vervisch E, Brohee S, van Helden J (2008). RSAT: regulatory sequence analysis tools. Nucleic acids research.

[27] Bailey TL (2011). DREME: motif discovery in transcription factor ChIP-seq data. Bioinformatics.

[28] Pesole G, Liuni S, Grillo G, Saccone C (1997). Structural and compositional features of untranslated regions of eukaryotic mRNAs. Gene.

[29] Lebedeva S, Jens M, Theil K, Schwanhausser B, Selbach M, Landthaler M, Rajewsky N (2011). Transcriptome-wide analysis of regulatory interactions of the RNA-binding protein HuR. Molecular cell.

[30] Wu X, Chesoni S, Rondeau G, Tempesta C, Patel R, Charles S, Daginawala N, Zucconi BE, Kishor A, Xu G, Shi Y, Li ML, Irizarry-Barreto P (2013). Combinatorial mRNA binding by AUF1 and Argonaute 2 controls decay of selected target mRNAs. Nucleic acids research.

[31] Stoecklin G, Tenenbaum SA, Mayo T, Chittur SV, George AD, Baroni TE, Blackshear PJ, Anderson P (2008). Genome-wide analysis identifies interleukin-10 mRNA as target of tristetraprolin. J Biol Chem.

[32] Madar S, Harel E, Goldstein I, Stein Y, Kogan-Sakin I, Kamer I, Solomon H, Dekel E, Tal P, Goldfinger N, Friedlander G, Rotter V (2013). Mutant p53 attenuates the anti-tumorigenic activity of fibroblasts-secreted interferon beta. PloS one.

[33] Hinman MN, Lou H (2008). Diverse molecular functions of Hu proteins. Cellular and molecular life sciences.

[34] Huang LE, Arany Z, Livingston DM, Bunn HF (1996). Activation of hypoxia-inducible transcription factor depends primarily upon redox-sensitive stabilization of its alpha subunit. J Biol Chem.

[35] Iwai K, Yamanaka K, Kamura T, Minato N, Conaway RC, Conaway JW, Klausner RD, Pause A (1999). Identification of the von Hippel-lindau tumor-suppressor protein as part of an active E3 ubiquitin ligase complex. Proc Natl Acad Sci U S A.

[36] Jain M, Nilsson R, Sharma S, Madhusudhan N, Kitami T, Souza AL, Kafri R, Kirschner MW, Clish CB, Mootha VK (2012). Metabolite profiling identifies a key role for glycine in rapid cancer cell proliferation. Science.

[37] Nilsson R, Jain M, Madhusudhan N, Sheppard NG, Strittmatter L, Kampf C, Huang J, Asplund A, Mootha VK (2014). Metabolic enzyme expression highlights a key role for MTHFD2 and the mitochondrial folate pathway in cancer. Nat Commun.

[38] Mercier I, Jasmin JF, Pavlides S, Minetti C, Flomenberg N, Pestell RG, Frank PG, Sotgia F, Lisanti MP (2009). Clinical and translational implications of the caveolin gene family: lessons from mouse models and human genetic disorders. Lab Invest.

[39] Goetz JG, Lajoie P, Wiseman SM, Nabi IR (2008). Caveolin-1 in tumor progression: the good, the bad and the ugly. Cancer Metastasis Rev.

[40] Zou H, Volonte D, Galbiati F (2012). Interaction of caveolin-1 with Ku70 inhibits Bax-mediated apoptosis. PloS one.

[41] Nathan CO, Amirghahari N, Abreo F, Rong X, Caldito G, Jones ML, Zhou H, Smith M, Kimberly D, Glass J (2004). Overexpressed eIF4E is functionally active in surgical margins of head and neck cancer patients via activation of the Akt/mammalian target of rapamycin pathway. Clin Cancer Res.

[42] Nasr Z, Robert F, Porco JA, Muller WJ, Pelletier J (2013). eIF4F suppression in breast cancer affects maintenance and progression. Oncogene.

[43] Liang S, Guo R, Zhang Z, Liu D, Xu H, Xu Z, Wang X, Yang L (2013). Upregulation of the eIF4E signaling pathway contributes to the progression of gastric cancer, and targeting eIF4E by perifosine inhibits cell growth. Oncol Rep.

[44] Kao CL, Hsu HS, Chen HW, Cheng TH (2009). Rapamycin increases the p53/MDM2 protein ratio and p53-dependent apoptosis by translational inhibition of mdm2 in cancer cells. Cancer letters.

[45] Topisirovic I, Siddiqui N, Orolicki S, Skrabanek LA, Tremblay M, Hoang T, Borden KL (2009). Stability of eukaryotic translation initiation factor 4E mRNA is regulated by HuR, and this activity is dysregulated in cancer. Mol Cell Biol.

[46] Yang J, O'Donnell L, Durocher D, Brown GW (2012). RMI1 promotes DNA replication fork progression and recovery from replication fork stress. Molecular and cellular biology.

[47] Hirschhaeuser F, Sattler UG, Mueller-Klieser W (2011). Lactate: a metabolic key player in cancer. Cancer research.

[48] Yasuda M, Hatanaka T, Shirato H, Nishioka T (2014). Cell type-specific reciprocal regulation of HIF1A gene expression is dependent on 5′- and 3′-UTRs. Biochemical and biophysical research communications.

[49] Chamboredon S, Ciais D, Desroches-Castan A, Savi P, Bono F, Feige JJ, Cherradi N (2011). Hypoxia-inducible factor-1alpha mRNA: a new target for destabilization by tristetraprolin in endothelial cells. Mol Biol Cell.

[50] Tomasevic G, Shamloo M, Israeli D, Wieloch T (1999). Activation of p53 and its target genes p21(WAF1/Cip1) and PAG608/Wig-1 in ischemic preconditioning. Brain Res Mol Brain Res.

[51] Helton R, Cui J, Scheel JR, Ellison JA, Ames C, Gibson C, Blouw B, Ouyang L, Dragatsis I, Zeitlin S, Johnson RS, Lipton SA, Barlow C (2005). Brain-specific knock-out of hypoxia-inducible factor-1alpha reduces rather than increases hypoxic-ischemic damage. J Neurosci.

[52] Brahimi-Horn MC, Pouyssegur J (2007). Hypoxia in cancer cell metabolism and pH regulation. Essays Biochem.

[53] Lu JY, Bergman N, Sadri N, Schneider RJ (2006). Assembly of AUF1 with eIF4G-poly(A) binding protein complex suggests a translation function in AU-rich mRNA decay. RNA.

[54] Gouble A, Grazide S, Meggetto F, Mercier P, Delsol G, Morello D (2002). A new player in oncogenesis: AUF1/hnRNPD overexpression leads to tumorigenesis in transgenic mice. Cancer research.

[55] Wang W, Caldwell MC, Lin S, Furneaux H, Gorospe M (2000). HuR regulates cyclin A and cyclin B1 mRNA stability during cell proliferation. The EMBO journal.

[56] Kim HH, Kuwano Y, Srikantan S, Lee EK, Martindale JL, Gorospe M (2009). HuR recruits let-7/RISC to repress c-Myc expression. Genes & development.

[57] Vilborg A, Bersani C, Wilhelm MT, Wiman KG (2011). The p53 target Wig-1: a regulator of mRNA stability and stem cell fate?. Cell death and differentiation.

[58] Borgstrom E, Lundin S, Lundeberg J (2011). Large scale library generation for high throughput sequencing. PloS one.

[59] Trapnell C, Pachter L, Salzberg SL (2009). TopHat: discovering splice junctions with RNA-Seq. Bioinformatics.

[60] Trapnell C, Roberts A, Goff L, Pertea G, Kim D, Kelley DR, Pimentel H, Salzberg SL, Rinn JL, Pachter L (2012). Differential gene and transcript expression analysis of RNA-seq experiments with TopHat and Cufflinks. Nat Protoc.

[61] Law CW, Chen Y, Shi W, Smyth GK (2014). Voom: precision weights unlock linear model analysis tools for RNA-seq read counts. Genome Biol.

[62] Alexeyenko A, Lee W, Pernemalm M, Guegan J, Dessen P, Lazar V, Lehtio J, Pawitan Y (2012). Network enrichment analysis: extension of gene-set enrichment analysis to gene networks. BMC bioinformatics.

[63] Alexeyenko A, Sonnhammer EL (2009). Global networks of functional coupling in eukaryotes from comprehensive data integration. Genome research.

[64] Kanehisa M (2002). The KEGG database. Novartis Found Symp.

[65] Hornbeck PV, Chabra I, Kornhauser JM, Skrzypek E, Zhang B (2004). PhosphoSite: A bioinformatics resource dedicated to physiological protein phosphorylation. Proteomics.

[66] Ruepp A, Brauner B, Dunger-Kaltenbach I, Frishman G, Montrone C, Stransky M, Waegele B, Schmidt T, Doudieu ON, Stumpflen V, Mewes HW (2008). CORUM: the comprehensive resource of mammalian protein complexes. Nucleic acids research.

[67] Subramanian A, Tamayo P, Mootha VK, Mukherjee S, Ebert BL, Gillette MA, Paulovich A, Pomeroy SL, Golub TR, Lander ES, Mesirov JP (2005). Gene set enrichment analysis: a knowledge-based approach for interpreting genome-wide expression profiles. Proceedings of the National Academy of Sciences of the United States of America.

[68] Bovolenta LA, Acencio ML, Lemke N (2012). HTRIdb: an open-access database for experimentally verified human transcriptional regulation interactions. BMC genomics.

[69] Merid SK, Goranskaya D, Alexeyenko A (2014). Distinguishing between driver and passenger mutations in individual cancer genomes by network enrichment analysis. BMC bioinformatics.

[70] Lorenz R, Bernhart SH, Honer Zu Siederdissen C, Tafer H, Flamm C, Stadler PF, Hofacker IL (2011). ViennaRNA Package 2. 0. Algorithms Mol Biol.

[71] Will S, Joshi T, Hofacker IL, Stadler PF, Backofen R (2012). LocARNA-P: accurate boundary prediction and improved detection of structural RNAs. RNA.

