# Supplementary material for: Genome-wide identification of Wig-1 mRNA targets by RIP-Seq analysis
Source: Oncotarget. 2015 Dec 11;7(2):1895–911. doi: 10.18632/oncotarget.6557 (PMC4811505; doi:10.18632/oncotarget.6557)
Supplement: Supplementary file 1 [file oncotarget-07-1895-s001.pdf]

SUPPLEMENTARY FIGURES AND TABLES

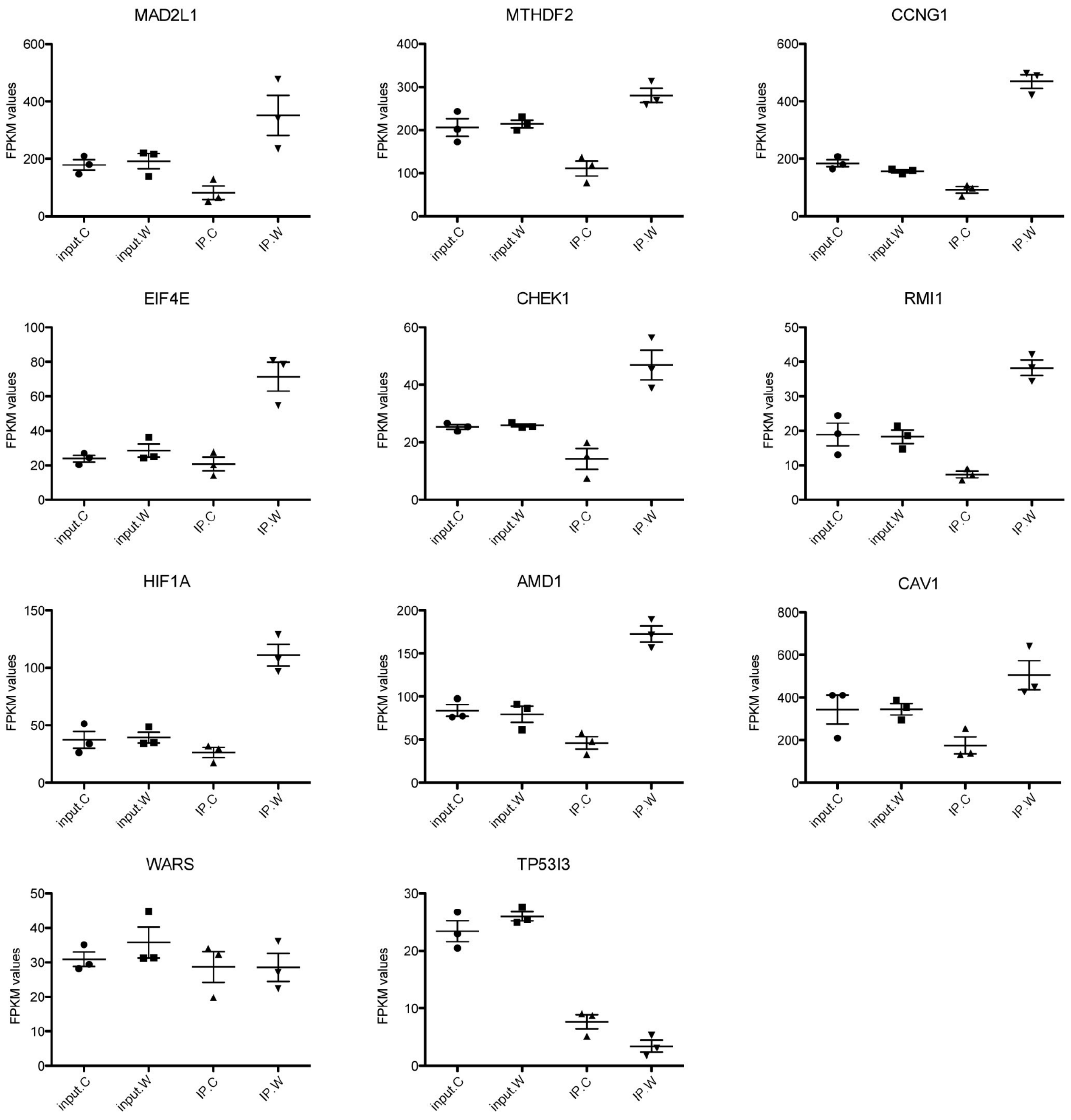

Supplementary Figure S1: FPKM values calculated in the triplicates RIP-Seq experiments per sample for the selected targets in HCT116 cells.

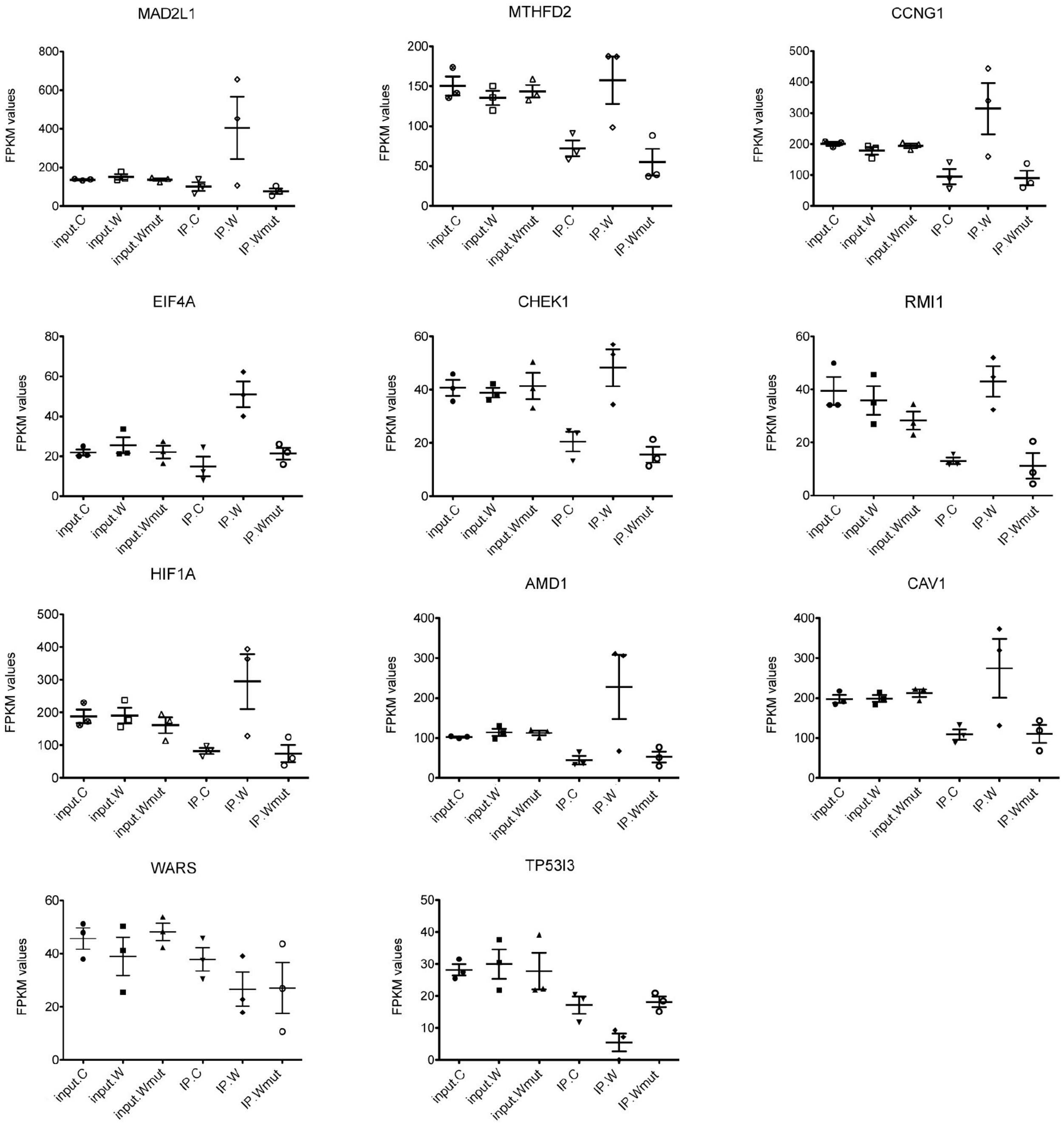

Supplementary Figure S2: FPKM values calculated in the triplicates RIP-Seq experiments per sample for the selected targets in Saos-2 cells.

a. CCNG1

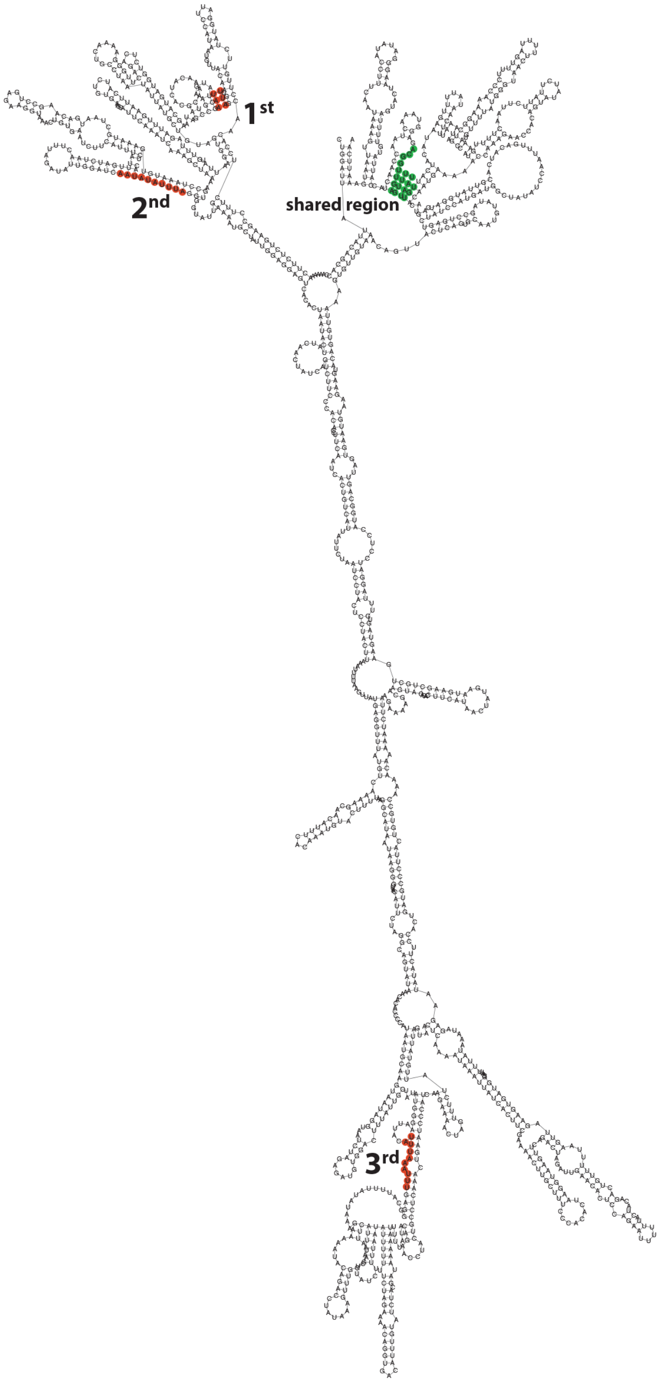

Supplementary Figure S3: 2D sequence structures predicted by RNAfold for the 9 bound 3′UTR sequences. ARE elements are colored in red, consensus 2D motifs are colored in green (indicated as "shared motif"). A. CCNG1. (*Continued*).

b. RMI1

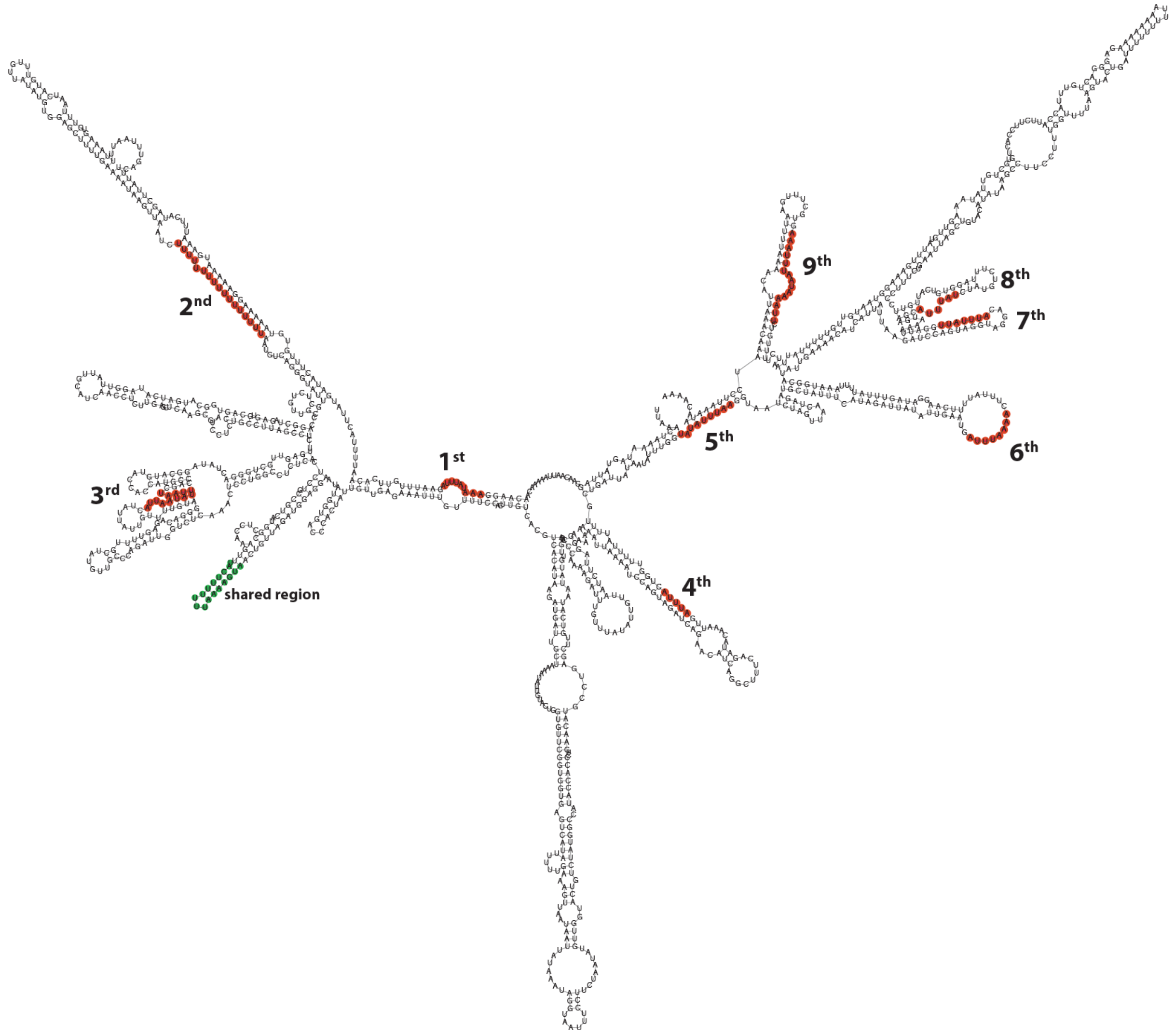

Supplementary Figure S3: (*Continued*) 2D sequence structures predicted by RNAfold for the 9 bound 3′UTR sequences. ARE elements are colored in red, consensus 2D motifs are colored in green (indicated as "shared motif"). B. RMI1. (*Continued*)

c. CHEK1

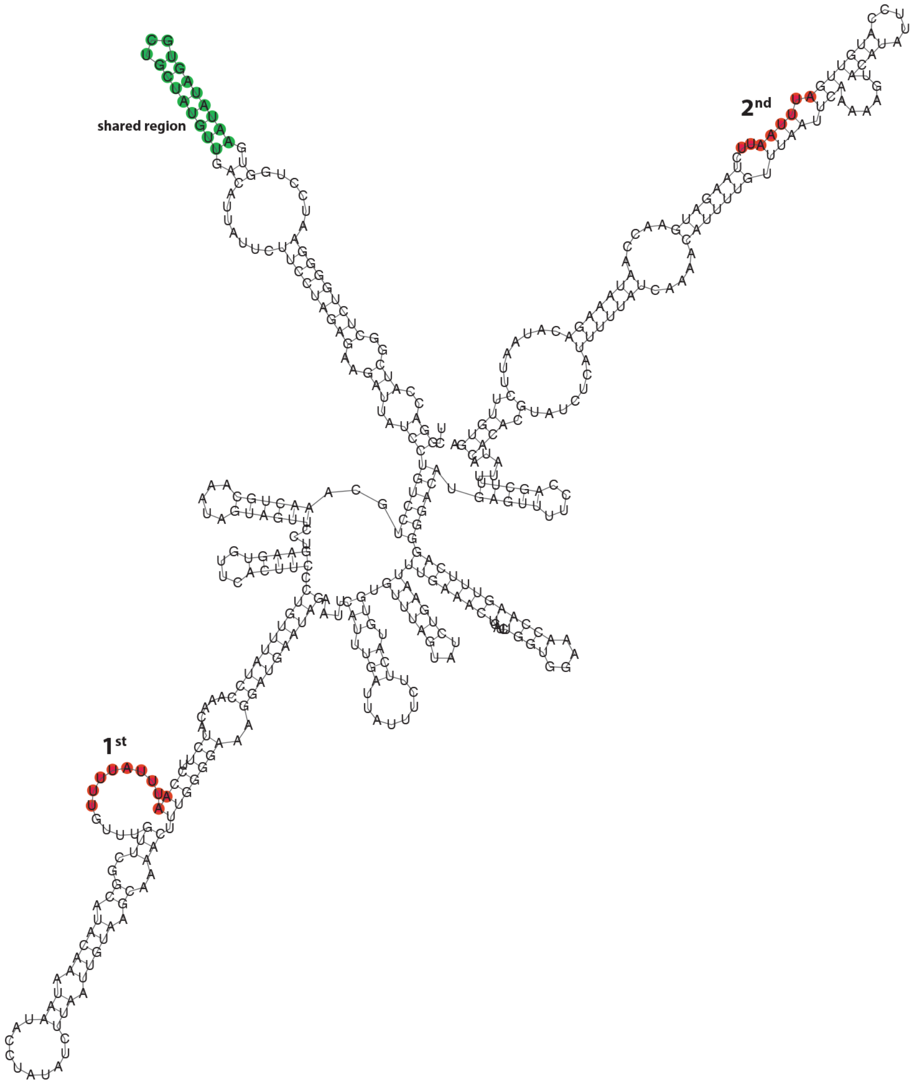

Supplementary Figure S3: (*Continued*) 2D sequence structures predicted by RNAfold for the 9 bound 3′UTR sequences. ARE elements are colored in red, consensus 2D motifs are colored in green (indicated as "shared motif"). **C.** CHEK1. (*Continued*)

d. MTHFD2

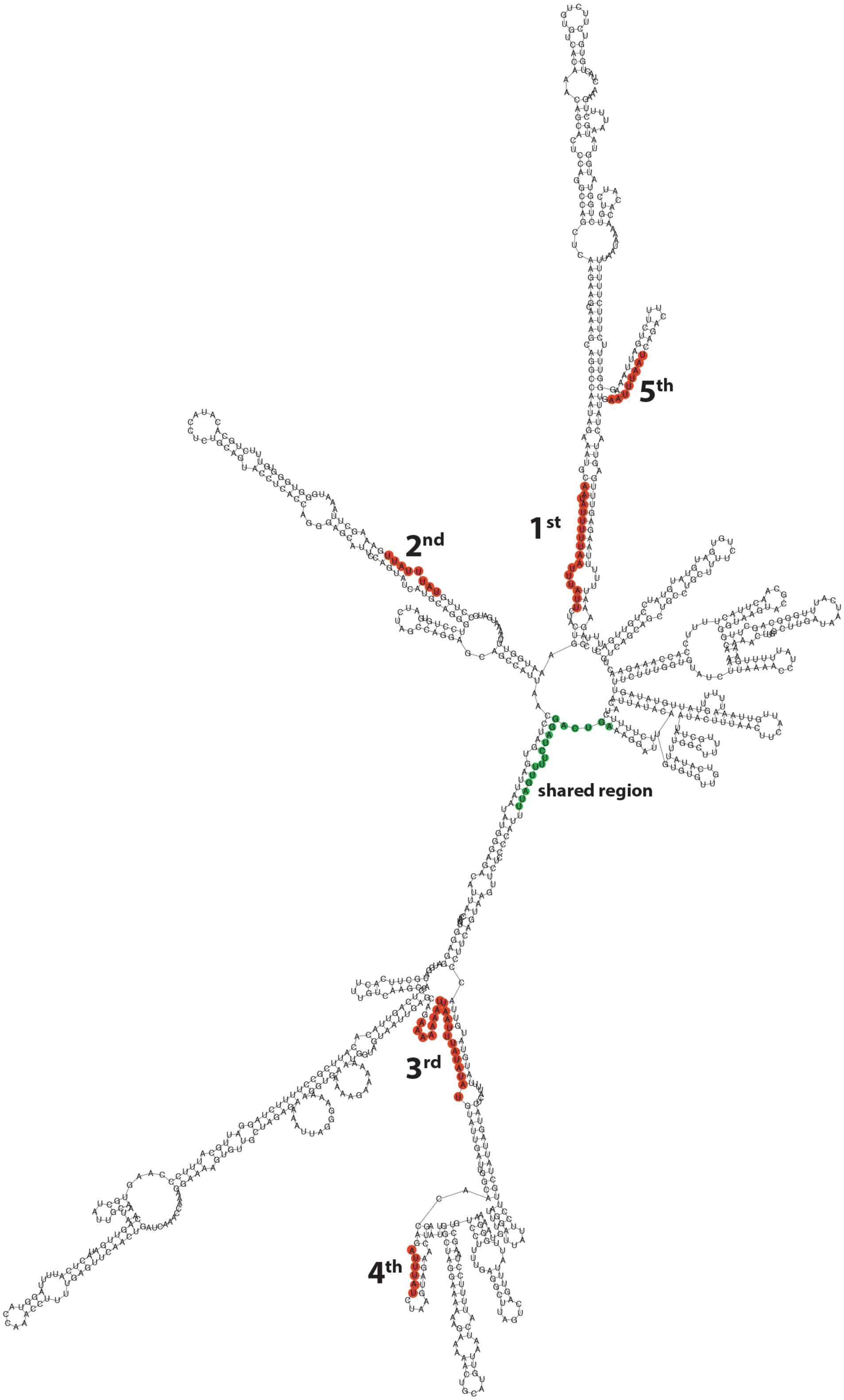

Supplementary Figure S3: (*Continued*) 2D sequence structures predicted by RNAfold for the 9 bound 3′UTR sequences. ARE elements are colored in red, consensus 2D motifs are colored in green (indicated as "shared motif"). D. MTHFD2 (*Continued*)

e. CAV1

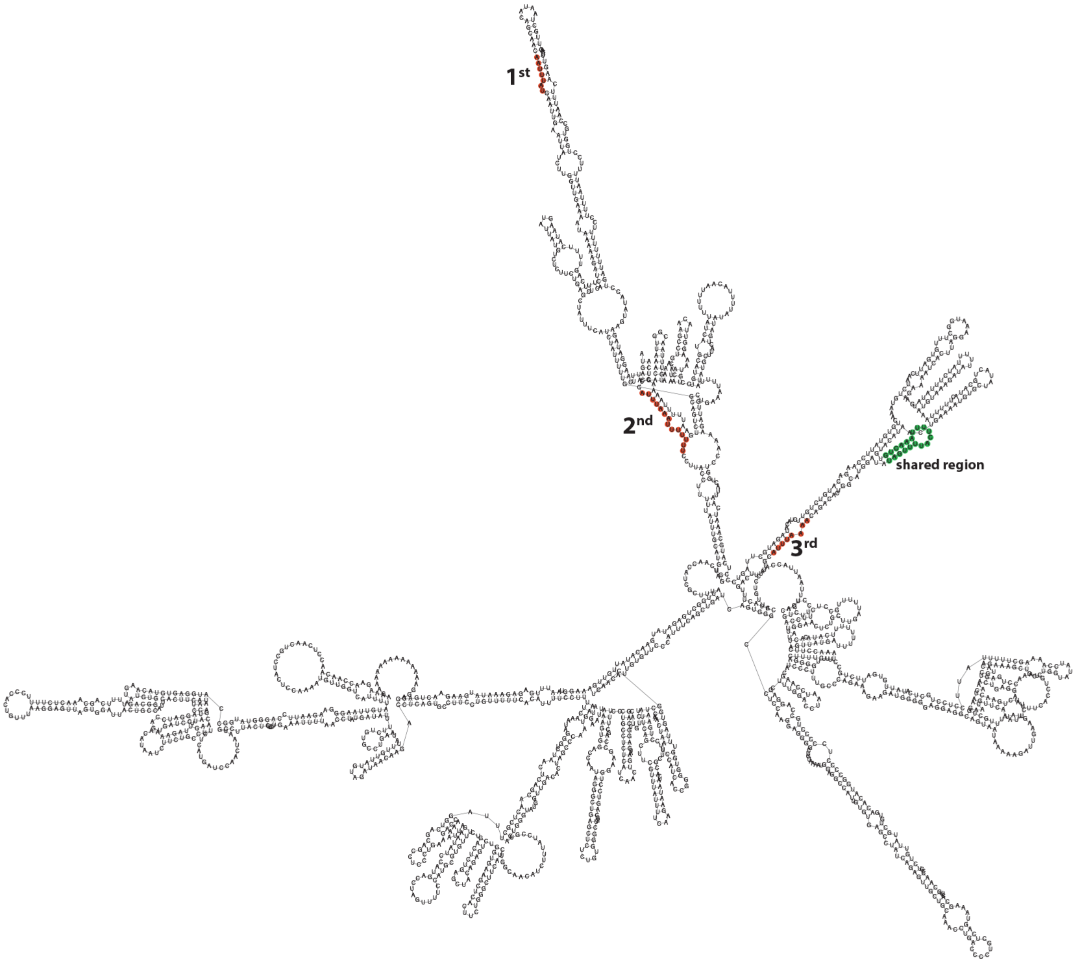

Supplementary Figure S3: (*Continued*) 2D sequence structures predicted by RNAfold for the 9 bound 3′UTR sequences. ARE elements are colored in red, consensus 2D motifs are colored in green (indicated as “shared motif”).
E. CAV1. (*Continued*)

f. AMD1

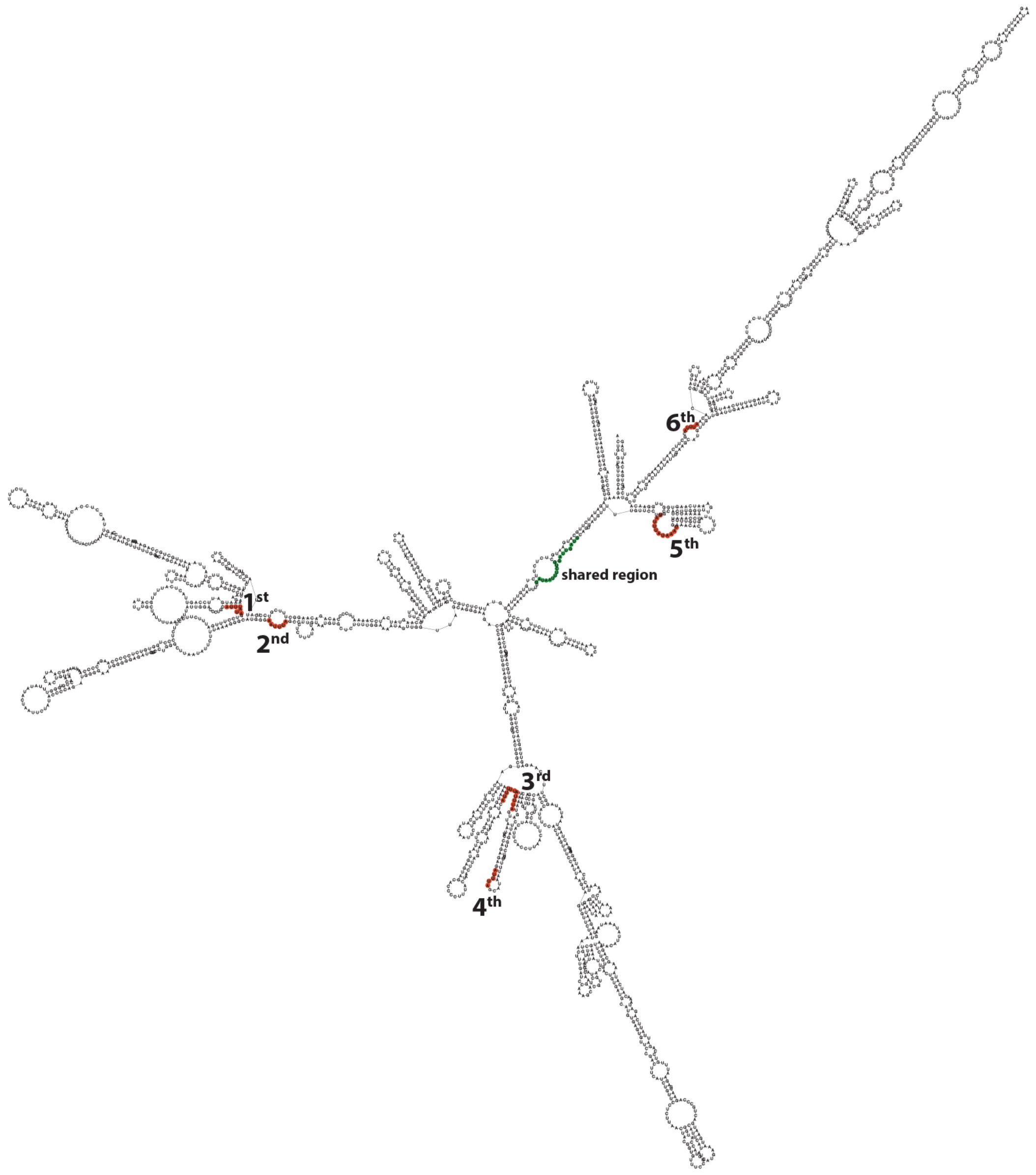

Supplementary Figure S3: (*Continued*) 2D sequence structures predicted by RNAfold for the 9 bound 3′UTR sequences. ARE elements are colored in red, consensus 2D motifs are colored in green (indicated as "shared motif"). **F.** AMD1. (*Continued*)

g. HIF1A

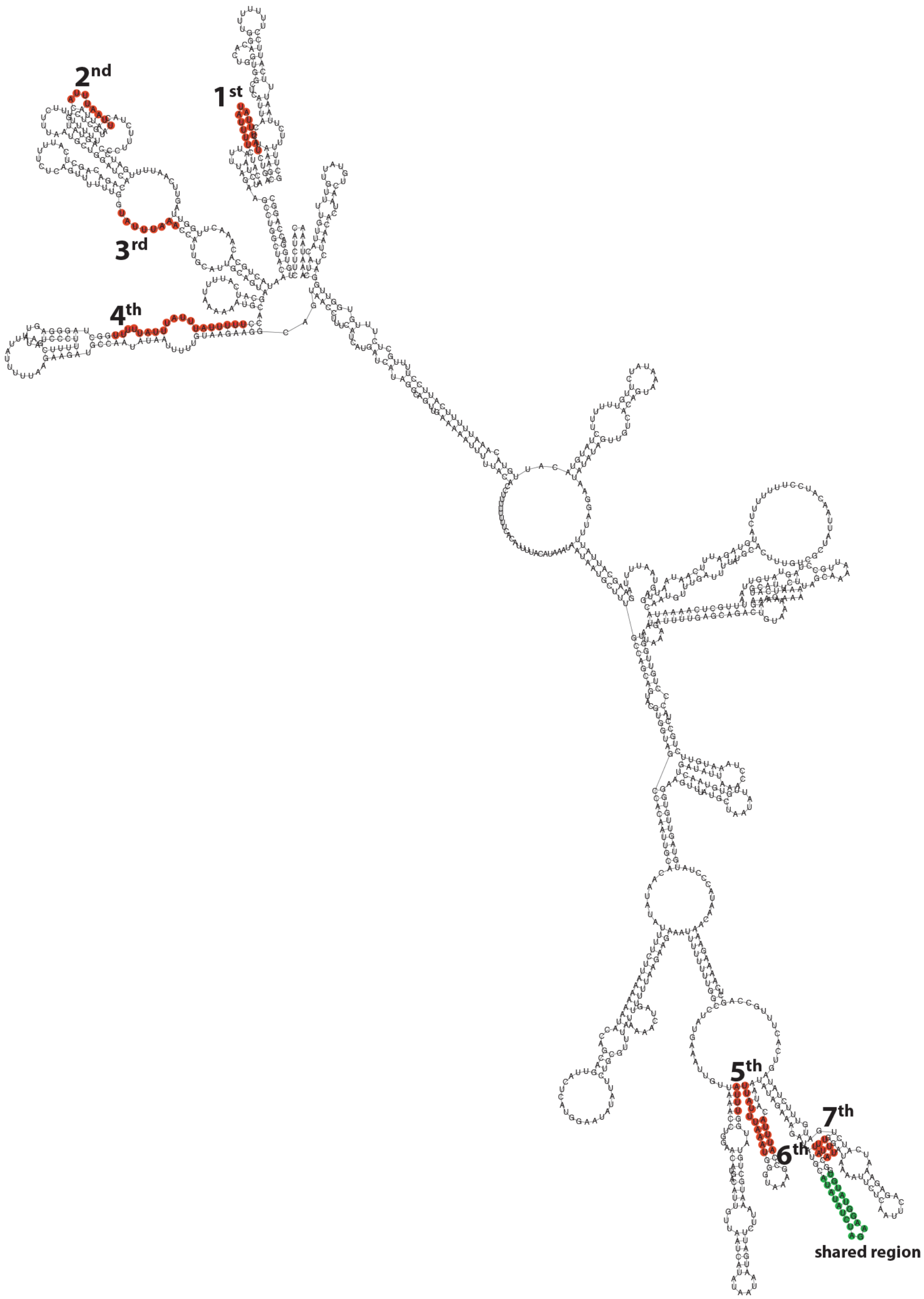

Supplementary Figure S3: (*Continued*) 2D sequence structures predicted by RNAfold for the 9 bound 3′UTR sequences. ARE elements are colored in red, consensus 2D motifs are colored in green (indicated as "shared motif"). G. HIF1A. (*Continued*)

h. MAD2L1

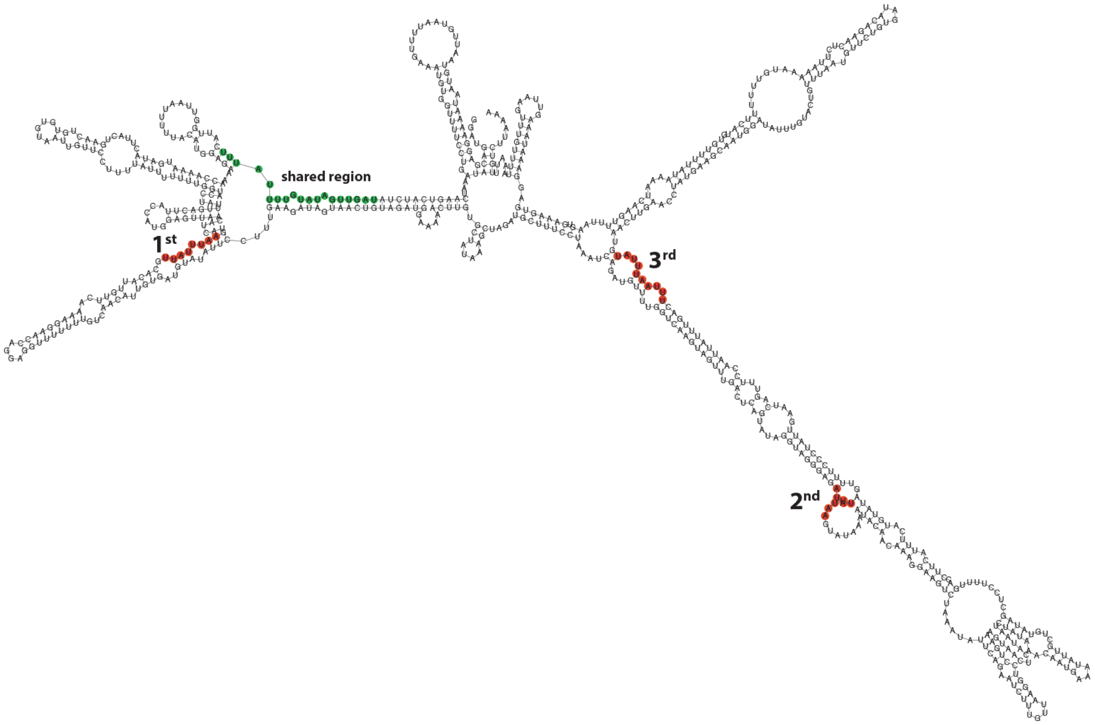

Supplementary Figure S3: (*Continued*) 2D sequence structures predicted by RNAfold for the 9 bound 3′UTR sequences. ARE elements are colored in red, consensus 2D motifs are colored in green (indicated as "shared motif"). H. MAD2L1. (*Continued*)

i. EIF4E

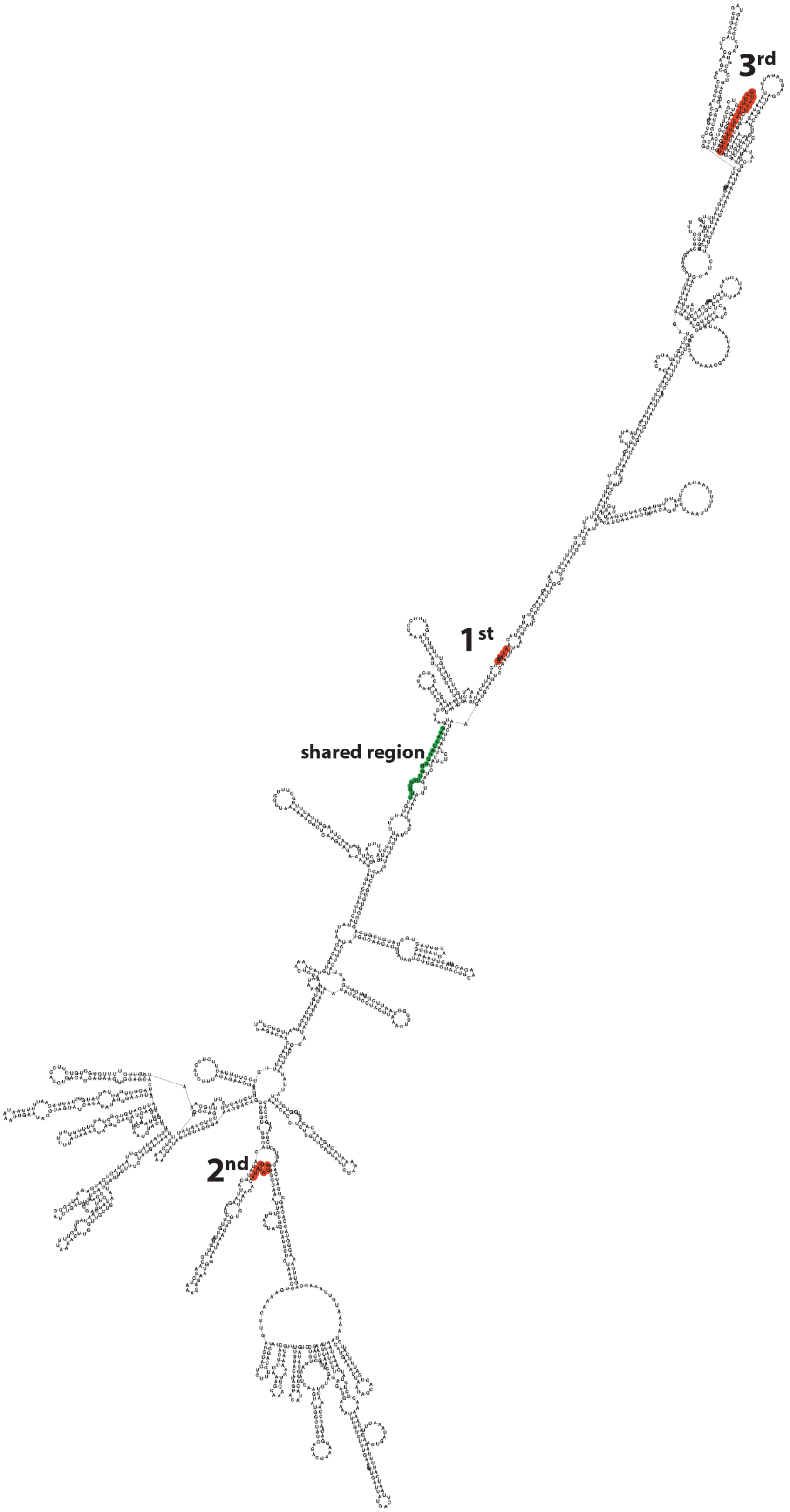

Supplementary Figure S3: (*Continued*) 2D sequence structures predicted by RNAfold for the 9 bound 3′UTR sequences. ARE elements are colored in red, consensus 2D motifs are colored in green (indicated as "shared motif").
I. EIF4E. (*Continued*)

>CCNG1
CTGGATTATTACAGCACCAAAAAACTTCTCTGAAGCCTTTCTCCACAACCTTGTTCTATGGATTCCATAATGTTACAATGGATTTA
AGCTATGAAGCCTCAAAACATCACGAGATAAGCATGATGGTCTCAGACTTGGGAAAACTGCCTAATATTATGCTGTAGTGGAATTA
TGTTTAGATTTGAATTCATCTGTGAAGCATTCAAATCAAAGCTAAAAGCCTAAATGTGAAATGCTAATGACAAGCCTGAGAAGGT
AAACTGTGAATCTTCATTTCTATCATTGATCTAACTTTAGATATTGGATCAATATATTTAGGTGGTATTGAAAATGCTATTGGAGG
AGTCACACTAATACTATCAACTATCAGTCTTCCCACAGCTTCAATCACTGTCATTATTCTAATCCTACTCCTACTTAAATTTTAAGT
TATGAGGTTTATGTCAAAAGCAACATTTCACAAATGTACTTTTAAGGCATAATAAGGGTTAACATTCTAGGCAGTATAAACACACC
CCATAATGCAAGTAATAGGTAATCTAGAGATGTGGACTTTATTGCTATATGGGAATTACATTTAAATTTGAGGGCATTTTATATA
AAGAAAAATACAGACCTATAAAGTTTGGCATATTCATTAAGTTATCTTTTAATATTTTTTTCTAGAAAACAGGTGACATTTGTATC
TACGATAAAAATTTTTATACAGAACCTACTGCCTCAAACTGAATCCCATCAAGAAAACTAGTTTCTATTGTATTAGTAACTCAAAA
TAAATTATCACTTCGAAAACTTGCTTTCCCACACTAAGGTAAGTTCAGACTAGATTGAACACTCCAGAATTTTTTACTACAGACTG
TTTTTAAGTTAGAAGTGATGGCAATTTTATAAATAGAGAATATACTTCCACTGATGCCCTTACTGTGCCAAAACAAAAATCTTAAG
AAAAGCAAGTAGACACCTTCATAACTATGAATGAAGCTGCTGAAGTAGTGTTTAGGATCCTCCATGGCAGTTAGTGAATGTAAGA
AGTACAGTGTTAAAGTGTTGTAAACAGTTACTCAGTGCAATGTATAGCCTGAGTCTATCCATGATGGCTATATCCAATTTGACATC
ACGTTATGGATCAGTACACAATGAAAAACCAAAGAACCACAGTATATCTTATTCTTAACTTTTGTAAACCATGTTTTATGGGTAAC
TTTTTAGTTTTCCCAAAAGGCTGATAAATTTCAATATTTTGAATACATCATTGTTAATTTTGAGTTGGCAGAGGTAAACTAACCAA
CTACCATTATGTTTTAGTACTAAGGGATATACCTTTCAATAAAGTTAATGAAATTCAA

>RMI1
TTAAACTAAAATAGTATTAGGAACAATTAAAAACAACAAGGAAATATTTAGAATTTGTTCACAATTTTACTTATGATACTTTGTG
TAAAAAGGAAAAATGAAATTTCATAGCTTATTTCAGTTTAATTTTAAAGTGTTTAATCATGTTTGTTATATGTGGAGCTTTTGAA
AATAAGTTAATCTTTTTTTTTTTTTTTTTAATGTCAGGGTATTGCTCTGTTGCCCAGGCTAGAGTGCAGTGGCATGATCATAGGTT
ATTGCATCAACCTCTTGAGCTCAAGCGATCCTCCTGCCTTAGCCTCTTGAGTTGCTGGGACTATAGGCATGTACCACCATGCCCAGC
TAATTTATAATTACTATTATTGTTTTGTAGGGACAGAGTTTTGCTATGTTGCCCAGATTGGTCTCAAACTCCTGGCCTCTCACTCCT
GCCGTCATGGCCTCCAAAGTTTACTTTTTTTTAAAAGTAACTGTTAGATGGAGGAATATGGTGACCCACTATTGTTGAGAAATTTGT
TTTCCACTTGTCACGTCACATAAGATGATTGCTAAAATATTGTACTGGTGTTCGGTGGTGAGTCATATTTTTGAAAGTTAATAATT
ATAAATAGGTAATTTCCTTCTAATATGTTGGTACTGTCTATGGCCATACCACCCTGAACATGCCTGAGCTTGTCATAATATGTTGA
GTACCCAAAAGATTTGTTTATATTGTTAATCTTAGGGAAAAAAAATTAAAATCCAGTAGATCAGAACATCAGGCTTTCAGATACA
AATTGATTTACTGGTTTTTATTTTGCTGATTATAATATTTGGTATATTTAAGGTAATCTAGTTAACTAGATGCTATTTCATAGATT
ATATTGAATGATTTAAAACTTTATTTTCAAGGATAGTTTATTTTAAATGGCATATTGAAAACATCATTATTAAGATCCAGTAGGT
AGGACATTTATTGGATTAAAATGAAGCATTTATCTATGTCTTTAGGTGTCATTGTTCCCTTTCTGAATTAGCTGTACATATAAGCC
TTCCTTTGGTTTTAAGTACTGATTTTTTTTTAAAAAAAAGAGGGACTGTTTACCATTCTTCCACTGTGCTGTTATAAAGTTGTATT
TGAAAGGTAATGTTGTTTTTATTAATCTTTTGTCTTAAAATAATTTAAAGTGCTTTGAATTTTAAAACATTAAACAAATCCTTAA
ATAACAAAA

>CHEK1
TCGGACCATCGGCTCTGGGGAATCCTGGTGAATATAGTGCTGCTATGTTGACATTATTCTTCCTAGAGAAGATTATCCTGTCCTGC
AAACTGCAAATAGTAGTTCCTGAAGTGTTCACTTCCCTGTTTATCCAAACATCTTCCAATTTATTTTGTTTGTTCGGCATACAAAT
AATACCTATATCTTAATTGTAAGCAAAACTTTGGGGAAAGGATGAATAGAATTCATTTGATTATTTCTTCATGTGTGTTTAGTATC
TGAATTTGAAACTCATCTGGTGGAAACCAAGTTTCAGGGGACATGAGTTTTCCAGCTTTTATACACACGTATCTCATTTTTATCAA
AACATTTTGTTTAATTCAAAAAGTACATATTCCATGTTGATTTAATTCTAAGATGAACCAATAAAGACATAATTCTTGTGA

>MTHFD2
CTACTGTGTCTTCTGTGTCACAAACAGCACTCCAGGCCAGCTCAAGAAGCAAAGCAGGCCAATAGAAATGCAATATTTTTAATTTA
TTCTACTGAAATGGTTTAAAATGATGCCTTGTATTTATTGAAAGCTTAAATGGGTGGGTGTTTCTGCACATACCTCTGCAGTACCT
CACCAGGGAGCATTCCAGTATCATGCAGGGTCCTGTGATCTAGCCAGGAGCAGCCATTAACCTAGTGATTAATATGGGAGACATTA
CCATATGGAGGATGGATGCTTCACTTTGTCAAGCACCTCAGTTACACATTCGCCTTTTCTAGGATTGCATTTCCCAAGTGCTATTGC
AATAACAGTTGATACTCATTTTAGGTACCAAACCTTTTGAGTTCAACTGATCAAACCAAAGGAAAAGTGTTGCTAGAGAAAATTA
GGGAAAAGGTGAAAAAGAAAAAATGGTAGTAATTGAGCAGAAAAAAATTAATTTATATATGTATTGATTGGCAACCAGATTTATC
TAAGTAGAACTGAATTGGCTAGGAAAAAAGAAAAACTGCATGTTAATCATTTTCCTAAGCTGTCCTTTTGAGGCTTAGTCAGTTTA
TTGGGAAAATGTTTAGGATTATTCCTTGCTATTAGTACTCATTTTATGTATGTTACCCTTCAGTAAGTTCTCCCCATTTTAGTTTT
CTAGGACTGAAAGGATTCTTTTCTACATTATACATGTGTGTTGTCATATTTGGCTTTTGCTATATACTTTAACTTCATTGTTAAAT
TTTTGTATTGTATAGTTTCTTTGGTGTATCTTAAAACCTATTTTTGAAAAACAAACTTGGCTTGATAATCATTTGGGCAGCTTGGG
TAAGTACGCAACTTACTTTTCCACCAAAGAACTGTCAGCAGCTGCCTGCTTTTCTGTGATGTATGTATCCTGTTGACTTTTCCAGAA
ATTTTTTAAGAGTTTGAGTTACTATTGAATTTAATCAGACTTTCTGATTAAAGGGTTTTCTTTCTTTTTTAATAAAACACATCTGT
CTGGTATGGTATGAATTTCTGAAA

>CAV1
ATGACATTTCAAGGATAGAAGTATACCTGATTTTTTTTCCTTTTAATTTTCCTGGTGCCAATTTCAAGTTCCAAGTTGCTAATACA
GCAACAATTTATGAATTGAATTATCTTGGTTGAAAATAAAAAGATCACTTTCTCAGTTTTCATAAGTATTATGTCTCTTCTGAGCT
ATTTCATCTATTTTTGGCAGTCTGAATTTTTAAAACCCATTTAAATTTTTTTCCTTACCTTTTTATTTGCATGTGGATCAACCATC
GCTTTATTGGCTGAGATATGAACATATTGTTGAAAGGTAATTTGAGAGAAATATGAAGAACTGAGGAGGAAAAAAAAAAAAAAGA
AAAGAACCAACAACCTCAACTGCCTACTCCAAAATGTTGGTCATTTTATGTTAAGGGAAGAATTCCAGGGTATGGCCATGGAGTGT
ACAAGTATGTGGGCAGATTTTCAGCAAACTCTTTTCCCACTGTTTAAGGAGTTAGTGGATTACTGCCATTCACTTCATAATCCAGT
AGGATCCAGTGATCCTTACAAGTTAGAAAACATAATCTTCTGCCTTCTCATGATCCAACTAATGCCTTACTCTTCTTGAAATTTTA
ACCTATGATATTTTCTGTGCCTGAATATTTGTTATGTAGATAACAAGACCTCAGTGCCTTCCTGTTTTTCACATTTTCCTTTTCAAA
TAGGGTCTAACTCAGCAACTCGCTTTAGGTCAGCAGCCTCCCTGAAGACCAAAATTAGAATATCCATGACCTAGTTTTCCATGCGT
GTTTCTGACTCTGAGCTACAGAGTCTGGTGAAGCTCACTTCTGGGCTTCATCTGGCAACATCTTTATCCGTAGTGGGTATGGTTGA
CACTAGCCCAATGAAATGAATTAAAGTGGACCAATAGGGCTGAGCTCTCTGTGGGCTGGCAGTCCTGGAAGCCAGCTTTCCCTGCC
TCTCATCAACTGAATGAGGTCAGCATGTCTATTCAGCTTCGTTTATTTTCAAGAATAATCACGCTTTCCTGAATCCAAACTAATCC

Supplementary Figure S4: (*Continued*) Sequences of the nine positively validated 3′UTRs bound by Wig- 1. Putative ARE elements are highlighted in red, consensus 2D motifs are highlighted in green. (*Continued*)

ATCACCGGGGTGGTTTAGTGGCTCAACATTGTGTTCCCATTTCAGCTGATCAGTGGGCCTCCAAGGAGGGGCTGTAAAATGGAGGC
CATTGTGTGAGCCTATCAGAGTTGCTGCAAACCTGACCCCTGCTCAGTAAAGCACTTGCAACCGTCTGTTATGCTGTGACACATGGC
CCCTCCCCCTGCCAGGAGCTTTGGACCTAATCCAAGCATCCCTTTGCCCAGAAAGAAGATGGGGGAGGAGGCAGTAATAAAAAGAT
TGAAGTATTTTGCTGGAATAAGTTCAAATTCTTCTGAACTCAAACTGAGGAATTTCACCTGTAAACCTGAGTCGTACAGAAAGCTG
CCTGGTATATCCAAAAGCTTTTTATTCCTCCTGCTCATATTGTGATTCTGCCTTTGGGGACTTTTCTTAAACCTTCAGTTATGATTT
TTTTTTCATACACTTATTGGAACTCTGCTTGATTTTTGCCTCTTCCAGTCTTCCTGACACTTTAATTACCAACCTGTTACCTACTTT
GACTTTTTGCATTTAAAACAGACACTGGCATGGATATAGTTTTACTTTTAAACTGTGTACATAACTGAAAATGTGCTATACTGCAT
ACTTTTTAAATGTAAAGATATTTTTATCTTTATATGAAGAAAATCACTTAGGAAATGGCTTTGTGATTCAATCTGTAAACTGTGT
ATTCCAAGACATGTCTGTTCTACATAGATGCTTAGTCCCTCATGCAAATCAATTACTGGTCCAAAAGATTGCTGAAATTTTATATG
CTTACTGATATATTTTACAATTTTTTATCATGCATGTCCTGTAAAGGTTACAAGCCTGCACAATAAAAATGTTTAACGGTTAAACA
GTCA

>AMD1
ACTGTGTAGTTGCAGAAAGCCCTAGATGTAATGATAGTGTAATCATTTTGAATTGTATGCATTATTATATCAAGGAGTTAGATAT
CTTGCATGAATGCTCTCTTCTGTGTTTAGGTATTCTCTGCCACTCTTGCTGTGAAATTGAAGTGCATGTAGAAAAAACCTTTTACT
ATATGAAACTTTACAACACTTGTGAAAGCAACTCAATTTGGTTTATGCACAGTGTAATATTTCTCCAAGTATCATCCAAAATTCCC
CACAGACAAGGCTTTCGTCCTCATTAGGTGTTGGCCTCAGCCTAACCCTCTAGGACTGTTCTATTAAATTGCTGCCAGAATTTTACA
TCCAGTTACCTCCACTTTCTAGAACATATTCTTTACTAATGTTATTGAAACCAATTTCTACTTCATACTGATGTTTTTGGAAACAG
CAATTAAAGTTTTTCTTCCATGAGTTGAGTCCTTAAGAAAATGATTCCAGTTACTCATTTTGCATATTTGCTATTTTAACATTATT
GGACCCTGCATTTATAGTCCTTTGATTTCTTCCCTCTCCCTGGTGTCTCCCCCAAGACCCCAAATAAAGCAATACACTGTTAACACT
GTGGGTTTATATACTAATTCTATACCCCAGATGGGGAATGGGGGAGATGGTCCCTGGGCTTAATATTCTTTAAAGGGCATGGGAAT
TTAGCCTCTCTTTTATTGTAATGTGCTCTTTTGGAAAATAGTTGGTTAGCAGGGAAGACCCAGAGTTGTAGATTGAGATTAGGGTG
TACTGGCTGAACTGTGGAAAACATACAATTCTGTGTTCCTCAGTAAATGAGATTAGCGTCTAATGAGTAGCACCCCTTTACTAACT
TAGTAGTAGTATAAAATCATTTTTATTTAGTTAATTACCAGAGAGATTTAGCATAATTTTGTTCTGGATTCAGTAAATCAAGTCA
GCTTGGATCATTCACCTTAACTTTTCCTTTAGCAGCCATTTCCACTAGTTTCCATTAAGTAGTGTTCTATAAACTTTGATCCAAAGC
AGAATCAATGTCTTTTCCATCTCGTGACTTAAAGTTCTGTGACTGTGATGCATGTGAGTGTTCCGACTTCATCTGTTCCTCTTAACT
ACGGTGTTTCCCTTACCATGGCATTCATAGGATGAAATGAATGACTGCCCAGAATGAGAATTTGTCCAGATTATTCAGATAAACAT
CATAAAGCAGAATACATTATAAATAAGTAGAATATGAATAAATAGAATAATAAAATTCCAAAATACTCAATGGGAAATGACTAGT
AATATAGGCTTTCAAGAGTTGGTACCTTTTAGCTATATTTGCAGATTCTCTGGGATTTTAAGGAACTGAGAAAACAGCAAAGTTGA
CTAAATTTTATATTTCTTGTCCTCTAAATATTTTGATAATTTCTGGATTGATGCAGTGATGTTTTTGTTCCTTCCGTATTTATAAA
TGAAACACCTTTTTTTAGTGTTTCTAAACCTAAAATCTACTTGGTTTGAAATCAAGTGGTTGGAACACTGTTTGACTTTTATTTGA
AGCATGTTGTTGATTGAAAATTTCATTGAGGAAGTTTTCAATCAGTGTGATCAGTTTGATTCTGTAATGAGCACAGCACCTAATAT
TTTGAGGAGCTCTGTTTTGAGGACCAATGCTTAAGGTGGACTTTGTTCGTAAACAATATCCCAATAGATTTGTTGACTTGAGGTCT
GGTTTGGTTTTGTTTTTGTTTTGTTTTGTTTTGTTTTGTTTCCAATAGAATTAAGAATTCTAATGTTGAAAAACTGCACAAATTTT
TATGGGACAAAGCCTAGAAAAGAGAAATGTAGTTTGAATCATAATCTAAATCATCGTATGATAGAAGAGGGAAAGTTTTGGTGCC
ATAATTTCTCCTTTCACTGGTGTTGGACTTAAATCAGTTGAAATGTATTTCTGTACCACAATTTACGCTTCAATAAAAGTTTAATT
GTCTAGTGACATTCAG

>HIF1A
GCTTTTTCTTAATTTCATTCCTTTTTTTGGACACTGGTGGCTCATTACCTAAAGCAGTCTATTTATATTTTCTACATCTAATTTTAG
AAGCCTGGCTACAATACTGCACAAACTTGGTTAGTTCAATTTTGATCCCCTTTCTACTTAATTTACATTAATGCTCTTTTTTAGTA
TGTTCTTTAATGCTGGATCACAGACAGCTCATTTTCTCAGTTTTTTGGTATTTAAACCATTGCATTGCAGTAGCATCATTTTAAAA
AATGCACCTTTTTATTTATTTATTTTTGGCTAGGGAGTTTATCCCTTTTTCGAATTATTTTTAAGAAGATGCCAATATAATTTTTG
TAAGAAGGCAGTAACCTTTCATCATGATCATAGGCAGTTGAAAAATTTTTACACCTTTTTTTTCACATTTTACATAAATAATAATG
CTTTGCCAGCAGTACGTGGTAGCCACAATTGCACAATATATTTTCTTAAAAAATACCAGCAGTTACTCATGGAATATATTCTGCGT
TTATAAAACTAGTTTTTAAGAAGAAATTTTTTTTGGCCTATGAAATTGTTAAACCTGGAACATGACATTGTTAATCATATAATAA
TGATTCTTAAATGCTGTATGGTTTATTATTTAAATGGGTAAAGCCATTTACATAATATAGAAAGATATGCATATATCTAGAAGGT
ATGTGGCATTTATTTGGATAAAATTCTCAATTCAGAGAAATCATCTGATGTTTCTATAGTCACTTTGCCAGCTCAAAAGAAAACAA
TACCCTATGTAGTTGTGGAAGTTTATGCTAATATTGTGTAACTGATATTAAACCTAAATGTTCTGCCTACCCTGTTGGTATAAAGA
TATTTTGAGCAGACTGTAAACAAGAAAAAAAAAAATCATGCATTCTTAGCAAAATTGCCTAGTATGTTAATTTGCTCAAAATACAA
TGTTTGATTTTATGCACTTTGTCGCTATTAACATCCTTTTTTTCATGTAGATTTCAATAATTGAGTAATTTTAGAAGCATTATTTT
AGGAATATATAGTTGTCACAGTAAATATCTTGTTTTTTCTATGTACATTGTACAAATTTTTCATTCCTTTTGCTCTTTGTGGTTGG
ATCTAACACTAACTGTATTGTTTTGTTACATCAAATAAACATCTTCTGTGGACCAGGC

>MAD2L1
GGATGACATGAGGAAAATAATGTAATTGTAATTTTGAAATGTGGTTTTCCTGAAATCAAGTCATCTATAGTTGATATGTTTTATT
TCATTGGTTAATTTTTACATGGAGAAAACCAAAATGATACTTACTGAACTGTGTGTAATTGTTCCTTTTATTTTTTTGGTACCTAT
TTGACTTACCATGGAGTTAACATCATGAATTTATTGCACATTGTTCAAAAGGAACCAGGAGGTTTTTTTGTCAACATTGTGATGTA
TATTCCTTTGAAGATAGTAACTGTAGATGGAAAAACTTGTGCTATAAAGCTAGATGCTTTCCTAAATCAGATGTTTTGGTCAAGTA
GTTTGACTCAGTATAGGTAGGGAGATATTTAAGTATAAAATACAACAAAGGAAGTCTAAATATTCAGAATCTTTGTTAAGGTCCT
GAAAGTAACTCATAATCTATAAACAATGAAATATTGCTGTATAGCTCCTTTTGACCTTCATTTCATGTATAGTTTTCCCTATTGAA
TCAGTTTCCAATTATTTGACTTTAATTTATGTAACTTGAACCTATGAAGCAATGGATATTTGTACTGTTTAATGTTCTGTGATACA
GAACTCTTAAAAATGTTTTTTCATGTGTTTTATAAAATCAAGTTTTAAGTGAAAGTGAGGAAATAAAGTTAAGTTTGTTTTAAAT
TTGTCTTAAAA

>EIF4E
TTTCCTCATGTAACATTGGTGAAGGATCCAGGAATATGACACAAAGGTGGAATAAACATTAATTTTGTGCATTCTTTGGTAATTTT
TTTTGTTTTTTGTAACTACAAAGCTTTGCTACAAATTTATGCATTTCATTCAAATCAGTGATCTATGTTTGTGTGATTTCCTAAAC
ATAATTGTGGATTATAAAAAATGTAACATCATAATTACATTCCTAACTAGAATTAGTATGTCTGTTTTTGTATCTTTATGCTGTAT
TTTAACACTTTGTATTACTTAGGTTATTTTGCTTTGGTTAAAAATGGCTCAAGTAGAAAAGCAGTCCCATTCATATTAAGACAGTG
TACAAAACTGTAAATAAAATGTGTACAGTGAATTGTCTTTTAGACAACTAGATTTGTCCTTTTATTTCTCCATCTTTATAGAAGGA
ATTTGTACTTCTTATTGCAAGGCAGTCTCTATATTATGTCTTCTTTTGTGGTGTCTTCCATGTGAACAGCATAAGTTTGGAGCACT
AGTTTGATTATTATGTTTATTACAATTTTTAATAAATTGAATAGGTAGTATCATATATATGGAATTAAATTGATGTGGCTATCTT

Supplementary Figure S4: (*Continued*) Sequences of the nine positively validated 3′UTRs bound by Wig- 1. Putative ARE elements are highlighted in red, consensus 2D motifs are highlighted in green. (*Continued*)

TGTTTTTTTATAAAGTAAGGCACAGTCATTCAGTCTTAGGTAAATAATGTACTCTCTTAATATGTTAATACTCATGAGAATTGGGA
TCTGATGCATCACCATTTGATTGGTAGCAACAGTGGTTGTAAAACTTGGTTGCTGAATTGAGTTGTTTCTATGTTAAGTGTCAAAA
TGATAGTGTAGGGAAAGTACAGGTGGTGGGGACATATGCATTAAGAATCTTGTTAGTGTTGCAATCTAAATAGAATGGAATAAAC
AGGTGTTAAGACATATTTATAGTGGTAAATTGTTGTAGTATGGTATTCTGTAAACTTGAAAACTTGATCTACTCTTTGTAGGTATC
ATTTGAAAGCAAACTTGAAAATGTTTTGTACATAGTACATACTTGTATAGTCCTGTGAGATGAAGTATGGCTATCAGACCAAAGG
ATAAGCCAAACTGTAGGTAGCAGAATGGAAATTATTATTTTGAGAGGAAAATTTGTCTTTGAATGGTGATTATGACTTAATCATT
TTAAAACTGATAAACTTGACAAAAACCCTGTATGAAATAAACATGAAATTAATAGCACTGATTTCATTGTAAAATTTTAAAGCAG
TTTAAAGGGTACCACAGGTTATCACAGTACTCTCAATGCCACAAACACCTCTTGTTCAGTATTCTAGAAATACTGAATCAGAATTC
TGTGTTTATTATAATCTCAGCATACTGTACATAATATCTGCTAGTTAAACTTGGGTAATTGGTTAAGGTGACTTACTGTCTATGTC
AATATGTATAGTTTTGAGTACTTCAAGAGTTTACTTAAAAGTGATGATGTTACTGGTATGTTGGCAGTGGGTGGGACTGAAGTAG
TGTATCTATTATAAATTGATCTATTTTCTTAATTCTAAGATGAAGTCCAATTTTAAGCATCAGCTTTTAGGTGCAAAGGAGGAATT
AACACATTAAATGTATACAGTTCTAAATTTTTGAAATAACTGATGTGTAGCATTTGATTATTGGTATTACCATTTTAGAATCATGA
TGTTATTTTAAACCTTTTTCCTGGGGACAAGAAAGGATAATAAATTACGCTGAATCACTTTTGGCAGTTGCCACTTAAATAGTACA
GTGACTTGCAACTTTTATAACTTTATCAGCATCTTCTCTAAATACAAAATTAGGCTATATGTTATTTTCCAACTTACTGTTTTCTC
TCTGTTTAGCAGGATATTATAAATAGATTAAATAGATATATTTTCTTTTTTTTTTTTTTTTTTTTGAGACGGAGTCTCGCTTTGTCT
CCCAGGCTGGAGTGCAGTGGCGTGATCTCCCAGTAGCTGGGACTACAAGCACCTGCCACCATGCCCGGCTAATTTTTTTTGTATTTT
TAGTAGAGACGGGGTTTC

Supplementary Figure S4: (*Continued*) Sequences of the nine positively validated 3′UTRs bound by Wig- 1. Putative ARE elements are highlighted in red, consensus 2D motifs are highlighted in green.

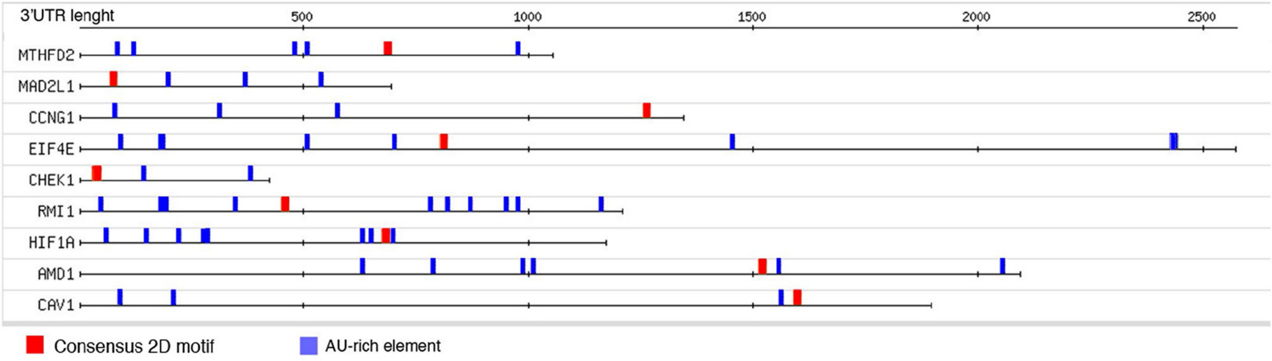

Supplementary Figure S5: Visualization the position of the consensus 2D motifs and the ARE motifs in the 3′UTR sequences of the validated Wig-1 bound targets. Figure generated by RSAT.

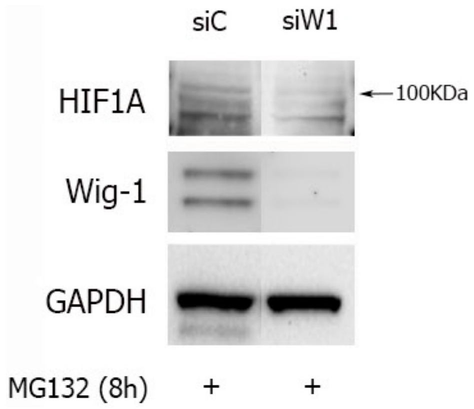

Supplementary Figure S6: Wig-1 knockdown does not affect HIF1A protein levels. HCT116 cells were transfected with 10nM siRNA against Wig-1 (siW1) or control siRNA (siC). Forty-eight hours after transfections cells were treated with 20 µM MG132 proteasome inhibitor for 8 h and the levels of nuclear HIF1A protein was measured by Western Blotting.

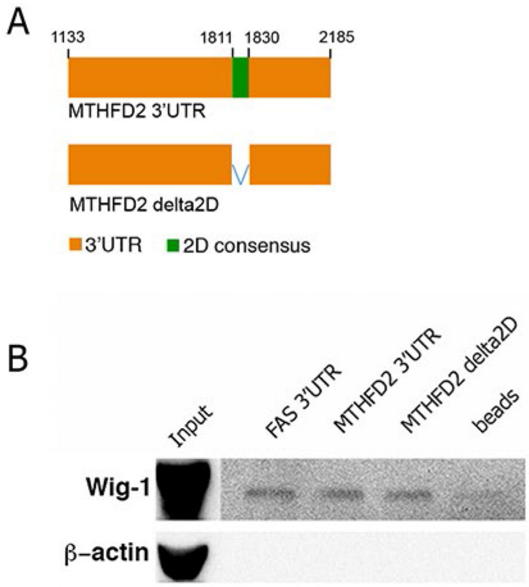

Supplementary Figure S7: A. Model of the MTHFD2 constructs used for biotin pulldown (see materials and methods for details)**. B.** Biotin pull-down assay using the probes in **(A.)** or a FAS full-length 3′UTR probe as positive control followed by Western blotting for Wig-1 shows that Wig-1 binds to the full length FAS 3′UTR (positive control), the full length MTHFD2 3′UTR probe and the MTHFD2 3′UTR probe with consensus 2D motif deletion (MTHFD2 delta2D) with the same intensity**.**

Supplementary Table S1: Percentage of reads mapped to human genome

Samples (*n*=3)	Average number of paired reads sequenced (MEAN ± S.D.)	Percentage of reads mapped to human genome (MEAN % ± S.D.)	Percentage of reads after duplicate removal (MEAN % ± S.D.)
HCT116 input.C	15,446,432 ± 1,253,607	64 ± 2.55	41 ± 1.25
HCT116 input.W	15,403,089 ± 318,777	62 ± 0.95	38 ± 1.43
HCT116 IP.C	15,466,119 ± 1,577,956	58 ± 11.73	20 ± 7.79
HCT116 IP.W	15,870,292 ± 2,191,340	61 ± 3.39	34 ± 1.81
Saos2 input.C	16,683,516 ± 6,422,055	70 ± 4.56	52 ± 4.43
Saos2 input.W	17,592,957 ± 5,131,259	71 ± 5.57	51 ± 6.56
Saos2 input. Wmut	19,000,702 ± 3,109,401	71 ± 3.76	53 ± 3.53
Saos2 IP.C	20,610,044 ± 11,505,023	41 ± 3.36	12 ± 8.10
Saos2 IP.W	17,340,104 ± 3,692,727	46 ± 35.38	28 ± 27.12
Saos2 IP.Wmut	21,239,267 ± 7,806,368	42 ± 4.13	8 ± 1.67

Input.C: input control (empty vector); input.W: input Flag-Wig-1; input.Wmut: input Flag-Wig-1-point mutant; IP.C: IP control (empty vector); IP.W: IP Flag-Wig-1; IP.Wmut: IP Flag-Wig-1point mutant.

Supplementary Table S2: List of the 2335 Wig-1-bound RNAs in HCT116 cells.

Supplementary Table S3: List of the 354 Wig-1-bound RNAs in Saos-2 cells.

Supplementary Table S4: List of the 286 common Wig-1-bound RNAs in HCT116 and Saos2 cells.

Supplementary Table S5: List of transcripts that are bound by Wig-1 and whose gene expression levels are affected by Wig-1 silencing in HCT116 cells (GEO database GSE43046), at least 4-fold difference (log2(siRNA Wig-1/siRNA control)) in at least two out of the three replicates.

Supplementary Table S6: Gene ontology Analysis of Wig-1-bound RNAs by DAVID.

Supplementary Table S7: List of the 300 pathways identified by network enrichment analysis as enriched at significance level FDR < 0.01.

Supplementary Table S8: List of the 133 Reactome pathways enriched at significant level of FDR < 0.01.

Supplementary Table S9: List of pathways identified by network enrichment analysis as enriched at significance level FDR < 0.01 for bound and regulated Wig-1 targets in HCT116 cell line.

Supplementary Table S10: List of the 286 RNAs included in the unbound group.

Supplementary Table S11: List of the TaqMan probes used for qRT-PCR.
