# Supplementary material for: Genome-wide identification of Wig-1 mRNA targets by RIP-Seq analysis
Source: Oncotarget. 2015 Dec 11;7(2):1895–911. doi: 10.18632/oncotarget.6557 (PMC4811505; doi:10.18632/oncotarget.6557)
Supplement: Supplementary file 2 [file oncotarget-07-1895-s002.docx]

| Supplementary Table S2: List of the 2335 Wig-1-bound RNAs in HCT116 cells. | | | |
| --- | --- | --- | --- |
|  |  |  |  |
| **Gene symbol** | **Ensembl ID** | **logFC** | **P.Value** |
| PHEX | ENSG00000102174 | 4,34 | 3,3E-04 |
| FMN1 | ENSG00000248905 | 4,30 | 3,7E-04 |
| RP11-515G11.1.1 | ENSG00000260531 | 4,15 | 3,3E-05 |
| HECTD2 | ENSG00000165338 | 4,06 | 9,1E-07 |
| SMAD9 | ENSG00000120693 | 4,04 | 6,2E-05 |
| SATL1 | ENSG00000184788 | 4,01 | 1,1E-07 |
| RERG | ENSG00000134533 | 3,99 | 1,5E-03 |
| SP3P | ENSG00000225530 | 3,94 | 7,6E-04 |
| FAM198B | ENSG00000164125 | 3,90 | 1,6E-04 |
| CNTF | ENSG00000242689 | 3,84 | 8,2E-04 |
| PFKFB1 | ENSG00000158571 | 3,72 | 9,4E-04 |
| CTD-2008P7.1 | ENSG00000260777 | 3,68 | 2,9E-03 |
| RP11-341B24.3 | ENSG00000233292 | 3,66 | 9,3E-04 |
| ITGA1 | ENSG00000213949 | 3,58 | 8,4E-04 |
| PDK4 | ENSG00000004799 | 3,58 | 3,0E-03 |
| PDE7B | ENSG00000171408 | 3,56 | 1,6E-03 |
| MACC1 | ENSG00000183742 | 3,54 | 4,4E-05 |
| SYNPO2 | ENSG00000172403 | 3,52 | 3,7E-03 |
| AC007383.4 | ENSG00000231955 | 3,49 | 4,7E-04 |
| TNFRSF19 | ENSG00000127863 | 3,46 | 6,1E-04 |
| RP11-16E18.3 | ENSG00000261542 | 3,41 | 1,3E-03 |
| RP11-408H20.1 | ENSG00000261521 | 3,39 | 1,6E-03 |
| RP11-1006G14.4 | ENSG00000260534 | 3,38 | 8,9E-04 |
| U4 | ENSG00000202181 | 3,38 | 2,5E-03 |
| LINC00849 | ENSG00000234635 | 3,37 | 2,8E-04 |
| RP11-350F4.2 | ENSG00000257742 | 3,36 | 1,3E-06 |
| RP11-1114A5.4 | ENSG00000232611 | 3,25 | 3,9E-03 |
| SNX7 | ENSG00000162627 | 3,23 | 9,5E-09 |
| IGIP | ENSG00000182700 | 3,23 | 1,4E-04 |
| TMEM135 | ENSG00000166575 | 3,21 | 5,1E-09 |
| CCNYL2 | ENSG00000182632 | 3,21 | 3,3E-07 |
| RP11-690D19.3 | ENSG00000260966 | 3,18 | 7,1E-04 |
| RP11-553L6.5 | ENSG00000259976 | 3,15 | 1,4E-05 |
| IL8 | ENSG00000169429 | 3,15 | 1,1E-06 |
| RP11-472B18.2 | ENSG00000238206 | 3,15 | 6,6E-04 |
| LEPR | ENSG00000116678 | 3,14 | 9,8E-07 |
| IPO7P1 | ENSG00000232951 | 3,12 | 3,0E-03 |
| EDNRA | ENSG00000151617 | 3,12 | 3,1E-04 |
| KIAA1430 | ENSG00000164323 | 3,11 | 2,0E-08 |
| GAS2L3 | ENSG00000139354 | 3,07 | 5,3E-07 |
| RAB27B | ENSG00000041353 | 3,06 | 2,1E-04 |
| RNF138 | ENSG00000134758 | 3,03 | 8,9E-08 |
| SNTB1 | ENSG00000172164 | 3,03 | 4,4E-05 |
| MRAP2 | ENSG00000135324 | 3,03 | 1,7E-04 |
| YTHDF3 | ENSG00000185728 | 3,01 | 8,7E-08 |
| RP11-501I18.2 | ENSG00000236163 | 3,01 | 3,4E-03 |
| RNF138P1 | ENSG00000250853 | 3,00 | 2,1E-04 |
| PDE8B | ENSG00000113231 | 2,99 | 3,2E-04 |
| LMAN1 | ENSG00000074695 | 2,99 | 2,2E-08 |
| RP11-698N11.4 | ENSG00000261355 | 2,99 | 5,9E-03 |
| CTD-2651C21.3 | ENSG00000254702 | 2,98 | 9,1E-04 |
| RP11-473O4.5 | ENSG00000254317 | 2,98 | 9,4E-04 |
| ANO5 | ENSG00000171714 | 2,98 | 2,0E-03 |
| HNF4G | ENSG00000164749 | 2,97 | 2,0E-04 |
| PRR5L | ENSG00000135362 | 2,97 | 6,2E-03 |
| RP11-1407O15.2 | ENSG00000174093 | 2,97 | 2,5E-03 |
| CA5B | ENSG00000169239 | 2,96 | 2,3E-04 |
| DENR | ENSG00000139726 | 2,96 | 1,2E-07 |
| PAQR3 | ENSG00000163291 | 2,96 | 3,6E-07 |
| CTC-462L7.1 | ENSG00000261589 | 2,95 | 1,0E-03 |
| DCLK1 | ENSG00000133083 | 2,95 | 1,1E-03 |
| RRM2B | ENSG00000048392 | 2,94 | 8,3E-08 |
| NAA30 | ENSG00000139977 | 2,94 | 7,8E-08 |
| THAP9 | ENSG00000168152 | 2,94 | 8,4E-06 |
| ABCD3 | ENSG00000117528 | 2,92 | 1,2E-07 |
| KPNA5 | ENSG00000196911 | 2,92 | 3,5E-07 |
| OSTM1 | ENSG00000081087 | 2,90 | 4,8E-08 |
| RP11-48B3.4 | ENSG00000260317 | 2,90 | 4,7E-03 |
| RP11-61A14.3 | ENSG00000261088 | 2,90 | 1,0E-05 |
| RNF217 | ENSG00000146373 | 2,90 | 7,9E-07 |
| FAM171B | ENSG00000144369 | 2,89 | 1,2E-07 |
| RP11-61A14.2 | ENSG00000261705 | 2,89 | 3,4E-03 |
| GDPD1 | ENSG00000153982 | 2,89 | 2,1E-06 |
| NEK7 | ENSG00000151414 | 2,88 | 1,2E-07 |
| RP11-517B11.7 | ENSG00000261167 | 2,88 | 5,1E-04 |
| RNU6-8 | ENSG00000202337 | 2,87 | 1,3E-03 |
| PLEKHF2 | ENSG00000175895 | 2,86 | 1,3E-07 |
| IL7 | ENSG00000104432 | 2,85 | 1,4E-05 |
| SGPP1 | ENSG00000126821 | 2,85 | 1,6E-06 |
| AC016716.1 | ENSG00000223427 | 2,85 | 1,6E-03 |
| PRDM1 | ENSG00000057657 | 2,85 | 7,3E-04 |
| PJA2 | ENSG00000198961 | 2,84 | 6,8E-08 |
| COX18 | ENSG00000163626 | 2,83 | 2,3E-06 |
| TMPO | ENSG00000120802 | 2,83 | 8,1E-08 |
| CRYGS | ENSG00000213139 | 2,83 | 4,6E-03 |
| LYPLA1 | ENSG00000120992 | 2,82 | 1,1E-07 |
| SGMS2 | ENSG00000164023 | 2,82 | 3,3E-06 |
| RP13-923O23.7.1 | ENSG00000260397 | 2,82 | 3,3E-03 |
| ZDHHC21 | ENSG00000175893 | 2,82 | 1,2E-06 |
| RAB14 | ENSG00000119396 | 2,81 | 7,3E-08 |
| MUM1L1 | ENSG00000157502 | 2,81 | 2,0E-04 |
| RPA2P3 | ENSG00000255547 | 2,81 | 5,5E-03 |
| ZDHHC20 | ENSG00000215570 | 2,81 | 9,7E-08 |
| HEATR3 | ENSG00000155393 | 2,80 | 2,6E-06 |
| FPGT | ENSG00000254685 | 2,80 | 1,9E-06 |
| NMD3 | ENSG00000169251 | 2,79 | 2,0E-07 |
| KLRC3 | ENSG00000205810 | 2,79 | 6,3E-03 |
| IARS2 | ENSG00000067704 | 2,79 | 8,8E-08 |
| SP4 | ENSG00000105866 | 2,79 | 7,2E-06 |
| ZBTB41 | ENSG00000177888 | 2,78 | 6,5E-04 |
| MOB1B | ENSG00000173542 | 2,78 | 4,7E-07 |
| TTPA | ENSG00000137561 | 2,78 | 1,9E-03 |
| PLGRKT | ENSG00000107020 | 2,78 | 1,2E-07 |
| C21orf91 | ENSG00000154642 | 2,77 | 1,2E-05 |
| BPNT1 | ENSG00000162813 | 2,77 | 2,8E-07 |
| TAF13 | ENSG00000197780 | 2,77 | 1,9E-07 |
| AHCYL2 | ENSG00000158467 | 2,76 | 9,1E-06 |
| MTMR6 | ENSG00000139505 | 2,76 | 1,8E-06 |
| STRN | ENSG00000115808 | 2,76 | 3,3E-06 |
| EXOC5 | ENSG00000070367 | 2,76 | 1,9E-07 |
| PIK3R1 | ENSG00000145675 | 2,76 | 1,3E-04 |
| TMEM30A | ENSG00000112697 | 2,75 | 1,9E-07 |
| RP11-25G10.2 | ENSG00000232739 | 2,75 | 5,7E-03 |
| SLC26A2 | ENSG00000155850 | 2,75 | 2,6E-06 |
| FAM102B | ENSG00000162636 | 2,75 | 1,1E-06 |
| EIF5A2 | ENSG00000163577 | 2,75 | 1,1E-06 |
| SLC2A13 | ENSG00000151229 | 2,75 | 6,2E-06 |
| SDR16C5 | ENSG00000170786 | 2,74 | 5,3E-05 |
| RP11-227D13.2 | ENSG00000259216 | 2,74 | 4,0E-03 |
| LYST-IT1 | ENSG00000226135 | 2,74 | 3,5E-03 |
| SLC39A8 | ENSG00000138821 | 2,74 | 4,0E-07 |
| FAM117B | ENSG00000138439 | 2,73 | 4,8E-05 |
| TMEM117 | ENSG00000139173 | 2,73 | 1,1E-04 |
| ENPP4 | ENSG00000001561 | 2,73 | 5,7E-07 |
| ARL5B | ENSG00000165997 | 2,73 | 5,2E-08 |
| EIF2AK2 | ENSG00000055332 | 2,72 | 1,1E-07 |
| CLOCK | ENSG00000134852 | 2,72 | 2,5E-07 |
| KIAA1715 | ENSG00000144320 | 2,72 | 4,0E-07 |
| ZNF708 | ENSG00000182141 | 2,72 | 1,9E-05 |
| ZFR | ENSG00000056097 | 2,71 | 3,3E-07 |
| FAM73A | ENSG00000180488 | 2,71 | 2,6E-04 |
| FAM35CP | ENSG00000259096 | 2,70 | 3,0E-04 |
| TMEM64 | ENSG00000180694 | 2,70 | 8,6E-07 |
| KITLG | ENSG00000049130 | 2,70 | 3,4E-08 |
| RP3-481A17.1 | ENSG00000232332 | 2,70 | 1,6E-03 |
| ELMOD2 | ENSG00000179387 | 2,70 | 3,3E-07 |
| RP2 | ENSG00000102218 | 2,70 | 7,3E-06 |
| MCTP1 | ENSG00000175471 | 2,70 | 2,9E-03 |
| SP3 | ENSG00000172845 | 2,70 | 7,3E-08 |
| ATP13A3 | ENSG00000133657 | 2,69 | 7,6E-07 |
| STARD4 | ENSG00000164211 | 2,69 | 7,6E-08 |
| MAGT1 | ENSG00000102158 | 2,69 | 2,6E-07 |
| MTMR12 | ENSG00000150712 | 2,69 | 3,1E-07 |
| RP11-156E6.1 | ENSG00000259623 | 2,69 | 7,2E-06 |
| PDIK1L | ENSG00000175087 | 2,69 | 4,8E-06 |
| UFL1 | ENSG00000014123 | 2,68 | 7,0E-07 |
| ZNF100 | ENSG00000197020 | 2,68 | 5,9E-06 |
| CALCRL | ENSG00000064989 | 2,67 | 1,2E-03 |
| PTGS2 | ENSG00000073756 | 2,67 | 7,4E-04 |
| PRKAR2A | ENSG00000114302 | 2,67 | 2,9E-07 |
| RP11-154D6.1 | ENSG00000232295 | 2,66 | 2,0E-05 |
| HOOK1 | ENSG00000134709 | 2,66 | 2,5E-06 |
| ECT2 | ENSG00000114346 | 2,66 | 1,7E-07 |
| PHTF2 | ENSG00000006576 | 2,65 | 4,6E-07 |
| CCDC126 | ENSG00000169193 | 2,65 | 1,7E-06 |
| FAM199X | ENSG00000123575 | 2,65 | 3,8E-07 |
| SLC35A3 | ENSG00000117620 | 2,65 | 6,9E-07 |
| RAP2C | ENSG00000123728 | 2,65 | 1,5E-07 |
| ARL5A | ENSG00000162980 | 2,64 | 1,8E-07 |
| ZNF816 | ENSG00000180257 | 2,64 | 2,4E-03 |
| RAB11FIP2 | ENSG00000107560 | 2,64 | 3,9E-06 |
| MED18 | ENSG00000130772 | 2,64 | 4,1E-07 |
| NBPF12 | ENSG00000186275 | 2,64 | 8,5E-04 |
| CXADR | ENSG00000154639 | 2,62 | 1,3E-06 |
| CNOT6L | ENSG00000138767 | 2,62 | 1,0E-06 |
| UHMK1 | ENSG00000152332 | 2,62 | 5,2E-07 |
| C4orf32 | ENSG00000174749 | 2,61 | 4,6E-07 |
| FAM46A | ENSG00000112773 | 2,61 | 4,3E-06 |
| LRRC58 | ENSG00000163428 | 2,61 | 1,6E-06 |
| SESN3 | ENSG00000149212 | 2,61 | 2,5E-04 |
| CRADD | ENSG00000169372 | 2,61 | 8,3E-07 |
| TBL1XR1 | ENSG00000177565 | 2,60 | 6,8E-07 |
| MMGT1 | ENSG00000169446 | 2,60 | 1,9E-06 |
| TWSG1 | ENSG00000128791 | 2,60 | 7,9E-07 |
| FOXN2 | ENSG00000170802 | 2,60 | 2,8E-06 |
| GLS | ENSG00000115419 | 2,60 | 1,1E-06 |
| BLOC1S6 | ENSG00000104164 | 2,60 | 3,8E-07 |
| RP11-361F15.2 | ENSG00000225135 | 2,59 | 3,4E-04 |
| DNAJB14 | ENSG00000164031 | 2,59 | 2,8E-07 |
| NSL1 | ENSG00000117697 | 2,59 | 5,6E-07 |
| CCNG1 | ENSG00000113328 | 2,59 | 5,5E-08 |
| SLC30A1 | ENSG00000170385 | 2,59 | 1,2E-05 |
| C2orf69 | ENSG00000178074 | 2,59 | 8,0E-07 |
| KLHL23 | ENSG00000213160 | 2,59 | 5,4E-07 |
| FGF2 | ENSG00000138685 | 2,59 | 5,2E-06 |
| CPEB4 | ENSG00000113742 | 2,59 | 2,0E-06 |
| PAPSS2 | ENSG00000198682 | 2,59 | 7,1E-06 |
| SLC30A4 | ENSG00000104154 | 2,58 | 2,0E-04 |
| ICK | ENSG00000112144 | 2,58 | 2,1E-06 |
| AKT3 | ENSG00000117020 | 2,58 | 2,0E-04 |
| MBNL3 | ENSG00000076770 | 2,58 | 1,1E-03 |
| FKTN | ENSG00000106692 | 2,58 | 1,8E-06 |
| RP11-4O1.2 | ENSG00000259953 | 2,58 | 2,2E-03 |
| UBE2Q2 | ENSG00000140367 | 2,57 | 8,4E-07 |
| PRKCI | ENSG00000163558 | 2,57 | 1,1E-06 |
| ST8SIA6 | ENSG00000148488 | 2,57 | 5,6E-03 |
| GPR180 | ENSG00000152749 | 2,57 | 1,2E-06 |
| TWISTNB | ENSG00000105849 | 2,57 | 9,6E-06 |
| MAN2A1 | ENSG00000112893 | 2,57 | 1,4E-05 |
| CMPK2 | ENSG00000134326 | 2,57 | 3,2E-03 |
| TSNAX | ENSG00000116918 | 2,57 | 4,6E-07 |
| MAN1A2 | ENSG00000198162 | 2,57 | 3,7E-06 |
| C7orf60 | ENSG00000164603 | 2,56 | 4,1E-05 |
| GTDC1 | ENSG00000121964 | 2,56 | 9,8E-07 |
| NAA50 | ENSG00000121579 | 2,56 | 2,2E-07 |
| XK | ENSG00000047597 | 2,56 | 3,1E-05 |
| NMD3P1 | ENSG00000226342 | 2,56 | 2,4E-04 |
| GNA13 | ENSG00000120063 | 2,55 | 4,3E-07 |
| DENND1B | ENSG00000213047 | 2,55 | 5,6E-06 |
| BMI1 | ENSG00000168283 | 2,55 | 4,7E-07 |
| COMMD8 | ENSG00000169019 | 2,55 | 2,2E-06 |
| PROS1 | ENSG00000184500 | 2,55 | 4,9E-04 |
| CASC5 | ENSG00000137812 | 2,54 | 1,2E-06 |
| CCNYL1 | ENSG00000163249 | 2,54 | 1,0E-06 |
| SLC25A36 | ENSG00000114120 | 2,54 | 1,3E-07 |
| C9orf41 | ENSG00000156017 | 2,54 | 2,1E-06 |
| SELT | ENSG00000198843 | 2,53 | 2,0E-06 |
| GALNT7 | ENSG00000109586 | 2,53 | 2,3E-06 |
| TMEM56 | ENSG00000152078 | 2,53 | 3,6E-06 |
| EPC2 | ENSG00000135999 | 2,53 | 1,3E-05 |
| TMX3 | ENSG00000166479 | 2,53 | 6,6E-07 |
| RP11-697E2.7 | ENSG00000228998 | 2,53 | 1,0E-04 |
| EREG | ENSG00000124882 | 2,53 | 3,0E-07 |
| WDR72 | ENSG00000166415 | 2,53 | 1,9E-06 |
| FAM91A1 | ENSG00000176853 | 2,52 | 2,2E-07 |
| PIK3CB | ENSG00000051382 | 2,52 | 6,4E-07 |
| SLC25A21 | ENSG00000183032 | 2,52 | 1,4E-03 |
| UBE2WP1 | ENSG00000234422 | 2,52 | 5,6E-03 |
| PRKAA2 | ENSG00000162409 | 2,51 | 2,0E-05 |
| CDK6 | ENSG00000105810 | 2,51 | 2,6E-06 |
| MREG | ENSG00000118242 | 2,51 | 5,4E-07 |
| ZNF365 | ENSG00000138311 | 2,50 | 1,2E-03 |
| AC009299.3 | ENSG00000227403 | 2,50 | 6,5E-06 |
| FAM222A-AS1 | ENSG00000255650 | 2,50 | 8,4E-04 |
| DNAJB9 | ENSG00000128590 | 2,50 | 4,5E-06 |
| ZC2HC1A | ENSG00000104427 | 2,50 | 1,7E-03 |
| RP11-121C2.2 | ENSG00000259959 | 2,50 | 1,7E-03 |
| DDX50P1 | ENSG00000229816 | 2,49 | 8,4E-04 |
| NBPF3 | ENSG00000142794 | 2,49 | 7,1E-05 |
| BACH1 | ENSG00000156273 | 2,49 | 2,1E-06 |
| STS | ENSG00000101846 | 2,49 | 5,7E-03 |
| HDHD2 | ENSG00000167220 | 2,48 | 4,9E-06 |
| PM20D2 | ENSG00000146281 | 2,48 | 1,7E-06 |
| RP11-226L15.5 | ENSG00000260766 | 2,48 | 2,8E-03 |
| RBM7 | ENSG00000076053 | 2,48 | 2,9E-06 |
| ZNF320 | ENSG00000182986 | 2,48 | 3,3E-05 |
| RICTOR | ENSG00000164327 | 2,48 | 9,9E-06 |
| XPR1 | ENSG00000143324 | 2,48 | 1,0E-06 |
| ATG12 | ENSG00000145782 | 2,48 | 1,2E-06 |
| MBNL1 | ENSG00000152601 | 2,47 | 7,0E-07 |
| HECA | ENSG00000112406 | 2,47 | 2,3E-05 |
| ABCB10 | ENSG00000135776 | 2,47 | 2,3E-06 |
| PUS7L | ENSG00000129317 | 2,47 | 2,9E-05 |
| SPRED1 | ENSG00000166068 | 2,47 | 8,7E-07 |
| NAPEPLD | ENSG00000161048 | 2,47 | 1,8E-05 |
| CPNE8 | ENSG00000139117 | 2,46 | 7,4E-06 |
| KDM7A | ENSG00000006459 | 2,46 | 5,1E-04 |
| TMEM161BP1 | ENSG00000237970 | 2,46 | 3,3E-03 |
| RASA2 | ENSG00000155903 | 2,46 | 4,9E-05 |
| MST4 | ENSG00000134602 | 2,46 | 2,1E-06 |
| CTC-428G20.3 | ENSG00000248175 | 2,46 | 2,0E-03 |
| CASD1 | ENSG00000127995 | 2,45 | 6,6E-06 |
| CLIC4 | ENSG00000169504 | 2,45 | 1,0E-06 |
| ACSL3 | ENSG00000123983 | 2,45 | 1,3E-07 |
| PDP2 | ENSG00000172840 | 2,45 | 1,4E-03 |
| HS3ST3B1 | ENSG00000125430 | 2,45 | 1,2E-03 |
| NR1D2 | ENSG00000174738 | 2,45 | 1,0E-04 |
| DCP2 | ENSG00000172795 | 2,45 | 2,3E-06 |
| SLC25A40 | ENSG00000075303 | 2,45 | 4,9E-07 |
| HS2ST1 | ENSG00000153936 | 2,45 | 1,1E-06 |
| RGS5 | ENSG00000143248 | 2,44 | 2,2E-03 |
| NEBL | ENSG00000078114 | 2,44 | 1,2E-04 |
| ACAP2 | ENSG00000114331 | 2,44 | 1,1E-06 |
| CCSAP | ENSG00000154429 | 2,44 | 1,0E-05 |
| ESCO2 | ENSG00000171320 | 2,44 | 7,5E-06 |
| ABHD13 | ENSG00000139826 | 2,44 | 6,4E-06 |
| RP11-469M7.1 | ENSG00000260006 | 2,44 | 5,4E-04 |
| IPMK | ENSG00000151151 | 2,44 | 3,0E-06 |
| BNIP3L | ENSG00000104765 | 2,43 | 2,2E-05 |
| RMI1 | ENSG00000178966 | 2,43 | 2,1E-06 |
| GPR37 | ENSG00000170775 | 2,43 | 5,3E-03 |
| AMER1 | ENSG00000184675 | 2,43 | 6,0E-03 |
| UGCG | ENSG00000148154 | 2,43 | 1,8E-06 |
| MAP3K1 | ENSG00000095015 | 2,43 | 4,3E-05 |
| UPRT | ENSG00000094841 | 2,43 | 3,5E-05 |
| DENND6A | ENSG00000174839 | 2,43 | 1,8E-06 |
| PPP1CB | ENSG00000213639 | 2,42 | 3,8E-07 |
| GPRASP2 | ENSG00000158301 | 2,42 | 2,2E-03 |
| ZNF277 | ENSG00000198839 | 2,42 | 1,4E-06 |
| C12orf23 | ENSG00000151135 | 2,42 | 4,3E-06 |
| SOCS4 | ENSG00000180008 | 2,42 | 4,1E-06 |
| GNG12 | ENSG00000172380 | 2,42 | 1,4E-06 |
| RP11-305E6.4 | ENSG00000259994 | 2,42 | 2,8E-04 |
| VASH2 | ENSG00000143494 | 2,42 | 3,4E-05 |
| RP11-366L20.2 | ENSG00000197301 | 2,42 | 5,2E-03 |
| GOLT1B | ENSG00000111711 | 2,41 | 1,5E-06 |
| MGAT4A | ENSG00000071073 | 2,41 | 1,3E-05 |
| TRUB1 | ENSG00000165832 | 2,41 | 4,4E-06 |
| EXOC8 | ENSG00000116903 | 2,41 | 9,1E-05 |
| RP11-718G2.4 | ENSG00000258889 | 2,41 | 3,1E-04 |
| RAB23 | ENSG00000112210 | 2,41 | 9,3E-07 |
| PTPLB | ENSG00000206527 | 2,41 | 1,2E-05 |
| CPNE3 | ENSG00000085719 | 2,41 | 6,7E-07 |
| TMEM161B | ENSG00000164180 | 2,41 | 2,1E-06 |
| RNF128 | ENSG00000133135 | 2,41 | 5,7E-04 |
| TROVE2 | ENSG00000116747 | 2,40 | 1,6E-06 |
| ZFP30 | ENSG00000120784 | 2,40 | 1,2E-03 |
| FNIP1 | ENSG00000217128 | 2,40 | 1,3E-05 |
| ATL1 | ENSG00000198513 | 2,40 | 1,6E-03 |
| INSIG2 | ENSG00000125629 | 2,40 | 3,6E-06 |
| SNX10 | ENSG00000086300 | 2,40 | 1,7E-06 |
| TMED5 | ENSG00000117500 | 2,40 | 1,8E-06 |
| N4BP2 | ENSG00000078177 | 2,40 | 5,3E-05 |
| ATP11C | ENSG00000101974 | 2,40 | 2,3E-06 |
| SLC25A32 | ENSG00000164933 | 2,39 | 3,9E-06 |
| HACE1 | ENSG00000085382 | 2,39 | 3,9E-06 |
| CHAC2 | ENSG00000143942 | 2,39 | 4,6E-05 |
| EIF4E3 | ENSG00000163412 | 2,39 | 4,8E-06 |
| FCHO2 | ENSG00000157107 | 2,39 | 5,2E-05 |
| MANEA | ENSG00000172469 | 2,39 | 9,0E-06 |
| ZNF654 | ENSG00000175105 | 2,39 | 1,7E-04 |
| MEF2C | ENSG00000081189 | 2,39 | 7,5E-05 |
| MIB1 | ENSG00000101752 | 2,39 | 2,6E-05 |
| OTUD7B | ENSG00000163113 | 2,39 | 6,1E-05 |
| C6orf211 | ENSG00000146476 | 2,39 | 9,2E-06 |
| UTP14C | ENSG00000253797 | 2,39 | 2,7E-05 |
| SPATA7 | ENSG00000042317 | 2,38 | 7,3E-04 |
| SIRT1 | ENSG00000096717 | 2,38 | 5,5E-05 |
| SENP7 | ENSG00000138468 | 2,38 | 5,2E-05 |
| ELOVL7 | ENSG00000164181 | 2,38 | 2,9E-06 |
| TMEM45A | ENSG00000181458 | 2,38 | 1,5E-04 |
| THAP5 | ENSG00000177683 | 2,38 | 1,6E-05 |
| FZD3 | ENSG00000104290 | 2,38 | 2,2E-06 |
| WASL | ENSG00000106299 | 2,38 | 2,2E-06 |
| TMEM133 | ENSG00000170647 | 2,38 | 1,2E-04 |
| LANCL1 | ENSG00000115365 | 2,38 | 1,7E-06 |
| LCOR | ENSG00000196233 | 2,38 | 3,3E-05 |
| RP11-50D9.3 | ENSG00000261668 | 2,37 | 5,5E-03 |
| PTAR1 | ENSG00000188647 | 2,37 | 2,8E-06 |
| GDA | ENSG00000119125 | 2,37 | 1,6E-06 |
| TMEM38B | ENSG00000095209 | 2,37 | 5,2E-06 |
| KIAA1033 | ENSG00000136051 | 2,37 | 5,2E-06 |
| LIFR | ENSG00000113594 | 2,37 | 1,5E-05 |
| ZDHHC20 | ENSG00000180776 | 2,36 | 5,9E-03 |
| ZMPSTE24 | ENSG00000084073 | 2,36 | 4,7E-05 |
| FOPNL | ENSG00000133393 | 2,36 | 7,0E-07 |
| SLC22A15 | ENSG00000163393 | 2,36 | 1,0E-03 |
| PLOD2 | ENSG00000152952 | 2,36 | 1,0E-05 |
| SLC44A1 | ENSG00000070214 | 2,36 | 2,2E-05 |
| SLC35E3 | ENSG00000175782 | 2,36 | 7,1E-05 |
| NETO2 | ENSG00000171208 | 2,36 | 4,4E-06 |
| RP11-296K13.1.1 | ENSG00000260110 | 2,36 | 8,6E-06 |
| ZMYM1 | ENSG00000197056 | 2,36 | 1,8E-05 |
| GK5 | ENSG00000175066 | 2,36 | 3,4E-04 |
| ZBTB33 | ENSG00000177485 | 2,36 | 2,0E-06 |
| TGFBR1 | ENSG00000106799 | 2,35 | 1,0E-05 |
| TMEM170A | ENSG00000166822 | 2,35 | 4,8E-06 |
| PDE5A | ENSG00000138735 | 2,35 | 5,8E-04 |
| SLC44A5 | ENSG00000137968 | 2,35 | 4,0E-04 |
| ASAH2 | ENSG00000188611 | 2,35 | 3,5E-03 |
| CNIH1 | ENSG00000100528 | 2,35 | 1,3E-06 |
| EIF1AX | ENSG00000173674 | 2,34 | 8,1E-06 |
| SLC7A11 | ENSG00000151012 | 2,34 | 1,9E-05 |
| TMEM59 | ENSG00000116209 | 2,34 | 8,5E-07 |
| LPP | ENSG00000145012 | 2,34 | 5,1E-05 |
| SPAST | ENSG00000021574 | 2,34 | 5,0E-06 |
| SCAMP1 | ENSG00000085365 | 2,34 | 1,2E-06 |
| C5orf24 | ENSG00000181904 | 2,34 | 2,8E-06 |
| UBXN2B | ENSG00000215114 | 2,34 | 2,8E-06 |
| FAM3C | ENSG00000196937 | 2,33 | 1,8E-06 |
| MBTPS2 | ENSG00000012174 | 2,33 | 4,4E-06 |
| SPIRE1 | ENSG00000134278 | 2,33 | 5,4E-06 |
| ALG10 | ENSG00000139133 | 2,33 | 4,3E-04 |
| GS1-358P8.4 | ENSG00000260822 | 2,33 | 3,3E-04 |
| ZWILCH | ENSG00000174442 | 2,33 | 9,7E-06 |
| RALGAPA1 | ENSG00000174373 | 2,33 | 1,6E-04 |
| LNX2 | ENSG00000139517 | 2,33 | 1,1E-05 |
| RP11-318L16.6 | ENSG00000261252 | 2,33 | 5,8E-03 |
| SUZ12 | ENSG00000178691 | 2,33 | 1,9E-06 |
| NDFIP2 | ENSG00000102471 | 2,32 | 1,1E-05 |
| FAM160B1 | ENSG00000151553 | 2,32 | 1,3E-05 |
| TMEM87B | ENSG00000153214 | 2,32 | 1,6E-05 |
| TMEM243 | ENSG00000135185 | 2,32 | 1,5E-05 |
| TNPO1 | ENSG00000083312 | 2,32 | 5,7E-07 |
| RP11-568K15.1 | ENSG00000242193 | 2,32 | 1,6E-04 |
| IL6ST | ENSG00000134352 | 2,32 | 3,8E-06 |
| APPL1 | ENSG00000157500 | 2,32 | 2,8E-06 |
| TMEM200A | ENSG00000164484 | 2,32 | 1,8E-05 |
| PPAT | ENSG00000128059 | 2,31 | 1,6E-06 |
| FOXO1 | ENSG00000150907 | 2,31 | 2,2E-04 |
| ZNF800 | ENSG00000048405 | 2,31 | 1,4E-05 |
| TTC8 | ENSG00000165533 | 2,31 | 7,4E-06 |
| EVI5 | ENSG00000067208 | 2,31 | 8,1E-06 |
| PRRG4 | ENSG00000135378 | 2,31 | 1,4E-04 |
| FUBP3 | ENSG00000107164 | 2,31 | 2,5E-06 |
| MBOAT2 | ENSG00000143797 | 2,31 | 1,3E-05 |
| AHR | ENSG00000106546 | 2,31 | 3,2E-05 |
| RFK | ENSG00000135002 | 2,31 | 8,5E-06 |
| RUNDC3B | ENSG00000105784 | 2,30 | 3,9E-04 |
| CAV2 | ENSG00000105971 | 2,30 | 6,8E-06 |
| GPAM | ENSG00000119927 | 2,30 | 6,0E-05 |
| NIPSNAP3A | ENSG00000136783 | 2,30 | 5,3E-05 |
| CPD | ENSG00000108582 | 2,30 | 4,0E-06 |
| NXT2 | ENSG00000101888 | 2,29 | 1,8E-05 |
| GALNT1 | ENSG00000141429 | 2,29 | 2,6E-06 |
| LBR | ENSG00000143815 | 2,29 | 1,6E-06 |
| LPCAT2 | ENSG00000087253 | 2,29 | 1,8E-05 |
| GMFB | ENSG00000197045 | 2,29 | 6,6E-06 |
| UBE2W | ENSG00000104343 | 2,29 | 8,7E-07 |
| SPOPL | ENSG00000144228 | 2,29 | 3,8E-06 |
| PIGN | ENSG00000197563 | 2,29 | 7,0E-05 |
| CRCP | ENSG00000241258 | 2,28 | 2,3E-06 |
| OSGIN2 | ENSG00000164823 | 2,28 | 2,3E-06 |
| GULP1 | ENSG00000144366 | 2,28 | 1,1E-05 |
| MKLN1 | ENSG00000128585 | 2,28 | 1,9E-06 |
| NAA25 | ENSG00000111300 | 2,28 | 2,3E-06 |
| FAR1 | ENSG00000197601 | 2,28 | 3,3E-06 |
| RAB33B | ENSG00000172007 | 2,28 | 6,9E-05 |
| ZNF543 | ENSG00000178229 | 2,28 | 6,1E-03 |
| PPP3CA | ENSG00000138814 | 2,28 | 1,0E-06 |
| TMEM170B | ENSG00000205269 | 2,28 | 1,0E-03 |
| SOCS6 | ENSG00000170677 | 2,28 | 3,1E-06 |
| RBM24 | ENSG00000112183 | 2,28 | 4,2E-04 |
| SCML2 | ENSG00000102098 | 2,27 | 3,0E-04 |
| MMD | ENSG00000108960 | 2,27 | 1,9E-05 |
| GSKIP | ENSG00000100744 | 2,27 | 2,9E-06 |
| HCFC2 | ENSG00000111727 | 2,27 | 2,6E-04 |
| KLHL8 | ENSG00000145332 | 2,27 | 4,0E-06 |
| KLF6 | ENSG00000067082 | 2,27 | 1,2E-05 |
| SPCS3 | ENSG00000129128 | 2,27 | 1,6E-06 |
| SLC2A12 | ENSG00000146411 | 2,27 | 2,5E-03 |
| PRKD3 | ENSG00000115825 | 2,27 | 5,4E-06 |
| RTKN2 | ENSG00000182010 | 2,26 | 2,6E-06 |
| CASP10 | ENSG00000003400 | 2,26 | 6,1E-03 |
| LIN7C | ENSG00000148943 | 2,26 | 2,5E-06 |
| SLC4A7 | ENSG00000033867 | 2,26 | 8,8E-06 |
| FBXL20 | ENSG00000108306 | 2,26 | 4,8E-04 |
| TMEM154 | ENSG00000170006 | 2,26 | 5,1E-03 |
| RP1-145M24.1 | ENSG00000254708 | 2,26 | 3,3E-04 |
| KBTBD8 | ENSG00000163376 | 2,26 | 1,3E-03 |
| RP11-421F16.3 | ENSG00000247903 | 2,26 | 6,1E-05 |
| AK3 | ENSG00000147853 | 2,26 | 1,5E-06 |
| SOCS5 | ENSG00000171150 | 2,26 | 5,1E-06 |
| CACNB4 | ENSG00000182389 | 2,26 | 5,1E-04 |
| LGR4 | ENSG00000205213 | 2,25 | 3,3E-04 |
| RBPJ | ENSG00000168214 | 2,25 | 3,0E-06 |
| SSBP2 | ENSG00000145687 | 2,25 | 4,1E-04 |
| ZNF329 | ENSG00000181894 | 2,25 | 2,5E-03 |
| KRAS | ENSG00000133703 | 2,25 | 5,8E-06 |
| BMPR1APS2 | ENSG00000232460 | 2,25 | 2,8E-03 |
| CNOT7 | ENSG00000198791 | 2,25 | 1,7E-06 |
| LCA5 | ENSG00000135338 | 2,25 | 1,4E-03 |
| SEC23A | ENSG00000100934 | 2,25 | 1,2E-06 |
| ZBTB21 | ENSG00000173276 | 2,24 | 3,6E-06 |
| TRIM2 | ENSG00000109654 | 2,24 | 3,9E-06 |
| PUM2 | ENSG00000055917 | 2,24 | 6,0E-06 |
| SGK3 | ENSG00000104205 | 2,24 | 3,4E-05 |
| YOD1 | ENSG00000180667 | 2,24 | 3,7E-05 |
| GZF1 | ENSG00000125812 | 2,24 | 3,5E-05 |
| FAM8A1 | ENSG00000137414 | 2,24 | 3,0E-06 |
| MDFIC | ENSG00000135272 | 2,24 | 2,0E-05 |
| LINS | ENSG00000140471 | 2,24 | 3,7E-05 |
| USP12 | ENSG00000152484 | 2,23 | 2,4E-06 |
| C4orf29 | ENSG00000164074 | 2,23 | 4,3E-05 |
| ZBTB6 | ENSG00000186130 | 2,23 | 1,4E-05 |
| CDC14A | ENSG00000079335 | 2,23 | 3,0E-04 |
| TAF2 | ENSG00000064313 | 2,23 | 5,4E-06 |
| DCUN1D1 | ENSG00000043093 | 2,23 | 1,5E-06 |
| YIPF4 | ENSG00000119820 | 2,22 | 9,1E-06 |
| AC108676.1 | ENSG00000244675 | 2,22 | 4,4E-04 |
| MIER3 | ENSG00000155545 | 2,22 | 6,3E-06 |
| ARMC1 | ENSG00000104442 | 2,22 | 2,8E-06 |
| ASB7 | ENSG00000183475 | 2,22 | 2,9E-05 |
| SLC39A10 | ENSG00000196950 | 2,22 | 1,3E-05 |
| CGGBP1 | ENSG00000163320 | 2,22 | 1,1E-06 |
| PANK3 | ENSG00000120137 | 2,22 | 6,1E-06 |
| PGGT1B | ENSG00000164219 | 2,22 | 9,4E-05 |
| TRHDE | ENSG00000072657 | 2,22 | 2,3E-03 |
| KLF11 | ENSG00000172059 | 2,22 | 2,1E-04 |
| NUS1 | ENSG00000153989 | 2,22 | 6,2E-06 |
| DR1 | ENSG00000117505 | 2,22 | 1,6E-06 |
| SNRNP48 | ENSG00000168566 | 2,22 | 5,6E-06 |
| ZIC5 | ENSG00000139800 | 2,22 | 2,8E-06 |
| XIAP | ENSG00000101966 | 2,21 | 9,0E-06 |
| DCK | ENSG00000156136 | 2,21 | 1,0E-05 |
| POLR2M | ENSG00000255529 | 2,21 | 1,2E-05 |
| ITGA6 | ENSG00000091409 | 2,21 | 9,1E-06 |
| INO80D | ENSG00000114933 | 2,21 | 4,6E-04 |
| ZFP1 | ENSG00000184517 | 2,21 | 1,1E-05 |
| HSPA13 | ENSG00000155304 | 2,21 | 9,5E-06 |
| NRIP1 | ENSG00000180530 | 2,21 | 3,9E-04 |
| MAP3K2 | ENSG00000169967 | 2,21 | 6,4E-06 |
| PKIB | ENSG00000135549 | 2,21 | 1,6E-06 |
| RYBP | ENSG00000163602 | 2,21 | 7,2E-06 |
| C11orf82 | ENSG00000165490 | 2,21 | 6,0E-05 |
| FBXL3 | ENSG00000005812 | 2,20 | 1,1E-05 |
| DTWD2 | ENSG00000169570 | 2,20 | 7,0E-04 |
| DIAPH3 | ENSG00000139734 | 2,20 | 5,9E-05 |
| POU2F1 | ENSG00000143190 | 2,20 | 8,3E-04 |
| CTGF | ENSG00000118523 | 2,20 | 3,3E-03 |
| SACM1L | ENSG00000211456 | 2,20 | 9,3E-06 |
| RBM43 | ENSG00000184898 | 2,20 | 1,2E-03 |
| B4GALT6 | ENSG00000118276 | 2,20 | 1,6E-05 |
| C14orf28 | ENSG00000179476 | 2,20 | 7,2E-05 |
| EEA1 | ENSG00000102189 | 2,20 | 3,9E-05 |
| ARNTL2 | ENSG00000029153 | 2,20 | 8,1E-06 |
| SMAD4 | ENSG00000141646 | 2,20 | 4,2E-06 |
| GRAMD1C | ENSG00000178075 | 2,20 | 1,9E-04 |
| YAF2 | ENSG00000015153 | 2,19 | 1,8E-05 |
| BTBD3 | ENSG00000132640 | 2,19 | 1,6E-05 |
| KRT222 | ENSG00000213424 | 2,19 | 2,6E-03 |
| PTER | ENSG00000165983 | 2,19 | 7,5E-06 |
| KIAA0232 | ENSG00000170871 | 2,19 | 1,1E-04 |
| ACER3 | ENSG00000078124 | 2,19 | 2,8E-06 |
| RP11-122G18.5 | ENSG00000235477 | 2,19 | 4,4E-03 |
| SMIM15 | ENSG00000188725 | 2,18 | 5,4E-05 |
| EPS15 | ENSG00000085832 | 2,18 | 6,2E-06 |
| THAP10 | ENSG00000129028 | 2,18 | 4,9E-03 |
| AKAP11 | ENSG00000023516 | 2,18 | 9,6E-06 |
| EGR3 | ENSG00000179388 | 2,18 | 1,3E-03 |
| CDCA7 | ENSG00000144354 | 2,18 | 2,1E-06 |
| MRI1 | ENSG00000037757 | 2,18 | 4,2E-04 |
| OSBPL8 | ENSG00000091039 | 2,18 | 1,6E-05 |
| CD109 | ENSG00000156535 | 2,18 | 9,2E-05 |
| COL4A3BP | ENSG00000113163 | 2,18 | 2,0E-05 |
| HMGN4 | ENSG00000182952 | 2,18 | 1,7E-06 |
| ACSL4 | ENSG00000068366 | 2,17 | 1,3E-06 |
| LRP6 | ENSG00000070018 | 2,17 | 1,5E-04 |
| AMD1 | ENSG00000123505 | 2,17 | 3,0E-06 |
| EML5 | ENSG00000165521 | 2,17 | 3,1E-04 |
| PPTC7 | ENSG00000196850 | 2,17 | 8,3E-06 |
| FAM178A | ENSG00000119906 | 2,17 | 2,7E-05 |
| API5 | ENSG00000166181 | 2,17 | 6,4E-06 |
| SFXN1 | ENSG00000164466 | 2,17 | 2,8E-06 |
| SLC25A24 | ENSG00000085491 | 2,16 | 1,0E-05 |
| SLC19A2 | ENSG00000117479 | 2,16 | 1,6E-05 |
| YES1 | ENSG00000176105 | 2,16 | 3,9E-06 |
| SMIM13 | ENSG00000224531 | 2,16 | 3,0E-06 |
| BZW1P2 | ENSG00000198406 | 2,16 | 1,3E-05 |
| PGM2L1 | ENSG00000165434 | 2,16 | 3,7E-03 |
| RP11-179H18.2 | ENSG00000233177 | 2,16 | 1,3E-04 |
| VEZF1 | ENSG00000136451 | 2,16 | 2,6E-06 |
| ARPP19 | ENSG00000128989 | 2,16 | 2,6E-06 |
| BIRC3 | ENSG00000023445 | 2,16 | 2,6E-04 |
| RAP2A | ENSG00000125249 | 2,16 | 1,6E-06 |
| C11orf54 | ENSG00000182919 | 2,16 | 1,6E-05 |
| ATP11B | ENSG00000058063 | 2,16 | 7,4E-06 |
| C5orf30 | ENSG00000181751 | 2,16 | 1,5E-05 |
| PIGK | ENSG00000142892 | 2,16 | 1,4E-05 |
| SLC35G2 | ENSG00000168917 | 2,15 | 3,2E-04 |
| PDP1 | ENSG00000164951 | 2,15 | 2,3E-06 |
| MELK | ENSG00000165304 | 2,15 | 1,3E-06 |
| GPD2 | ENSG00000115159 | 2,15 | 7,9E-06 |
| NADK2 | ENSG00000152620 | 2,15 | 6,4E-06 |
| C17orf104 | ENSG00000180336 | 2,15 | 1,2E-03 |
| HMGCR | ENSG00000113161 | 2,15 | 5,8E-06 |
| TMEM106B | ENSG00000106460 | 2,15 | 2,6E-05 |
| RP11-303E16.2 | ENSG00000261061 | 2,15 | 1,7E-04 |
| GPD1L | ENSG00000152642 | 2,15 | 3,9E-05 |
| RNF219 | ENSG00000152193 | 2,15 | 3,6E-05 |
| ATG14 | ENSG00000126775 | 2,15 | 1,9E-05 |
| DPY19L4 | ENSG00000156162 | 2,15 | 1,3E-05 |
| REPS2 | ENSG00000169891 | 2,15 | 4,7E-03 |
| GNAI1 | ENSG00000127955 | 2,15 | 2,6E-06 |
| METTL9 | ENSG00000197006 | 2,14 | 1,7E-06 |
| SEL1L | ENSG00000071537 | 2,14 | 4,0E-05 |
| CPEB2 | ENSG00000137449 | 2,14 | 1,9E-03 |
| RP3-512B11.3 | ENSG00000261189 | 2,14 | 2,8E-03 |
| AASDHPPT | ENSG00000149313 | 2,14 | 7,3E-06 |
| VHL | ENSG00000134086 | 2,14 | 3,0E-04 |
| PTEN | ENSG00000171862 | 2,14 | 3,5E-06 |
| MAPK1 | ENSG00000100030 | 2,14 | 2,3E-05 |
| HIPK3 | ENSG00000110422 | 2,14 | 1,3E-05 |
| DEPDC1 | ENSG00000024526 | 2,14 | 1,6E-06 |
| DENND4C | ENSG00000137145 | 2,14 | 4,0E-05 |
| RAB8B | ENSG00000166128 | 2,14 | 5,3E-06 |
| PCMTD1 | ENSG00000168300 | 2,14 | 8,9E-04 |
| ERO1LB | ENSG00000086619 | 2,14 | 1,1E-04 |
| RNF170 | ENSG00000120925 | 2,13 | 1,2E-05 |
| UBE2D1 | ENSG00000072401 | 2,13 | 6,9E-05 |
| EMB | ENSG00000170571 | 2,13 | 6,2E-05 |
| CMPK1 | ENSG00000162368 | 2,13 | 9,4E-07 |
| RFX7 | ENSG00000181827 | 2,13 | 1,4E-05 |
| CHML | ENSG00000203668 | 2,13 | 1,5E-05 |
| RP11-456J20.1 | ENSG00000259358 | 2,13 | 2,6E-03 |
| ITGAV | ENSG00000138448 | 2,13 | 3,5E-05 |
| SKAP2 | ENSG00000005020 | 2,13 | 8,7E-06 |
| C14orf142 | ENSG00000170270 | 2,13 | 8,2E-05 |
| AGPS | ENSG00000018510 | 2,13 | 8,2E-06 |
| AP1S2 | ENSG00000182287 | 2,13 | 1,3E-05 |
| REEP1 | ENSG00000068615 | 2,12 | 6,8E-05 |
| PRKAR1A | ENSG00000108946 | 2,12 | 1,2E-05 |
| MZT1 | ENSG00000204899 | 2,12 | 4,8E-06 |
| RAB2B | ENSG00000129472 | 2,12 | 1,0E-05 |
| FNDC3A | ENSG00000102531 | 2,12 | 9,9E-06 |
| ATL3 | ENSG00000184743 | 2,12 | 1,4E-05 |
| PGAP1 | ENSG00000197121 | 2,12 | 3,4E-04 |
| ERI2 | ENSG00000196678 | 2,12 | 2,5E-05 |
| RB1 | ENSG00000139687 | 2,12 | 4,9E-05 |
| SLC35F5 | ENSG00000115084 | 2,12 | 7,1E-05 |
| XPO4 | ENSG00000132953 | 2,12 | 2,2E-05 |
| SLC16A6 | ENSG00000108932 | 2,12 | 1,0E-03 |
| LRRC8C | ENSG00000171488 | 2,12 | 4,3E-05 |
| ALG10B | ENSG00000175548 | 2,12 | 1,8E-03 |
| IRS2 | ENSG00000185950 | 2,11 | 3,6E-03 |
| CAND1 | ENSG00000111530 | 2,11 | 4,5E-06 |
| EOGT | ENSG00000163378 | 2,11 | 1,0E-04 |
| SEH1L | ENSG00000085415 | 2,11 | 5,1E-06 |
| TMTC3 | ENSG00000139324 | 2,11 | 2,1E-05 |
| CNOT6 | ENSG00000113300 | 2,11 | 7,7E-06 |
| TRIQK | ENSG00000205133 | 2,11 | 1,3E-04 |
| FAM20B | ENSG00000116199 | 2,11 | 4,2E-05 |
| MCFD2 | ENSG00000180398 | 2,11 | 5,9E-06 |
| FRMD6 | ENSG00000139926 | 2,10 | 1,5E-04 |
| PIK3AP1 | ENSG00000155629 | 2,10 | 3,5E-04 |
| IBTK | ENSG00000005700 | 2,10 | 1,7E-05 |
| STYX | ENSG00000198252 | 2,10 | 7,6E-06 |
| MTM1 | ENSG00000171100 | 2,10 | 2,7E-03 |
| ASNSD1 | ENSG00000138381 | 2,10 | 1,5E-05 |
| FAM35A | ENSG00000122376 | 2,10 | 5,8E-06 |
| CYLD | ENSG00000083799 | 2,10 | 5,0E-05 |
| AEBP2 | ENSG00000139154 | 2,10 | 1,4E-05 |
| FYTTD1 | ENSG00000122068 | 2,10 | 3,8E-06 |
| UBXN2A | ENSG00000173960 | 2,10 | 3,3E-05 |
| USP1 | ENSG00000162607 | 2,10 | 4,6E-05 |
| RP11-566E18.3 | ENSG00000260804 | 2,09 | 4,1E-03 |
| MBNL2 | ENSG00000139793 | 2,09 | 4,4E-06 |
| PRPS2 | ENSG00000101911 | 2,09 | 2,2E-06 |
| SLC30A6 | ENSG00000152683 | 2,09 | 4,1E-05 |
| C6orf57 | ENSG00000154079 | 2,09 | 1,7E-05 |
| PI4K2B | ENSG00000038210 | 2,09 | 4,2E-06 |
| LIG4 | ENSG00000174405 | 2,09 | 1,1E-04 |
| RAB6A | ENSG00000175582 | 2,09 | 3,4E-06 |
| CMTM6 | ENSG00000091317 | 2,09 | 1,4E-05 |
| LSM11 | ENSG00000155858 | 2,09 | 1,4E-05 |
| TBC1D12 | ENSG00000108239 | 2,09 | 2,7E-04 |
| TC2N | ENSG00000165929 | 2,09 | 1,8E-05 |
| RPRD1A | ENSG00000141425 | 2,08 | 6,1E-06 |
| LYRM7 | ENSG00000186687 | 2,08 | 1,3E-05 |
| RPS6KB1 | ENSG00000108443 | 2,08 | 5,1E-06 |
| KATNBL1 | ENSG00000134152 | 2,08 | 6,6E-05 |
| CREBRF | ENSG00000164463 | 2,08 | 2,7E-03 |
| BZW1P1 | ENSG00000236686 | 2,08 | 4,0E-03 |
| ABCC4 | ENSG00000125257 | 2,08 | 2,5E-05 |
| SLC35A5 | ENSG00000138459 | 2,08 | 8,4E-06 |
| SNX14 | ENSG00000135317 | 2,08 | 5,9E-06 |
| ATP7A | ENSG00000165240 | 2,08 | 9,7E-04 |
| PDLIM5 | ENSG00000163110 | 2,08 | 2,4E-06 |
| TMEM67 | ENSG00000164953 | 2,08 | 1,4E-05 |
| ZC3H12C | ENSG00000149289 | 2,08 | 5,3E-05 |
| DDX46 | ENSG00000145833 | 2,08 | 5,7E-05 |
| SRP72 | ENSG00000174780 | 2,08 | 1,2E-06 |
| SPTLC1 | ENSG00000090054 | 2,08 | 1,3E-05 |
| GNAQ | ENSG00000156052 | 2,08 | 3,2E-05 |
| GSR | ENSG00000104687 | 2,07 | 2,3E-06 |
| ARRDC3 | ENSG00000113369 | 2,07 | 2,2E-05 |
| FSD1L | ENSG00000106701 | 2,07 | 8,8E-05 |
| RP11-56B16.3.1 | ENSG00000259748 | 2,07 | 6,4E-04 |
| UBA6 | ENSG00000033178 | 2,07 | 8,4E-06 |
| SOS2 | ENSG00000100485 | 2,07 | 8,0E-05 |
| HACL1 | ENSG00000131373 | 2,07 | 2,6E-04 |
| HDAC9 | ENSG00000048052 | 2,07 | 1,6E-04 |
| KIAA0895 | ENSG00000164542 | 2,07 | 5,0E-05 |
| TM9SF3 | ENSG00000077147 | 2,07 | 2,3E-06 |
| FBXO30 | ENSG00000118496 | 2,07 | 2,1E-05 |
| TWF1 | ENSG00000151239 | 2,07 | 1,9E-05 |
| NUP133 | ENSG00000069248 | 2,07 | 1,9E-05 |
| RBM41 | ENSG00000089682 | 2,07 | 4,9E-03 |
| OXR1 | ENSG00000164830 | 2,07 | 1,2E-05 |
| HS6ST2 | ENSG00000171004 | 2,07 | 1,0E-04 |
| EIF4E | ENSG00000151247 | 2,07 | 1,5E-05 |
| PTBP3 | ENSG00000119314 | 2,07 | 2,4E-05 |
| PAWR | ENSG00000177425 | 2,06 | 8,9E-06 |
| LATS1 | ENSG00000131023 | 2,06 | 2,5E-05 |
| PARP11 | ENSG00000111224 | 2,06 | 2,6E-03 |
| ESCO1 | ENSG00000141446 | 2,06 | 9,0E-05 |
| GAN | ENSG00000261609 | 2,06 | 7,4E-05 |
| TCF12 | ENSG00000140262 | 2,06 | 1,4E-05 |
| RIC8B | ENSG00000111785 | 2,06 | 1,2E-04 |
| RBBP9 | ENSG00000089050 | 2,06 | 9,5E-05 |
| G2E3 | ENSG00000092140 | 2,06 | 6,9E-06 |
| USP53 | ENSG00000145390 | 2,06 | 1,7E-05 |
| BZW1 | ENSG00000082153 | 2,06 | 2,7E-06 |
| PRKAR2B | ENSG00000005249 | 2,06 | 5,1E-05 |
| SHOC2 | ENSG00000108061 | 2,06 | 2,9E-05 |
| ATE1 | ENSG00000107669 | 2,06 | 1,1E-05 |
| ZFYVE16 | ENSG00000039319 | 2,06 | 4,6E-05 |
| SLC25A46 | ENSG00000164209 | 2,06 | 3,4E-05 |
| SEC24A | ENSG00000113615 | 2,06 | 1,8E-05 |
| PPP3R1 | ENSG00000221823 | 2,06 | 5,9E-06 |
| ALG9 | ENSG00000086848 | 2,06 | 2,2E-03 |
| SYPL1 | ENSG00000008282 | 2,06 | 5,1E-06 |
| HIATL1 | ENSG00000148110 | 2,06 | 1,2E-05 |
| CXorf56 | ENSG00000018610 | 2,05 | 9,7E-05 |
| DCAF16 | ENSG00000163257 | 2,05 | 3,5E-05 |
| FBXL5 | ENSG00000118564 | 2,05 | 4,9E-06 |
| RBMS1 | ENSG00000153250 | 2,05 | 2,9E-05 |
| VPS26A | ENSG00000122958 | 2,05 | 9,5E-06 |
| ARHGAP42 | ENSG00000165895 | 2,05 | 2,9E-04 |
| STT3B | ENSG00000163527 | 2,05 | 2,3E-06 |
| ATF1 | ENSG00000123268 | 2,05 | 3,8E-05 |
| ZNF148 | ENSG00000163848 | 2,05 | 1,4E-05 |
| XRN1 | ENSG00000114127 | 2,05 | 1,7E-04 |
| ETF1 | ENSG00000120705 | 2,05 | 5,1E-06 |
| NRAS | ENSG00000213281 | 2,05 | 1,0E-05 |
| SLC5A3 | ENSG00000198743 | 2,05 | 2,1E-04 |
| KLF3 | ENSG00000109787 | 2,04 | 1,1E-05 |
| JAZF1 | ENSG00000153814 | 2,04 | 3,4E-04 |
| TGFBR2 | ENSG00000163513 | 2,04 | 2,6E-05 |
| TMEM9B | ENSG00000175348 | 2,04 | 1,7E-05 |
| RPS6KA5 | ENSG00000100784 | 2,04 | 2,9E-04 |
| ST3GAL5 | ENSG00000115525 | 2,04 | 1,8E-03 |
| RP11-307L3.2 | ENSG00000233846 | 2,04 | 2,7E-04 |
| CDC14B | ENSG00000081377 | 2,04 | 2,3E-05 |
| VAMP7 | ENSG00000124333 | 2,04 | 1,2E-05 |
| GCLM | ENSG00000023909 | 2,04 | 1,6E-04 |
| CMTR2 | ENSG00000180917 | 2,04 | 5,5E-05 |
| RP11-473I1.5 | ENSG00000260349 | 2,04 | 1,3E-03 |
| DSG2 | ENSG00000046604 | 2,04 | 1,7E-05 |
| SAMD8 | ENSG00000156671 | 2,04 | 3,6E-05 |
| CTBS | ENSG00000117151 | 2,04 | 2,7E-05 |
| PPAPDC2 | ENSG00000205808 | 2,04 | 1,7E-03 |
| RNGTT | ENSG00000111880 | 2,04 | 1,3E-05 |
| ASAP1 | ENSG00000153317 | 2,04 | 7,3E-05 |
| MPZL2 | ENSG00000149573 | 2,03 | 1,8E-04 |
| HIF1A | ENSG00000100644 | 2,03 | 1,5E-05 |
| LARP4 | ENSG00000161813 | 2,03 | 7,2E-06 |
| DCUN1D3 | ENSG00000188215 | 2,03 | 3,8E-04 |
| ZNF518A | ENSG00000177853 | 2,03 | 8,5E-05 |
| WDR41 | ENSG00000164253 | 2,03 | 6,2E-06 |
| IPP | ENSG00000197429 | 2,03 | 1,1E-03 |
| TAF9B | ENSG00000187325 | 2,02 | 2,7E-05 |
| MICU2 | ENSG00000165487 | 2,02 | 1,6E-04 |
| DLGAP5 | ENSG00000126787 | 2,02 | 1,5E-05 |
| SHOX2 | ENSG00000168779 | 2,02 | 6,1E-04 |
| TMEM168 | ENSG00000146802 | 2,02 | 2,3E-05 |
| LRRC8B | ENSG00000197147 | 2,02 | 5,2E-05 |
| RRM1 | ENSG00000167325 | 2,02 | 1,7E-05 |
| MTMR2 | ENSG00000087053 | 2,02 | 3,4E-05 |
| TBCK | ENSG00000145348 | 2,02 | 2,5E-04 |
| MFAP3 | ENSG00000037749 | 2,01 | 2,9E-05 |
| RC3H1 | ENSG00000135870 | 2,01 | 7,6E-05 |
| BTBD7 | ENSG00000011114 | 2,01 | 3,3E-05 |
| PIGA | ENSG00000165195 | 2,01 | 4,1E-05 |
| SH3BGRL2 | ENSG00000198478 | 2,01 | 1,1E-04 |
| TBC1D8B | ENSG00000133138 | 2,01 | 5,5E-04 |
| TAP2 | ENSG00000204267 | 2,01 | 3,5E-04 |
| LMBRD2 | ENSG00000164187 | 2,01 | 2,8E-03 |
| ELL2 | ENSG00000118985 | 2,01 | 1,1E-05 |
| KDELC2 | ENSG00000178202 | 2,01 | 4,1E-05 |
| NSF | ENSG00000073969 | 2,01 | 1,5E-05 |
| DDAH1 | ENSG00000153904 | 2,01 | 2,3E-05 |
| AMMECR1 | ENSG00000101935 | 2,01 | 4,5E-05 |
| MAN1A1 | ENSG00000111885 | 2,01 | 5,0E-05 |
| G3BP2 | ENSG00000138757 | 2,01 | 1,4E-05 |
| CSNK1G3 | ENSG00000151292 | 2,01 | 1,4E-05 |
| SNX24 | ENSG00000064652 | 2,01 | 7,2E-05 |
| FKBP14 | ENSG00000106080 | 2,01 | 4,6E-05 |
| NDUFC2 | ENSG00000151366 | 2,01 | 7,9E-05 |
| LEMD3 | ENSG00000174106 | 2,01 | 3,8E-05 |
| PRKACB | ENSG00000142875 | 2,01 | 6,0E-06 |
| LAMP2 | ENSG00000005893 | 2,01 | 1,4E-05 |
| TMX1 | ENSG00000139921 | 2,01 | 2,1E-05 |
| RNF182 | ENSG00000180537 | 2,01 | 3,9E-05 |
| BRI3BP | ENSG00000184992 | 2,01 | 1,0E-05 |
| IKZF5 | ENSG00000095574 | 2,00 | 2,9E-05 |
| ANO6 | ENSG00000177119 | 2,00 | 3,1E-05 |
| C16orf52 | ENSG00000185716 | 2,00 | 3,7E-05 |
| MFN1 | ENSG00000171109 | 2,00 | 1,6E-05 |
| TEAD1 | ENSG00000187079 | 2,00 | 1,6E-04 |
| FRS2 | ENSG00000166225 | 2,00 | 7,9E-05 |
| RASSF8 | ENSG00000123094 | 2,00 | 5,7E-04 |
| RP11-488L18.10 | ENSG00000259865 | 2,00 | 1,4E-03 |
| TRPC1 | ENSG00000144935 | 2,00 | 9,6E-04 |
| USP46 | ENSG00000109189 | 2,00 | 1,7E-05 |
| FASTKD2 | ENSG00000118246 | 2,00 | 1,1E-04 |
| GINS1 | ENSG00000101003 | 2,00 | 3,0E-05 |
| THNSL1 | ENSG00000185875 | 2,00 | 2,6E-04 |
| PARD6B | ENSG00000124171 | 2,00 | 1,8E-04 |
| WDR36 | ENSG00000134987 | 2,00 | 3,1E-05 |
| CBX5 | ENSG00000094916 | 2,00 | 3,0E-04 |
| SVIP | ENSG00000198168 | 2,00 | 6,3E-05 |
| ZZZ3 | ENSG00000036549 | 2,00 | 2,6E-05 |
| WDFY1 | ENSG00000085449 | 2,00 | 1,2E-05 |
| CCNI | ENSG00000118816 | 1,99 | 3,0E-05 |
| XPO1 | ENSG00000082898 | 1,99 | 2,3E-06 |
| AP1S3 | ENSG00000152056 | 1,99 | 8,5E-05 |
| SIX4 | ENSG00000100625 | 1,99 | 2,4E-05 |
| TRAF3IP3 | ENSG00000009790 | 1,99 | 7,4E-04 |
| CAB39L | ENSG00000102547 | 1,99 | 1,7E-03 |
| UFSP2 | ENSG00000109775 | 1,99 | 1,8E-04 |
| FAM126A | ENSG00000122591 | 1,99 | 9,5E-05 |
| GABPA | ENSG00000154727 | 1,99 | 3,0E-05 |
| COPS8 | ENSG00000198612 | 1,99 | 2,9E-05 |
| CCDC125 | ENSG00000183323 | 1,99 | 5,4E-05 |
| ZNF808 | ENSG00000198482 | 1,99 | 3,2E-03 |
| GPNMB | ENSG00000136235 | 1,99 | 6,6E-04 |
| MMADHC | ENSG00000168288 | 1,99 | 6,5E-05 |
| DHX15 | ENSG00000109606 | 1,99 | 3,2E-06 |
| CYB561D1 | ENSG00000174151 | 1,99 | 1,2E-03 |
| TEX10 | ENSG00000136891 | 1,99 | 1,3E-05 |
| MDM2 | ENSG00000135679 | 1,98 | 5,4E-05 |
| CASP3 | ENSG00000164305 | 1,98 | 4,4E-04 |
| FAM208B | ENSG00000108021 | 1,98 | 1,0E-05 |
| ASAH2B | ENSG00000204147 | 1,98 | 8,5E-05 |
| TAF1A | ENSG00000143498 | 1,98 | 4,5E-05 |
| UHRF1BP1L | ENSG00000111647 | 1,98 | 4,0E-05 |
| EFR3A | ENSG00000132294 | 1,98 | 2,1E-05 |
| ZRANB2 | ENSG00000132485 | 1,98 | 1,7E-05 |
| ZNF25 | ENSG00000175395 | 1,98 | 3,6E-04 |
| CCNC | ENSG00000112237 | 1,98 | 5,1E-06 |
| PAPD5 | ENSG00000121274 | 1,98 | 4,9E-05 |
| MYNN | ENSG00000085274 | 1,98 | 1,3E-04 |
| BBX | ENSG00000114439 | 1,98 | 7,4E-05 |
| RSL24D1 | ENSG00000137876 | 1,98 | 2,3E-05 |
| B3GALNT1 | ENSG00000169255 | 1,98 | 5,0E-05 |
| LIPA | ENSG00000107798 | 1,97 | 1,7E-05 |
| RP3-523K23.2 | ENSG00000261116 | 1,97 | 5,6E-04 |
| TRIM36 | ENSG00000152503 | 1,97 | 1,1E-04 |
| IFT52 | ENSG00000101052 | 1,97 | 1,4E-05 |
| CASC2 | ENSG00000177640 | 1,97 | 1,3E-03 |
| ONECUT2 | ENSG00000119547 | 1,97 | 2,3E-03 |
| RP4-604K5.1 | ENSG00000183291 | 1,97 | 2,6E-05 |
| TMEM181 | ENSG00000146433 | 1,97 | 7,9E-05 |
| USP14 | ENSG00000101557 | 1,97 | 1,8E-05 |
| JADE3 | ENSG00000102221 | 1,97 | 5,1E-05 |
| BMPR2 | ENSG00000204217 | 1,97 | 1,0E-04 |
| JMY | ENSG00000152409 | 1,97 | 1,8E-04 |
| ST6GALNAC5 | ENSG00000117069 | 1,97 | 4,1E-03 |
| NUBPL | ENSG00000151413 | 1,97 | 2,5E-03 |
| TTC33 | ENSG00000113638 | 1,97 | 4,1E-05 |
| ASPHD2 | ENSG00000128203 | 1,96 | 3,3E-03 |
| CSE1L | ENSG00000124207 | 1,96 | 1,0E-05 |
| ATF2 | ENSG00000115966 | 1,96 | 1,6E-05 |
| ZNF680 | ENSG00000173041 | 1,96 | 2,3E-04 |
| SMC5 | ENSG00000198887 | 1,96 | 3,1E-05 |
| EPB41 | ENSG00000159023 | 1,96 | 3,7E-03 |
| RP11-166D19.1 | ENSG00000255248 | 1,96 | 6,3E-06 |
| TXNDC9 | ENSG00000115514 | 1,96 | 3,9E-04 |
| NUP43 | ENSG00000120253 | 1,96 | 1,7E-05 |
| HINT3 | ENSG00000111911 | 1,96 | 1,8E-05 |
| G3BP1 | ENSG00000145907 | 1,96 | 9,1E-06 |
| PRKAB2 | ENSG00000131791 | 1,96 | 1,9E-04 |
| LPGAT1 | ENSG00000123684 | 1,95 | 2,0E-05 |
| DBT | ENSG00000137992 | 1,95 | 8,6E-05 |
| MBTD1 | ENSG00000011258 | 1,95 | 4,4E-05 |
| CCPG1 | ENSG00000260916 | 1,95 | 3,8E-03 |
| FAM63B | ENSG00000128923 | 1,95 | 1,3E-04 |
| IL15 | ENSG00000164136 | 1,95 | 1,3E-03 |
| SLAIN2 | ENSG00000109171 | 1,95 | 4,8E-05 |
| B3GALNT2 | ENSG00000162885 | 1,95 | 2,9E-05 |
| NEDD4 | ENSG00000069869 | 1,95 | 9,5E-05 |
| C10orf32 | ENSG00000166275 | 1,95 | 5,1E-04 |
| C6orf62 | ENSG00000112308 | 1,95 | 9,9E-06 |
| C3orf58 | ENSG00000181744 | 1,95 | 4,9E-04 |
| USP33 | ENSG00000077254 | 1,95 | 6,4E-05 |
| SUCLG2 | ENSG00000172340 | 1,95 | 7,7E-05 |
| USP45 | ENSG00000123552 | 1,95 | 8,5E-05 |
| PRPSAP2 | ENSG00000141127 | 1,95 | 9,4E-05 |
| PAQR5 | ENSG00000137819 | 1,94 | 7,3E-04 |
| POC1B | ENSG00000139323 | 1,94 | 3,6E-05 |
| CEBPZ-AS1 | ENSG00000218739 | 1,94 | 1,0E-05 |
| STEAP2 | ENSG00000157214 | 1,94 | 1,6E-04 |
| ABHD10 | ENSG00000144827 | 1,94 | 1,3E-05 |
| SOWAHC | ENSG00000198142 | 1,94 | 1,1E-04 |
| THAP6 | ENSG00000174796 | 1,94 | 8,1E-05 |
| RC3H2 | ENSG00000056586 | 1,94 | 6,8E-05 |
| LRR1 | ENSG00000165501 | 1,94 | 1,7E-04 |
| GMCL1 | ENSG00000087338 | 1,94 | 1,5E-05 |
| PPP2R5A | ENSG00000066027 | 1,94 | 1,5E-05 |
| IRAK1BP1 | ENSG00000146243 | 1,94 | 6,8E-04 |
| WRB | ENSG00000182093 | 1,94 | 2,7E-05 |
| SLC36A4 | ENSG00000180773 | 1,94 | 4,8E-05 |
| VPS54 | ENSG00000143952 | 1,94 | 1,1E-04 |
| ZDHHC2 | ENSG00000104219 | 1,94 | 1,2E-05 |
| TGFBR3 | ENSG00000069702 | 1,94 | 1,8E-03 |
| NUFIP2 | ENSG00000108256 | 1,93 | 4,7E-05 |
| COBLL1 | ENSG00000082438 | 1,93 | 2,7E-04 |
| DCAF17 | ENSG00000115827 | 1,93 | 2,3E-05 |
| MDM4 | ENSG00000198625 | 1,93 | 3,5E-05 |
| PCGF5 | ENSG00000180628 | 1,93 | 1,1E-05 |
| PPM1A | ENSG00000100614 | 1,93 | 1,8E-05 |
| ARFIP1 | ENSG00000164144 | 1,93 | 9,4E-05 |
| KIAA1524 | ENSG00000163507 | 1,93 | 3,1E-04 |
| PLAGL1 | ENSG00000118495 | 1,93 | 3,8E-05 |
| PLEKHA1 | ENSG00000107679 | 1,93 | 3,7E-05 |
| CAPZA1 | ENSG00000116489 | 1,93 | 1,4E-05 |
| MAD2L1 | ENSG00000164109 | 1,93 | 1,4E-04 |
| GAREM | ENSG00000141441 | 1,92 | 1,2E-03 |
| BTC | ENSG00000174808 | 1,92 | 3,6E-03 |
| KLF4 | ENSG00000136826 | 1,92 | 1,1E-04 |
| PWWP2A | ENSG00000170234 | 1,92 | 5,4E-04 |
| COG5 | ENSG00000164597 | 1,92 | 4,2E-04 |
| NDUFB5 | ENSG00000136521 | 1,92 | 3,3E-05 |
| DRAM2 | ENSG00000156171 | 1,92 | 3,5E-05 |
| ANKRD46 | ENSG00000186106 | 1,92 | 8,2E-05 |
| FAM76B | ENSG00000077458 | 1,92 | 1,3E-04 |
| MTMR10 | ENSG00000166912 | 1,92 | 2,4E-04 |
| ENPP1 | ENSG00000197594 | 1,92 | 1,4E-03 |
| SLC46A3 | ENSG00000139508 | 1,92 | 5,6E-03 |
| MLTK | ENSG00000091436 | 1,92 | 6,3E-05 |
| C10orf88 | ENSG00000119965 | 1,92 | 7,2E-05 |
| RNF141 | ENSG00000110315 | 1,92 | 2,1E-05 |
| TMEM87A | ENSG00000103978 | 1,92 | 2,1E-04 |
| CAB39 | ENSG00000135932 | 1,92 | 1,8E-05 |
| DEK | ENSG00000124795 | 1,92 | 3,0E-05 |
| PCNXL4 | ENSG00000126773 | 1,92 | 2,1E-05 |
| SGTB | ENSG00000197860 | 1,92 | 8,5E-05 |
| MSL2 | ENSG00000174579 | 1,91 | 1,3E-04 |
| GNAI3 | ENSG00000065135 | 1,91 | 4,9E-05 |
| SLC30A7 | ENSG00000162695 | 1,91 | 1,7E-04 |
| SIX1 | ENSG00000126778 | 1,91 | 1,9E-03 |
| KCTD18 | ENSG00000155729 | 1,91 | 9,2E-05 |
| HIAT1 | ENSG00000156875 | 1,91 | 4,6E-05 |
| WWP1 | ENSG00000123124 | 1,91 | 1,1E-05 |
| PLA2G4A | ENSG00000116711 | 1,91 | 6,6E-04 |
| DUSP19 | ENSG00000162999 | 1,91 | 2,5E-03 |
| FEM1C | ENSG00000145780 | 1,91 | 1,1E-04 |
| QSER1 | ENSG00000060749 | 1,91 | 2,3E-05 |
| PAPOLA | ENSG00000090060 | 1,91 | 2,9E-05 |
| SEMA3C | ENSG00000075223 | 1,91 | 5,6E-03 |
| THAP1 | ENSG00000131931 | 1,91 | 8,7E-04 |
| MYSM1 | ENSG00000162601 | 1,91 | 8,7E-05 |
| EXOC5P1 | ENSG00000180673 | 1,91 | 3,0E-03 |
| PPP4R2 | ENSG00000163605 | 1,91 | 4,9E-05 |
| GPM6A | ENSG00000150625 | 1,91 | 3,6E-03 |
| GOPC | ENSG00000047932 | 1,91 | 5,1E-05 |
| SOAT1 | ENSG00000057252 | 1,91 | 2,9E-04 |
| TIPARP | ENSG00000163659 | 1,91 | 3,0E-04 |
| LRRC40 | ENSG00000066557 | 1,91 | 1,4E-04 |
| ASXL2 | ENSG00000143970 | 1,91 | 2,1E-04 |
| FBXL17 | ENSG00000145743 | 1,91 | 5,3E-04 |
| FNIP2 | ENSG00000052795 | 1,91 | 2,9E-04 |
| TBCEL | ENSG00000154114 | 1,90 | 1,0E-04 |
| CDC27 | ENSG00000004897 | 1,90 | 2,1E-05 |
| C18orf54 | ENSG00000166845 | 1,90 | 8,4E-05 |
| DLG1 | ENSG00000075711 | 1,90 | 8,0E-05 |
| SDCBPP3 | ENSG00000232801 | 1,90 | 5,8E-03 |
| RFX3 | ENSG00000080298 | 1,90 | 1,3E-03 |
| PARD6G | ENSG00000178184 | 1,90 | 1,7E-04 |
| PICALM | ENSG00000073921 | 1,90 | 4,3E-05 |
| BIVM | ENSG00000134897 | 1,89 | 2,8E-04 |
| MAL2 | ENSG00000147676 | 1,89 | 6,5E-06 |
| FNDC3B | ENSG00000075420 | 1,89 | 3,8E-05 |
| RLF | ENSG00000117000 | 1,89 | 4,0E-04 |
| SPIN4 | ENSG00000186767 | 1,89 | 2,1E-04 |
| PTP4A1 | ENSG00000112245 | 1,89 | 3,5E-05 |
| CACUL1 | ENSG00000151893 | 1,89 | 2,6E-05 |
| BROX | ENSG00000162819 | 1,89 | 5,7E-05 |
| PPP2R5C | ENSG00000078304 | 1,89 | 7,9E-06 |
| PHF6 | ENSG00000156531 | 1,89 | 1,1E-05 |
| DYRK2 | ENSG00000127334 | 1,89 | 2,3E-05 |
| MTFR1 | ENSG00000066855 | 1,89 | 2,1E-05 |
| VCPIP1 | ENSG00000175073 | 1,89 | 1,0E-04 |
| RP5-1085F17.3 | ENSG00000260257 | 1,89 | 5,7E-04 |
| MAP2K4 | ENSG00000065559 | 1,89 | 6,1E-05 |
| FBXO9 | ENSG00000112146 | 1,89 | 2,1E-05 |
| TMED7 | ENSG00000134970 | 1,89 | 2,8E-04 |
| LIN9 | ENSG00000183814 | 1,89 | 5,9E-05 |
| SLC18B1 | ENSG00000146409 | 1,89 | 2,5E-04 |
| ADAM9 | ENSG00000168615 | 1,89 | 6,2E-06 |
| GPR137C | ENSG00000180998 | 1,89 | 6,3E-03 |
| ARL8B | ENSG00000134108 | 1,89 | 9,1E-05 |
| APBB2 | ENSG00000163697 | 1,88 | 3,3E-05 |
| MKX | ENSG00000150051 | 1,88 | 2,8E-05 |
| KAT2B | ENSG00000114166 | 1,88 | 6,9E-04 |
| RNF111 | ENSG00000157450 | 1,88 | 1,0E-04 |
| FCF1 | ENSG00000119616 | 1,88 | 1,6E-05 |
| KATNAL1 | ENSG00000102781 | 1,88 | 5,5E-05 |
| SENP1 | ENSG00000079387 | 1,88 | 2,0E-04 |
| PSD3 | ENSG00000156011 | 1,88 | 1,0E-03 |
| GNPDA2 | ENSG00000163281 | 1,88 | 4,5E-05 |
| SRSF1 | ENSG00000136450 | 1,88 | 9,6E-05 |
| DIP2B | ENSG00000066084 | 1,88 | 8,8E-05 |
| SNX4 | ENSG00000114520 | 1,88 | 7,3E-05 |
| CFL2 | ENSG00000165410 | 1,88 | 1,8E-05 |
| ARPC5 | ENSG00000162704 | 1,88 | 4,7E-05 |
| USP6NL | ENSG00000148429 | 1,88 | 2,9E-05 |
| ATG5 | ENSG00000057663 | 1,88 | 1,5E-04 |
| TNKS2 | ENSG00000107854 | 1,88 | 3,2E-04 |
| CAMK4 | ENSG00000152495 | 1,88 | 5,8E-05 |
| ELOVL5 | ENSG00000012660 | 1,87 | 2,3E-05 |
| AP1AR | ENSG00000138660 | 1,87 | 4,5E-05 |
| CEP97 | ENSG00000182504 | 1,87 | 4,2E-04 |
| ZNF28 | ENSG00000198538 | 1,87 | 1,8E-04 |
| ZNF674 | ENSG00000251192 | 1,87 | 4,4E-03 |
| NHLRC2 | ENSG00000196865 | 1,87 | 6,4E-05 |
| C5orf22 | ENSG00000082213 | 1,87 | 1,2E-04 |
| SPDYE3 | ENSG00000214300 | 1,87 | 2,9E-03 |
| ERBB2IP | ENSG00000112851 | 1,87 | 9,0E-05 |
| ARL6IP6 | ENSG00000177917 | 1,87 | 4,5E-05 |
| KIAA1143 | ENSG00000163807 | 1,87 | 4,3E-05 |
| NDUFA5 | ENSG00000128609 | 1,87 | 3,6E-04 |
| PCNP | ENSG00000081154 | 1,87 | 1,5E-05 |
| FLVCR1 | ENSG00000162769 | 1,87 | 4,9E-05 |
| DDHD2 | ENSG00000085788 | 1,86 | 5,4E-05 |
| ATP2B1 | ENSG00000070961 | 1,86 | 4,5E-05 |
| SEPSECS | ENSG00000109618 | 1,86 | 1,5E-04 |
| FBXO3 | ENSG00000110429 | 1,86 | 4,8E-05 |
| STAM | ENSG00000136738 | 1,86 | 3,7E-05 |
| TFPI | ENSG00000003436 | 1,86 | 4,8E-04 |
| ANP32E | ENSG00000143401 | 1,86 | 6,1E-05 |
| LRPPRC | ENSG00000138095 | 1,86 | 3,0E-05 |
| ZNF507 | ENSG00000168813 | 1,86 | 6,9E-05 |
| CHUK | ENSG00000213341 | 1,86 | 2,9E-05 |
| RALGPS2 | ENSG00000116191 | 1,86 | 1,2E-03 |
| NSUN3 | ENSG00000178694 | 1,85 | 1,9E-03 |
| SSX2IP | ENSG00000117155 | 1,85 | 2,3E-05 |
| SETD7 | ENSG00000145391 | 1,85 | 1,1E-04 |
| RAB7L1 | ENSG00000117280 | 1,85 | 1,3E-04 |
| PEX7 | ENSG00000112357 | 1,85 | 4,8E-04 |
| YIPF5 | ENSG00000145817 | 1,85 | 3,1E-05 |
| HNRNPLL | ENSG00000143889 | 1,85 | 3,6E-05 |
| ABI1 | ENSG00000136754 | 1,85 | 4,0E-05 |
| RRAGC | ENSG00000116954 | 1,85 | 5,1E-05 |
| ANKRD42 | ENSG00000137494 | 1,85 | 6,1E-04 |
| MYCBP | ENSG00000214114 | 1,85 | 6,1E-05 |
| FOXO3 | ENSG00000118689 | 1,85 | 3,5E-04 |
| ZNF566 | ENSG00000186017 | 1,85 | 1,3E-03 |
| RAVER2 | ENSG00000162437 | 1,85 | 2,9E-05 |
| ZNF697 | ENSG00000143067 | 1,85 | 2,0E-03 |
| UGT8 | ENSG00000174607 | 1,85 | 7,5E-05 |
| ATP10D | ENSG00000145246 | 1,85 | 4,5E-03 |
| ARHGEF12 | ENSG00000196914 | 1,85 | 6,0E-05 |
| MPP7 | ENSG00000150054 | 1,84 | 1,2E-05 |
| TMEM123 | ENSG00000152558 | 1,84 | 3,2E-05 |
| ZNF22 | ENSG00000165512 | 1,84 | 1,4E-04 |
| SPATA6 | ENSG00000132122 | 1,84 | 4,1E-03 |
| ZNF587 | ENSG00000198466 | 1,84 | 3,7E-03 |
| SLC35D1 | ENSG00000116704 | 1,84 | 5,7E-04 |
| SNX13 | ENSG00000071189 | 1,84 | 5,7E-05 |
| PDK1 | ENSG00000152256 | 1,84 | 3,4E-03 |
| ARFGEF2 | ENSG00000124198 | 1,84 | 1,7E-04 |
| EIF2A | ENSG00000144895 | 1,84 | 5,5E-05 |
| FAM135A | ENSG00000082269 | 1,84 | 4,3E-05 |
| PDGFC | ENSG00000145431 | 1,84 | 3,6E-04 |
| PGAM5 | ENSG00000247077 | 1,84 | 1,8E-05 |
| IPO11 | ENSG00000086200 | 1,84 | 5,1E-05 |
| UBTD2 | ENSG00000168246 | 1,84 | 4,3E-05 |
| C4orf33 | ENSG00000151470 | 1,84 | 7,4E-04 |
| CISD2 | ENSG00000145354 | 1,84 | 1,6E-04 |
| TRDMT1 | ENSG00000107614 | 1,84 | 4,8E-04 |
| NIPAL1 | ENSG00000163293 | 1,84 | 7,6E-04 |
| C5orf28 | ENSG00000151881 | 1,84 | 1,5E-04 |
| RAB28 | ENSG00000157869 | 1,83 | 7,8E-05 |
| ZNRF2 | ENSG00000180233 | 1,83 | 1,7E-04 |
| HAUS6 | ENSG00000147874 | 1,83 | 2,0E-05 |
| ANKRD28 | ENSG00000206560 | 1,83 | 7,0E-05 |
| MOSPD1 | ENSG00000101928 | 1,83 | 6,5E-05 |
| WWTR1 | ENSG00000018408 | 1,83 | 4,4E-05 |
| MPP5 | ENSG00000072415 | 1,83 | 6,1E-05 |
| TNFRSF10D | ENSG00000173530 | 1,83 | 8,7E-05 |
| GTF2H3 | ENSG00000111358 | 1,83 | 4,2E-05 |
| ZBTB44 | ENSG00000196323 | 1,83 | 1,8E-04 |
| ZNF780B | ENSG00000128000 | 1,83 | 3,7E-03 |
| BRWD1 | ENSG00000185658 | 1,83 | 5,1E-04 |
| RAB3GAP2 | ENSG00000118873 | 1,83 | 1,9E-04 |
| TRIM59 | ENSG00000213186 | 1,83 | 8,0E-05 |
| IDE | ENSG00000119912 | 1,83 | 2,7E-05 |
| CCDC68 | ENSG00000166510 | 1,83 | 6,8E-04 |
| COX15 | ENSG00000014919 | 1,83 | 2,5E-05 |
| TRERF1 | ENSG00000124496 | 1,83 | 1,1E-03 |
| VMA21 | ENSG00000160131 | 1,82 | 7,3E-05 |
| NLN | ENSG00000123213 | 1,82 | 2,1E-05 |
| GNS | ENSG00000135677 | 1,82 | 6,8E-05 |
| HSD17B12 | ENSG00000149084 | 1,82 | 9,5E-05 |
| ZXDA | ENSG00000198205 | 1,82 | 2,8E-03 |
| BMPR1A | ENSG00000107779 | 1,82 | 1,1E-04 |
| SKIL | ENSG00000136603 | 1,82 | 3,0E-04 |
| CREBZF | ENSG00000137504 | 1,82 | 1,8E-04 |
| CLASP2 | ENSG00000163539 | 1,82 | 1,0E-04 |
| PROM1 | ENSG00000007062 | 1,82 | 2,6E-04 |
| ZNF562 | ENSG00000171466 | 1,82 | 3,5E-04 |
| RIF1 | ENSG00000080345 | 1,82 | 1,6E-04 |
| KPNA4 | ENSG00000186432 | 1,81 | 4,9E-05 |
| NCOA7 | ENSG00000111912 | 1,81 | 5,9E-04 |
| UBL3 | ENSG00000122042 | 1,81 | 2,8E-05 |
| CNOT8 | ENSG00000155508 | 1,81 | 6,8E-05 |
| SLC39A6 | ENSG00000141424 | 1,81 | 3,6E-05 |
| MXD1 | ENSG00000059728 | 1,81 | 1,7E-03 |
| CCDC132 | ENSG00000004766 | 1,81 | 1,9E-03 |
| PTP4A2 | ENSG00000184007 | 1,81 | 1,2E-05 |
| C8orf37 | ENSG00000156172 | 1,81 | 7,9E-04 |
| TMEM68 | ENSG00000167904 | 1,81 | 1,9E-05 |
| SEMA3A | ENSG00000075213 | 1,81 | 2,5E-04 |
| B3GALTL | ENSG00000187676 | 1,81 | 1,3E-03 |
| RAB21 | ENSG00000080371 | 1,81 | 1,0E-04 |
| PAN3 | ENSG00000152520 | 1,81 | 2,8E-04 |
| ARRDC4 | ENSG00000140450 | 1,81 | 2,0E-04 |
| CHEK1 | ENSG00000149554 | 1,81 | 4,4E-05 |
| CAMK2D | ENSG00000145349 | 1,81 | 3,7E-04 |
| RAB30 | ENSG00000137502 | 1,81 | 7,2E-04 |
| HFE | ENSG00000010704 | 1,80 | 3,8E-03 |
| UBR7 | ENSG00000012963 | 1,80 | 5,7E-05 |
| RP11-142L4.3 | ENSG00000226723 | 1,80 | 7,2E-04 |
| FAM214A | ENSG00000047346 | 1,80 | 3,0E-03 |
| SEC22C | ENSG00000093183 | 1,80 | 1,1E-04 |
| CDK17 | ENSG00000059758 | 1,80 | 6,0E-05 |
| JPH1 | ENSG00000104369 | 1,80 | 8,6E-05 |
| RABGAP1L | ENSG00000152061 | 1,80 | 2,7E-03 |
| GABRG2 | ENSG00000113327 | 1,80 | 2,0E-03 |
| CCP110 | ENSG00000103540 | 1,80 | 6,6E-05 |
| NCKAP1 | ENSG00000061676 | 1,80 | 1,6E-05 |
| MFSD1 | ENSG00000118855 | 1,80 | 6,1E-04 |
| IL15RA | ENSG00000134470 | 1,80 | 6,3E-04 |
| RASA1 | ENSG00000145715 | 1,80 | 2,0E-04 |
| GTF2A1 | ENSG00000165417 | 1,80 | 1,2E-04 |
| RAB2A | ENSG00000104388 | 1,79 | 1,4E-04 |
| FER | ENSG00000151422 | 1,79 | 4,2E-04 |
| CD2AP | ENSG00000198087 | 1,79 | 1,4E-04 |
| ZNF468 | ENSG00000204604 | 1,79 | 8,3E-04 |
| ASF1A | ENSG00000111875 | 1,79 | 9,2E-04 |
| GALNT3 | ENSG00000115339 | 1,79 | 8,8E-04 |
| AGPAT5 | ENSG00000155189 | 1,79 | 1,0E-04 |
| SGCB | ENSG00000163069 | 1,79 | 6,2E-05 |
| DIAPH2 | ENSG00000147202 | 1,79 | 5,8E-03 |
| TP53INP1 | ENSG00000164938 | 1,79 | 7,1E-04 |
| ARMCX3 | ENSG00000102401 | 1,79 | 7,5E-04 |
| PRRG1 | ENSG00000130962 | 1,79 | 4,8E-04 |
| FBXL2 | ENSG00000153558 | 1,79 | 2,2E-03 |
| KPNA3 | ENSG00000102753 | 1,79 | 1,7E-05 |
| RP11-220I1.1 | ENSG00000233137 | 1,79 | 1,7E-04 |
| KLHL15 | ENSG00000174010 | 1,79 | 1,5E-04 |
| UBE2D2 | ENSG00000131508 | 1,79 | 2,0E-05 |
| QDPR | ENSG00000151552 | 1,79 | 1,0E-04 |
| NBPF10 | ENSG00000163386 | 1,78 | 5,3E-04 |
| MPP6 | ENSG00000105926 | 1,78 | 2,0E-04 |
| ANKEF1 | ENSG00000132623 | 1,78 | 9,3E-04 |
| KLF10 | ENSG00000155090 | 1,78 | 1,6E-04 |
| MTMR9 | ENSG00000104643 | 1,78 | 6,1E-04 |
| TRIM23 | ENSG00000113595 | 1,78 | 2,0E-04 |
| TUBD1 | ENSG00000108423 | 1,78 | 5,0E-04 |
| MED14 | ENSG00000180182 | 1,78 | 6,3E-05 |
| PTPN21 | ENSG00000070778 | 1,78 | 2,9E-03 |
| TMEM65 | ENSG00000164983 | 1,78 | 1,8E-04 |
| CALU | ENSG00000128595 | 1,78 | 9,8E-05 |
| FAM169A | ENSG00000198780 | 1,78 | 4,4E-05 |
| AVL9 | ENSG00000105778 | 1,78 | 1,7E-04 |
| RP11-307P22.1 | ENSG00000258445 | 1,78 | 7,7E-04 |
| ATMIN | ENSG00000166454 | 1,78 | 4,1E-05 |
| RAB9A | ENSG00000123595 | 1,78 | 2,6E-04 |
| COPS3 | ENSG00000141030 | 1,78 | 2,3E-04 |
| DCUN1D4 | ENSG00000109184 | 1,77 | 3,3E-05 |
| KBTBD6 | ENSG00000165572 | 1,77 | 3,0E-04 |
| ZBED3 | ENSG00000132846 | 1,77 | 2,2E-03 |
| OIP5-AS1 | ENSG00000247556 | 1,77 | 3,5E-05 |
| SLC11A2 | ENSG00000110911 | 1,77 | 6,5E-05 |
| GATC | ENSG00000257218 | 1,77 | 1,6E-04 |
| TRMT2B | ENSG00000188917 | 1,77 | 6,2E-04 |
| ACTR6 | ENSG00000075089 | 1,77 | 8,6E-04 |
| FAM134B | ENSG00000154153 | 1,77 | 5,6E-03 |
| SLC25A15 | ENSG00000102743 | 1,77 | 1,8E-04 |
| TRIM13 | ENSG00000204977 | 1,77 | 9,5E-05 |
| MAPK6 | ENSG00000069956 | 1,77 | 4,0E-05 |
| NDC1 | ENSG00000058804 | 1,77 | 1,8E-04 |
| AKAP7 | ENSG00000118507 | 1,77 | 3,3E-03 |
| DNAJB4 | ENSG00000162616 | 1,77 | 5,9E-04 |
| PUS10 | ENSG00000162927 | 1,77 | 5,7E-03 |
| NCOA2 | ENSG00000140396 | 1,77 | 3,6E-04 |
| GCH1 | ENSG00000131979 | 1,77 | 8,9E-05 |
| FEM1B | ENSG00000169018 | 1,77 | 2,3E-04 |
| IDS | ENSG00000010404 | 1,77 | 2,0E-04 |
| TMED8 | ENSG00000100580 | 1,77 | 4,0E-04 |
| CWF19L1 | ENSG00000095485 | 1,76 | 9,0E-05 |
| CD164 | ENSG00000135535 | 1,76 | 1,2E-04 |
| RP11-342D14.1 | ENSG00000229491 | 1,76 | 2,1E-03 |
| RPA3-AS1 | ENSG00000219545 | 1,76 | 2,6E-04 |
| DSC3 | ENSG00000134762 | 1,76 | 4,3E-03 |
| DTD2 | ENSG00000129480 | 1,76 | 3,5E-04 |
| PSEN1 | ENSG00000080815 | 1,76 | 2,4E-04 |
| FERMT2 | ENSG00000073712 | 1,76 | 8,1E-05 |
| JKAMP | ENSG00000050130 | 1,76 | 2,7E-04 |
| ZNF704 | ENSG00000164684 | 1,76 | 2,4E-03 |
| DUT | ENSG00000128951 | 1,76 | 2,0E-04 |
| PVRL3 | ENSG00000177707 | 1,76 | 1,1E-04 |
| MAP2 | ENSG00000078018 | 1,76 | 5,2E-03 |
| TRAPPC13 | ENSG00000113597 | 1,76 | 7,5E-04 |
| DMXL1 | ENSG00000172869 | 1,76 | 9,1E-05 |
| LYRM1 | ENSG00000102897 | 1,76 | 5,0E-05 |
| FAM60A | ENSG00000139146 | 1,76 | 1,4E-04 |
| RNF130 | ENSG00000113269 | 1,76 | 5,8E-05 |
| L2HGDH | ENSG00000087299 | 1,76 | 1,1E-03 |
| SH3BGRL | ENSG00000131171 | 1,76 | 1,6E-04 |
| CCSER2 | ENSG00000107771 | 1,76 | 7,7E-05 |
| MIER1 | ENSG00000198160 | 1,76 | 1,7E-04 |
| SIAH1 | ENSG00000196470 | 1,76 | 5,0E-04 |
| TMEM19 | ENSG00000139291 | 1,76 | 6,9E-04 |
| PIK3CA | ENSG00000121879 | 1,75 | 2,5E-04 |
| CDK14 | ENSG00000058091 | 1,75 | 7,6E-04 |
| CEP85L | ENSG00000111860 | 1,75 | 5,5E-03 |
| ERMP1 | ENSG00000099219 | 1,75 | 1,2E-03 |
| PRKRIR | ENSG00000137492 | 1,75 | 7,5E-05 |
| PIKFYVE | ENSG00000115020 | 1,75 | 9,7E-04 |
| SLC12A2 | ENSG00000064651 | 1,75 | 1,5E-04 |
| SLC35A1 | ENSG00000164414 | 1,75 | 1,5E-03 |
| CUL5 | ENSG00000166266 | 1,75 | 1,0E-04 |
| TMX4 | ENSG00000125827 | 1,75 | 2,2E-04 |
| CITED2 | ENSG00000164442 | 1,75 | 1,2E-04 |
| FUT11 | ENSG00000196968 | 1,75 | 2,2E-03 |
| TBC1D9 | ENSG00000109436 | 1,75 | 2,2E-03 |
| SREK1 | ENSG00000153914 | 1,75 | 3,6E-04 |
| HOXA13 | ENSG00000106031 | 1,75 | 3,5E-03 |
| CSGALNACT2 | ENSG00000169826 | 1,75 | 1,4E-04 |
| VTA1 | ENSG00000009844 | 1,75 | 4,0E-04 |
| UGP2 | ENSG00000169764 | 1,75 | 1,3E-04 |
| HAUS2 | ENSG00000137814 | 1,74 | 1,7E-04 |
| C6orf120 | ENSG00000185127 | 1,74 | 1,0E-04 |
| VPS41 | ENSG00000006715 | 1,74 | 1,3E-04 |
| IFNGR1 | ENSG00000027697 | 1,74 | 1,6E-04 |
| NUPL1 | ENSG00000139496 | 1,74 | 7,3E-05 |
| DPY19L3 | ENSG00000178904 | 1,74 | 5,9E-04 |
| RECQL | ENSG00000004700 | 1,74 | 4,2E-03 |
| NUDT4 | ENSG00000173598 | 1,74 | 3,6E-04 |
| PERP | ENSG00000112378 | 1,74 | 6,4E-05 |
| DESI2 | ENSG00000121644 | 1,74 | 1,1E-04 |
| KIAA2018 | ENSG00000176542 | 1,74 | 1,9E-03 |
| PDSS2 | ENSG00000164494 | 1,74 | 4,6E-04 |
| ATG4C | ENSG00000125703 | 1,74 | 6,3E-04 |
| TSPAN13 | ENSG00000106537 | 1,74 | 1,4E-04 |
| GFPT1 | ENSG00000198380 | 1,74 | 8,2E-05 |
| FAM84B | ENSG00000168672 | 1,74 | 3,4E-04 |
| MYBL1 | ENSG00000185697 | 1,74 | 6,0E-04 |
| LINC00657 | ENSG00000260032 | 1,74 | 7,4E-04 |
| SNX16 | ENSG00000104497 | 1,74 | 6,0E-04 |
| SLC9A6 | ENSG00000198689 | 1,74 | 7,8E-04 |
| MAP7 | ENSG00000135525 | 1,74 | 1,7E-04 |
| TNRC6A | ENSG00000090905 | 1,74 | 1,3E-04 |
| XPNPEP3 | ENSG00000196236 | 1,74 | 2,0E-04 |
| B3GNT2 | ENSG00000170340 | 1,73 | 1,1E-04 |
| C2CD5 | ENSG00000111731 | 1,73 | 5,8E-04 |
| RPL7L1 | ENSG00000146223 | 1,73 | 3,2E-05 |
| CDKN1B | ENSG00000111276 | 1,73 | 1,3E-04 |
| AC007405.6 | ENSG00000239467 | 1,73 | 2,4E-03 |
| TBC1D23 | ENSG00000036054 | 1,73 | 5,5E-04 |
| RNFT1 | ENSG00000189050 | 1,73 | 5,1E-04 |
| SLC18A2 | ENSG00000165646 | 1,73 | 3,7E-04 |
| ORC5 | ENSG00000164815 | 1,73 | 1,0E-03 |
| SUV39H2 | ENSG00000152455 | 1,73 | 7,4E-04 |
| SCOC | ENSG00000153130 | 1,73 | 6,6E-05 |
| DSE | ENSG00000111817 | 1,73 | 1,0E-04 |
| TMTC4 | ENSG00000125247 | 1,73 | 4,2E-04 |
| SMCHD1 | ENSG00000101596 | 1,73 | 6,1E-05 |
| QKI | ENSG00000112531 | 1,73 | 3,3E-04 |
| CTDSPL2 | ENSG00000137770 | 1,73 | 1,4E-04 |
| SSFA2 | ENSG00000138434 | 1,73 | 3,3E-05 |
| SEPT10 | ENSG00000186522 | 1,73 | 3,6E-05 |
| PDE8A | ENSG00000073417 | 1,73 | 2,3E-04 |
| LRRC1 | ENSG00000137269 | 1,73 | 1,7E-04 |
| NUP155 | ENSG00000113569 | 1,73 | 2,6E-04 |
| C5orf54 | ENSG00000221886 | 1,72 | 6,3E-04 |
| PTPN4 | ENSG00000088179 | 1,72 | 8,0E-04 |
| KIAA1462 | ENSG00000165757 | 1,72 | 2,8E-03 |
| IFT80 | ENSG00000068885 | 1,72 | 5,7E-03 |
| MTF2 | ENSG00000143033 | 1,72 | 1,5E-04 |
| KCTD9 | ENSG00000104756 | 1,72 | 1,5E-04 |
| SLC7A2 | ENSG00000003989 | 1,72 | 3,5E-03 |
| POLR3G | ENSG00000113356 | 1,72 | 2,9E-04 |
| UBR3 | ENSG00000144357 | 1,72 | 1,7E-04 |
| TNKS | ENSG00000173273 | 1,72 | 4,8E-04 |
| INPP5B | ENSG00000204084 | 1,72 | 1,3E-03 |
| FITM2 | ENSG00000197296 | 1,72 | 7,7E-04 |
| PPIP5K2 | ENSG00000145725 | 1,72 | 2,7E-04 |
| CPOX | ENSG00000080819 | 1,72 | 5,7E-05 |
| SDCBP | ENSG00000137575 | 1,72 | 4,8E-05 |
| PHLDA1 | ENSG00000139289 | 1,72 | 7,4E-05 |
| DTWD1 | ENSG00000104047 | 1,72 | 1,2E-03 |
| ZNF614 | ENSG00000142556 | 1,72 | 6,0E-03 |
| ZSWIM1 | ENSG00000168612 | 1,72 | 9,4E-04 |
| ZNF561 | ENSG00000171469 | 1,72 | 6,7E-04 |
| DNAJC6 | ENSG00000116675 | 1,72 | 3,8E-03 |
| ETV1 | ENSG00000006468 | 1,72 | 3,1E-04 |
| NR3C1 | ENSG00000113580 | 1,72 | 4,4E-04 |
| EPT1 | ENSG00000138018 | 1,72 | 1,8E-04 |
| TNFSF18 | ENSG00000120337 | 1,72 | 2,5E-03 |
| LRP11 | ENSG00000120256 | 1,72 | 1,4E-03 |
| C1GALT1 | ENSG00000106392 | 1,72 | 1,0E-04 |
| DIP2C | ENSG00000151240 | 1,71 | 1,5E-03 |
| TOMM70A | ENSG00000154174 | 1,71 | 9,7E-05 |
| B3GNT5 | ENSG00000176597 | 1,71 | 3,0E-04 |
| ST13 | ENSG00000100380 | 1,71 | 1,8E-04 |
| ELK4 | ENSG00000158711 | 1,71 | 2,8E-04 |
| TSPAN5 | ENSG00000168785 | 1,71 | 1,8E-04 |
| STK38L | ENSG00000211455 | 1,71 | 8,7E-05 |
| SPDL1 | ENSG00000040275 | 1,71 | 5,7E-04 |
| OTUD4 | ENSG00000164164 | 1,71 | 2,5E-04 |
| AC104389.28 | ENSG00000167355 | 1,71 | 3,9E-05 |
| CCDC91 | ENSG00000123106 | 1,71 | 4,3E-04 |
| BAG2 | ENSG00000112208 | 1,71 | 1,6E-03 |
| CHM | ENSG00000188419 | 1,71 | 6,6E-04 |
| SLC35G1 | ENSG00000176273 | 1,71 | 2,9E-04 |
| CCNT2 | ENSG00000082258 | 1,71 | 3,7E-04 |
| PTPLAD1 | ENSG00000074696 | 1,71 | 4,3E-04 |
| MED13 | ENSG00000108510 | 1,71 | 3,0E-04 |
| PARP8 | ENSG00000151883 | 1,71 | 5,5E-04 |
| MORC3 | ENSG00000159256 | 1,71 | 1,8E-04 |
| CCAT1 | ENSG00000247844 | 1,71 | 4,7E-03 |
| AJUBA | ENSG00000129474 | 1,71 | 1,8E-04 |
| KAT6A | ENSG00000083168 | 1,70 | 5,6E-03 |
| GAN | ENSG00000127688 | 1,70 | 1,5E-03 |
| CEP44 | ENSG00000164118 | 1,70 | 4,0E-03 |
| ERI1 | ENSG00000104626 | 1,70 | 4,1E-05 |
| ABCE1 | ENSG00000164163 | 1,70 | 1,0E-04 |
| VEGFA | ENSG00000112715 | 1,70 | 4,9E-04 |
| RIPK4 | ENSG00000183421 | 1,70 | 5,7E-03 |
| GSK3B | ENSG00000082701 | 1,70 | 6,7E-05 |
| SWAP70 | ENSG00000133789 | 1,70 | 1,3E-03 |
| UBQLN1 | ENSG00000135018 | 1,70 | 7,6E-05 |
| GPR157 | ENSG00000180758 | 1,70 | 3,2E-03 |
| CCNT1 | ENSG00000129315 | 1,70 | 1,0E-04 |
| TET2 | ENSG00000168769 | 1,70 | 1,3E-03 |
| SPTSSA | ENSG00000165389 | 1,70 | 1,2E-04 |
| ERCC4 | ENSG00000175595 | 1,70 | 3,3E-04 |
| RNF144A | ENSG00000151692 | 1,70 | 8,6E-04 |
| SOS1 | ENSG00000115904 | 1,69 | 5,0E-04 |
| TIA1 | ENSG00000116001 | 1,69 | 1,2E-04 |
| RBMX | ENSG00000147274 | 1,69 | 1,2E-04 |
| ZNF623 | ENSG00000183309 | 1,69 | 1,7E-04 |
| ZNF286A | ENSG00000187607 | 1,69 | 1,4E-03 |
| BCAR3 | ENSG00000137936 | 1,69 | 1,5E-03 |
| RABGAP1 | ENSG00000011454 | 1,69 | 1,8E-03 |
| ELOVL6 | ENSG00000170522 | 1,69 | 1,2E-04 |
| CNOT11 | ENSG00000158435 | 1,69 | 2,6E-04 |
| LRRC8D | ENSG00000171492 | 1,69 | 7,6E-04 |
| OSBPL9 | ENSG00000117859 | 1,69 | 8,6E-05 |
| TRAPPC2 | ENSG00000196459 | 1,69 | 4,9E-04 |
| RP11-89C21.2 | ENSG00000242629 | 1,69 | 2,4E-03 |
| FKBP5 | ENSG00000096060 | 1,69 | 1,3E-04 |
| GNPTAB | ENSG00000111670 | 1,69 | 9,3E-05 |
| ZYG11B | ENSG00000162378 | 1,69 | 8,8E-04 |
| MTAP | ENSG00000099810 | 1,69 | 3,1E-05 |
| GS1-251I9.4 | ENSG00000253738 | 1,69 | 1,4E-03 |
| ACTR2 | ENSG00000138071 | 1,69 | 6,5E-05 |
| CCNJ | ENSG00000107443 | 1,69 | 2,5E-03 |
| EEF1E1 | ENSG00000124802 | 1,69 | 3,5E-04 |
| RAB12 | ENSG00000206418 | 1,69 | 1,0E-04 |
| BAG4 | ENSG00000156735 | 1,69 | 1,6E-04 |
| ERO1L | ENSG00000197930 | 1,69 | 6,5E-05 |
| MET | ENSG00000105976 | 1,69 | 3,9E-04 |
| BEND6 | ENSG00000151917 | 1,69 | 3,0E-03 |
| NBPF9 | ENSG00000168614 | 1,69 | 3,1E-03 |
| SORT1 | ENSG00000134243 | 1,69 | 1,8E-03 |
| UAP1 | ENSG00000117143 | 1,69 | 1,7E-04 |
| OTUD6B | ENSG00000155100 | 1,69 | 1,5E-04 |
| STXBP5 | ENSG00000164506 | 1,69 | 1,4E-03 |
| CLPX | ENSG00000166855 | 1,68 | 2,9E-04 |
| PRKAA1 | ENSG00000132356 | 1,68 | 1,2E-04 |
| GRPEL2 | ENSG00000164284 | 1,68 | 3,6E-04 |
| SGOL2 | ENSG00000163535 | 1,68 | 1,2E-03 |
| MLK4 | ENSG00000143674 | 1,68 | 4,8E-04 |
| HOMER1 | ENSG00000152413 | 1,68 | 2,4E-04 |
| ARID2 | ENSG00000189079 | 1,68 | 3,3E-04 |
| APOBEC3F | ENSG00000128394 | 1,68 | 4,5E-03 |
| PIK3C2A | ENSG00000011405 | 1,68 | 3,8E-04 |
| SMAD5 | ENSG00000113658 | 1,68 | 7,7E-05 |
| PKN2 | ENSG00000065243 | 1,68 | 8,1E-05 |
| CNOT7P1 | ENSG00000233229 | 1,68 | 3,6E-04 |
| POLR2K | ENSG00000147669 | 1,68 | 3,2E-04 |
| RNF125 | ENSG00000101695 | 1,68 | 4,2E-03 |
| CREB1 | ENSG00000118260 | 1,68 | 2,8E-04 |
| MTMR11 | ENSG00000014914 | 1,68 | 4,9E-03 |
| PPP2R5E | ENSG00000154001 | 1,67 | 2,2E-04 |
| PURB | ENSG00000146676 | 1,67 | 2,4E-04 |
| COPS2 | ENSG00000166200 | 1,67 | 3,6E-04 |
| RYK | ENSG00000163785 | 1,67 | 4,5E-05 |
| NEK1 | ENSG00000137601 | 1,67 | 1,0E-03 |
| RAPGEF6 | ENSG00000158987 | 1,67 | 2,1E-03 |
| MSX2 | ENSG00000120149 | 1,67 | 1,8E-03 |
| PARM1 | ENSG00000169116 | 1,67 | 1,7E-03 |
| FZD5 | ENSG00000163251 | 1,67 | 2,0E-03 |
| RRN3 | ENSG00000085721 | 1,67 | 3,6E-04 |
| TMEM231 | ENSG00000205084 | 1,67 | 2,7E-03 |
| FAM172A | ENSG00000113391 | 1,67 | 3,6E-04 |
| C5orf15 | ENSG00000113583 | 1,67 | 3,0E-04 |
| FAM69A | ENSG00000154511 | 1,67 | 6,9E-04 |
| SPIN1 | ENSG00000106723 | 1,67 | 3,4E-04 |
| UBE2D3 | ENSG00000109332 | 1,67 | 6,2E-05 |
| MXRA7 | ENSG00000182534 | 1,67 | 1,2E-04 |
| ATAD5 | ENSG00000176208 | 1,67 | 2,7E-03 |
| RP11-329A14.1 | ENSG00000235105 | 1,67 | 7,7E-04 |
| CDC40 | ENSG00000168438 | 1,67 | 6,3E-04 |
| TMEM194A | ENSG00000166881 | 1,66 | 6,3E-04 |
| ITCH | ENSG00000078747 | 1,66 | 1,6E-04 |
| BTBD1 | ENSG00000064726 | 1,66 | 1,1E-04 |
| GEN1 | ENSG00000178295 | 1,66 | 4,3E-04 |
| MLLT3 | ENSG00000171843 | 1,66 | 4,7E-03 |
| LIMD1 | ENSG00000144791 | 1,66 | 2,0E-03 |
| NOC3L | ENSG00000173145 | 1,66 | 2,2E-04 |
| ZXDB | ENSG00000198455 | 1,66 | 5,5E-04 |
| VPS8 | ENSG00000156931 | 1,66 | 2,1E-03 |
| PTPN11 | ENSG00000179295 | 1,66 | 1,4E-04 |
| ADAM10 | ENSG00000137845 | 1,66 | 1,9E-04 |
| TAF4B | ENSG00000141384 | 1,66 | 3,0E-04 |
| FGD5-AS1 | ENSG00000225733 | 1,66 | 1,2E-04 |
| FAM98B | ENSG00000171262 | 1,66 | 4,5E-04 |
| GPR107 | ENSG00000148358 | 1,66 | 3,0E-03 |
| MCPH1 | ENSG00000147316 | 1,66 | 1,3E-03 |
| GOSR1 | ENSG00000108587 | 1,66 | 2,4E-04 |
| PCGF6 | ENSG00000156374 | 1,65 | 6,4E-04 |
| TUBE1 | ENSG00000074935 | 1,65 | 1,5E-04 |
| NFE2L3 | ENSG00000050344 | 1,65 | 2,6E-04 |
| TAPT1 | ENSG00000169762 | 1,65 | 9,1E-04 |
| DNAAF2 | ENSG00000165506 | 1,65 | 1,3E-03 |
| TOPBP1 | ENSG00000163781 | 1,65 | 3,1E-04 |
| C4orf46 | ENSG00000205208 | 1,65 | 2,0E-04 |
| SLC38A2 | ENSG00000134294 | 1,65 | 2,7E-04 |
| ZNF175 | ENSG00000105497 | 1,65 | 1,7E-03 |
| TFRC | ENSG00000072274 | 1,65 | 2,0E-04 |
| LONRF3 | ENSG00000175556 | 1,65 | 2,7E-03 |
| MCOLN3 | ENSG00000055732 | 1,65 | 7,9E-04 |
| TPK1 | ENSG00000196511 | 1,65 | 1,5E-03 |
| ZBTB34 | ENSG00000177125 | 1,64 | 1,3E-03 |
| ANXA10 | ENSG00000109511 | 1,64 | 3,1E-03 |
| SLC25A16 | ENSG00000122912 | 1,64 | 1,4E-03 |
| ZNF292 | ENSG00000188994 | 1,64 | 1,9E-03 |
| SLC39A9 | ENSG00000029364 | 1,64 | 2,7E-04 |
| ZNF678 | ENSG00000181450 | 1,64 | 6,2E-03 |
| RAP1GDS1 | ENSG00000138698 | 1,64 | 1,0E-04 |
| IMPAD1 | ENSG00000104331 | 1,64 | 1,0E-04 |
| ATP6V1B2 | ENSG00000147416 | 1,64 | 2,7E-04 |
| GJC1 | ENSG00000182963 | 1,64 | 6,2E-04 |
| WWP1P1 | ENSG00000244153 | 1,64 | 4,8E-04 |
| PRMT3 | ENSG00000185238 | 1,64 | 5,5E-04 |
| CDC42SE2 | ENSG00000158985 | 1,64 | 4,8E-04 |
| ANKRD13C | ENSG00000118454 | 1,64 | 4,6E-04 |
| PEX1 | ENSG00000127980 | 1,64 | 2,5E-04 |
| C17orf75 | ENSG00000108666 | 1,64 | 1,2E-03 |
| C3orf17 | ENSG00000163608 | 1,64 | 1,7E-04 |
| ZNF367 | ENSG00000165244 | 1,64 | 7,3E-04 |
| SYNCRIP | ENSG00000135316 | 1,64 | 7,8E-05 |
| FAS | ENSG00000026103 | 1,64 | 3,1E-03 |
| ATP2B4 | ENSG00000058668 | 1,64 | 4,1E-03 |
| ZDHHC17 | ENSG00000186908 | 1,64 | 5,7E-04 |
| SLC30A9 | ENSG00000014824 | 1,64 | 1,5E-04 |
| MEF2A | ENSG00000068305 | 1,64 | 4,7E-04 |
| CYB5R4 | ENSG00000065615 | 1,63 | 8,0E-05 |
| NT5E | ENSG00000135318 | 1,63 | 7,9E-04 |
| MALT1 | ENSG00000172175 | 1,63 | 6,3E-05 |
| CYP24A1 | ENSG00000019186 | 1,63 | 3,2E-04 |
| C5orf34 | ENSG00000172244 | 1,63 | 2,4E-03 |
| OSMR | ENSG00000145623 | 1,63 | 5,8E-04 |
| GYG1 | ENSG00000163754 | 1,63 | 4,8E-04 |
| CEP192 | ENSG00000101639 | 1,63 | 4,3E-03 |
| MAPRE2 | ENSG00000166974 | 1,63 | 1,3E-03 |
| IRAK4 | ENSG00000198001 | 1,63 | 7,0E-04 |
| HDHD1 | ENSG00000130021 | 1,63 | 5,3E-04 |
| FAM210A | ENSG00000177150 | 1,63 | 8,2E-04 |
| MAP4K3 | ENSG00000011566 | 1,63 | 4,7E-04 |
| NCOA4 | ENSG00000138293 | 1,63 | 8,0E-05 |
| LMBR1 | ENSG00000105983 | 1,63 | 3,3E-04 |
| RBM47 | ENSG00000163694 | 1,63 | 1,5E-03 |
| STAG2 | ENSG00000101972 | 1,62 | 1,3E-04 |
| FBXO45 | ENSG00000174013 | 1,62 | 1,7E-04 |
| ZNF596 | ENSG00000172748 | 1,62 | 5,3E-03 |
| TTK | ENSG00000112742 | 1,62 | 5,5E-04 |
| MINPP1 | ENSG00000107789 | 1,62 | 3,7E-04 |
| UBE3A | ENSG00000114062 | 1,62 | 1,2E-04 |
| DDX58 | ENSG00000107201 | 1,62 | 4,7E-03 |
| DUSP12 | ENSG00000081721 | 1,62 | 4,1E-04 |
| LRCH3 | ENSG00000186001 | 1,62 | 1,4E-03 |
| ARNTL | ENSG00000133794 | 1,62 | 2,0E-03 |
| KANSL1L | ENSG00000144445 | 1,62 | 7,4E-04 |
| PLS3 | ENSG00000102024 | 1,62 | 2,4E-04 |
| KIF11 | ENSG00000138160 | 1,62 | 8,5E-05 |
| DLEU2 | ENSG00000231607 | 1,62 | 3,6E-03 |
| TTC37 | ENSG00000198677 | 1,62 | 2,7E-04 |
| ALS2 | ENSG00000003393 | 1,62 | 5,0E-04 |
| YWHAZ | ENSG00000164924 | 1,62 | 6,3E-05 |
| RAD17 | ENSG00000152942 | 1,62 | 4,6E-04 |
| KLHDC10 | ENSG00000128607 | 1,62 | 5,3E-03 |
| TDG | ENSG00000139372 | 1,62 | 1,4E-03 |
| THUMPD1 | ENSG00000066654 | 1,61 | 5,6E-04 |
| TRAM1 | ENSG00000067167 | 1,61 | 1,1E-03 |
| C12orf73 | ENSG00000204954 | 1,61 | 8,4E-05 |
| SLC25A43 | ENSG00000077713 | 1,61 | 3,1E-04 |
| STRN3 | ENSG00000196792 | 1,61 | 2,6E-04 |
| PUM1 | ENSG00000134644 | 1,61 | 3,0E-04 |
| ENDOD1 | ENSG00000149218 | 1,61 | 2,1E-03 |
| KIF2A | ENSG00000068796 | 1,61 | 1,8E-04 |
| ZNF700 | ENSG00000196757 | 1,61 | 3,8E-03 |
| ZHX1 | ENSG00000165156 | 1,61 | 3,6E-04 |
| STK3 | ENSG00000104375 | 1,61 | 4,0E-04 |
| METTL10 | ENSG00000203791 | 1,61 | 4,3E-04 |
| ARHGAP11A | ENSG00000198826 | 1,61 | 1,3E-04 |
| EIF5 | ENSG00000100664 | 1,61 | 2,2E-04 |
| SERPINB5 | ENSG00000206075 | 1,61 | 4,3E-04 |
| RNF13 | ENSG00000082996 | 1,61 | 2,9E-04 |
| GATA6 | ENSG00000141448 | 1,61 | 1,4E-03 |
| ARL13B | ENSG00000169379 | 1,61 | 3,4E-03 |
| ZBTB24 | ENSG00000112365 | 1,61 | 5,5E-04 |
| AGA | ENSG00000038002 | 1,61 | 4,4E-03 |
| APPBP2 | ENSG00000062725 | 1,61 | 4,4E-04 |
| KDSR | ENSG00000119537 | 1,61 | 1,6E-04 |
| MIS12 | ENSG00000167842 | 1,61 | 6,8E-04 |
| DHX36 | ENSG00000174953 | 1,61 | 4,3E-04 |
| RP11-421L21.3 | ENSG00000233184 | 1,61 | 2,0E-03 |
| ZBTB10 | ENSG00000205189 | 1,61 | 8,7E-04 |
| LYSMD2 | ENSG00000140280 | 1,61 | 5,3E-04 |
| CYP51A1P2 | ENSG00000233588 | 1,61 | 4,4E-03 |
| PXK | ENSG00000168297 | 1,60 | 1,3E-03 |
| CASP8 | ENSG00000064012 | 1,60 | 1,1E-03 |
| UBE2K | ENSG00000078140 | 1,60 | 1,2E-04 |
| CBFB | ENSG00000067955 | 1,60 | 3,0E-04 |
| GTF3C4 | ENSG00000125484 | 1,60 | 1,3E-04 |
| RBM18 | ENSG00000119446 | 1,60 | 2,5E-04 |
| TTLL7 | ENSG00000137941 | 1,60 | 9,8E-04 |
| FZD6 | ENSG00000164930 | 1,60 | 4,9E-04 |
| DDX50 | ENSG00000107625 | 1,60 | 5,9E-04 |
| TTL | ENSG00000114999 | 1,60 | 3,2E-04 |
| RNF146 | ENSG00000118518 | 1,60 | 2,3E-03 |
| SLC41A2 | ENSG00000136052 | 1,60 | 1,4E-03 |
| NUDT12 | ENSG00000112874 | 1,60 | 1,2E-03 |
| ARHGAP12 | ENSG00000165322 | 1,60 | 1,4E-04 |
| IREB2 | ENSG00000136381 | 1,60 | 8,2E-04 |
| DDI2 | ENSG00000197312 | 1,60 | 4,7E-03 |
| MOSPD2 | ENSG00000130150 | 1,60 | 2,0E-03 |
| INSIG1 | ENSG00000186480 | 1,60 | 2,2E-04 |
| EGLN1 | ENSG00000135766 | 1,60 | 1,5E-04 |
| ADH5 | ENSG00000197894 | 1,60 | 4,2E-04 |
| MB21D2 | ENSG00000180611 | 1,60 | 1,3E-03 |
| NARG2 | ENSG00000128915 | 1,60 | 1,5E-04 |
| SIK2 | ENSG00000170145 | 1,59 | 2,6E-03 |
| POT1 | ENSG00000128513 | 1,59 | 2,7E-04 |
| DPH5 | ENSG00000117543 | 1,59 | 4,8E-04 |
| STAM2 | ENSG00000115145 | 1,59 | 4,2E-04 |
| ERCC8 | ENSG00000049167 | 1,59 | 6,3E-04 |
| WSB2 | ENSG00000176871 | 1,59 | 1,4E-04 |
| VPS35 | ENSG00000069329 | 1,59 | 1,2E-04 |
| VKORC1L1 | ENSG00000196715 | 1,59 | 2,2E-04 |
| TBC1D32 | ENSG00000146350 | 1,59 | 5,2E-03 |
| SSR1 | ENSG00000124783 | 1,59 | 2,0E-04 |
| AP5M1 | ENSG00000053770 | 1,59 | 2,9E-04 |
| ZADH2 | ENSG00000180011 | 1,59 | 6,6E-04 |
| DLAT | ENSG00000150768 | 1,59 | 8,8E-05 |
| MSMO1 | ENSG00000052802 | 1,59 | 2,3E-04 |
| KLHL20 | ENSG00000076321 | 1,59 | 3,4E-03 |
| SIAH2 | ENSG00000181788 | 1,59 | 4,7E-04 |
| RNF20 | ENSG00000155827 | 1,59 | 4,2E-03 |
| ALCAM | ENSG00000170017 | 1,59 | 7,6E-04 |
| MYO1B | ENSG00000128641 | 1,59 | 2,3E-04 |
| DENND4A | ENSG00000174485 | 1,59 | 5,3E-03 |
| PDE12 | ENSG00000174840 | 1,59 | 5,5E-04 |
| COMMD2 | ENSG00000114744 | 1,59 | 1,3E-03 |
| MED21 | ENSG00000152944 | 1,59 | 3,4E-04 |
| ATP2C1 | ENSG00000017260 | 1,59 | 7,6E-05 |
| HELZ | ENSG00000198265 | 1,58 | 6,3E-04 |
| CUL4B | ENSG00000158290 | 1,58 | 1,6E-04 |
| DPYSL2 | ENSG00000092964 | 1,58 | 1,1E-03 |
| E2F5 | ENSG00000133740 | 1,58 | 1,4E-03 |
| SLC30A5 | ENSG00000145740 | 1,58 | 2,5E-04 |
| TEX2 | ENSG00000136478 | 1,58 | 6,3E-04 |
| PGM2 | ENSG00000169299 | 1,58 | 1,7E-04 |
| WDR48 | ENSG00000114742 | 1,58 | 3,8E-04 |
| FAM105A | ENSG00000145569 | 1,58 | 2,7E-03 |
| ADSS | ENSG00000035687 | 1,58 | 3,9E-04 |
| RIOK3 | ENSG00000101782 | 1,58 | 1,9E-04 |
| HNRNPA3 | ENSG00000170144 | 1,58 | 5,5E-04 |
| KIAA0196 | ENSG00000164961 | 1,58 | 2,9E-04 |
| ALG8 | ENSG00000159063 | 1,58 | 7,7E-04 |
| ANKRD50 | ENSG00000151458 | 1,58 | 7,8E-04 |
| CAMSAP2 | ENSG00000118200 | 1,58 | 2,1E-04 |
| STARD3NL | ENSG00000010270 | 1,58 | 1,3E-04 |
| NUP35 | ENSG00000163002 | 1,58 | 2,5E-03 |
| GPR110 | ENSG00000153292 | 1,58 | 3,1E-04 |
| GPSM2 | ENSG00000121957 | 1,58 | 1,4E-03 |
| MFSD8 | ENSG00000164073 | 1,58 | 8,9E-04 |
| RAB3B | ENSG00000169213 | 1,58 | 1,7E-03 |
| HSD17B11 | ENSG00000198189 | 1,58 | 2,4E-04 |
| CALML4 | ENSG00000129007 | 1,58 | 4,2E-03 |
| NARS2 | ENSG00000137513 | 1,58 | 1,6E-03 |
| PDE4B | ENSG00000184588 | 1,58 | 6,9E-04 |
| EPB41L4B | ENSG00000095203 | 1,58 | 5,1E-04 |
| AIDA | ENSG00000186063 | 1,58 | 9,8E-04 |
| NAMPT | ENSG00000105835 | 1,58 | 2,4E-04 |
| NMRK1 | ENSG00000106733 | 1,58 | 3,1E-03 |
| F2R | ENSG00000181104 | 1,58 | 1,4E-03 |
| ACADM | ENSG00000117054 | 1,58 | 7,1E-04 |
| MARCH7 | ENSG00000136536 | 1,57 | 8,1E-04 |
| CCBL2 | ENSG00000137944 | 1,57 | 1,8E-03 |
| DPH3 | ENSG00000154813 | 1,57 | 5,1E-04 |
| ELP2 | ENSG00000134759 | 1,57 | 3,2E-04 |
| ACSL1 | ENSG00000151726 | 1,57 | 8,3E-04 |
| DSC2 | ENSG00000134755 | 1,57 | 3,5E-04 |
| CD55 | ENSG00000196352 | 1,57 | 5,4E-04 |
| MARS2 | ENSG00000247626 | 1,57 | 1,3E-03 |
| SNAPC1 | ENSG00000023608 | 1,57 | 1,7E-03 |
| GLMN | ENSG00000174842 | 1,57 | 3,3E-03 |
| FBXO21 | ENSG00000135108 | 1,57 | 3,9E-04 |
| RCOR1 | ENSG00000089902 | 1,57 | 6,6E-04 |
| FBXO28 | ENSG00000143756 | 1,57 | 4,9E-04 |
| KDM1B | ENSG00000165097 | 1,57 | 1,9E-03 |
| AK4 | ENSG00000162433 | 1,57 | 4,8E-04 |
| AP4E1 | ENSG00000081014 | 1,57 | 3,7E-03 |
| STX2 | ENSG00000111450 | 1,57 | 2,9E-04 |
| RIOK2 | ENSG00000058729 | 1,57 | 4,2E-03 |
| ZDHHC13 | ENSG00000177054 | 1,57 | 2,8E-04 |
| PHF20L1 | ENSG00000129292 | 1,57 | 6,5E-04 |
| CAPN7 | ENSG00000131375 | 1,57 | 7,1E-04 |
| SLC16A9 | ENSG00000165449 | 1,57 | 1,4E-03 |
| GUF1 | ENSG00000151806 | 1,56 | 4,4E-04 |
| SAMD12 | ENSG00000177570 | 1,56 | 5,3E-03 |
| UBE2N | ENSG00000177889 | 1,56 | 4,8E-04 |
| SLC35B3 | ENSG00000124786 | 1,56 | 4,2E-03 |
| TTC13 | ENSG00000143643 | 1,56 | 2,5E-03 |
| GSTCD | ENSG00000138780 | 1,56 | 1,5E-03 |
| CNST | ENSG00000162852 | 1,56 | 1,2E-03 |
| FAM92A1 | ENSG00000188343 | 1,56 | 5,6E-04 |
| WDYHV1 | ENSG00000156795 | 1,56 | 1,2E-04 |
| LIN54 | ENSG00000189308 | 1,56 | 6,9E-04 |
| DPY19L1 | ENSG00000173852 | 1,56 | 6,1E-04 |
| CCDC71L | ENSG00000253276 | 1,56 | 2,8E-03 |
| CLIP4 | ENSG00000115295 | 1,56 | 3,6E-04 |
| UBE2B | ENSG00000119048 | 1,56 | 6,1E-04 |
| TULP4 | ENSG00000130338 | 1,56 | 6,0E-03 |
| SLC35B4 | ENSG00000205060 | 1,56 | 2,2E-03 |
| SUPT20H | ENSG00000102710 | 1,56 | 4,6E-04 |
| GCNT1 | ENSG00000187210 | 1,56 | 5,7E-03 |
| RCAN1 | ENSG00000159200 | 1,56 | 1,8E-03 |
| KLHL42 | ENSG00000087448 | 1,56 | 4,6E-04 |
| SCML1 | ENSG00000047634 | 1,56 | 1,6E-03 |
| UBR1 | ENSG00000159459 | 1,56 | 7,2E-04 |
| FOXN3 | ENSG00000053254 | 1,55 | 1,9E-03 |
| TMEM106C | ENSG00000134291 | 1,55 | 1,6E-04 |
| DCAF10 | ENSG00000122741 | 1,55 | 9,4E-04 |
| USP25 | ENSG00000155313 | 1,55 | 1,8E-03 |
| BRWD3 | ENSG00000165288 | 1,55 | 3,9E-03 |
| TGDS | ENSG00000088451 | 1,55 | 1,1E-03 |
| PLEKHA3 | ENSG00000116095 | 1,55 | 3,6E-04 |
| COPS8P2 | ENSG00000214552 | 1,55 | 1,6E-03 |
| DDX3X | ENSG00000215301 | 1,55 | 5,0E-04 |
| CAV1 | ENSG00000105974 | 1,55 | 1,0E-03 |
| TPST2 | ENSG00000128294 | 1,55 | 1,0E-03 |
| AFF4 | ENSG00000072364 | 1,55 | 2,7E-03 |
| KLHL9 | ENSG00000198642 | 1,55 | 2,0E-04 |
| CLINT1 | ENSG00000113282 | 1,55 | 4,6E-04 |
| MEX3C | ENSG00000176624 | 1,55 | 7,6E-04 |
| GPATCH2 | ENSG00000092978 | 1,55 | 8,1E-04 |
| CDK8 | ENSG00000132964 | 1,55 | 3,2E-04 |
| RFWD2 | ENSG00000143207 | 1,55 | 9,0E-04 |
| ATP6V1C1 | ENSG00000155097 | 1,55 | 4,7E-04 |
| OSGEPL1 | ENSG00000128694 | 1,55 | 9,0E-04 |
| TPD52 | ENSG00000076554 | 1,55 | 3,2E-04 |
| SET | ENSG00000119335 | 1,55 | 1,9E-04 |
| UBASH3B | ENSG00000154127 | 1,55 | 8,7E-04 |
| AZIN1 | ENSG00000155096 | 1,54 | 1,6E-04 |
| ESRP1 | ENSG00000104413 | 1,54 | 4,6E-04 |
| STRADB | ENSG00000082146 | 1,54 | 6,9E-04 |
| SEPT10P1 | ENSG00000253541 | 1,54 | 4,2E-03 |
| CA13 | ENSG00000185015 | 1,54 | 1,1E-03 |
| FAM107B | ENSG00000065809 | 1,54 | 6,5E-04 |
| PHKB | ENSG00000102893 | 1,54 | 4,3E-04 |
| ZNF557 | ENSG00000130544 | 1,54 | 2,8E-03 |
| EIF1AXP1 | ENSG00000236698 | 1,54 | 5,3E-03 |
| PLA2G12A | ENSG00000123739 | 1,54 | 1,1E-04 |
| IPO7 | ENSG00000205339 | 1,54 | 8,1E-04 |
| SUCLA2 | ENSG00000136143 | 1,54 | 9,3E-04 |
| PDS5A | ENSG00000121892 | 1,54 | 2,6E-04 |
| RP11-513I15.6 | ENSG00000225339 | 1,54 | 2,7E-03 |
| TBC1D19 | ENSG00000109680 | 1,54 | 1,7E-03 |
| C9orf72 | ENSG00000147894 | 1,54 | 6,0E-04 |
| ANKRA2 | ENSG00000164331 | 1,54 | 1,7E-03 |
| METTL4 | ENSG00000101574 | 1,54 | 2,9E-04 |
| DGKG | ENSG00000058866 | 1,54 | 2,3E-03 |
| TOR1AIP2 | ENSG00000169905 | 1,54 | 2,5E-04 |
| RND3 | ENSG00000115963 | 1,53 | 2,3E-03 |
| RFC3 | ENSG00000133119 | 1,53 | 1,1E-03 |
| JAK2 | ENSG00000096968 | 1,53 | 6,1E-03 |
| CPPED1 | ENSG00000103381 | 1,53 | 2,5E-03 |
| C3orf38 | ENSG00000179021 | 1,53 | 2,1E-03 |
| CDC7 | ENSG00000097046 | 1,53 | 4,4E-04 |
| RRM2 | ENSG00000171848 | 1,53 | 4,8E-04 |
| TBPL1 | ENSG00000028839 | 1,53 | 1,7E-03 |
| XBP1 | ENSG00000100219 | 1,53 | 1,1E-03 |
| TOPORS | ENSG00000197579 | 1,53 | 2,0E-03 |
| ANLN | ENSG00000011426 | 1,53 | 2,7E-04 |
| QRSL1 | ENSG00000130348 | 1,53 | 9,8E-04 |
| FASTKD1 | ENSG00000138399 | 1,53 | 2,4E-03 |
| ATAD2B | ENSG00000119778 | 1,53 | 4,5E-03 |
| RHOBTB3 | ENSG00000164292 | 1,53 | 3,0E-04 |
| RNF4 | ENSG00000063978 | 1,53 | 3,0E-04 |
| NFIA | ENSG00000162599 | 1,53 | 8,6E-04 |
| ROCK2 | ENSG00000134318 | 1,53 | 1,1E-03 |
| SACS | ENSG00000151835 | 1,53 | 1,1E-03 |
| PMAIP1 | ENSG00000141682 | 1,52 | 4,4E-03 |
| SEC23B | ENSG00000101310 | 1,52 | 5,5E-04 |
| NBN | ENSG00000104320 | 1,52 | 1,1E-03 |
| MAPK14 | ENSG00000112062 | 1,52 | 2,1E-03 |
| MAPK8 | ENSG00000107643 | 1,52 | 7,1E-04 |
| GXYLT2 | ENSG00000172986 | 1,52 | 5,5E-03 |
| GTF2B | ENSG00000137947 | 1,52 | 2,5E-03 |
| METTL15 | ENSG00000169519 | 1,52 | 1,3E-03 |
| RP11-351I24.3 | ENSG00000254719 | 1,52 | 1,1E-03 |
| PPA2 | ENSG00000138777 | 1,52 | 8,2E-04 |
| SRD5A1 | ENSG00000145545 | 1,52 | 2,0E-03 |
| CSNK1G1 | ENSG00000169118 | 1,52 | 1,1E-03 |
| ESYT2 | ENSG00000117868 | 1,52 | 2,4E-04 |
| ARGLU1 | ENSG00000134884 | 1,52 | 8,9E-04 |
| USP15 | ENSG00000135655 | 1,52 | 1,3E-03 |
| RAB10 | ENSG00000084733 | 1,52 | 6,2E-04 |
| FECH | ENSG00000066926 | 1,52 | 3,6E-04 |
| LEPROT | ENSG00000213625 | 1,52 | 2,3E-03 |
| NDUFS1 | ENSG00000023228 | 1,52 | 8,0E-04 |
| RNFT2 | ENSG00000135119 | 1,52 | 3,9E-03 |
| TYW3 | ENSG00000162623 | 1,51 | 1,4E-03 |
| PFN2 | ENSG00000070087 | 1,51 | 1,6E-04 |
| RLIM | ENSG00000131263 | 1,51 | 4,7E-04 |
| KIRREL | ENSG00000183853 | 1,51 | 2,8E-03 |
| TMEM194B | ENSG00000189362 | 1,51 | 1,7E-03 |
| RAD18 | ENSG00000070950 | 1,51 | 2,9E-03 |
| SBF2-AS1 | ENSG00000246273 | 1,51 | 5,3E-03 |
| CEP170 | ENSG00000143702 | 1,51 | 7,4E-04 |
| NUP160 | ENSG00000030066 | 1,51 | 2,3E-04 |
| RRAGD | ENSG00000025039 | 1,51 | 5,9E-04 |
| NECAP1 | ENSG00000089818 | 1,51 | 2,3E-03 |
| FAM208A | ENSG00000163946 | 1,51 | 5,3E-04 |
| MAOA | ENSG00000189221 | 1,51 | 1,9E-03 |
| CASC7 | ENSG00000259758 | 1,51 | 2,7E-03 |
| AC104297.1 | ENSG00000213383 | 1,51 | 2,9E-03 |
| TRAK2 | ENSG00000115993 | 1,50 | 1,5E-03 |
| SERBP1 | ENSG00000142864 | 1,50 | 3,8E-04 |
| CXorf57 | ENSG00000147231 | 1,50 | 5,6E-03 |
| YTHDC2 | ENSG00000047188 | 1,50 | 2,0E-03 |
| RAB31 | ENSG00000168461 | 1,50 | 4,1E-04 |
| AP1G1 | ENSG00000166747 | 1,50 | 7,2E-04 |
| SAMD13 | ENSG00000203943 | 1,50 | 2,6E-03 |
| NUP54 | ENSG00000138750 | 1,50 | 1,5E-03 |
| RNF38 | ENSG00000137075 | 1,50 | 6,4E-04 |
| RNF11 | ENSG00000123091 | 1,50 | 1,6E-04 |
| SLC33A1 | ENSG00000169359 | 1,50 | 5,4E-04 |
| ZNF558 | ENSG00000167785 | 1,50 | 9,0E-04 |
| PARG | ENSG00000227345 | 1,50 | 3,8E-03 |
| AAED1 | ENSG00000158122 | 1,50 | 1,1E-03 |
| UBA3 | ENSG00000144744 | 1,50 | 1,1E-03 |
| IER3IP1 | ENSG00000134049 | 1,50 | 4,1E-04 |
| RAD54B | ENSG00000197275 | 1,50 | 1,7E-03 |
| LTN1 | ENSG00000198862 | 1,50 | 3,8E-03 |
| CYBRD1 | ENSG00000071967 | 1,49 | 1,0E-03 |
| SENP5 | ENSG00000119231 | 1,49 | 6,2E-04 |
| PPT1 | ENSG00000131238 | 1,49 | 1,7E-03 |
| ZNF652 | ENSG00000198740 | 1,49 | 4,9E-03 |
| TBC1D15 | ENSG00000121749 | 1,49 | 8,6E-04 |
| RAB18 | ENSG00000099246 | 1,49 | 1,9E-03 |
| MBP | ENSG00000197971 | 1,49 | 1,1E-03 |
| CLTC | ENSG00000141367 | 1,49 | 1,6E-04 |
| VTI1B | ENSG00000100568 | 1,49 | 4,2E-04 |
| ATP6V1A | ENSG00000114573 | 1,49 | 6,6E-04 |
| SMURF1 | ENSG00000198742 | 1,49 | 3,4E-03 |
| BRCC3 | ENSG00000185515 | 1,49 | 4,0E-03 |
| TCEA1 | ENSG00000187735 | 1,49 | 5,8E-04 |
| ACOX1 | ENSG00000161533 | 1,49 | 3,3E-04 |
| BBS10 | ENSG00000179941 | 1,49 | 5,9E-04 |
| CTNNAL1 | ENSG00000119326 | 1,49 | 7,7E-04 |
| RCN2 | ENSG00000117906 | 1,49 | 1,6E-04 |
| PDE7A | ENSG00000205268 | 1,49 | 1,4E-03 |
| NLK | ENSG00000087095 | 1,49 | 1,8E-03 |
| GLCE | ENSG00000138604 | 1,48 | 9,7E-04 |
| FAM98A | ENSG00000119812 | 1,48 | 1,2E-03 |
| TMEM182 | ENSG00000170417 | 1,48 | 2,5E-03 |
| CLCN3 | ENSG00000109572 | 1,48 | 6,9E-04 |
| RNF14 | ENSG00000013561 | 1,48 | 5,9E-04 |
| NANP | ENSG00000170191 | 1,48 | 8,2E-04 |
| TMEM167B | ENSG00000215717 | 1,48 | 6,0E-04 |
| PRPF39 | ENSG00000185246 | 1,48 | 1,6E-03 |
| IL13RA1 | ENSG00000131724 | 1,48 | 1,3E-03 |
| ATAD1 | ENSG00000138138 | 1,48 | 3,2E-04 |
| ZNF644 | ENSG00000122482 | 1,48 | 3,7E-04 |
| DNAJC13 | ENSG00000138246 | 1,48 | 3,3E-03 |
| SRPX | ENSG00000101955 | 1,48 | 3,3E-03 |
| MED23 | ENSG00000112282 | 1,48 | 3,0E-03 |
| GJA3 | ENSG00000121743 | 1,48 | 1,1E-03 |
| EEF2K | ENSG00000103319 | 1,48 | 2,0E-03 |
| PABPC1P3 | ENSG00000230673 | 1,48 | 4,7E-04 |
| FAM217B | ENSG00000196227 | 1,48 | 1,7E-03 |
| SBF2 | ENSG00000133812 | 1,48 | 4,7E-03 |
| EMP2 | ENSG00000213853 | 1,48 | 1,1E-03 |
| KIAA1432 | ENSG00000107036 | 1,48 | 3,6E-03 |
| SCLT1 | ENSG00000151466 | 1,48 | 3,8E-03 |
| FGFR1OP2 | ENSG00000111790 | 1,48 | 3,9E-04 |
| MLH1 | ENSG00000076242 | 1,48 | 4,3E-03 |
| ZFP91 | ENSG00000186660 | 1,48 | 8,0E-04 |
| NCEH1 | ENSG00000144959 | 1,48 | 4,7E-03 |
| DDX20 | ENSG00000064703 | 1,48 | 3,1E-03 |
| DSCC1 | ENSG00000136982 | 1,47 | 4,1E-04 |
| ZNF711 | ENSG00000147180 | 1,47 | 4,6E-03 |
| PSMG2 | ENSG00000128789 | 1,47 | 4,5E-04 |
| CMTM4 | ENSG00000183723 | 1,47 | 1,5E-03 |
| PAPD4 | ENSG00000164329 | 1,47 | 3,2E-04 |
| INPP4B | ENSG00000109452 | 1,47 | 1,0E-03 |
| PDSS1 | ENSG00000148459 | 1,47 | 5,1E-03 |
| WWC2 | ENSG00000151718 | 1,47 | 2,9E-03 |
| C16orf72 | ENSG00000182831 | 1,47 | 8,1E-04 |
| SCARB2 | ENSG00000138760 | 1,47 | 7,9E-04 |
| SPPL2A | ENSG00000138600 | 1,47 | 9,0E-04 |
| UNK | ENSG00000132478 | 1,47 | 1,2E-03 |
| ZFAND6 | ENSG00000086666 | 1,47 | 1,2E-03 |
| HMGA2 | ENSG00000149948 | 1,47 | 7,2E-04 |
| TM9SF2 | ENSG00000125304 | 1,47 | 6,0E-04 |
| LPAR3 | ENSG00000171517 | 1,47 | 2,3E-03 |
| INPP4A | ENSG00000040933 | 1,47 | 2,6E-03 |
| CDS1 | ENSG00000163624 | 1,47 | 3,9E-04 |
| SRPRB | ENSG00000144867 | 1,47 | 2,5E-03 |
| PAK2 | ENSG00000180370 | 1,47 | 6,9E-04 |
| GDAP2 | ENSG00000196505 | 1,46 | 3,2E-03 |
| CKAP2 | ENSG00000136108 | 1,46 | 7,4E-04 |
| C12orf29 | ENSG00000133641 | 1,46 | 2,3E-03 |
| BARD1 | ENSG00000138376 | 1,46 | 1,5E-03 |
| CASP8AP2 | ENSG00000118412 | 1,46 | 5,5E-03 |
| PPP3CB | ENSG00000107758 | 1,46 | 2,5E-04 |
| NUP50 | ENSG00000093000 | 1,46 | 4,1E-04 |
| UBE2J1 | ENSG00000198833 | 1,46 | 9,2E-04 |
| RSBN1 | ENSG00000081019 | 1,46 | 7,7E-04 |
| RANBP2 | ENSG00000153201 | 1,46 | 1,2E-03 |
| ZNF398 | ENSG00000197024 | 1,46 | 2,3E-03 |
| COPB1 | ENSG00000129083 | 1,46 | 3,1E-04 |
| SC5D | ENSG00000109929 | 1,46 | 9,9E-04 |
| TIMM17A | ENSG00000134375 | 1,46 | 3,2E-03 |
| EPS8 | ENSG00000151491 | 1,46 | 8,2E-04 |
| GPCPD1 | ENSG00000125772 | 1,46 | 9,2E-04 |
| YIPF6 | ENSG00000181704 | 1,46 | 2,3E-04 |
| KLHL5 | ENSG00000109790 | 1,46 | 2,2E-03 |
| ZFP90 | ENSG00000184939 | 1,45 | 9,4E-04 |
| FIG4 | ENSG00000112367 | 1,45 | 5,9E-03 |
| OSBPL3 | ENSG00000070882 | 1,45 | 9,7E-04 |
| MFAP3L | ENSG00000198948 | 1,45 | 1,7E-03 |
| WDR75 | ENSG00000115368 | 1,45 | 3,8E-03 |
| POGLUT1 | ENSG00000163389 | 1,45 | 4,1E-03 |
| ATP6V1H | ENSG00000047249 | 1,45 | 1,3E-03 |
| TMED2 | ENSG00000086598 | 1,45 | 6,9E-04 |
| CPT1A | ENSG00000110090 | 1,45 | 5,5E-03 |
| OXSM | ENSG00000151093 | 1,45 | 2,0E-03 |
| TRAPPC6B | ENSG00000182400 | 1,45 | 3,1E-03 |
| EXTL2 | ENSG00000162694 | 1,45 | 8,8E-04 |
| CCNA2 | ENSG00000145386 | 1,45 | 1,4E-03 |
| MRS2 | ENSG00000124532 | 1,45 | 6,0E-04 |
| ABCB7 | ENSG00000131269 | 1,45 | 2,3E-03 |
| EFNB2 | ENSG00000125266 | 1,45 | 1,6E-03 |
| TMEM50B | ENSG00000142188 | 1,45 | 1,5E-03 |
| CREG1 | ENSG00000143162 | 1,45 | 4,1E-04 |
| GCSH | ENSG00000140905 | 1,45 | 5,9E-03 |
| KLHL2 | ENSG00000109466 | 1,45 | 5,3E-04 |
| RRM2P3 | ENSG00000214018 | 1,44 | 3,9E-03 |
| PSMC6 | ENSG00000100519 | 1,44 | 4,4E-03 |
| MON1B | ENSG00000103111 | 1,44 | 2,2E-03 |
| C16orf62 | ENSG00000103544 | 1,44 | 1,1E-03 |
| SKP2 | ENSG00000145604 | 1,44 | 4,8E-04 |
| FCHSD2 | ENSG00000137478 | 1,44 | 4,3E-03 |
| RIPK2 | ENSG00000104312 | 1,44 | 4,0E-04 |
| PITPNB | ENSG00000180957 | 1,44 | 9,8E-04 |
| STK17B | ENSG00000081320 | 1,44 | 1,1E-03 |
| CRK | ENSG00000167193 | 1,44 | 1,1E-03 |
| ZNF689 | ENSG00000156853 | 1,44 | 1,8E-03 |
| PKIA | ENSG00000171033 | 1,44 | 4,6E-03 |
| SMEK2 | ENSG00000138041 | 1,44 | 4,6E-04 |
| GXYLT1 | ENSG00000151233 | 1,44 | 1,8E-03 |
| MPHOSPH8 | ENSG00000196199 | 1,44 | 2,6E-03 |
| TRAPPC11 | ENSG00000168538 | 1,44 | 5,5E-03 |
| AGFG1 | ENSG00000173744 | 1,44 | 5,4E-04 |
| LCLAT1 | ENSG00000172954 | 1,43 | 2,9E-03 |
| PHACTR2 | ENSG00000112419 | 1,43 | 2,1E-03 |
| EED | ENSG00000074266 | 1,43 | 2,4E-03 |
| OAT | ENSG00000065154 | 1,43 | 1,5E-03 |
| SAT1 | ENSG00000130066 | 1,43 | 2,5E-03 |
| IMPDH2 | ENSG00000178035 | 1,43 | 2,8E-04 |
| NUDCD1 | ENSG00000120526 | 1,43 | 1,9E-03 |
| SYNJ1 | ENSG00000159082 | 1,43 | 5,2E-03 |
| SPG21 | ENSG00000090487 | 1,43 | 1,2E-03 |
| RBM12 | ENSG00000244462 | 1,43 | 5,9E-04 |
| CRY1 | ENSG00000008405 | 1,43 | 2,9E-03 |
| KIAA1468 | ENSG00000134444 | 1,43 | 3,5E-03 |
| ZBTB26 | ENSG00000171448 | 1,43 | 4,2E-03 |
| PHACTR4 | ENSG00000204138 | 1,43 | 7,2E-04 |
| CRIM1 | ENSG00000150938 | 1,43 | 2,4E-03 |
| DHX40 | ENSG00000108406 | 1,42 | 5,3E-04 |
| CAPRIN1 | ENSG00000135387 | 1,42 | 4,2E-04 |
| MTRR | ENSG00000124275 | 1,42 | 2,9E-03 |
| IKBKAP | ENSG00000070061 | 1,42 | 7,7E-04 |
| DEPDC1B | ENSG00000035499 | 1,42 | 8,6E-04 |
| SMAD2 | ENSG00000175387 | 1,42 | 3,1E-04 |
| C14orf119 | ENSG00000179933 | 1,42 | 9,4E-04 |
| KRR1 | ENSG00000111615 | 1,42 | 2,0E-03 |
| CREBL2 | ENSG00000111269 | 1,42 | 1,8E-03 |
| APPL2 | ENSG00000136044 | 1,42 | 2,8E-03 |
| GBAS | ENSG00000146729 | 1,42 | 3,4E-04 |
| KCTD3 | ENSG00000136636 | 1,42 | 6,7E-04 |
| MOB1A | ENSG00000114978 | 1,42 | 1,0E-03 |
| SUCO | ENSG00000094975 | 1,42 | 2,0E-03 |
| ZMYM6 | ENSG00000163867 | 1,42 | 2,1E-03 |
| USP38 | ENSG00000170185 | 1,42 | 1,8E-03 |
| SERINC3 | ENSG00000132824 | 1,42 | 2,2E-03 |
| APC | ENSG00000134982 | 1,42 | 6,1E-03 |
| RBM27 | ENSG00000091009 | 1,42 | 1,4E-03 |
| GLTSCR1L | ENSG00000112624 | 1,41 | 5,3E-03 |
| DTL | ENSG00000143476 | 1,41 | 8,2E-04 |
| EPB41L5 | ENSG00000115109 | 1,41 | 1,1E-03 |
| SMC2 | ENSG00000136824 | 1,41 | 9,5E-04 |
| IMPACT | ENSG00000154059 | 1,41 | 4,0E-04 |
| VEZT | ENSG00000028203 | 1,41 | 1,1E-03 |
| PEX2 | ENSG00000164751 | 1,41 | 7,1E-04 |
| SUMO1 | ENSG00000116030 | 1,41 | 1,7E-03 |
| MON2 | ENSG00000061987 | 1,41 | 5,6E-03 |
| PPP2R1B | ENSG00000137713 | 1,41 | 2,2E-03 |
| ENAH | ENSG00000154380 | 1,41 | 4,2E-04 |
| RNF19A | ENSG00000034677 | 1,41 | 2,9E-03 |
| HPSE | ENSG00000173083 | 1,41 | 2,6E-03 |
| PPP2CA | ENSG00000113575 | 1,41 | 3,5E-03 |
| CAAP1 | ENSG00000120159 | 1,41 | 2,1E-03 |
| C8orf76 | ENSG00000189376 | 1,41 | 3,7E-03 |
| RAD21 | ENSG00000164754 | 1,40 | 4,6E-04 |
| KPNA1 | ENSG00000114030 | 1,40 | 1,0E-03 |
| RCOR3 | ENSG00000117625 | 1,40 | 9,5E-04 |
| CDC37L1 | ENSG00000106993 | 1,40 | 3,1E-03 |
| VAMP4 | ENSG00000117533 | 1,40 | 3,5E-03 |
| CDC42EP3 | ENSG00000163171 | 1,40 | 9,5E-04 |
| ZBTB38 | ENSG00000177311 | 1,40 | 4,3E-03 |
| IRF2BP2 | ENSG00000168264 | 1,40 | 9,7E-04 |
| RP11-700P18.1 | ENSG00000223559 | 1,40 | 2,9E-03 |
| AUH | ENSG00000148090 | 1,40 | 3,1E-03 |
| REPS1 | ENSG00000135597 | 1,40 | 6,4E-04 |
| MFHAS1 | ENSG00000147324 | 1,40 | 4,2E-03 |
| HPS3 | ENSG00000163755 | 1,40 | 2,5E-03 |
| ZFYVE20 | ENSG00000131381 | 1,40 | 2,0E-03 |
| RPRD1B | ENSG00000101413 | 1,40 | 2,9E-03 |
| UTP23 | ENSG00000147679 | 1,40 | 3,4E-03 |
| IFNAR1 | ENSG00000142166 | 1,40 | 9,2E-04 |
| CDC73 | ENSG00000134371 | 1,40 | 1,0E-03 |
| AFTPH | ENSG00000119844 | 1,40 | 2,6E-03 |
| MAP4K5 | ENSG00000012983 | 1,40 | 1,5E-03 |
| H2AFV | ENSG00000105968 | 1,40 | 4,2E-04 |
| DBR1 | ENSG00000138231 | 1,39 | 5,1E-03 |
| HPS5 | ENSG00000110756 | 1,39 | 5,4E-03 |
| SNRPE | ENSG00000182004 | 1,39 | 1,8E-03 |
| RDX | ENSG00000137710 | 1,39 | 1,0E-03 |
| CNOT10 | ENSG00000182973 | 1,39 | 3,1E-03 |
| PCNX | ENSG00000100731 | 1,39 | 5,3E-03 |
| ORC4 | ENSG00000115947 | 1,39 | 2,7E-03 |
| REST | ENSG00000084093 | 1,39 | 2,8E-03 |
| PPA1 | ENSG00000180817 | 1,39 | 2,0E-03 |
| OCLN | ENSG00000197822 | 1,39 | 5,7E-03 |
| ARHGAP5 | ENSG00000100852 | 1,39 | 8,9E-04 |
| ORC2 | ENSG00000115942 | 1,39 | 1,4E-03 |
| CLDND1 | ENSG00000080822 | 1,39 | 2,8E-04 |
| RNF6 | ENSG00000127870 | 1,39 | 3,1E-03 |
| ZNF440 | ENSG00000171295 | 1,39 | 5,2E-03 |
| C5orf51 | ENSG00000205765 | 1,39 | 3,0E-03 |
| BDP1 | ENSG00000145734 | 1,39 | 3,6E-03 |
| FAM105B | ENSG00000154124 | 1,39 | 4,2E-03 |
| PPP6R3 | ENSG00000110075 | 1,39 | 4,5E-04 |
| HIPK1 | ENSG00000163349 | 1,39 | 3,8E-03 |
| CUL2 | ENSG00000108094 | 1,39 | 8,1E-04 |
| RMND1 | ENSG00000155906 | 1,39 | 2,2E-03 |
| CD47 | ENSG00000196776 | 1,39 | 1,2E-03 |
| TMEM41B | ENSG00000166471 | 1,38 | 1,0E-03 |
| ZNF780A | ENSG00000197782 | 1,38 | 1,4E-03 |
| AC026271.5 | ENSG00000174977 | 1,38 | 2,5E-03 |
| STAT1 | ENSG00000115415 | 1,38 | 1,3E-03 |
| PRKRA | ENSG00000180228 | 1,38 | 8,7E-04 |
| TUG1 | ENSG00000253352 | 1,38 | 1,3E-03 |
| NUDT21 | ENSG00000167005 | 1,38 | 6,2E-04 |
| ELK3 | ENSG00000111145 | 1,38 | 3,3E-04 |
| ITFG1 | ENSG00000129636 | 1,38 | 3,4E-03 |
| ITGB1 | ENSG00000150093 | 1,38 | 7,4E-04 |
| CNEP1R1 | ENSG00000205423 | 1,38 | 2,2E-03 |
| ARHGAP29 | ENSG00000137962 | 1,38 | 6,4E-04 |
| TMEM66 | ENSG00000133872 | 1,38 | 6,5E-04 |
| RPS6KA3 | ENSG00000177189 | 1,38 | 8,7E-04 |
| PCCB | ENSG00000114054 | 1,38 | 8,0E-04 |
| GLT8D1 | ENSG00000016864 | 1,38 | 1,0E-03 |
| PCYOX1 | ENSG00000116005 | 1,37 | 2,1E-03 |
| IPO8 | ENSG00000133704 | 1,37 | 2,5E-03 |
| SND1 | ENSG00000197157 | 1,37 | 2,3E-03 |
| STXBP4 | ENSG00000166263 | 1,37 | 4,0E-03 |
| RBM26 | ENSG00000139746 | 1,37 | 1,8E-03 |
| UFM1 | ENSG00000120686 | 1,37 | 6,5E-04 |
| EID1 | ENSG00000255302 | 1,37 | 5,2E-03 |
| CBX1 | ENSG00000108468 | 1,37 | 2,0E-03 |
| ALG6 | ENSG00000088035 | 1,37 | 2,9E-03 |
| RSPRY1 | ENSG00000159579 | 1,37 | 1,4E-03 |
| SPAG9 | ENSG00000008294 | 1,37 | 1,2E-03 |
| UGDH | ENSG00000109814 | 1,37 | 7,5E-04 |
| ADD3 | ENSG00000148700 | 1,37 | 3,4E-03 |
| TMED10 | ENSG00000170348 | 1,37 | 5,1E-04 |
| DDX21 | ENSG00000165732 | 1,37 | 1,3E-03 |
| MMS22L | ENSG00000146263 | 1,37 | 1,5E-03 |
| FDX1 | ENSG00000137714 | 1,37 | 2,4E-03 |
| BTG3 | ENSG00000154640 | 1,37 | 3,5E-03 |
| ABHD3 | ENSG00000158201 | 1,37 | 1,6E-03 |
| C12orf66 | ENSG00000174206 | 1,37 | 1,6E-03 |
| CCDC6 | ENSG00000108091 | 1,37 | 1,8E-03 |
| RAC1 | ENSG00000136238 | 1,37 | 1,2E-03 |
| NARS | ENSG00000134440 | 1,37 | 7,2E-04 |
| RAI14 | ENSG00000039560 | 1,36 | 2,5E-03 |
| ASCC3 | ENSG00000112249 | 1,36 | 2,8E-03 |
| ZFAND5 | ENSG00000107372 | 1,36 | 2,3E-03 |
| SLC25A13 | ENSG00000004864 | 1,36 | 4,2E-03 |
| GSPT1 | ENSG00000103342 | 1,36 | 2,4E-04 |
| TRIM69 | ENSG00000185880 | 1,36 | 2,2E-03 |
| ZNHIT6 | ENSG00000117174 | 1,36 | 5,2E-03 |
| MTHFD2 | ENSG00000065911 | 1,36 | 6,7E-04 |
| TOR1AIP1 | ENSG00000143337 | 1,36 | 2,2E-03 |
| LCORL | ENSG00000178177 | 1,36 | 4,0E-03 |
| LTA4H | ENSG00000111144 | 1,36 | 1,8E-03 |
| CPS1 | ENSG00000021826 | 1,36 | 3,5E-03 |
| U2SURP | ENSG00000163714 | 1,36 | 1,2E-03 |
| YWHAB | ENSG00000166913 | 1,36 | 1,0E-03 |
| FBXO33 | ENSG00000165355 | 1,36 | 5,8E-03 |
| PDCD6IP | ENSG00000170248 | 1,36 | 1,2E-03 |
| SSR3 | ENSG00000114850 | 1,36 | 7,2E-04 |
| PNPLA8 | ENSG00000135241 | 1,36 | 4,3E-03 |
| TMEM192 | ENSG00000170088 | 1,35 | 4,6E-03 |
| INTS8 | ENSG00000164941 | 1,35 | 8,7E-04 |
| CHD9 | ENSG00000177200 | 1,35 | 3,7E-03 |
| GOLPH3 | ENSG00000113384 | 1,35 | 3,7E-03 |
| PRKAG2 | ENSG00000106617 | 1,35 | 1,8E-03 |
| RRAS2 | ENSG00000133818 | 1,35 | 2,9E-03 |
| CHORDC1 | ENSG00000110172 | 1,35 | 5,9E-03 |
| PREPL | ENSG00000138078 | 1,35 | 1,1E-03 |
| NUPL2 | ENSG00000136243 | 1,35 | 2,3E-03 |
| YWHAZP2 | ENSG00000213236 | 1,35 | 2,2E-03 |
| TSPAN6 | ENSG00000000003 | 1,35 | 1,5E-03 |
| AGL | ENSG00000162688 | 1,35 | 1,4E-03 |
| NAA15 | ENSG00000164134 | 1,35 | 8,3E-04 |
| TMOD3 | ENSG00000138594 | 1,35 | 2,2E-03 |
| ANKIB1 | ENSG00000001629 | 1,35 | 1,7E-03 |
| PAM | ENSG00000145730 | 1,35 | 1,3E-03 |
| ANKRD13A | ENSG00000076513 | 1,35 | 2,8E-03 |
| NPEPPS | ENSG00000141279 | 1,35 | 1,2E-03 |
| CCNY | ENSG00000108100 | 1,35 | 6,4E-04 |
| DUS4L | ENSG00000105865 | 1,35 | 6,0E-03 |
| CELF1 | ENSG00000149187 | 1,34 | 3,7E-03 |
| CD46 | ENSG00000117335 | 1,34 | 7,7E-04 |
| DNAJC16 | ENSG00000116138 | 1,34 | 4,2E-03 |
| RHOQ | ENSG00000119729 | 1,34 | 1,0E-03 |
| NIPA1 | ENSG00000170113 | 1,34 | 7,2E-04 |
| TMEM144 | ENSG00000164124 | 1,34 | 2,4E-03 |
| NKIRAS1 | ENSG00000197885 | 1,34 | 3,8E-03 |
| BNIP2 | ENSG00000140299 | 1,34 | 1,6E-03 |
| PTENP1 | ENSG00000237984 | 1,34 | 1,8E-03 |
| TMEM209 | ENSG00000146842 | 1,34 | 2,2E-03 |
| MOB4 | ENSG00000115540 | 1,34 | 2,8E-03 |
| SATB2 | ENSG00000119042 | 1,34 | 2,5E-03 |
| SIKE1 | ENSG00000052723 | 1,34 | 1,7E-03 |
| CENPL | ENSG00000120334 | 1,34 | 5,1E-03 |
| FBXO5 | ENSG00000112029 | 1,34 | 2,4E-03 |
| PPP1CC | ENSG00000186298 | 1,34 | 4,4E-04 |
| CDC23 | ENSG00000094880 | 1,34 | 2,7E-03 |
| H2AFY2 | ENSG00000099284 | 1,34 | 1,7E-03 |
| EDEM3 | ENSG00000116406 | 1,34 | 3,7E-03 |
| PTCD3 | ENSG00000132300 | 1,34 | 2,3E-03 |
| UBXN4 | ENSG00000144224 | 1,34 | 1,1E-03 |
| TMF1 | ENSG00000144747 | 1,33 | 2,2E-03 |
| FAM49B | ENSG00000153310 | 1,33 | 1,8E-03 |
| C1GALT1C1 | ENSG00000171155 | 1,33 | 4,0E-03 |
| SERAC1 | ENSG00000122335 | 1,33 | 4,2E-03 |
| FXR1 | ENSG00000114416 | 1,33 | 1,2E-03 |
| ECHDC1 | ENSG00000093144 | 1,33 | 3,3E-03 |
| SNAPC3 | ENSG00000164975 | 1,33 | 1,0E-03 |
| ZNF627 | ENSG00000198551 | 1,33 | 3,3E-03 |
| SGMS1 | ENSG00000198964 | 1,33 | 4,2E-03 |
| PABPC3 | ENSG00000151846 | 1,33 | 7,0E-04 |
| PCMTD2 | ENSG00000203880 | 1,33 | 3,9E-03 |
| DCUN1D5 | ENSG00000137692 | 1,33 | 3,9E-03 |
| POLR1B | ENSG00000125630 | 1,33 | 1,9E-03 |
| GOLGA7 | ENSG00000147533 | 1,33 | 1,3E-03 |
| HADHB | ENSG00000138029 | 1,33 | 3,0E-03 |
| ZNF451 | ENSG00000112200 | 1,33 | 1,2E-03 |
| MAPK9 | ENSG00000050748 | 1,33 | 6,7E-04 |
| SNRPEP4 | ENSG00000233270 | 1,33 | 2,6E-03 |
| MARCH5 | ENSG00000198060 | 1,33 | 1,1E-03 |
| CAPZA2 | ENSG00000198898 | 1,33 | 3,5E-03 |
| KDM6A | ENSG00000147050 | 1,33 | 3,6E-03 |
| SMYD2 | ENSG00000143499 | 1,32 | 1,7E-03 |
| RANBP6 | ENSG00000137040 | 1,32 | 5,4E-03 |
| RFFL | ENSG00000092871 | 1,32 | 1,3E-03 |
| TPMT | ENSG00000137364 | 1,32 | 1,5E-03 |
| ANTXR1 | ENSG00000169604 | 1,32 | 2,1E-03 |
| SLC16A1 | ENSG00000155380 | 1,32 | 1,3E-03 |
| RP11-333E13.2 | ENSG00000250568 | 1,32 | 4,5E-03 |
| PROSC | ENSG00000147471 | 1,32 | 9,4E-04 |
| SS18L1 | ENSG00000184402 | 1,32 | 2,4E-03 |
| PSMD7 | ENSG00000103035 | 1,32 | 3,0E-03 |
| IGF2BP2 | ENSG00000073792 | 1,32 | 1,5E-03 |
| INTS7 | ENSG00000143493 | 1,32 | 1,5E-03 |
| LGMN | ENSG00000100600 | 1,32 | 2,9E-03 |
| AKAP10 | ENSG00000108599 | 1,32 | 2,8E-03 |
| CSTF2T | ENSG00000177613 | 1,32 | 3,1E-03 |
| MRPL42 | ENSG00000198015 | 1,32 | 4,2E-03 |
| USO1 | ENSG00000138768 | 1,32 | 1,4E-03 |
| CNOT2 | ENSG00000111596 | 1,32 | 1,4E-03 |
| CCDC109B | ENSG00000005059 | 1,32 | 9,4E-04 |
| ZNF639 | ENSG00000121864 | 1,32 | 4,3E-03 |
| FNBP1L | ENSG00000137942 | 1,31 | 2,8E-03 |
| RAD23B | ENSG00000119318 | 1,31 | 2,6E-03 |
| ZBTB1 | ENSG00000126804 | 1,31 | 5,8E-03 |
| MEST | ENSG00000106484 | 1,31 | 1,7E-03 |
| SH3D19 | ENSG00000109686 | 1,31 | 2,9E-03 |
| EXOC6 | ENSG00000138190 | 1,31 | 3,5E-03 |
| RP11-551L14.1 | ENSG00000177359 | 1,31 | 2,2E-03 |
| NRBF2 | ENSG00000148572 | 1,31 | 3,9E-03 |
| ATP6V0A2 | ENSG00000185344 | 1,31 | 3,1E-03 |
| PQLC3 | ENSG00000162976 | 1,31 | 2,5E-03 |
| SENP2 | ENSG00000163904 | 1,31 | 1,9E-03 |
| TMEM245 | ENSG00000106771 | 1,31 | 2,5E-03 |
| SEC24D | ENSG00000150961 | 1,31 | 5,3E-03 |
| TMEM254 | ENSG00000133678 | 1,31 | 3,7E-03 |
| MAK16 | ENSG00000198042 | 1,31 | 3,1E-03 |
| SPATA13 | ENSG00000182957 | 1,31 | 4,3E-03 |
| ALKBH1 | ENSG00000100601 | 1,31 | 3,8E-03 |
| NUP153 | ENSG00000124789 | 1,31 | 2,7E-03 |
| TRMT13 | ENSG00000122435 | 1,30 | 4,5E-03 |
| NEK4 | ENSG00000114904 | 1,30 | 2,9E-03 |
| UBE2G1 | ENSG00000132388 | 1,30 | 1,0E-03 |
| USP24 | ENSG00000162402 | 1,30 | 5,8E-03 |
| SH3RF1 | ENSG00000154447 | 1,30 | 9,4E-04 |
| TXNRD1 | ENSG00000198431 | 1,30 | 7,9E-04 |
| GBE1 | ENSG00000114480 | 1,30 | 2,2E-03 |
| PSAT1 | ENSG00000135069 | 1,30 | 1,5E-03 |
| PELI1 | ENSG00000197329 | 1,30 | 4,6E-03 |
| DIMT1 | ENSG00000086189 | 1,30 | 4,2E-03 |
| PTPRK | ENSG00000152894 | 1,30 | 3,2E-03 |
| RABL3 | ENSG00000144840 | 1,30 | 2,8E-03 |
| ADO | ENSG00000181915 | 1,30 | 2,5E-03 |
| PTGES3 | ENSG00000110958 | 1,30 | 2,4E-03 |
| DCBLD2 | ENSG00000057019 | 1,30 | 6,2E-04 |
| RMDN1 | ENSG00000176623 | 1,30 | 1,8E-03 |
| ZC3HAV1 | ENSG00000105939 | 1,30 | 5,9E-03 |
| PSME4 | ENSG00000068878 | 1,30 | 3,9E-03 |
| IARS | ENSG00000196305 | 1,29 | 1,2E-03 |
| GABARAPL2 | ENSG00000034713 | 1,29 | 2,2E-03 |
| YME1L1 | ENSG00000136758 | 1,29 | 1,1E-03 |
| DNM1L | ENSG00000087470 | 1,29 | 1,3E-03 |
| TTC39C | ENSG00000168234 | 1,29 | 3,0E-03 |
| ZRANB1 | ENSG00000019995 | 1,29 | 2,1E-03 |
| CTC-308K20.3 | ENSG00000253785 | 1,29 | 3,9E-03 |
| SNX9 | ENSG00000130340 | 1,29 | 2,1E-03 |
| REEP3 | ENSG00000165476 | 1,29 | 1,5E-03 |
| DERL1 | ENSG00000136986 | 1,29 | 2,2E-03 |
| HLTF | ENSG00000071794 | 1,29 | 2,1E-03 |
| STAU1 | ENSG00000124214 | 1,29 | 1,5E-03 |
| PMPCB | ENSG00000105819 | 1,29 | 1,5E-03 |
| YAP1 | ENSG00000137693 | 1,29 | 4,0E-03 |
| RNF139 | ENSG00000170881 | 1,29 | 2,8E-03 |
| AREL1 | ENSG00000119682 | 1,29 | 4,4E-03 |
| NEDD1 | ENSG00000139350 | 1,29 | 3,1E-03 |
| MCM4 | ENSG00000104738 | 1,29 | 1,2E-03 |
| CD99P1 | ENSG00000223773 | 1,28 | 3,1E-03 |
| OSTC | ENSG00000198856 | 1,28 | 4,4E-03 |
| ZFAND1 | ENSG00000104231 | 1,28 | 6,8E-04 |
| MTDH | ENSG00000147649 | 1,28 | 3,9E-03 |
| COPS4 | ENSG00000138663 | 1,28 | 6,2E-03 |
| NIN | ENSG00000100503 | 1,28 | 3,8E-03 |
| F11R | ENSG00000158769 | 1,28 | 2,2E-03 |
| GPR160 | ENSG00000173890 | 1,28 | 4,3E-03 |
| F2RL1 | ENSG00000164251 | 1,27 | 9,0E-04 |
| MED30 | ENSG00000164758 | 1,27 | 4,1E-03 |
| UBE2D3P1 | ENSG00000225022 | 1,27 | 1,7E-03 |
| CEP78 | ENSG00000148019 | 1,27 | 2,1E-03 |
| FIGNL1 | ENSG00000132436 | 1,27 | 3,0E-03 |
| GPR126 | ENSG00000112414 | 1,27 | 4,3E-03 |
| PSIP1 | ENSG00000164985 | 1,27 | 2,4E-03 |
| ACO1 | ENSG00000122729 | 1,27 | 3,2E-03 |
| PAICS | ENSG00000128050 | 1,27 | 6,5E-04 |
| PLK4 | ENSG00000142731 | 1,27 | 2,4E-03 |
| PTGES3P1 | ENSG00000234518 | 1,27 | 3,6E-03 |
| RTCA | ENSG00000137996 | 1,26 | 3,7E-03 |
| TCAIM | ENSG00000179152 | 1,26 | 1,9E-03 |
| CRLS1 | ENSG00000088766 | 1,26 | 3,3E-03 |
| C18orf25 | ENSG00000152242 | 1,26 | 4,8E-03 |
| ABHD17B | ENSG00000107362 | 1,26 | 3,4E-03 |
| SNX18 | ENSG00000178996 | 1,26 | 4,4E-03 |
| GCFC2 | ENSG00000005436 | 1,26 | 5,4E-03 |
| OPA1 | ENSG00000198836 | 1,26 | 1,4E-03 |
| YWHAQ | ENSG00000134308 | 1,26 | 2,7E-03 |
| SLK | ENSG00000065613 | 1,25 | 2,1E-03 |
| AP3B1 | ENSG00000132842 | 1,25 | 3,6E-03 |
| MCL1 | ENSG00000143384 | 1,25 | 3,3E-03 |
| KBTBD2 | ENSG00000170852 | 1,25 | 2,9E-03 |
| CSDE1 | ENSG00000009307 | 1,25 | 1,7E-03 |
| NUCKS1 | ENSG00000069275 | 1,25 | 1,9E-03 |
| WDR76 | ENSG00000092470 | 1,25 | 2,8E-03 |
| VSIG10 | ENSG00000176834 | 1,25 | 4,5E-03 |
| ZNF280D | ENSG00000137871 | 1,25 | 5,0E-03 |
| ADK | ENSG00000156110 | 1,25 | 3,0E-03 |
| EIF2S1 | ENSG00000134001 | 1,25 | 5,0E-03 |
| HEATR2 | ENSG00000164818 | 1,25 | 3,8E-03 |
| P4HA1 | ENSG00000122884 | 1,25 | 5,3E-03 |
| MID1 | ENSG00000101871 | 1,25 | 3,4E-03 |
| SRSF10 | ENSG00000188529 | 1,25 | 3,8E-03 |
| ABLIM1 | ENSG00000099204 | 1,25 | 5,4E-03 |
| OXCT1 | ENSG00000083720 | 1,25 | 4,9E-03 |
| HMGB1 | ENSG00000189403 | 1,25 | 4,0E-03 |
| IPO5 | ENSG00000065150 | 1,24 | 1,8E-03 |
| TANK | ENSG00000136560 | 1,24 | 1,7E-03 |
| HSDL1 | ENSG00000103160 | 1,24 | 5,5E-03 |
| BIRC2 | ENSG00000110330 | 1,24 | 3,8E-03 |
| TTC19 | ENSG00000011295 | 1,24 | 5,0E-03 |
| DLD | ENSG00000091140 | 1,24 | 3,8E-03 |
| ITM2B | ENSG00000136156 | 1,24 | 6,2E-03 |
| CLDN12 | ENSG00000157224 | 1,24 | 3,4E-03 |
| USP31 | ENSG00000103404 | 1,24 | 6,1E-03 |
| KLHL7 | ENSG00000122550 | 1,24 | 3,6E-03 |
| MCM8 | ENSG00000125885 | 1,24 | 5,6E-03 |
| DHFR | ENSG00000228716 | 1,24 | 5,0E-03 |
| AASDH | ENSG00000157426 | 1,24 | 5,6E-03 |
| FAM188A | ENSG00000148481 | 1,24 | 3,6E-03 |
| DNAJC10 | ENSG00000077232 | 1,24 | 1,6E-03 |
| MTHFD2L | ENSG00000163738 | 1,24 | 3,1E-03 |
| HELLS | ENSG00000119969 | 1,24 | 1,3E-03 |
| EFCAB14 | ENSG00000159658 | 1,24 | 3,4E-03 |
| GTPBP8 | ENSG00000163607 | 1,24 | 6,1E-03 |
| SEPT2 | ENSG00000168385 | 1,24 | 1,9E-03 |
| XRN2 | ENSG00000088930 | 1,24 | 2,8E-03 |
| CALD1 | ENSG00000122786 | 1,23 | 5,0E-03 |
| SOD2 | ENSG00000112096 | 1,23 | 1,5E-03 |
| GALNT10 | ENSG00000164574 | 1,23 | 5,2E-03 |
| SPOP | ENSG00000121067 | 1,23 | 1,8E-03 |
| COX11 | ENSG00000166260 | 1,23 | 2,7E-03 |
| TRIM33 | ENSG00000197323 | 1,23 | 4,5E-03 |
| PPP2CB | ENSG00000104695 | 1,23 | 3,1E-03 |
| BICD1 | ENSG00000151746 | 1,23 | 4,7E-03 |
| GNB1 | ENSG00000078369 | 1,23 | 1,6E-03 |
| DDX1 | ENSG00000079785 | 1,23 | 3,3E-03 |
| NSMAF | ENSG00000035681 | 1,23 | 5,0E-03 |
| NEIL3 | ENSG00000109674 | 1,23 | 4,2E-03 |
| PBK | ENSG00000168078 | 1,23 | 5,7E-03 |
| CBX3 | ENSG00000122565 | 1,23 | 4,6E-03 |
| GNPAT | ENSG00000116906 | 1,23 | 2,6E-03 |
| COG3 | ENSG00000136152 | 1,23 | 3,3E-03 |
| ANAPC13 | ENSG00000129055 | 1,23 | 4,7E-03 |
| ARL6IP1 | ENSG00000170540 | 1,22 | 4,5E-03 |
| PHF10 | ENSG00000130024 | 1,22 | 4,1E-03 |
| ATXN7 | ENSG00000163635 | 1,22 | 6,3E-03 |
| PLEKHB2 | ENSG00000115762 | 1,22 | 5,9E-03 |
| SLMAP | ENSG00000163681 | 1,22 | 4,1E-03 |
| OSBPL11 | ENSG00000144909 | 1,22 | 6,1E-03 |
| CCNDBP1 | ENSG00000166946 | 1,22 | 3,8E-03 |
| EHD4 | ENSG00000103966 | 1,21 | 2,2E-03 |
| MLF1 | ENSG00000178053 | 1,21 | 1,6E-03 |
| PIAS2 | ENSG00000078043 | 1,21 | 4,1E-03 |
| SH3GLB1 | ENSG00000097033 | 1,21 | 3,0E-03 |
| TAB2 | ENSG00000055208 | 1,21 | 2,1E-03 |
| EML4 | ENSG00000143924 | 1,21 | 5,4E-03 |
| RP11-43F13.1 | ENSG00000188002 | 1,21 | 6,1E-03 |
| RFWD3 | ENSG00000168411 | 1,21 | 3,6E-03 |
| OMA1 | ENSG00000162600 | 1,21 | 5,4E-03 |
| ZNF252P | ENSG00000196922 | 1,21 | 5,8E-03 |
| NPTN | ENSG00000156642 | 1,21 | 3,3E-03 |
| RHBDD1 | ENSG00000144468 | 1,21 | 4,9E-03 |
| LONP2 | ENSG00000102910 | 1,20 | 4,7E-03 |
| PKP2 | ENSG00000057294 | 1,20 | 4,9E-03 |
| DCLRE1A | ENSG00000198924 | 1,20 | 5,8E-03 |
| DDHD1 | ENSG00000100523 | 1,20 | 5,8E-03 |
| WAPAL | ENSG00000062650 | 1,20 | 3,4E-03 |
| RHOT1 | ENSG00000126858 | 1,20 | 2,3E-03 |
| ACVR1 | ENSG00000115170 | 1,20 | 4,5E-03 |
| RABGGTB | ENSG00000137955 | 1,20 | 5,7E-03 |
| SHCBP1 | ENSG00000171241 | 1,20 | 6,0E-03 |
| ISOC1 | ENSG00000066583 | 1,20 | 4,8E-03 |
| CLN5 | ENSG00000102805 | 1,20 | 4,9E-03 |
| SMARCAD1 | ENSG00000163104 | 1,20 | 5,1E-03 |
| HMG20A | ENSG00000140382 | 1,20 | 3,2E-03 |
| FBXW11 | ENSG00000072803 | 1,20 | 4,2E-03 |
| KHDRBS1 | ENSG00000121774 | 1,20 | 1,8E-03 |
| TRAPPC8 | ENSG00000153339 | 1,20 | 5,6E-03 |
| TSG101 | ENSG00000074319 | 1,20 | 5,0E-03 |
| RWDD3 | ENSG00000122481 | 1,19 | 5,5E-03 |
| TMEM216 | ENSG00000187049 | 1,19 | 5,5E-03 |
| HDAC2 | ENSG00000196591 | 1,19 | 4,2E-03 |
| BAG5 | ENSG00000166170 | 1,19 | 5,8E-03 |
| HBS1L | ENSG00000112339 | 1,19 | 2,5E-03 |
| FAF2 | ENSG00000113194 | 1,19 | 4,6E-03 |
| PLAA | ENSG00000137055 | 1,19 | 3,9E-03 |
| AKIRIN1 | ENSG00000174574 | 1,19 | 4,3E-03 |
| LDLRAD3 | ENSG00000179241 | 1,19 | 5,2E-03 |
| ANAPC4 | ENSG00000053900 | 1,19 | 6,1E-03 |
| EIF4B | ENSG00000063046 | 1,18 | 3,2E-03 |
| DBF4 | ENSG00000006634 | 1,18 | 6,4E-03 |
| ABRACL | ENSG00000146386 | 1,18 | 2,4E-03 |
| SUB1 | ENSG00000113387 | 1,18 | 6,0E-03 |
| CHMP2B | ENSG00000083937 | 1,18 | 4,3E-03 |
| METAP1 | ENSG00000164024 | 1,18 | 3,1E-03 |
| ANKRD32 | ENSG00000133302 | 1,18 | 5,7E-03 |
| H3F3B | ENSG00000132475 | 1,18 | 4,1E-03 |
| XPOT | ENSG00000184575 | 1,18 | 3,5E-03 |
| HSPH1 | ENSG00000120694 | 1,18 | 2,9E-03 |
| TUBA1A | ENSG00000167552 | 1,17 | 3,1E-03 |
| MAPK1IP1L | ENSG00000168175 | 1,17 | 4,3E-03 |
| DYNLT3 | ENSG00000165169 | 1,17 | 4,8E-03 |
| CASC4 | ENSG00000166734 | 1,17 | 3,9E-03 |
| KIAA0947 | ENSG00000164151 | 1,17 | 5,9E-03 |
| BUB1 | ENSG00000169679 | 1,17 | 4,2E-03 |
| CPSF6 | ENSG00000111605 | 1,17 | 3,9E-03 |
| SERPINE2 | ENSG00000135919 | 1,17 | 4,6E-03 |
| WASF1 | ENSG00000112290 | 1,17 | 2,7E-03 |
| CXXC5 | ENSG00000171604 | 1,17 | 3,4E-03 |
| PABPC1P4 | ENSG00000255642 | 1,16 | 5,5E-03 |
| CDC42EP4 | ENSG00000179604 | 1,16 | 6,1E-03 |
| RAD1 | ENSG00000113456 | 1,16 | 4,3E-03 |
| TMEM165 | ENSG00000134851 | 1,16 | 5,6E-03 |
| ATXN10 | ENSG00000130638 | 1,15 | 6,2E-03 |
| LARP4B | ENSG00000107929 | 1,15 | 3,1E-03 |
| ZNF138 | ENSG00000197008 | 1,15 | 6,2E-03 |
| HADH | ENSG00000138796 | 1,15 | 5,7E-03 |
| PABPC1 | ENSG00000070756 | 1,14 | 3,1E-03 |
| C7orf73 | ENSG00000243317 | 1,14 | 4,4E-03 |
| RDH11 | ENSG00000072042 | 1,14 | 6,1E-03 |
| NGLY1 | ENSG00000151092 | 1,14 | 5,7E-03 |
| NT5DC1 | ENSG00000178425 | 1,14 | 3,2E-03 |
| VPS36 | ENSG00000136100 | 1,14 | 3,8E-03 |
| RBMXL1 | ENSG00000213516 | 1,13 | 5,2E-03 |
| DCTN4 | ENSG00000132912 | 1,13 | 3,5E-03 |
| SNX3 | ENSG00000112335 | 1,13 | 3,7E-03 |
| AMZ2 | ENSG00000196704 | 1,13 | 4,3E-03 |
| TMEM33 | ENSG00000109133 | 1,13 | 3,8E-03 |
| SCP2 | ENSG00000116171 | 1,13 | 6,3E-03 |
| CANX | ENSG00000127022 | 1,12 | 5,1E-03 |
| LEPROTL1 | ENSG00000104660 | 1,12 | 4,1E-03 |
| SEC23IP | ENSG00000107651 | 1,11 | 4,6E-03 |
| GMPS | ENSG00000163655 | 1,11 | 3,9E-03 |
| ZNF655 | ENSG00000197343 | 1,11 | 5,6E-03 |
| TGFA | ENSG00000163235 | 1,11 | 4,9E-03 |
| HTATIP2 | ENSG00000109854 | 1,10 | 4,3E-03 |
| C12orf75 | ENSG00000235162 | 1,10 | 5,4E-03 |
| TRIM37 | ENSG00000108395 | 1,10 | 5,5E-03 |
| PPARG | ENSG00000132170 | 1,09 | 5,7E-03 |
| MPHOSPH9 | ENSG00000051825 | 1,09 | 6,0E-03 |
| GLO1 | ENSG00000124767 | 1,08 | 5,0E-03 |
| PHLDB2 | ENSG00000144824 | 1,08 | 4,5E-03 |
| PTK2 | ENSG00000169398 | 1,07 | 5,6E-03 |
| TOMM20 | ENSG00000173726 | 1,07 | 3,4E-03 |
| ME1 | ENSG00000065833 | 1,07 | 6,2E-03 |
| SERP1 | ENSG00000120742 | 1,07 | 5,6E-03 |
| DPP8 | ENSG00000074603 | 1,07 | 6,3E-03 |
| GRSF1 | ENSG00000132463 | 1,06 | 4,9E-03 |
| CKS2 | ENSG00000123975 | 1,06 | 2,7E-03 |
| SAV1 | ENSG00000151748 | 1,05 | 6,1E-03 |
| EIF4G2 | ENSG00000110321 | 1,03 | 4,3E-03 |
