# Supplementary material for: Genome-wide identification of Wig-1 mRNA targets by RIP-Seq analysis
Source: Oncotarget. 2015 Dec 11;7(2):1895–911. doi: 10.18632/oncotarget.6557 (PMC4811505; doi:10.18632/oncotarget.6557)
Supplement: Supplementary file 3 [file oncotarget-07-1895-s003.docx]

| Supplementary Table S3: List of the 354 Wig-1-bound RNAs in Saos-2 cells. | | | |
| --- | --- | --- | --- |
|  |  |  |  |
| **Gene symbol** | **Ensembl ID** | **logFC** | **p.value** |
| ANKRD7 | ENSG00000106013 | 3,12 | 6,1E-04 |
| PHOSPHO2 | ENSG00000144362 | 3,05 | 1,1E-03 |
| C14orf129 | ENSG00000100744 | 2,89 | 2,7E-06 |
| RP11-307L3.2 | ENSG00000233846 | 2,65 | 3,0E-06 |
| LYPLA1 | ENSG00000120992 | 2,63 | 2,4E-04 |
| STARD3NL | ENSG00000010270 | 2,59 | 6,4E-05 |
| SCOC | ENSG00000153130 | 2,58 | 1,1E-04 |
| DPH3 | ENSG00000154813 | 2,57 | 3,7E-05 |
| GOLT1B | ENSG00000111711 | 2,56 | 1,1E-04 |
| MOSPD1 | ENSG00000101928 | 2,55 | 1,2E-04 |
| RP1-145M24.1 | ENSG00000254708 | 2,55 | 9,5E-04 |
| C13orf27 | ENSG00000151287 | 2,54 | 8,1E-05 |
| CTC-534A2.2 | ENSG00000253251 | 2,53 | 7,6E-04 |
| SLC25A16 | ENSG00000122912 | 2,53 | 8,4E-05 |
| MTHFD2L | ENSG00000163738 | 2,52 | 1,2E-04 |
| RP11-392O18.1 | ENSG00000198843 | 2,49 | 1,1E-04 |
| CRADD | ENSG00000169372 | 2,48 | 1,6E-04 |
| CHAC2 | ENSG00000143942 | 2,46 | 3,9E-04 |
| MZT1 | ENSG00000204899 | 2,46 | 7,2E-05 |
| CISD2 | ENSG00000145354 | 2,44 | 1,0E-04 |
| RP11-533F5.2 | ENSG00000227040 | 2,42 | 1,4E-04 |
| TXNDC9 | ENSG00000115514 | 2,41 | 2,4E-04 |
| RBM7 | ENSG00000076053 | 2,41 | 1,0E-04 |
| C14orf28 | ENSG00000179476 | 2,40 | 2,9E-04 |
| CYB5R4 | ENSG00000065615 | 2,40 | 2,6E-05 |
| TMEM38B | ENSG00000095209 | 2,40 | 7,6E-05 |
| MRPL42 | ENSG00000198015 | 2,39 | 4,5E-05 |
| CAV2 | ENSG00000105971 | 2,39 | 2,6E-05 |
| SGPP1 | ENSG00000126821 | 2,38 | 5,8E-05 |
| LRRIQ3 | ENSG00000162620 | 2,38 | 3,7E-04 |
| C6orf211 | ENSG00000146476 | 2,38 | 4,5E-05 |
| YIPF4 | ENSG00000119820 | 2,36 | 8,3E-06 |
| COMMD8 | ENSG00000169019 | 2,36 | 3,2E-04 |
| TEX9 | ENSG00000151575 | 2,36 | 2,0E-03 |
| AP1S3 | ENSG00000152056 | 2,35 | 3,2E-04 |
| DRAM2 | ENSG00000156171 | 2,32 | 8,1E-05 |
| NDUFA5 | ENSG00000128609 | 2,31 | 8,6E-05 |
| SLC35A3 | ENSG00000117620 | 2,30 | 2,6E-05 |
| C12orf29 | ENSG00000133641 | 2,29 | 9,0E-05 |
| RP11-307P22.1 | ENSG00000258445 | 2,28 | 3,7E-05 |
| NDUFB5 | ENSG00000136521 | 2,28 | 8,1E-05 |
| EEF1E1 | ENSG00000124802 | 2,27 | 2,8E-04 |
| C9orf46 | ENSG00000107020 | 2,27 | 1,5E-04 |
| MMD | ENSG00000108960 | 2,26 | 1,2E-04 |
| RNF138 | ENSG00000134758 | 2,24 | 3,9E-04 |
| ZNF277 | ENSG00000198839 | 2,23 | 8,4E-05 |
| RP4-604K5.1 | ENSG00000183291 | 2,23 | 1,2E-04 |
| NUP35 | ENSG00000163002 | 2,22 | 3,5E-05 |
| C6orf115 | ENSG00000146386 | 2,20 | 2,4E-05 |
| MTMR6 | ENSG00000139505 | 2,19 | 2,9E-05 |
| LIN9 | ENSG00000183814 | 2,19 | 1,4E-04 |
| ZDHHC13 | ENSG00000177054 | 2,18 | 4,3E-04 |
| UBE2B | ENSG00000119048 | 2,17 | 1,4E-04 |
| AMD1 | ENSG00000123505 | 2,17 | 2,6E-05 |
| FPGT | ENSG00000254685 | 2,17 | 8,2E-05 |
| C5orf44 | ENSG00000113597 | 2,16 | 1,4E-04 |
| NIPSNAP3A | ENSG00000136783 | 2,15 | 6,6E-05 |
| MAD2L1 | ENSG00000164109 | 2,15 | 6,6E-04 |
| METTL10 | ENSG00000203791 | 2,15 | 9,1E-04 |
| NECAB1 | ENSG00000123119 | 2,15 | 5,1E-04 |
| CCNG1 | ENSG00000113328 | 2,14 | 5,2E-05 |
| GMFB | ENSG00000197045 | 2,14 | 2,1E-04 |
| TWF1 | ENSG00000151239 | 2,14 | 1,2E-04 |
| SAR1B | ENSG00000152700 | 2,14 | 9,0E-04 |
| PEX3 | ENSG00000034693 | 2,13 | 1,2E-04 |
| TFB2M | ENSG00000162851 | 2,13 | 2,0E-03 |
| LAMTOR3 | ENSG00000109270 | 2,13 | 6,9E-04 |
| ARL5A | ENSG00000162980 | 2,12 | 7,6E-05 |
| GPR180 | ENSG00000152749 | 2,12 | 1,5E-04 |
| GIN1 | ENSG00000145723 | 2,12 | 1,7E-03 |
| ORMDL1 | ENSG00000128699 | 2,11 | 6,9E-04 |
| NUP37 | ENSG00000075188 | 2,10 | 1,0E-04 |
| RP11-320A16.1 | ENSG00000261549 | 2,10 | 4,1E-04 |
| AC011533.1 | ENSG00000233270 | 2,10 | 3,2E-04 |
| ALG13 | ENSG00000101901 | 2,09 | 1,5E-04 |
| RNFT1 | ENSG00000189050 | 2,08 | 2,6E-04 |
| YAF2 | ENSG00000015153 | 2,08 | 1,4E-03 |
| ASTE1 | ENSG00000034533 | 2,07 | 3,8E-04 |
| XRCC4 | ENSG00000152422 | 2,07 | 2,8E-03 |
| GEMIN2 | ENSG00000092208 | 2,06 | 3,6E-04 |
| ANAPC13 | ENSG00000129055 | 2,05 | 7,0E-05 |
| RP11-166D19.1 | ENSG00000255248 | 2,05 | 1,8E-03 |
| C5orf30 | ENSG00000181751 | 2,04 | 3,6E-04 |
| CCDC126 | ENSG00000169193 | 2,04 | 2,4E-04 |
| TMEM135 | ENSG00000166575 | 2,04 | 5,3E-04 |
| TTC33 | ENSG00000113638 | 2,03 | 1,6E-04 |
| EIF5A2 | ENSG00000163577 | 2,03 | 3,2E-04 |
| MRPL50 | ENSG00000136897 | 2,03 | 1,4E-03 |
| SLC25A32 | ENSG00000164933 | 2,02 | 1,4E-04 |
| RP11-421L21.3 | ENSG00000233184 | 2,02 | 2,7E-04 |
| CNOT8 | ENSG00000155508 | 2,02 | 1,5E-04 |
| SCML1 | ENSG00000047634 | 2,02 | 1,6E-04 |
| ATG12 | ENSG00000145782 | 2,02 | 9,6E-04 |
| TMEM188 | ENSG00000205423 | 2,01 | 2,9E-04 |
| DPM1 | ENSG00000000419 | 2,01 | 5,3E-04 |
| FDX1 | ENSG00000137714 | 2,01 | 8,2E-05 |
| FOPNL | ENSG00000133393 | 2,01 | 3,0E-04 |
| OSGEPL1 | ENSG00000128694 | 2,00 | 7,5E-04 |
| NDFIP2 | ENSG00000102471 | 2,00 | 3,8E-04 |
| RRM2B | ENSG00000048392 | 2,00 | 2,0E-04 |
| TMEM117 | ENSG00000139173 | 2,00 | 9,6E-04 |
| TMEM68 | ENSG00000167904 | 2,00 | 4,2E-04 |
| CAPZA2 | ENSG00000198898 | 1,99 | 1,5E-04 |
| ANKRD46 | ENSG00000186106 | 1,99 | 6,9E-04 |
| ORC4 | ENSG00000115947 | 1,99 | 9,5E-05 |
| MOB4 | ENSG00000115540 | 1,99 | 3,4E-04 |
| RFK | ENSG00000135002 | 1,99 | 1,4E-04 |
| SLC39A8 | ENSG00000138821 | 1,98 | 2,6E-05 |
| C10orf32 | ENSG00000166275 | 1,97 | 1,9E-04 |
| PPA2 | ENSG00000138777 | 1,97 | 3,5E-04 |
| METTL9 | ENSG00000197006 | 1,96 | 4,9E-05 |
| CNOT7 | ENSG00000198791 | 1,96 | 2,7E-04 |
| STARD4 | ENSG00000164211 | 1,95 | 3,9E-05 |
| SNX7 | ENSG00000162627 | 1,95 | 6,4E-05 |
| BZW1P2 | ENSG00000198406 | 1,95 | 3,8E-04 |
| TSPAN6 | ENSG00000000003 | 1,95 | 8,4E-04 |
| RWDD4 | ENSG00000182552 | 1,95 | 9,5E-04 |
| ATG5 | ENSG00000057663 | 1,94 | 4,8E-04 |
| ABHD13 | ENSG00000139826 | 1,94 | 1,8E-04 |
| STRADB | ENSG00000082146 | 1,94 | 3,0E-04 |
| GTF2B | ENSG00000137947 | 1,94 | 4,2E-04 |
| FAM188A | ENSG00000148481 | 1,94 | 1,4E-04 |
| TRUB1 | ENSG00000165832 | 1,93 | 4,3E-04 |
| MGAT4A | ENSG00000071073 | 1,93 | 9,5E-05 |
| HSD17B11 | ENSG00000198189 | 1,93 | 4,5E-04 |
| DCK | ENSG00000156136 | 1,93 | 1,1E-03 |
| ANAPC10 | ENSG00000164162 | 1,93 | 1,8E-03 |
| ZCCHC10 | ENSG00000155329 | 1,93 | 9,0E-04 |
| IFT52 | ENSG00000101052 | 1,93 | 6,5E-04 |
| C11orf54 | ENSG00000182919 | 1,92 | 4,4E-04 |
| PLEKHF2 | ENSG00000175895 | 1,91 | 3,7E-04 |
| ARMC1 | ENSG00000104442 | 1,91 | 3,4E-04 |
| C14orf126 | ENSG00000129480 | 1,91 | 1,9E-03 |
| ACTR6 | ENSG00000075089 | 1,90 | 1,3E-03 |
| ELMOD2 | ENSG00000179387 | 1,90 | 1,0E-03 |
| PPIL3 | ENSG00000240344 | 1,90 | 6,9E-04 |
| NDUFAF4 | ENSG00000123545 | 1,90 | 8,5E-04 |
| THAP2 | ENSG00000173451 | 1,90 | 2,7E-04 |
| ERCC8 | ENSG00000049167 | 1,89 | 1,2E-03 |
| RMI1 | ENSG00000178966 | 1,89 | 8,1E-05 |
| RP11-444I9.2 | ENSG00000234369 | 1,89 | 1,1E-03 |
| VAMP7 | ENSG00000124333 | 1,88 | 1,9E-04 |
| NMD3 | ENSG00000169251 | 1,88 | 1,4E-04 |
| ASF1A | ENSG00000111875 | 1,88 | 2,4E-04 |
| CMPK1 | ENSG00000162368 | 1,87 | 2,5E-05 |
| SEC22C | ENSG00000093183 | 1,87 | 1,7E-04 |
| SLMO2 | ENSG00000101166 | 1,87 | 1,6E-04 |
| SNRPE | ENSG00000182004 | 1,87 | 1,6E-03 |
| ZFAND6 | ENSG00000086666 | 1,86 | 4,3E-04 |
| NUP54 | ENSG00000138750 | 1,86 | 8,1E-04 |
| MRPL47 | ENSG00000136522 | 1,86 | 5,5E-04 |
| DEPDC1 | ENSG00000024526 | 1,85 | 3,8E-04 |
| RHEB | ENSG00000106615 | 1,84 | 3,1E-04 |
| TSNAX | ENSG00000116918 | 1,84 | 1,2E-03 |
| MYNN | ENSG00000085274 | 1,84 | 5,1E-04 |
| TMEM59 | ENSG00000116209 | 1,83 | 2,7E-04 |
| MRPL13 | ENSG00000172172 | 1,83 | 2,1E-03 |
| HSPA13 | ENSG00000155304 | 1,83 | 4,6E-04 |
| RSL24D1 | ENSG00000137876 | 1,83 | 1,9E-03 |
| ARRDC3 | ENSG00000113369 | 1,83 | 5,3E-04 |
| FAM206A | ENSG00000119328 | 1,83 | 2,0E-03 |
| PPP1CB | ENSG00000213639 | 1,83 | 1,3E-04 |
| EIF4E | ENSG00000151247 | 1,83 | 1,3E-03 |
| NCK1 | ENSG00000158092 | 1,82 | 1,2E-03 |
| SUV39H2 | ENSG00000152455 | 1,82 | 2,9E-04 |
| CPNE8 | ENSG00000139117 | 1,81 | 1,2E-03 |
| ASNSD1 | ENSG00000138381 | 1,81 | 3,3E-04 |
| HMGN4 | ENSG00000182952 | 1,81 | 5,3E-05 |
| COPS8 | ENSG00000198612 | 1,81 | 1,5E-03 |
| BMI1 | ENSG00000168283 | 1,81 | 1,7E-04 |
| SEC23A | ENSG00000100934 | 1,80 | 1,4E-04 |
| ELOVL4 | ENSG00000118402 | 1,80 | 1,2E-03 |
| MFSD1 | ENSG00000118855 | 1,80 | 4,2E-04 |
| SNX24 | ENSG00000064652 | 1,79 | 1,2E-03 |
| MIS12 | ENSG00000167842 | 1,79 | 6,4E-04 |
| CFL2 | ENSG00000165410 | 1,79 | 8,0E-04 |
| ABHD10 | ENSG00000144827 | 1,79 | 3,5E-04 |
| DNAJB9 | ENSG00000128590 | 1,79 | 2,0E-03 |
| RNF11 | ENSG00000123091 | 1,79 | 3,8E-05 |
| DUSP11 | ENSG00000144048 | 1,79 | 3,8E-04 |
| PBK | ENSG00000168078 | 1,78 | 2,9E-04 |
| ARL8B | ENSG00000134108 | 1,78 | 4,9E-05 |
| SCP2 | ENSG00000116171 | 1,78 | 1,0E-04 |
| BCAP29 | ENSG00000075790 | 1,78 | 1,8E-04 |
| RAB2A | ENSG00000104388 | 1,77 | 1,4E-04 |
| ZMPSTE24 | ENSG00000084073 | 1,77 | 1,2E-04 |
| C14orf119 | ENSG00000179933 | 1,77 | 3,8E-04 |
| KRCC1 | ENSG00000172086 | 1,77 | 6,9E-04 |
| DYNC2LI1 | ENSG00000138036 | 1,76 | 1,8E-03 |
| TUBD1 | ENSG00000108423 | 1,76 | 1,6E-03 |
| ATF1 | ENSG00000123268 | 1,76 | 8,6E-04 |
| CMTM6 | ENSG00000091317 | 1,76 | 2,3E-04 |
| NEK7 | ENSG00000151414 | 1,76 | 4,0E-04 |
| HIAT1 | ENSG00000156875 | 1,76 | 2,2E-04 |
| GLRB | ENSG00000109738 | 1,75 | 7,4E-04 |
| AASDHPPT | ENSG00000149313 | 1,75 | 2,6E-04 |
| FAM35A | ENSG00000122376 | 1,75 | 4,2E-04 |
| UFM1 | ENSG00000120686 | 1,74 | 3,5E-04 |
| BZW1 | ENSG00000082153 | 1,74 | 3,0E-04 |
| DYNC1I1 | ENSG00000158560 | 1,74 | 1,2E-03 |
| SLC35A5 | ENSG00000138459 | 1,73 | 1,3E-03 |
| HAUS2 | ENSG00000137814 | 1,73 | 4,3E-04 |
| SLC25A24 | ENSG00000085491 | 1,72 | 2,2E-04 |
| COPS4 | ENSG00000138663 | 1,72 | 2,8E-03 |
| UBLCP1 | ENSG00000164332 | 1,72 | 1,0E-03 |
| POMP | ENSG00000132963 | 1,72 | 8,5E-04 |
| C14orf142 | ENSG00000170270 | 1,72 | 7,5E-04 |
| C5orf43 | ENSG00000188725 | 1,71 | 9,5E-04 |
| BAG2 | ENSG00000112208 | 1,71 | 8,4E-04 |
| TWSG1 | ENSG00000128791 | 1,71 | 7,6E-04 |
| C3orf23 | ENSG00000179152 | 1,71 | 4,9E-04 |
| WDR89 | ENSG00000140006 | 1,70 | 3,2E-03 |
| ESCO2 | ENSG00000171320 | 1,70 | 1,2E-03 |
| NSL1 | ENSG00000117697 | 1,70 | 5,2E-04 |
| GLS | ENSG00000115419 | 1,69 | 5,4E-04 |
| KIAA1715 | ENSG00000144320 | 1,69 | 5,3E-04 |
| RAB18 | ENSG00000099246 | 1,69 | 7,2E-04 |
| PJA2 | ENSG00000198961 | 1,68 | 1,2E-03 |
| CGGBP1 | ENSG00000163320 | 1,68 | 2,5E-04 |
| MTFR1 | ENSG00000066855 | 1,68 | 4,5E-04 |
| GTDC1 | ENSG00000121964 | 1,67 | 2,4E-03 |
| LBR | ENSG00000143815 | 1,67 | 1,5E-04 |
| RCN2 | ENSG00000117906 | 1,67 | 3,0E-04 |
| CRYZ | ENSG00000116791 | 1,66 | 1,3E-03 |
| C9orf21 | ENSG00000158122 | 1,66 | 1,1E-03 |
| DCUN1D1 | ENSG00000043093 | 1,66 | 1,7E-03 |
| C4orf46 | ENSG00000205208 | 1,66 | 1,0E-03 |
| PPPDE1 | ENSG00000121644 | 1,65 | 1,1E-03 |
| DENR | ENSG00000139726 | 1,65 | 4,2E-04 |
| ESD | ENSG00000139684 | 1,65 | 9,6E-04 |
| GYG1 | ENSG00000163754 | 1,64 | 7,4E-04 |
| RAD51AP1 | ENSG00000111247 | 1,64 | 1,1E-03 |
| C12orf23 | ENSG00000151135 | 1,63 | 2,2E-04 |
| TMX3 | ENSG00000166479 | 1,63 | 1,0E-03 |
| ATP6V1C1 | ENSG00000155097 | 1,62 | 9,1E-04 |
| NUDT15 | ENSG00000136159 | 1,62 | 7,7E-04 |
| AK3 | ENSG00000147853 | 1,62 | 5,5E-04 |
| RAB14 | ENSG00000119396 | 1,62 | 2,0E-03 |
| PRKRA | ENSG00000180228 | 1,62 | 1,3E-03 |
| LIPA | ENSG00000107798 | 1,62 | 6,7E-05 |
| BROX | ENSG00000162819 | 1,62 | 3,0E-04 |
| FAR1 | ENSG00000197601 | 1,62 | 8,6E-04 |
| EIF1AX | ENSG00000173674 | 1,61 | 1,3E-03 |
| TUSC3 | ENSG00000104723 | 1,61 | 8,0E-04 |
| TMX1 | ENSG00000139921 | 1,61 | 7,3E-04 |
| RABGGTB | ENSG00000137955 | 1,61 | 5,7E-04 |
| FAM91A1 | ENSG00000176853 | 1,61 | 4,6E-04 |
| C11orf82 | ENSG00000165490 | 1,60 | 1,1E-03 |
| DR1 | ENSG00000117505 | 1,60 | 6,3E-05 |
| SUB1 | ENSG00000113387 | 1,60 | 3,2E-03 |
| TMEM19 | ENSG00000139291 | 1,60 | 1,6E-03 |
| EXOC5 | ENSG00000070367 | 1,59 | 6,3E-04 |
| SDCBP | ENSG00000137575 | 1,59 | 1,1E-03 |
| PRKAG2 | ENSG00000106617 | 1,59 | 2,0E-03 |
| GLMN | ENSG00000174842 | 1,59 | 3,0E-03 |
| TBC1D19 | ENSG00000109680 | 1,59 | 1,7E-03 |
| RNF141 | ENSG00000110315 | 1,58 | 1,8E-03 |
| TMEM167B | ENSG00000215717 | 1,58 | 2,4E-04 |
| ZWILCH | ENSG00000174442 | 1,58 | 4,9E-04 |
| ELOVL5 | ENSG00000012660 | 1,58 | 4,8E-04 |
| HIF1A | ENSG00000100644 | 1,58 | 6,4E-04 |
| AC004797.1 | ENSG00000229944 | 1,57 | 1,9E-03 |
| TAF9 | ENSG00000085231 | 1,57 | 2,7E-03 |
| CENPN | ENSG00000166451 | 1,56 | 1,2E-03 |
| CAV1 | ENSG00000105974 | 1,56 | 3,2E-04 |
| CYCS | ENSG00000172115 | 1,56 | 1,8E-03 |
| ECT2 | ENSG00000114346 | 1,56 | 8,2E-04 |
| GPX8 | ENSG00000164294 | 1,55 | 1,0E-03 |
| MRS2 | ENSG00000124532 | 1,55 | 7,9E-04 |
| OXR1 | ENSG00000164830 | 1,55 | 6,3E-04 |
| PLOD2 | ENSG00000152952 | 1,54 | 2,2E-04 |
| TMEM30A | ENSG00000112697 | 1,54 | 2,4E-04 |
| FAM60A | ENSG00000139146 | 1,53 | 4,0E-03 |
| OSTM1 | ENSG00000081087 | 1,53 | 4,3E-04 |
| PM20D2 | ENSG00000146281 | 1,53 | 1,7E-03 |
| G3BP1 | ENSG00000145907 | 1,53 | 1,0E-03 |
| SNX14 | ENSG00000135317 | 1,52 | 2,1E-03 |
| ABCE1 | ENSG00000164163 | 1,52 | 2,2E-03 |
| INSIG2 | ENSG00000125629 | 1,52 | 2,0E-03 |
| EFHA1 | ENSG00000165487 | 1,52 | 3,1E-03 |
| TMED5 | ENSG00000117500 | 1,52 | 1,1E-03 |
| ACADM | ENSG00000117054 | 1,51 | 2,4E-03 |
| SLC38A2 | ENSG00000134294 | 1,51 | 9,9E-04 |
| SPTLC1 | ENSG00000090054 | 1,51 | 9,8E-04 |
| VPS29 | ENSG00000111237 | 1,51 | 3,1E-03 |
| RFC3 | ENSG00000133119 | 1,51 | 1,7E-03 |
| FYTTD1 | ENSG00000122068 | 1,50 | 7,4E-04 |
| SUCLA2 | ENSG00000136143 | 1,50 | 2,3E-03 |
| PGM2 | ENSG00000169299 | 1,50 | 2,8E-04 |
| TIMM17A | ENSG00000134375 | 1,50 | 6,7E-04 |
| ABCD3 | ENSG00000117528 | 1,49 | 1,5E-03 |
| LAMP2 | ENSG00000005893 | 1,49 | 7,7E-04 |
| HSF2 | ENSG00000025156 | 1,48 | 3,9E-03 |
| MELK | ENSG00000165304 | 1,48 | 2,3E-04 |
| PSMC6 | ENSG00000100519 | 1,47 | 2,0E-03 |
| BPNT1 | ENSG00000162813 | 1,47 | 2,7E-03 |
| CRBN | ENSG00000113851 | 1,47 | 3,6E-03 |
| UGCG | ENSG00000148154 | 1,47 | 8,3E-04 |
| CHORDC1 | ENSG00000110172 | 1,46 | 3,4E-03 |
| LMAN1 | ENSG00000074695 | 1,46 | 5,0E-04 |
| FAM49B | ENSG00000153310 | 1,46 | 1,9E-03 |
| GS1-251I9.4 | ENSG00000253738 | 1,46 | 3,4E-03 |
| PLDN | ENSG00000104164 | 1,46 | 2,2E-03 |
| SP3 | ENSG00000172845 | 1,46 | 6,0E-04 |
| SNX6 | ENSG00000129515 | 1,45 | 4,2E-03 |
| MFN1 | ENSG00000171109 | 1,45 | 1,7E-03 |
| TFAM | ENSG00000108064 | 1,45 | 1,9E-03 |
| COPS3 | ENSG00000141030 | 1,45 | 4,1E-03 |
| RAB11A | ENSG00000103769 | 1,45 | 2,5E-03 |
| ACSL3 | ENSG00000123983 | 1,44 | 4,0E-04 |
| EIF3E | ENSG00000104408 | 1,44 | 1,4E-03 |
| SSR3 | ENSG00000114850 | 1,44 | 9,3E-04 |
| TMEM123 | ENSG00000152558 | 1,43 | 1,0E-03 |
| MTMR2 | ENSG00000087053 | 1,42 | 1,4E-03 |
| C16orf87 | ENSG00000155330 | 1,42 | 4,2E-03 |
| PRPSAP2 | ENSG00000141127 | 1,41 | 1,5E-03 |
| TIA1 | ENSG00000116001 | 1,40 | 1,6E-03 |
| CHEK1 | ENSG00000149554 | 1,40 | 3,1E-03 |
| TBC1D15 | ENSG00000121749 | 1,39 | 3,6E-03 |
| FBXL3 | ENSG00000005812 | 1,38 | 2,0E-03 |
| MBNL1 | ENSG00000152601 | 1,37 | 3,2E-03 |
| KBTBD6 | ENSG00000165572 | 1,36 | 2,5E-03 |
| SEH1L | ENSG00000085415 | 1,36 | 3,3E-03 |
| CLIC4 | ENSG00000169504 | 1,36 | 3,8E-04 |
| GALNT1 | ENSG00000141429 | 1,34 | 1,8E-03 |
| FAM3C | ENSG00000196937 | 1,34 | 3,1E-03 |
| SLC39A6 | ENSG00000141424 | 1,33 | 2,6E-03 |
| ANXA7 | ENSG00000138279 | 1,33 | 2,6E-03 |
| CDC7 | ENSG00000097046 | 1,33 | 2,2E-03 |
| SOAT1 | ENSG00000057252 | 1,33 | 2,4E-03 |
| BIRC2 | ENSG00000110330 | 1,32 | 3,8E-03 |
| CTGF | ENSG00000118523 | 1,32 | 1,9E-03 |
| DLD | ENSG00000091140 | 1,32 | 2,6E-03 |
| TSN | ENSG00000211460 | 1,32 | 1,7E-03 |
| BCKDHB | ENSG00000083123 | 1,32 | 2,6E-03 |
| CAND1 | ENSG00000111530 | 1,31 | 1,7E-03 |
| NAA50 | ENSG00000121579 | 1,30 | 5,9E-04 |
| TMPO | ENSG00000120802 | 1,30 | 3,1E-03 |
| UBE2N | ENSG00000177889 | 1,30 | 2,7E-03 |
| MTHFD2 | ENSG00000065911 | 1,29 | 2,0E-03 |
| TAF13 | ENSG00000197780 | 1,29 | 2,8E-03 |
| BNIP3L | ENSG00000104765 | 1,29 | 2,5E-03 |
| SLC30A9 | ENSG00000014824 | 1,27 | 2,1E-03 |
| STT3B | ENSG00000163527 | 1,27 | 1,8E-03 |
| HS2ST1 | ENSG00000153936 | 1,27 | 3,7E-03 |
| API5 | ENSG00000166181 | 1,26 | 2,5E-03 |
| ZFR | ENSG00000056097 | 1,25 | 2,4E-03 |
| RAB23 | ENSG00000112210 | 1,25 | 4,1E-03 |
| UBE2D3 | ENSG00000109332 | 1,24 | 4,3E-03 |
| HSPA14 | ENSG00000187522 | 1,23 | 3,7E-03 |
| UGP2 | ENSG00000169764 | 1,21 | 3,5E-03 |
| PRNP | ENSG00000171867 | 1,21 | 4,2E-03 |
| BTBD1 | ENSG00000064726 | 1,20 | 3,2E-03 |
| GRSF1 | ENSG00000132463 | 1,15 | 2,4E-03 |
