# Supplementary material for: Genome-wide identification of Wig-1 mRNA targets by RIP-Seq analysis
Source: Oncotarget. 2015 Dec 11;7(2):1895–911. doi: 10.18632/oncotarget.6557 (PMC4811505; doi:10.18632/oncotarget.6557)
Supplement: Supplementary file 4 [file oncotarget-07-1895-s004.doc]

| Supplementary Table S4: List of the 286 common Wig-1-bound RNAs in HCT116 and Saos-2 cells.   | **Gene Name** | **Ensembl Gene ID** | **Description** | **Gene Biotype** | | --- | --- | --- | --- | | TSPAN6 | ENSG00000000003 | tetraspanin 6 | protein_coding | | FBXL3 | ENSG00000005812 | F-box and leucine-rich repeat protein 3 | protein_coding | | LAMP2 | ENSG00000005893 | lysosomal-associated membrane protein 2 | protein_coding | | STARD3NL | ENSG00000010270 | STARD3 N-terminal like | protein_coding | | ELOVL5 | ENSG00000012660 | ELOVL fatty acid elongase 5 | protein_coding | | SLC30A9 | ENSG00000014824 | solute carrier family 30 (zinc transporter), member 9 | protein_coding | | YAF2 | ENSG00000015153 | YY1 associated factor 2 | protein_coding | | DEPDC1 | ENSG00000024526 | DEP domain containing 1 | protein_coding | | DCUN1D1 | ENSG00000043093 | DCN1, defective in cullin neddylation 1, domain containing 1 | protein_coding | | SCML1 | ENSG00000047634 | sex comb on midleg-like 1 (Drosophila) | protein_coding | | RRM2B | ENSG00000048392 | ribonucleotide reductase M2 B (TP53 inducible) | protein_coding | | ERCC8 | ENSG00000049167 | excision repair cross-complementing rodent repair deficiency, complementation group 8 | protein_coding | | ZFR | ENSG00000056097 | zinc finger RNA binding protein | protein_coding | | SOAT1 | ENSG00000057252 | sterol O-acyltransferase 1 | protein_coding | | ATG5 | ENSG00000057663 | autophagy related 5 | protein_coding | | SNX24 | ENSG00000064652 | sorting nexin 24 | protein_coding | | BTBD1 | ENSG00000064726 | BTB (POZ) domain containing 1 | protein_coding | | CYB5R4 | ENSG00000065615 | cytochrome b5 reductase 4 | protein_coding | | MTHFD2 | ENSG00000065911 | methylenetetrahydrofolate dehydrogenase (NADP+ dependent) 2 | protein_coding | | MTFR1 | ENSG00000066855 | mitochondrial fission regulator 1 | protein_coding | | EXOC5 | ENSG00000070367 | exocyst complex component 5 | protein_coding | | MGAT4A | ENSG00000071073 | mannosyl (alpha-1,3-)-glycoprotein beta-1,4-N-acetylglucosaminyltransferase, isozyme A | protein_coding | | LMAN1 | ENSG00000074695 | lectin, mannose-binding, 1 | protein_coding | | ACTR6 | ENSG00000075089 | ARP6 actin-related protein 6 homolog (yeast) | protein_coding | | RBM7 | ENSG00000076053 | RNA binding motif protein 7 | protein_coding | | OSTM1 | ENSG00000081087 | osteopetrosis associated transmembrane protein 1 | protein_coding | | STRADB | ENSG00000082146 | STE20-related kinase adaptor beta | protein_coding | | BZW1 | ENSG00000082153 | basic leucine zipper and W2 domains 1 | protein_coding | | ZMPSTE24 | ENSG00000084073 | zinc metallopeptidase STE24 | protein_coding | | MYNN | ENSG00000085274 | myoneurin | protein_coding | | SEH1L | ENSG00000085415 | SEH1-like (S. cerevisiae) | protein_coding | | SLC25A24 | ENSG00000085491 | solute carrier family 25 (mitochondrial carrier; phosphate carrier), member 24 | protein_coding | | ZFAND6 | ENSG00000086666 | zinc finger, AN1-type domain 6 | protein_coding | | MTMR2 | ENSG00000087053 | myotubularin related protein 2 | protein_coding | | SPTLC1 | ENSG00000090054 | serine palmitoyltransferase, long chain base subunit 1 | protein_coding | | DLD | ENSG00000091140 | dihydrolipoamide dehydrogenase | protein_coding | | CMTM6 | ENSG00000091317 | CKLF-like MARVEL transmembrane domain containing 6 | protein_coding | | SEC22C | ENSG00000093183 | SEC22 vesicle trafficking protein homolog C (S. cerevisiae) | protein_coding | | TMEM38B | ENSG00000095209 | transmembrane protein 38B | protein_coding | | CDC7 | ENSG00000097046 | cell division cycle 7 | protein_coding | | RAB18 | ENSG00000099246 | RAB18, member RAS oncogene family | protein_coding | | PSMC6 | ENSG00000100519 | proteasome (prosome, macropain) 26S subunit, ATPase, 6 | protein_coding | | HIF1A | ENSG00000100644 | hypoxia inducible factor 1, alpha subunit (basic helix-loop-helix transcription factor) | protein_coding | | GSKIP | ENSG00000100744 | GSK3B interacting protein | protein_coding | | SEC23A | ENSG00000100934 | Sec23 homolog A (S. cerevisiae) | protein_coding | | IFT52 | ENSG00000101052 | intraflagellar transport 52 homolog (Chlamydomonas) | protein_coding | | MOSPD1 | ENSG00000101928 | motile sperm domain containing 1 | protein_coding | | NDFIP2 | ENSG00000102471 | Nedd4 family interacting protein 2 | protein_coding | | BLOC1S6 | ENSG00000104164 | biogenesis of lysosomal organelles complex-1, subunit 6, pallidin | protein_coding | | RAB2A | ENSG00000104388 | RAB2A, member RAS oncogene family | protein_coding | | ARMC1 | ENSG00000104442 | armadillo repeat containing 1 | protein_coding | | BNIP3L | ENSG00000104765 | BCL2/adenovirus E1B 19kDa interacting protein 3-like | protein_coding | | CAV2 | ENSG00000105971 | caveolin 2 | protein_coding | | CAV1 | ENSG00000105974 | caveolin 1, caveolae protein, 22kDa | protein_coding | | PRKAG2 | ENSG00000106617 | protein kinase, AMP-activated, gamma 2 non-catalytic subunit | protein_coding | | PLGRKT | ENSG00000107020 | plasminogen receptor, C-terminal lysine transmembrane protein | protein_coding | | LIPA | ENSG00000107798 | lipase A, lysosomal acid, cholesterol esterase | protein_coding | | TUBD1 | ENSG00000108423 | tubulin, delta 1 | protein_coding | | MMD | ENSG00000108960 | monocyte to macrophage differentiation-associated | protein_coding | | UBE2D3 | ENSG00000109332 | ubiquitin-conjugating enzyme E2D 3 | protein_coding | | TBC1D19 | ENSG00000109680 | TBC1 domain family, member 19 | protein_coding | | CHORDC1 | ENSG00000110172 | cysteine and histidine-rich domain (CHORD) containing 1 | protein_coding | | RNF141 | ENSG00000110315 | ring finger protein 141 | protein_coding | | BIRC2 | ENSG00000110330 | baculoviral IAP repeat containing 2 | protein_coding | | CAND1 | ENSG00000111530 | cullin-associated and neddylation-dissociated 1 | protein_coding | | GOLT1B | ENSG00000111711 | golgi transport 1B | protein_coding | | ASF1A | ENSG00000111875 | anti-silencing function 1A histone chaperone | protein_coding | | BAG2 | ENSG00000112208 | BCL2-associated athanogene 2 | protein_coding | | RAB23 | ENSG00000112210 | RAB23, member RAS oncogene family | protein_coding | | TMEM30A | ENSG00000112697 | transmembrane protein 30A | protein_coding | | CCNG1 | ENSG00000113328 | cyclin G1 | protein_coding | | ARRDC3 | ENSG00000113369 | arrestin domain containing 3 | protein_coding | | SUB1 | ENSG00000113387 | SUB1 homolog (S. cerevisiae) | protein_coding | | TRAPPC13 | ENSG00000113597 | trafficking protein particle complex 13 | protein_coding | | TTC33 | ENSG00000113638 | tetratricopeptide repeat domain 33 | protein_coding | | ECT2 | ENSG00000114346 | epithelial cell transforming sequence 2 oncogene | protein_coding | | SSR3 | ENSG00000114850 | signal sequence receptor, gamma (translocon-associated protein gamma) | protein_coding | | GLS | ENSG00000115419 | glutaminase | protein_coding | | TXNDC9 | ENSG00000115514 | thioredoxin domain containing 9 | protein_coding | | MOB4 | ENSG00000115540 | MOB family member 4, phocein | protein_coding | | ORC4 | ENSG00000115947 | origin recognition complex, subunit 4 | protein_coding | | TIA1 | ENSG00000116001 | TIA1 cytotoxic granule-associated RNA binding protein | protein_coding | | SCP2 | ENSG00000116171 | sterol carrier protein 2 | protein_coding | | TMEM59 | ENSG00000116209 | transmembrane protein 59 | protein_coding | | TSNAX | ENSG00000116918 | translin-associated factor X | protein_coding | | ACADM | ENSG00000117054 | acyl-CoA dehydrogenase, C-4 to C-12 straight chain | protein_coding | | TMED5 | ENSG00000117500 | transmembrane emp24 protein transport domain containing 5 | protein_coding | | DR1 | ENSG00000117505 | down-regulator of transcription 1, TBP-binding (negative cofactor 2) | protein_coding | | ABCD3 | ENSG00000117528 | ATP-binding cassette, sub-family D (ALD), member 3 | protein_coding | | SLC35A3 | ENSG00000117620 | solute carrier family 35 (UDP-N-acetylglucosamine (UDP-GlcNAc) transporter), member A3 | protein_coding | | NSL1 | ENSG00000117697 | NSL1, MIS12 kinetochore complex component | protein_coding | | RCN2 | ENSG00000117906 | reticulocalbin 2, EF-hand calcium binding domain | protein_coding | | CTGF | ENSG00000118523 | connective tissue growth factor | protein_coding | | MFSD1 | ENSG00000118855 | major facilitator superfamily domain containing 1 | protein_coding | | UBE2B | ENSG00000119048 | ubiquitin-conjugating enzyme E2B | protein_coding | | RAB14 | ENSG00000119396 | RAB14, member RAS oncogene family | protein_coding | | YIPF4 | ENSG00000119820 | Yip1 domain family, member 4 | protein_coding | | UFM1 | ENSG00000120686 | ubiquitin-fold modifier 1 | protein_coding | | TMPO | ENSG00000120802 | thymopoietin | protein_coding | | LYPLA1 | ENSG00000120992 | lysophospholipase I | protein_coding | | NAA50 | ENSG00000121579 | N(alpha)-acetyltransferase 50, NatE catalytic subunit | protein_coding | | DESI2 | ENSG00000121644 | desumoylating isopeptidase 2 | protein_coding | | TBC1D15 | ENSG00000121749 | TBC1 domain family, member 15 | protein_coding | | GTDC1 | ENSG00000121964 | glycosyltransferase-like domain containing 1 | protein_coding | | FYTTD1 | ENSG00000122068 | forty-two-three domain containing 1 | protein_coding | | FAM35A | ENSG00000122376 | family with sequence similarity 35, member A | protein_coding | | SLC25A16 | ENSG00000122912 | solute carrier family 25 (mitochondrial carrier; Graves disease autoantigen), member 16 | protein_coding | | RNF11 | ENSG00000123091 | ring finger protein 11 | protein_coding | | ATF1 | ENSG00000123268 | activating transcription factor 1 | protein_coding | | AMD1 | ENSG00000123505 | adenosylmethionine decarboxylase 1 | protein_coding | | ACSL3 | ENSG00000123983 | acyl-CoA synthetase long-chain family member 3 | protein_coding | | VAMP7 | ENSG00000124333 | vesicle-associated membrane protein 7 | protein_coding | | MRS2 | ENSG00000124532 | MRS2 magnesium transporter | protein_coding | | EEF1E1 | ENSG00000124802 | eukaryotic translation elongation factor 1 epsilon 1 | protein_coding | | INSIG2 | ENSG00000125629 | insulin induced gene 2 | protein_coding | | SGPP1 | ENSG00000126821 | sphingosine-1-phosphate phosphatase 1 | protein_coding | | DNAJB9 | ENSG00000128590 | DnaJ (Hsp40) homolog, subfamily B, member 9 | protein_coding | | NDUFA5 | ENSG00000128609 | NADH dehydrogenase (ubiquinone) 1 alpha subcomplex, 5 | protein_coding | | OSGEPL1 | ENSG00000128694 | O-sialoglycoprotein endopeptidase-like 1 | protein_coding | | TWSG1 | ENSG00000128791 | twisted gastrulation BMP signaling modulator 1 | protein_coding | | ANAPC13 | ENSG00000129055 | anaphase promoting complex subunit 13 | protein_coding | | DTD2 | ENSG00000129480 | D-tyrosyl-tRNA deacylase 2 (putative) | protein_coding | | GRSF1 | ENSG00000132463 | G-rich RNA sequence binding factor 1 | protein_coding | | RFC3 | ENSG00000133119 | replication factor C (activator 1) 3, 38kDa | protein_coding | | FOPNL | ENSG00000133393 | FGFR1OP N-terminal like | protein_coding | | C12orf29 | ENSG00000133641 | chromosome 12 open reading frame 29 | protein_coding | | ARL8B | ENSG00000134108 | ADP-ribosylation factor-like 8B | protein_coding | | SLC38A2 | ENSG00000134294 | solute carrier family 38, member 2 | protein_coding | | TIMM17A | ENSG00000134375 | translocase of inner mitochondrial membrane 17 homolog A (yeast) | protein_coding | | RNF138 | ENSG00000134758 | ring finger protein 138, E3 ubiquitin protein ligase | protein_coding | | RFK | ENSG00000135002 | riboflavin kinase | protein_coding | | SNX14 | ENSG00000135317 | sorting nexin 14 | protein_coding | | SUCLA2 | ENSG00000136143 | succinate-CoA ligase, ADP-forming, beta subunit | protein_coding | | NDUFB5 | ENSG00000136521 | NADH dehydrogenase (ubiquinone) 1 beta subcomplex, 5, 16kDa | protein_coding | | NIPSNAP3A | ENSG00000136783 | nipsnap homolog 3A (C. elegans) | protein_coding | | SDCBP | ENSG00000137575 | syndecan binding protein (syntenin) | protein_coding | | FDX1 | ENSG00000137714 | ferredoxin 1 | protein_coding | | HAUS2 | ENSG00000137814 | HAUS augmin-like complex, subunit 2 | protein_coding | | RSL24D1 | ENSG00000137876 | ribosomal L24 domain containing 1 | protein_coding | | GTF2B | ENSG00000137947 | general transcription factor IIB | protein_coding | | RABGGTB | ENSG00000137955 | Rab geranylgeranyltransferase, beta subunit | protein_coding | | ASNSD1 | ENSG00000138381 | asparagine synthetase domain containing 1 | protein_coding | | SLC35A5 | ENSG00000138459 | solute carrier family 35, member A5 | protein_coding | | COPS4 | ENSG00000138663 | COP9 signalosome subunit 4 | protein_coding | | NUP54 | ENSG00000138750 | nucleoporin 54kDa | protein_coding | | PPA2 | ENSG00000138777 | pyrophosphatase (inorganic) 2 | protein_coding | | SLC39A8 | ENSG00000138821 | solute carrier family 39 (zinc transporter), member 8 | protein_coding | | CPNE8 | ENSG00000139117 | copine VIII | protein_coding | | FAM60A | ENSG00000139146 | family with sequence similarity 60, member A | protein_coding | | TMEM117 | ENSG00000139173 | transmembrane protein 117 | protein_coding | | TMEM19 | ENSG00000139291 | transmembrane protein 19 | protein_coding | | MTMR6 | ENSG00000139505 | myotubularin related protein 6 | protein_coding | | DENR | ENSG00000139726 | density-regulated protein | protein_coding | | ABHD13 | ENSG00000139826 | abhydrolase domain containing 13 | protein_coding | | TMX1 | ENSG00000139921 | thioredoxin-related transmembrane protein 1 | protein_coding | | COPS3 | ENSG00000141030 | COP9 signalosome subunit 3 | protein_coding | | PRPSAP2 | ENSG00000141127 | phosphoribosyl pyrophosphate synthetase-associated protein 2 | protein_coding | | SLC39A6 | ENSG00000141424 | solute carrier family 39 (zinc transporter), member 6 | protein_coding | | GALNT1 | ENSG00000141429 | UDP-N-acetyl-alpha-D-galactosamine:polypeptide N-acetylgalactosaminyltransferase 1 (GalNAc-T1) | protein_coding | | LBR | ENSG00000143815 | lamin B receptor | protein_coding | | CHAC2 | ENSG00000143942 | ChaC, cation transport regulator homolog 2 (E. coli) | protein_coding | | KIAA1715 | ENSG00000144320 | KIAA1715 | protein_coding | | ABHD10 | ENSG00000144827 | abhydrolase domain containing 10 | protein_coding | | CISD2 | ENSG00000145354 | CDGSH iron sulfur domain 2 | protein_coding | | ATG12 | ENSG00000145782 | autophagy related 12 | protein_coding | | G3BP1 | ENSG00000145907 | GTPase activating protein (SH3 domain) binding protein 1 | protein_coding | | PM20D2 | ENSG00000146281 | peptidase M20 domain containing 2 | protein_coding | | ABRACL | ENSG00000146386 | ABRA C-terminal like | protein_coding | | C6orf211 | ENSG00000146476 | chromosome 6 open reading frame 211 | protein_coding | | AK3 | ENSG00000147853 | adenylate kinase 3 | protein_coding | | UGCG | ENSG00000148154 | UDP-glucose ceramide glucosyltransferase | protein_coding | | FAM188A | ENSG00000148481 | family with sequence similarity 188, member A | protein_coding | | AASDHPPT | ENSG00000149313 | aminoadipate-semialdehyde dehydrogenase-phosphopantetheinyl transferase | protein_coding | | CHEK1 | ENSG00000149554 | checkpoint kinase 1 | protein_coding | | C12orf23 | ENSG00000151135 | chromosome 12 open reading frame 23 | protein_coding | | TWF1 | ENSG00000151239 | twinfilin actin-binding protein 1 | protein_coding | | EIF4E | ENSG00000151247 | eukaryotic translation initiation factor 4E | protein_coding | | NEK7 | ENSG00000151414 | NIMA-related kinase 7 | protein_coding | | AP1S3 | ENSG00000152056 | adaptor-related protein complex 1, sigma 3 subunit | protein_coding | | SUV39H2 | ENSG00000152455 | suppressor of variegation 3-9 homolog 2 (Drosophila) | protein_coding | | TMEM123 | ENSG00000152558 | transmembrane protein 123 | protein_coding | | MBNL1 | ENSG00000152601 | muscleblind-like splicing regulator 1 | protein_coding | | GPR180 | ENSG00000152749 | G protein-coupled receptor 180 | protein_coding | | PLOD2 | ENSG00000152952 | procollagen-lysine, 2-oxoglutarate 5-dioxygenase 2 | protein_coding | | SCOC | ENSG00000153130 | short coiled-coil protein | protein_coding | | FAM49B | ENSG00000153310 | family with sequence similarity 49, member B | protein_coding | | HS2ST1 | ENSG00000153936 | heparan sulfate 2-O-sulfotransferase 1 | protein_coding | | DPH3 | ENSG00000154813 | diphthamide biosynthesis 3 | protein_coding | | ATP6V1C1 | ENSG00000155097 | ATPase, H+ transporting, lysosomal 42kDa, V1 subunit C1 | protein_coding | | HSPA13 | ENSG00000155304 | heat shock protein 70kDa family, member 13 | protein_coding | | CNOT8 | ENSG00000155508 | CCR4-NOT transcription complex, subunit 8 | protein_coding | | DCK | ENSG00000156136 | deoxycytidine kinase | protein_coding | | DRAM2 | ENSG00000156171 | DNA-damage regulated autophagy modulator 2 | protein_coding | | HIAT1 | ENSG00000156875 | hippocampus abundant transcript 1 | protein_coding | | AAED1 | ENSG00000158122 | AhpC/TSA antioxidant enzyme domain containing 1 | protein_coding | | CMPK1 | ENSG00000162368 | cytidine monophosphate (UMP-CMP) kinase 1, cytosolic | protein_coding | | SNX7 | ENSG00000162627 | sorting nexin 7 | protein_coding | | BPNT1 | ENSG00000162813 | 3'(2'), 5'-bisphosphate nucleotidase 1 | protein_coding | | BROX | ENSG00000162819 | BRO1 domain and CAAX motif containing | protein_coding | | ARL5A | ENSG00000162980 | ADP-ribosylation factor-like 5A | protein_coding | | NUP35 | ENSG00000163002 | nucleoporin 35kDa | protein_coding | | CGGBP1 | ENSG00000163320 | CGG triplet repeat binding protein 1 | protein_coding | | STT3B | ENSG00000163527 | STT3B, subunit of the oligosaccharyltransferase complex (catalytic) | protein_coding | | EIF5A2 | ENSG00000163577 | eukaryotic translation initiation factor 5A2 | protein_coding | | MTHFD2L | ENSG00000163738 | methylenetetrahydrofolate dehydrogenase (NADP+ dependent) 2-like | protein_coding | | GYG1 | ENSG00000163754 | glycogenin 1 | protein_coding | | MAD2L1 | ENSG00000164109 | MAD2 mitotic arrest deficient-like 1 (yeast) | protein_coding | | ABCE1 | ENSG00000164163 | ATP-binding cassette, sub-family E (OABP), member 1 | protein_coding | | STARD4 | ENSG00000164211 | StAR-related lipid transfer (START) domain containing 4 | protein_coding | | OXR1 | ENSG00000164830 | oxidation resistance 1 | protein_coding | | SLC25A32 | ENSG00000164933 | solute carrier family 25 (mitochondrial folate carrier), member 32 | protein_coding | | MELK | ENSG00000165304 | maternal embryonic leucine zipper kinase | protein_coding | | CFL2 | ENSG00000165410 | cofilin 2 (muscle) | protein_coding | | MICU2 | ENSG00000165487 | mitochondrial calcium uptake 2 | protein_coding | | C11orf82 | ENSG00000165490 | chromosome 11 open reading frame 82 | protein_coding | | KBTBD6 | ENSG00000165572 | kelch repeat and BTB (POZ) domain containing 6 | protein_coding | | TRUB1 | ENSG00000165832 | TruB pseudouridine (psi) synthase family member 1 | protein_coding | | API5 | ENSG00000166181 | apoptosis inhibitor 5 | protein_coding | | C10orf32 | ENSG00000166275 | chromosome 10 open reading frame 32 | protein_coding | | TMX3 | ENSG00000166479 | thioredoxin-related transmembrane protein 3 | protein_coding | | TMEM135 | ENSG00000166575 | transmembrane protein 135 | protein_coding | | MIS12 | ENSG00000167842 | MIS12 kinetochore complex component | protein_coding | | TMEM68 | ENSG00000167904 | transmembrane protein 68 | protein_coding | | PBK | ENSG00000168078 | PDZ binding kinase | protein_coding | | BMI1 | ENSG00000168283 | BMI1 polycomb ring finger oncogene | protein_coding | | COMMD8 | ENSG00000169019 | COMM domain containing 8 | protein_coding | | CCDC126 | ENSG00000169193 | coiled-coil domain containing 126 | protein_coding | | NMD3 | ENSG00000169251 | NMD3 ribosome export adaptor | protein_coding | | PGM2 | ENSG00000169299 | phosphoglucomutase 2 | protein_coding | | CRADD | ENSG00000169372 | CASP2 and RIPK1 domain containing adaptor with death domain | protein_coding | | CLIC4 | ENSG00000169504 | chloride intracellular channel 4 | protein_coding | | UGP2 | ENSG00000169764 | UDP-glucose pyrophosphorylase 2 | protein_coding | | C14orf142 | ENSG00000170270 | chromosome 14 open reading frame 142 | protein_coding | | MFN1 | ENSG00000171109 | mitofusin 1 | protein_coding | | ESCO2 | ENSG00000171320 | establishment of sister chromatid cohesion N-acetyltransferase 2 | protein_coding | | SP3 | ENSG00000172845 | Sp3 transcription factor | protein_coding | | EIF1AX | ENSG00000173674 | eukaryotic translation initiation factor 1A, X-linked | protein_coding | | ZWILCH | ENSG00000174442 | zwilch kinetochore protein | protein_coding | | GLMN | ENSG00000174842 | glomulin, FKBP associated protein | protein_coding | | PLEKHF2 | ENSG00000175895 | pleckstrin homology domain containing, family F (with FYVE domain) member 2 | protein_coding | | FAM91A1 | ENSG00000176853 | family with sequence similarity 91, member A1 | protein_coding | | ZDHHC13 | ENSG00000177054 | zinc finger, DHHC-type containing 13 | protein_coding | | UBE2N | ENSG00000177889 | ubiquitin-conjugating enzyme E2N | protein_coding | | RMI1 | ENSG00000178966 | RecQ mediated genome instability 1 | protein_coding | | TCAIM | ENSG00000179152 | T cell activation inhibitor, mitochondrial | protein_coding | | ELMOD2 | ENSG00000179387 | ELMO/CED-12 domain containing 2 | protein_coding | | C14orf28 | ENSG00000179476 | chromosome 14 open reading frame 28 | protein_coding | | C14orf119 | ENSG00000179933 | chromosome 14 open reading frame 119 | protein_coding | | PRKRA | ENSG00000180228 | protein kinase, interferon-inducible double stranded RNA dependent activator | protein_coding | | C5orf30 | ENSG00000181751 | chromosome 5 open reading frame 30 | protein_coding | | SNRPE | ENSG00000182004 | small nuclear ribonucleoprotein polypeptide E | protein_coding | | C11orf54 | ENSG00000182919 | chromosome 11 open reading frame 54 | protein_coding | | HMGN4 | ENSG00000182952 | high mobility group nucleosomal binding domain 4 | protein_coding | | SEP15 | ENSG00000183291 | Homo sapiens 15 kDa selenoprotein (SEP15), transcript variant 2, mRNA. | protein_coding | | LIN9 | ENSG00000183814 | lin-9 homolog (C. elegans) | protein_coding | | ANKRD46 | ENSG00000186106 | ankyrin repeat domain 46 | protein_coding | | SMIM15 | ENSG00000188725 | small integral membrane protein 15 | protein_coding | | RNFT1 | ENSG00000189050 | ring finger protein, transmembrane 1 | protein_coding | | FAM3C | ENSG00000196937 | family with sequence similarity 3, member C | protein_coding | | METTL9 | ENSG00000197006 | methyltransferase like 9 | protein_coding | | GMFB | ENSG00000197045 | glia maturation factor, beta | protein_coding | | FAR1 | ENSG00000197601 | fatty acyl CoA reductase 1 | protein_coding | | TAF13 | ENSG00000197780 | TAF13 RNA polymerase II, TATA box binding protein (TBP)-associated factor, 18kDa | protein_coding | | MRPL42 | ENSG00000198015 | mitochondrial ribosomal protein L42 | protein_coding | | HSD17B11 | ENSG00000198189 | hydroxysteroid (17-beta) dehydrogenase 11 | protein_coding | | BZW1P2 | ENSG00000198406 | basic leucine zipper and W2 domains 1 pseudogene 2 | pseudogene | | COPS8 | ENSG00000198612 | COP9 signalosome subunit 8 | protein_coding | | CNOT7 | ENSG00000198791 | CCR4-NOT transcription complex, subunit 7 | protein_coding | | ZNF277 | ENSG00000198839 | zinc finger protein 277 | protein_coding | | SELT | ENSG00000198843 | Selenoprotein T | protein_coding | | CAPZA2 | ENSG00000198898 | capping protein (actin filament) muscle Z-line, alpha 2 | protein_coding | | PJA2 | ENSG00000198961 | praja ring finger 2, E3 ubiquitin protein ligase | protein_coding | | METTL10 | ENSG00000203791 | methyltransferase like 10 | protein_coding | | MZT1 | ENSG00000204899 | mitotic spindle organizing protein 1 | protein_coding | | C4orf46 | ENSG00000205208 | chromosome 4 open reading frame 46 | protein_coding | | CNEP1R1 | ENSG00000205423 | CTD nuclear envelope phosphatase 1 regulatory subunit 1 | protein_coding | | PPP1CB | ENSG00000213639 | protein phosphatase 1, catalytic subunit, beta isozyme | protein_coding | | TMEM167B | ENSG00000215717 | transmembrane protein 167B | protein_coding | | RP11-421L21.3 | ENSG00000233184 |  | antisense | | SNRPEP4 | ENSG00000233270 | small nuclear ribonucleoprotein polypeptide E pseudogene 4 | pseudogene | | RP11-307L3.2 | ENSG00000233846 |  | pseudogene | | GS1-251I9.4 | ENSG00000253738 |  | antisense | | FPGT | ENSG00000254685 | fucose-1-phosphate guanylyltransferase | protein_coding | | RP1-145M24.1 | ENSG00000254708 |  | pseudogene | | RP11-166D19.1 | ENSG00000255248 |  | sense_overlapping | |  |
| --- | --- | --- | --- | --- | --- | --- | --- | --- | --- | --- | --- | --- | --- | --- | --- | --- | --- | --- | --- | --- | --- | --- | --- | --- | --- | --- | --- | --- | --- | --- | --- | --- | --- | --- | --- | --- | --- | --- | --- | --- | --- | --- | --- | --- | --- | --- | --- | --- | --- | --- | --- | --- | --- | --- | --- | --- | --- | --- | --- | --- | --- | --- | --- | --- | --- | --- | --- | --- | --- | --- | --- | --- | --- | --- | --- | --- | --- | --- | --- | --- | --- | --- | --- | --- | --- | --- | --- | --- | --- | --- | --- | --- | --- | --- | --- | --- | --- | --- | --- | --- | --- | --- | --- | --- | --- | --- | --- | --- | --- | --- | --- | --- | --- | --- | --- | --- | --- | --- | --- | --- | --- | --- | --- | --- | --- | --- | --- | --- | --- | --- | --- | --- | --- | --- | --- | --- | --- | --- | --- | --- | --- | --- | --- | --- | --- | --- | --- | --- | --- | --- | --- | --- | --- | --- | --- | --- | --- | --- | --- | --- | --- | --- | --- | --- | --- | --- | --- | --- | --- | --- | --- | --- | --- | --- | --- | --- | --- | --- | --- | --- | --- | --- | --- | --- | --- | --- | --- | --- | --- | --- | --- | --- | --- | --- | --- | --- | --- | --- | --- | --- | --- | --- | --- | --- | --- | --- | --- | --- | --- | --- | --- | --- | --- | --- | --- | --- | --- | --- | --- | --- | --- | --- | --- | --- | --- | --- | --- | --- | --- | --- | --- | --- | --- | --- | --- | --- | --- | --- | --- | --- | --- | --- | --- | --- | --- | --- | --- | --- | --- | --- | --- | --- | --- | --- | --- | --- | --- | --- | --- | --- | --- | --- | --- | --- | --- | --- | --- | --- | --- | --- | --- | --- | --- | --- | --- | --- | --- | --- | --- | --- | --- | --- | --- | --- | --- | --- | --- | --- | --- | --- | --- | --- | --- | --- | --- | --- | --- | --- | --- | --- | --- | --- | --- | --- | --- | --- | --- | --- | --- | --- | --- | --- | --- | --- | --- | --- | --- | --- | --- | --- | --- | --- | --- | --- | --- | --- | --- | --- | --- | --- | --- | --- | --- | --- | --- | --- | --- | --- | --- | --- | --- | --- | --- | --- | --- | --- | --- | --- | --- | --- | --- | --- | --- | --- | --- | --- | --- | --- | --- | --- | --- | --- | --- | --- | --- | --- | --- | --- | --- | --- | --- | --- | --- | --- | --- | --- | --- | --- | --- | --- | --- | --- | --- | --- | --- | --- | --- | --- | --- | --- | --- | --- | --- | --- | --- | --- | --- | --- | --- | --- | --- | --- | --- | --- | --- | --- | --- | --- | --- | --- | --- | --- | --- | --- | --- | --- | --- | --- | --- | --- | --- | --- | --- | --- | --- | --- | --- | --- | --- | --- | --- | --- | --- | --- | --- | --- | --- | --- | --- | --- | --- | --- | --- | --- | --- | --- | --- | --- | --- | --- | --- | --- | --- | --- | --- | --- | --- | --- | --- | --- | --- | --- | --- | --- | --- | --- | --- | --- | --- | --- | --- | --- | --- | --- | --- | --- | --- | --- | --- | --- | --- | --- | --- | --- | --- | --- | --- | --- | --- | --- | --- | --- | --- | --- | --- | --- | --- | --- | --- | --- | --- | --- | --- | --- | --- | --- | --- | --- | --- | --- | --- | --- | --- | --- | --- | --- | --- | --- | --- | --- | --- | --- | --- | --- | --- | --- | --- | --- | --- | --- | --- | --- | --- | --- | --- | --- | --- | --- | --- | --- | --- | --- | --- | --- | --- | --- | --- | --- | --- | --- | --- | --- | --- | --- | --- | --- | --- | --- | --- | --- | --- | --- | --- | --- | --- | --- | --- | --- | --- | --- | --- | --- | --- | --- | --- | --- | --- | --- | --- | --- | --- | --- | --- | --- | --- | --- | --- | --- | --- | --- | --- | --- | --- | --- | --- | --- | --- | --- | --- | --- | --- | --- | --- | --- | --- | --- | --- | --- | --- | --- | --- | --- | --- | --- | --- | --- | --- | --- | --- | --- | --- | --- | --- | --- | --- | --- | --- | --- | --- | --- | --- | --- | --- | --- | --- | --- | --- | --- | --- | --- | --- | --- | --- | --- | --- | --- | --- | --- | --- | --- | --- | --- | --- | --- | --- | --- | --- | --- | --- | --- | --- | --- | --- | --- | --- | --- | --- | --- | --- | --- | --- | --- | --- | --- | --- | --- | --- | --- | --- | --- | --- | --- | --- | --- | --- | --- | --- | --- | --- | --- | --- | --- | --- | --- | --- | --- | --- | --- | --- | --- | --- | --- | --- | --- | --- | --- | --- | --- | --- | --- | --- | --- | --- | --- | --- | --- | --- | --- | --- | --- | --- | --- | --- | --- | --- | --- | --- | --- | --- | --- | --- | --- | --- | --- | --- | --- | --- | --- | --- | --- | --- | --- | --- | --- | --- | --- | --- | --- | --- | --- | --- | --- | --- | --- | --- | --- | --- | --- | --- | --- | --- | --- | --- | --- | --- | --- | --- | --- | --- | --- | --- | --- | --- | --- | --- | --- | --- | --- | --- | --- | --- | --- | --- | --- | --- | --- | --- | --- | --- | --- | --- | --- | --- | --- | --- | --- | --- | --- | --- | --- | --- | --- | --- | --- | --- | --- | --- | --- | --- | --- | --- | --- | --- | --- | --- | --- | --- | --- | --- | --- | --- | --- | --- | --- | --- | --- | --- | --- | --- | --- | --- | --- | --- | --- | --- | --- | --- | --- | --- | --- | --- | --- | --- | --- | --- | --- | --- | --- | --- | --- | --- | --- | --- | --- | --- | --- | --- | --- | --- | --- | --- | --- | --- | --- | --- | --- | --- | --- | --- | --- | --- | --- | --- | --- | --- | --- | --- | --- | --- | --- | --- | --- | --- | --- | --- | --- | --- | --- | --- | --- | --- | --- | --- | --- | --- | --- | --- | --- | --- | --- | --- | --- | --- | --- | --- | --- | --- | --- | --- | --- | --- | --- | --- | --- | --- | --- | --- | --- | --- | --- | --- | --- | --- | --- | --- | --- | --- | --- | --- | --- | --- | --- | --- | --- | --- | --- | --- | --- | --- | --- | --- | --- | --- | --- | --- | --- | --- | --- | --- | --- | --- | --- | --- | --- | --- | --- | --- | --- | --- | --- | --- | --- | --- | --- | --- | --- | --- | --- | --- | --- | --- | --- | --- | --- | --- | --- | --- | --- | --- | --- | --- | --- | --- | --- | --- | --- | --- | --- | --- | --- | --- | --- | --- | --- | --- | --- | --- | --- | --- | --- | --- | --- | --- | --- | --- | --- | --- | --- | --- | --- | --- | --- | --- | --- | --- | --- | --- | --- | --- | --- | --- | --- | --- | --- | --- | --- | --- | --- | --- | --- | --- | --- | --- | --- | --- | --- | --- | --- | --- | --- | --- | --- | --- | --- | --- | --- | --- | --- | --- | --- | --- | --- | --- | --- | --- | --- | --- | --- | --- | --- | --- | --- | --- | --- | --- | --- | --- | --- | --- | --- | --- | --- | --- | --- | --- | --- | --- | --- | --- | --- | --- | --- | --- | --- | --- | --- | --- | --- | --- | --- | --- | --- | --- | --- | --- | --- | --- | --- | --- | --- | --- | --- | --- | --- | --- | --- | --- | --- | --- | --- | --- | --- | --- | --- | --- | --- | --- | --- | --- | --- | --- | --- | --- | --- | --- | --- | --- | --- | --- | --- | --- | --- | --- | --- | --- | --- | --- | --- | --- | --- | --- | --- | --- | --- | --- |

| RP11-307P22.1 | ENSG00000258445 |  | pseudogene |
| --- | --- | --- | --- |
