# Supplementary material for: Genome-wide identification of Wig-1 mRNA targets by RIP-Seq analysis
Source: Oncotarget. 2015 Dec 11;7(2):1895–911. doi: 10.18632/oncotarget.6557 (PMC4811505; doi:10.18632/oncotarget.6557)
Supplement: Supplementary file 5 [file oncotarget-07-1895-s005.doc]

| Supplementary Table S5: List of transcripts that are bound by Wig-1 and whose gene expression levels are affected by Wig-1 silencing in HCT116 cells (GEO database GSE43046), at least 4-fold difference (log2(siRNA Wig-1/siRNA control)) in at least two out of the three replicates. | | | | |
| --- | --- | --- | --- | --- |
| Gene Symbol | Name | log2 (siW1/siC)  array1 | log2 (siW1/siC)  array2 | log2 (siW1/siC)  array3 |
| ADH5 | Alcohol dehydrogenase 5 (class III), chi polypeptide | -2.84 | -3.26 |  |
| ANLN | Anillin, actin binding protein | -1.84 | -2.22 | -3.34 |
| ACTR2 | ARP2 actin-related protein 2 homolog (yeast) | -2.27 | -4.67 | -2.21 |
| BZW1 | Basic leucine zipper and W2 domains 1 | -2.06 | -3.13 | -2.63 |
| BZW1 | Basic leucine zipper and W2 domains 1 | -2.25 | -2.20 | -2.90 |
| BNIP2 | BCL2/adenovirus E1B 19kDa interacting protein 2 | -3.25 |  | -3.56 |
| CAPZA1 | Capping protein (actin filament) muscle Z-line, alpha 1 | -10.44 | -3.48 | -3.07 |
| CKS2 | CDC28 protein kinase regulatory subunit 2 | -3.30 | -2.23 |  |
| CSE1L | CSE1 chromosome segregation 1-like (yeast) | -1.71 | -2.75 | -2.73 |
| CSE1L | CSE1 chromosome segregation 1-like (yeast) | -2.04 | -1.14 | -2.82 |
| CSE1L | CSE1 chromosome segregation 1-like (yeast) |  | 3.15 | 4.34 |
| DEK | DEK oncogene | -1.37 | -2.28 | -3.16 |
| EIF4B | eukaryotic translation initiation factor 4B | -2.17 | -2.69 | -2.32 |
| GPNMB | Glycoprotein (transmembrane) nmb | -4.51 | -2.25 |  |
| ITGB1 | integrin, beta 1 (fibronectin receptor, beta polypeptide, antigen CD29 includes MDF2, MSK12) | -2.30 | -2.11 |  |
| MTHFD2 | Methylenetetrahydrofolate dehydrogenase (NADP+ dependent) 2, methenyltetrahydrofolate cyclohydrolase | -4.42 | -2.90 |  |
| OSTC | Oligosaccharyltransferase complex subunit | -1.39 | -2.34 | -4.20 |
| PPT1 | Palmitoyl-protein thioesterase 1 | -1.13 | -2.95 | -3.31 |
| PSIP1 | PC4 and SFRS1 interacting protein 1 | -4.40 | -1.25 | -2.27 |
| PFN2 | profilin 2 | -2.37 | -4.08 |  |
| PPP2CB | Protein phosphatase 2 (formerly 2A), catalytic subunit, beta isoform | -1.87 | -2.65 | -2.35 |
| RAB10 | RAB10, member RAS oncogene family | -3.48 | -2.09 |  |
| RRM1 | Ribonucleotide reductase M1 | -4.61 | -2.48 | -4.03 |
| TCEA1 | Transcription elongation factor A (SII), 1 | -4.26 | -4.25 |  |
| TRAM1 | Translocation associated membrane protein 1 | -2.32 | -1.89 | -4.45 |
| TRAM1 | Translocation associated membrane protein 1 | -3.30 | -0.46 | -3.46 |
| TMEM66 | Transmembrane protein 66 | -1.71 | -2.24 | -3.01 |
| UBQLN1 | Ubiquilin 1 | -3.28 | -2.62 | -5.34 |
| UGP2 | UDP-glucose pyrophosphorylase 2 | -3.03 | -1.28 | -2.32 |
| ABHD10 | abhydrolase domain containing 10 |  | 3.11 | 2.74 |
| AKAP11 | A kinase (PRKA) anchor protein 11 | 5.88 |  | 3.77 |
| AKT3 | V-akt murine thymoma viral oncogene homolog 3 (protein kinase B, gamma) | 2.12 | 3.24 |  |
| ALG10 | Asparagine-linked glycosylation 10 homolog (yeast, alpha-1,2-glucosyltransferase) | 1.10 | 3.08 | 4.16 |
| AP3B1 | Adaptor-related protein complex 3, beta 1 subunit |  | 3.07 | 3.81 |
| ARL8B | ADP-ribosylation factor-like 8B | 4.21 | 3.04 | 4.31 |
| ASNSD1 | Asparagine synthetase domain containing 1 | 2.17 | 5.44 |  |
| ATAD2B | ATPase family, AAA domain containing 2B | 4.81 |  | 5.23 |
| ATE1 | Arginyltransferase 1 |  | 3.41 | 3.99 |
| AVL9 | AVL9 homolog (S. cerevisiase) | 2.83 | 4.16 |  |
| B4GALT6 | UDP-Gal | 2.34 | 3.50 |  |
| BAG4 | BCL2-associated athanogene 4 |  | 3.62 | 4.37 |
| C3orf58 | Chromosome 3 open reading frame 58 | 2.33 |  | 4.09 |
| C5orf28 | Chromosome 5 open reading frame 28 |  | 5.25 | 3.11 |
| C6orf211 | Chromosome 6 open reading frame 211 |  | 3.00 | 3.26 |
| C9orf72 | Chromosome 9 open reading frame 72 | 2.83 |  | 4.11 |
| CASC2 | Cancer susceptibility candidate 2 | 4.76 | 4.03 |  |
| CASP10 | Caspase 10, apoptosis-related cysteine peptidase |  | 3.16 | 3.14 |
| CCDC6 | Coiled-coil domain containing 6 | 4.49 | 3.92 |  |
| CCNY | Cyclin Y |  | 2.84 | 2.76 |
| CDC14A | CDC14 cell division cycle 14 homolog A (S. cerevisiae) | 3.80 | 3.07 |  |
| CDC40 | Cell division cycle 40 homolog (S. cerevisiae) | 4.41 | 3.83 | 4.30 |
| CDCA7 | Cell division cycle associated 7 | 4.27 | 2.12 |  |
| CHEK1 | CHK1 checkpoint homolog (S. pombe) | 3.98 | 2.50 |  |
| CHMP2B | Chromatin modifying protein 2B | 4.38 | 3.28 |  |
| CHUK | Conserved helix-loop-helix ubiquitous kinase |  | 3.34 | 3.60 |
| COX15 | COX15 homolog, cytochrome c oxidase assembly protein (yeast) | 4.05 |  | 2.90 |
| CPEB4 | Cytoplasmic polyadenylation element binding protein 4 |  | 3.09 | 3.54 |
| CPS1 | Carbamoyl-phosphate synthetase 1, mitochondrial | 3.10 | 3.98 |  |
| CREBZF | CREB/ATF bZIP transcription factor |  | 3.61 | 3.09 |
| CSNK1G1 | KIAA0101 | 2.26 | 2.55 | 2.70 |
| CUL4B | Cullin 4B |  | 4.23 | 4.51 |
| CYB561D1 | Cytochrome b-561 domain containing 1 | 3.46 | 2.35 |  |
| DDX21 | DEAD (Asp-Glu-Ala-Asp) box polypeptide 21 | 2.54 | 2.21 | 3.31 |
| DDX3X | DEAD (Asp-Glu-Ala-Asp) box polypeptide 3, X-linked | 2.03 | 3.66 | 3.82 |
| DMXL1 | Dmx-like 1 |  | 3.18 | 3.52 |
| DNAJC16 | Transcribed locus | 3.27 | 3.53 |  |
| E2F5 | E2F transcription factor 5, p130-binding | 3.29 | 2.78 |  |
| ELL2 | Elongation factor, RNA polymerase II, 2 |  | 4.08 | 3.47 |
| ELOVL7 | ELOVL family member 7, elongation of long chain fatty acids (yeast) | 3.01 |  | 3.09 |
| EPB41L4B | Erythrocyte membrane protein band 4.1 like 4B | 3.52 | 3.30 |  |
| ERCC8 | Excision repair cross-complementing rodent repair deficiency, complementation group 8 | 3.08 | 4.26 |  |
| ESRP1 | Epithelial splicing regulatory protein 1 | 3.24 | 3.44 |  |
| EXOC8 | Exocyst complex component 8 | 3.57 |  | 5.43 |
| F2RL1 | Coagulation factor II (thrombin) receptor-like 1 |  | 4.71 | 3.05 |
| FAM105A | Family with sequence similarity 105, member A | 4.54 |  | 4.25 |
| FAM178A | Family with sequence similarity 178, member A |  | 4.51 | 3.76 |
| FAM35A | Family with sequence similarity 35, member A |  | 2.56 | 5.09 |
| FAM73A | Family with sequence similarity 73, member A | 3.73 | 2.91 |  |
| FAS | Fas (TNF receptor superfamily, member 6) |  | 4.37 | 4.44 |
| FBXL17 | F-box and leucine-rich repeat protein 17 | 3.67 | 2.17 |  |
| FECH | Ferrochelatase (protoporphyria) | 4.62 | 3.32 |  |
| FGFR1OP2 | FGFR1 oncogene partner 2 | 4.70 |  | 3.79 |
| FNBP1L | Formin binding protein 1-like | 6.16 | 4.81 |  |
| FUBP3 | FUSE binding protein 3 (FBP3) | 4.15 | 3.75 |  |
| FZD3 | Frizzled homolog 3 (Drosophila) | 3.74 | 3.89 |  |
| GPR137C | G protein-coupled receptor 137C | 4.70 |  | 3.72 |
| HAUS6 | HAUS augmin-like complex, subunit 6 | 3.34 | 2.14 | 1.88 |
| HIPK1 | Homeodomain interacting protein kinase 1 | 3.65 | 2.60 |  |
| HLTF | Helicase-like transcription factor |  | 3.52 | 3.26 |
| HMGCR | Transcribed locus | 3.77 |  | 2.34 |
| HMGCR | Transcribed locus |  | 2.73 | 4.01 |
| HMGCR | Transcribed locus | 3.48 | 4.60 |  |
| IDE | Insulin-degrading enzyme | 3.43 | 4.25 |  |
| IKBKAP | Inhibitor of kappa light polypeptide gene enhancer in B-cells, kinase complex-associated protein | 2.99 |  | 3.15 |
| IKBKAP | Inhibitor of kappa light polypeptide gene enhancer in B-cells, kinase complex-associated protein | 2.77 | 5.69 |  |
| IL15 | interleukin 15 | 4.21 | 3.95 |  |
| IL7 | Interleukin 7 |  | 3.24 | 4.78 |
| INO80D | INO80 complex subunit D |  | 3.60 | 3.23 |
| IPO7 | importin 7 | 3.92 |  | 2.78 |
| KAT2B | K(lysine) acetyltransferase 2B |  | 3.06 | 3.31 |
| KATNAL1 | Katanin p60 subunit A-like 1 |  | 4.51 | 2.76 |
| KBTBD8 | Kelch repeat and BTB (POZ) domain containing 8 | 2.81 | 4.28 | 2.18 |
| KCTD18 | potassium channel tetramerisation domain containing 18 |  | 3.88 | 2.79 |
| KIAA1715 | KIAA1715 | 3.04 |  | 2.34 |
| KITLG | KIT ligand | 2.80 |  | 3.02 |
| KLF3 | Kruppel-like factor 3 (basic) | 3.45 | 3.74 |  |
| LMBRD2 | LMBR1 domain containing 2 | 3.82 |  | 4.30 |
| LPGAT1 | lysophosphatidylglycerol acyltransferase 1 | 5.33 |  | 3.33 |
| LRRC1 | leucine rich repeat containing 1 | 2.78 |  | 4.31 |
| LSM11 | LSM11, U7 small nuclear RNA associated |  | 4.87 | 3.43 |
| MAPRE2 | Microtubule-associated protein, RP/EB family, member 2 | 4.12 | 2.97 |  |
| MBNL3 | Muscleblind-like 3 (Drosophila) | 4.11 | 4.42 |  |
| MEF2C | myocyte enhancer factor 2C | 4.03 |  | 5.03 |
| MFSD1 | Major facilitator superfamily domain containing 1 | 4.32 | 3.64 | 3.45 |
| MIER1 | mesoderm induction early response 1 homolog (Xenopus laevis) |  | 3.95 | 2.02 |
| MKLN1 | Muskelin 1, intracellular mediator containing kelch motifs |  | 4.66 | 5.36 |
| MLLT3 | Myeloid/lymphoid or mixed-lineage leukemia (trithorax homolog, Drosophila); translocated to, 3 | 3.48 | 3.23 |  |
| MRAP2 | Melanocortin 2 receptor accessory protein 2 | 2.98 | 4.09 | 4.02 |
| MTAP | Methylthioadenosine phosphorylase | 4.42 | 3.52 |  |
| NBN | Nibrin |  | 3.56 | 2.34 |
| NDUFA5 | NADH dehydrogenase (ubiquinone) 1 alpha subcomplex, 5, 13kDa | 5.95 | 2.67 |  |
| NEBL | Nebulette | 4.77 | 2.91 |  |
| NEK1 | NIMA (never in mitosis gene a)-related kinase 1 | 3.85 | 3.59 |  |
| NETO2 | Neuropilin (NRP) and tolloid (TLL)-like 2 | 4.99 |  | 5.76 |
| NIN | Ninein (GSK3B interacting protein) | 2.79 | 5.06 | 2.97 |
| NLK | Nemo-like kinase |  | 3.88 | 4.19 |
| NUFIP2 | Nuclear fragile X mental retardation protein interacting protein 2 | 3.09 | 2.97 | 4.42 |
| OSBPL8 | Oxysterol binding protein-like 8 | 3.77 | 3.97 |  |
| OTUD6B | OTU domain containing 6B | 2.20 | 2.94 |  |
| PABPC3 | poly(A) binding protein, cytoplasmic 3 | 3.73 | 5.16 |  |
| PARD6B | Par-6 partitioning defective 6 homolog beta (C. elegans) | 3.12 | 1.80 | 3.78 |
| PDE8A | Phosphodiesterase 8A | 2.71 |  | 3.23 |
| PDIK1L | PDLIM1 interacting kinase 1 like |  | 4.31 | 2.58 |
| PDLIM5 | PDZ and LIM domain 5 | 3.65 | 2.69 |  |
| PGAP1 | Post-GPI attachment to proteins 1 | 4.32 | 4.41 |  |
| PHF20L1 | PHD finger protein 20-like 1 | 2.68 | 3.82 |  |
| PIK3R1 | Phosphoinositide-3-kinase, regulatory subunit 1 (alpha) |  | 3.93 | 3.58 |
| PKIA | Protein kinase (cAMP-dependent, catalytic) inhibitor alpha | 4.00 |  | 2.22 |
| PLEKHF2 | Pleckstrin homology domain containing, family F (with FYVE domain) member 2 |  | 4.22 | 4.29 |
| POLR1B | polymerase (RNA) I polypeptide B, 128kDa |  | 4.62 | 2.69 |
| POT1 | POT1 protection of telomeres 1 homolog (S. pombe) |  | 3.38 | 3.73 |
| PQLC3 | PQ loop repeat containing 3 | 4.46 | 4.43 |  |
| PRDM1 | PR domain containing 1, with ZNF domain |  | 3.32 | 4.23 |
| PRDM1 | PR domain containing 1, with ZNF domain | 3.92 | 3.32 | 4.59 |
| PRDM1 | PR domain containing 1, with ZNF domain | 5.32 | 4.42 |  |
| PRKAB2 | Protein kinase, AMP-activated, beta 2 non-catalytic subunit | 4.55 | 4.74 |  |
| PRRG4 | Proline rich Gla (G-carboxyglutamic acid) 4 (transmembrane) | 3.33 | 4.29 |  |
| PSMD7 | Proteasome (prosome, macropain) 26S subunit, non-ATPase, 7 | 5.37 |  | 3.01 |
| PSME4 | Proteasome (prosome, macropain) activator subunit 4 |  | 2.48 | 3.71 |
| RAI14 | Retinoic acid induced 14 | 3.12 |  | 4.96 |
| RALGPS2 | Ral GEF with PH domain and SH3 binding motif 2 | 3.27 | 2.49 |  |
| RANBP2 | RAN binding protein 2 |  | 3.29 | 4.03 |
| RB1 | Retinoblastoma 1 | 3.16 |  | 2.50 |
| RB1 | Retinoblastoma 1 | 2.51 |  | 3.59 |
| RB1 | Retinoblastoma 1 | 3.65 | 5.33 |  |
| RBM18 | RNA binding motif protein 18 | 2.64 | 3.66 |  |
| RBM18 | RNA binding motif protein 18 | 4.07 |  | 4.96 |
| RFX3 | Regulatory factor X, 3 (influences HLA class II expression) | 4.13 |  | 4.72 |
| RHOBTB3 | Rho-related BTB domain containing 3 | 5.45 |  | 5.20 |
| RIOK3 | Transcribed locus |  | 3.39 | 4.91 |
| RWDD3 | RWD domain containing 3 | 3.08 |  | 3.62 |
| SAMD8 | Sterile alpha motif domain containing 8 |  | 5.46 | 4.21 |
| SATL1 | Spermidine/spermine N1-acetyl transferase-like 1 | 4.63 | 3.86 |  |
| SELT | Selenoprotein T |  | 4.85 | 2.53 |
| SENP2 | SUMO1/sentrin/SMT3 specific peptidase 2 | 4.62 |  | 5.47 |
| SEPT10 | Septin 10 | 2.41 | 2.00 | 3.03 |
| SGK3 | serum/glucocorticoid regulated kinase family, member 3 | 2.34 | 4.14 |  |
| SGK3 | Transcribed locus |  | 4.43 | 3.87 |
| SHOX2 | Short stature homeobox 2 | 4.47 |  | 3.96 |
| SLC25A24 | solute carrier family 25 (mitochondrial carrier; phosphate carrier), member 24 | 3.10 | 4.33 |  |
| SLC30A5 | Solute carrier family 30 (zinc transporter), member 5 | 5.08 | 4.08 |  |
| SLC35A1 | Solute carrier family 35 (CMP-sialic acid transporter), member A1 |  | 2.84 | 3.36 |
| SLC35A3 | Solute carrier family 35 (UDP-N-acetylglucosamine (UDP-GlcNAc) transporter), member A3 | 3.64 | 4.10 | 4.07 |
| SLC35B3 | Solute carrier family 35, member B3 | 3.34 |  | 3.00 |
| SLC35B4 | Solute carrier family 35, member B4 | 3.65 | 3.26 | 3.05 |
| SLMAP | Sarcolemma associated protein |  | 5.78 | 3.26 |
| SNAPC3 | Small nuclear RNA activating complex, polypeptide 3, 50kDa | 4.46 |  | 4.51 |
| SPAG9 | Sperm associated antigen 9 | 2.95 | 3.29 | 3.22 |
| SPATA13 | spermatogenesis associated 13 | 3.25 |  | 2.92 |
| SPTLC1 | Serine palmitoyltransferase, long chain base subunit 1 | 2.45 |  | 6.41 |
| SPTLC1 | Serine palmitoyltransferase, long chain base subunit 1 | 3.69 |  | 5.49 |
| SS18L1 | Synovial sarcoma translocation gene on chromosome 18-like 1 | 4.22 |  | 3.52 |
| STRN3 | Striatin, calmodulin binding protein 3 |  | 3.31 | 3.12 |
| STXBP5 | Syntaxin binding protein 5 (tomosyn) | 4.58 |  | 3.30 |
| SVIP | Small VCP/p97-interacting protein | 2.26 | 4.51 |  |
| SWAP70 | SWAP-70 protein |  | 4.24 | 5.27 |
| TAF13 | TAF13 RNA polymerase II, TATA box binding protein (TBP)-associated factor, 18kDa | 3.39 | 8.30 |  |
| TAP2 | Transporter 2, ATP-binding cassette, sub-family B (MDR/TAP) |  | 3.75 | 4.26 |
| TC2N | Tandem C2 domains, nuclear | 3.95 | 3.55 |  |
| TEAD1 | TEA domain family member 1 (SV40 transcriptional enhancer factor) | 6.75 |  | 4.34 |
| TGDS | TDP-glucose 4,6-dehydratase | 4.03 | 3.18 |  |
| TGFBR1 | Transforming growth factor, beta receptor 1 |  | 3.50 | 3.79 |
| THAP6 | THAP domain containing 6 |  | 3.43 | 2.50 |
| TMEM144 | Transmembrane protein 144 | 2.37 | 2.12 |  |
| TMEM33 | Transmembrane protein 33 |  | 3.25 | 4.09 |
| TMEM87A | Transcribed locus |  | 4.18 | 3.04 |
| TMPO | Thymopoietin | 4.54 | 2.40 |  |
| TMTC3 | Transmembrane and tetratricopeptide repeat containing 3 | 2.45 | 3.31 |  |
| TNFRSF19 | Tumor necrosis factor receptor superfamily, member 19 |  | 2.85 | 4.85 |
| TNFRSF19 | Tumor necrosis factor receptor superfamily, member 19 | 7.24 |  | 3.98 |
| TOMM20 | translocase of outer mitochondrial membrane 20 homolog (yeast) |  | 4.40 | 2.79 |
| TOMM70A | Translocase of outer mitochondrial membrane 70 homolog A (S. cerevisiae) | 5.43 | 4.17 |  |
| TOPORS | Topoisomerase I binding, arginine/serine-rich |  | 3.40 | 2.85 |
| TRIM2 | Tripartite motif-containing 2 | 3.85 | 3.64 |  |
| TROVE2 | TROVE domain family, member 2 | 2.50 | 3.10 |  |
| UBR7 | ubiquitin protein ligase E3 component n-recognin 7 (putative) | 4.48 |  | 4.99 |
| USP25 | Ubiquitin specific peptidase 25 | 4.70 | 3.66 |  |
| USP53 | Ubiquitin specific peptidase 53 | 3.38 | 7.67 |  |
| VMA21 | VMA21 vacuolar H+-ATPase homolog (S. cerevisiae) | 4.13 | 2.58 |  |
| VPS41 | Vacuolar protein sorting 41 homolog (S. cerevisiae) | 2.77 | 3.67 |  |
| VTA1 | Vps20-associated 1 homolog (S. cerevisiae) | 3.68 | 3.24 |  |
| WDR36 | WD repeat domain 36 | 6.47 | 3.25 |  |
| WDR41 | WD repeat domain 41 | 3.08 | 5.07 |  |
| WDR72 | WD repeat domain 72 | 3.10 |  | 4.28 |
| WDYHV1 | WDYHV motif containing 1 | 4.07 | 3.99 |  |
| WWP1 | WW domain containing E3 ubiquitin protein ligase 1 |  | 4.47 | 3.30 |
| ZC3HAV1 | Zinc finger CCCH-type, antiviral 1 | 3.83 | 3.41 |  |
| ZDHHC21 | zinc finger, DHHC-type containing 21 | 3.64 | 2.38 | 3.95 |
| ZFP30 | Zinc finger protein 607 | 5.09 |  | 4.46 |
| ZMYM1 | Zinc finger, MYM-type 1 |  | 3.32 | 3.19 |
| ZNF365 | zinc finger protein 365 | 3.97 | 3.49 |  |
| ZNF398 | zinc finger protein 398 |  | 3.44 | 3.59 |
| ZNF558 | zinc finger protein 558 |  | 3.49 | 4.09 |
| ZNF654 | zinc finger protein 654 | 3.72 | 3.54 | 4.05 |
