# Supplementary material for: Genome-wide identification of Wig-1 mRNA targets by RIP-Seq analysis
Source: Oncotarget. 2015 Dec 11;7(2):1895–911. doi: 10.18632/oncotarget.6557 (PMC4811505; doi:10.18632/oncotarget.6557)
Supplement: Supplementary file 6 [file oncotarget-07-1895-s006.doc]

| Supplementary Table S6: Gene Ontology Analysis of Wig-1-bound RNAs by DAVID   |  | **Category** | **GO ID** | **Count** | **%** | **p-value** | | --- | --- | --- | --- | --- | --- | |  |  |  |  |  |  | |  | **Molecular Function** |  |  |  |  | | 1 | lamin binding | GO:0005521 | 3 | 1.1 | 1.84E-03 | | 2 | catalytic activity | GO:0003824 | 90 | 34.5 | 7.61E-03 | | 3 | magnesium ion binding | GO:0000287 | 14 | 5.4 | 1.01E-02 | | 4 | C-acyltransferase activity | GO:0016408 | 3 | 1.1 | 2.00E-02 | | 5 | acyltransferase activity | GO:0008415 | 8 | 3.1 | 2.17E-02 | | 6 | transferase activity, transferring acyl groups other than amino-acyl groups | GO:0016747 | 8 | 3.1 | 2.27E-02 | | 7 | transferase activity | GO:0016740 | 35 | 13.4 | 2.49E-02 | | 8 | transferase activity, transferring acyl groups | GO:0016746 | 8 | 3.1 | 2.71E-02 | | 9 | methylenetetrahydrofolate dehydrogenase (NAD+) activity | GO:0004487 | 2 | 0.8 | 2.74E-02 | | 10 | methenyltetrahydrofolate cyclohydrolase activity | GO:0004477 | 2 | 0.8 | 4.08E-02 | | 11 | methylenetetrahydrofolate dehydrogenase activity | GO:0004486 | 2 | 0.8 | 4.08E-02 | | 12 | translation factor activity, nucleic acid binding | GO:0008135 | 5 | 1.9 | 4.67E-02 | |  |  |  |  |  |  | |  | **Biological processes** |  |  |  |  | | 1 | ER to Golgi vesicle-mediated transport | GO:0006888 | 6 | 2.3 | 3.38E-04 | | 2 | cellular protein metabolic process | GO:0044267 | 54 | 20.7 | 5.28E-04 | | 3 | Golgi vesicle transport | GO:0048193 | 9 | 3.4 | 6.49E-04 | | 4 | cellular metabolic process | GO:0044237 | 119 | 45.6 | 1.46E-03 | | 5 | protein transport | GO:0015031 | 23 | 8.8 | 1.64E-03 | | 6 | establishment of protein localization | GO:0045184 | 23 | 8.8 | 1.84E-03 | | 7 | protein metabolic process | GO:0019538 | 59 | 22.6 | 2.35E-03 | | 8 | positive regulation of I-kappaB kinase/NF-kappaB cascade | GO:0043123 | 7 | 2.7 | 2.88E-03 | | 9 | macromolecule localization | GO:0033036 | 28 | 10.7 | 3.69E-03 | | 10 | positive regulation of signal transduction | GO:0009967 | 12 | 4.6 | 3.95E-03 | | 11 | monocarboxylic acid metabolic process | GO:0032787 | 12 | 4.6 | 4.55E-03 | | 12 | protein localization | GO:0008104 | 24 | 9.2 | 4.63E-03 | | 13 | regulation of I-kappaB kinase/NF-kappaB cascade | GO:0043122 | 7 | 2.7 | 4.68E-03 | | 14 | carboxylic acid biosynthetic process | GO:0046394 | 8 | 3.1 | 7.50E-03 | | 15 | organic acid biosynthetic process | GO:0016053 | 8 | 3.1 | 7.50E-03 | | 16 | cell cycle phase | GO:0022403 | 14 | 5.4 | 7.63E-03 | | 17 | protein ubiquitination | GO:0016567 | 7 | 2.7 | 7.79E-03 | | 18 | mitotic cell cycle | GO:0000278 | 13 | 5.0 | 7.81E-03 | | 19 | M phase | GO:0000279 | 12 | 4.6 | 8.68E-03 | | 20 | positive regulation of cell communication | GO:0010647 | 12 | 4.6 | 8.68E-03 | |  |  |  |  |  |  | |  | **Cellular component** |  |  |  |  | | 1 | cytoplasm | GO:0005737 | 147 | 56.3 | 1.64E-10 | | 2 | membrane-bounded organelle | GO:0043227 | 154 | 59.0 | 7.96E-10 | | 3 | intracellular | GO:0005622 | 189 | 72.4 | 1.22E-09 | | 4 | endoplasmic reticulum | GO:0005783 | 37 | 14.2 | 2.83E-08 | | 5 | endomembrane system | GO:0012505 | 26 | 10.0 | 6.70E-05 | | 6 | organelle part | GO:0044422 | 84 | 32.2 | 1.25E-04 | | 7 | mitochondrion | GO:0005739 | 30 | 11.5 | 3.97E-04 | | 8 | organelle membrane | GO:0031090 | 30 | 11.5 | 4.54E-04 | | 9 | nuclear envelope | GO:0005635 | 10 | 3.8 | 2.05E-03 | | 10 | organelle envelope | GO:0031967 | 19 | 7.3 | 2.14E-03 | | 11 | protein complex | GO:0043234 | 52 | 19.9 | 3.49E-03 | | 12 | nuclear pore | GO:0005643 | 6 | 2.3 | 4.49E-03 | |  |  |  |  |  |  | | Count: number of genes involved in the annotation; %: % of gene in the annotation over total genes of the list; | | | | | |   p-value is calculated by Fisher Exact test. | |  | |  |
| --- | --- | --- | --- | --- | --- | --- | --- | --- | --- | --- | --- | --- | --- | --- | --- | --- | --- | --- | --- | --- | --- | --- | --- | --- | --- | --- | --- | --- | --- | --- | --- | --- | --- | --- | --- | --- | --- | --- | --- | --- | --- | --- | --- | --- | --- | --- | --- | --- | --- | --- | --- | --- | --- | --- | --- | --- | --- | --- | --- | --- | --- | --- | --- | --- | --- | --- | --- | --- | --- | --- | --- | --- | --- | --- | --- | --- | --- | --- | --- | --- | --- | --- | --- | --- | --- | --- | --- | --- | --- | --- | --- | --- | --- | --- | --- | --- | --- | --- | --- | --- | --- | --- | --- | --- | --- | --- | --- | --- | --- | --- | --- | --- | --- | --- | --- | --- | --- | --- | --- | --- | --- | --- | --- | --- | --- | --- | --- | --- | --- | --- | --- | --- | --- | --- | --- | --- | --- | --- | --- | --- | --- | --- | --- | --- | --- | --- | --- | --- | --- | --- | --- | --- | --- | --- | --- | --- | --- | --- | --- | --- | --- | --- | --- | --- | --- | --- | --- | --- | --- | --- | --- | --- | --- | --- | --- | --- | --- | --- | --- | --- | --- | --- | --- | --- | --- | --- | --- | --- | --- | --- | --- | --- | --- | --- | --- | --- | --- | --- | --- | --- | --- | --- | --- | --- | --- | --- | --- | --- | --- | --- | --- | --- | --- | --- | --- | --- | --- | --- | --- | --- | --- | --- | --- | --- | --- | --- | --- | --- | --- | --- | --- | --- | --- | --- | --- | --- | --- | --- | --- | --- | --- | --- | --- | --- | --- | --- | --- | --- | --- | --- | --- | --- | --- | --- | --- | --- | --- | --- | --- | --- | --- | --- | --- | --- | --- | --- | --- | --- | --- | --- | --- | --- | --- | --- | --- | --- | --- | --- | --- | --- | --- | --- | --- | --- | --- | --- | --- | --- | --- | --- | --- | --- | --- | --- | --- | --- | --- | --- | --- | --- | --- | --- | --- | --- | --- | --- | --- | --- | --- | --- | --- | --- | --- | --- | --- | --- | --- | --- | --- | --- | --- | --- |
|  |  |  |  | |
