# Supplementary material for: Genome-wide identification of Wig-1 mRNA targets by RIP-Seq analysis
Source: Oncotarget. 2015 Dec 11;7(2):1895–911. doi: 10.18632/oncotarget.6557 (PMC4811505; doi:10.18632/oncotarget.6557)
Supplement: Supplementary file 8 [file oncotarget-07-1895-s008.doc]

| Supplementary Table S8: List of the 133 Reactome pathways enriched at significant level of FDR <0.01   | **Rank** | **Pathway** | **Network enrichment  p-value** | | --- | --- | --- | | 1 | Cell Cycle Mitotic | 0.00E+00 | | 2 | Mitotic M-M/G1 Phases | 0.00E+00 | | 3 | Mitotic Prometaphase | 1.39E-80 | | 4 | G1/S Transition | 2.30E-63 | | 5 | S Phase | 4.00E-63 | | 6 | HIV Infection | 8.04E-63 | | 7 | Cell Cycle Checkpoints | 4.08E-60 | | 8 | Host Interactions Of HIV Factors | 1.06E-53 | | 9 | Synthesis Of DNA | 1.17E-51 | | 10 | Metabolism Of RNA | 2.83E-48 | | 11 | Regulation Of APC Activators Between G1/S And Early Anaphase | 5.29E-48 | | 12 | Nep Ns2 Interacts With The Cellular Export Machinery | 3.67E-45 | | 13 | Rev Mediated Nuclear Export Of HIV1 Rna | 1.65E-44 | | 14 | Transport Of Mature mRNA Derived From An Intron Containing Transcript | 2.51E-43 | | 15 | Transport Of Ribonucleoproteins Into The Host Nucleus | 2.95E-43 | | 16 | Transport Of The Slbp Independent Mature mRNA | 3.46E-43 | | 17 | Nuclear Import Of Rev Protein | 7.60E-43 | | 18 | Late Phase Of HIV Life Cycle | 2.26E-41 | | 19 | Dna Replication Pre Initiation | 2.03E-40 | | 20 | HIV Life Cycle | 2.24E-40 | | 21 | Regulation Of Glucokinase By Glucokinase Regulatory Protein | 1.48E-39 | | 22 | Cdc20 Phospho Apc Mediated Degradation Of Cyclin A | 1.24E-38 | | 23 | Glucose Transport | 3.14E-38 | | 24 | snRNP Assembly | 5.84E-37 | | 25 | Vpr Mediated Nuclear Import Of Pics | 2.19E-36 | | 26 | Processing Of Capped Intron Containing Pre mRNA | 2.20E-35 | | 27 | G2/M Checkpoints | 3.93E-35 | | 28 | Cyclin E Associated Events During G1/S Transition | 1.12E-34 | | 29 | Orc1 Removal From Chromatin | 1.51E-34 | | 30 | Activation Of Atr In Response To Replication Stress | 1.67E-32 | | 31 | M/G1 Transition | 5.79E-32 | | 32 | Signaling By Wnt | 2.14E-31 | | 33 | Scf Skp2 Mediated Degradation Of P27 P21 | 3.66E-30 | | 34 | Scf Beta Trcp Mediated Degradation Of Emi1 | 8.39E-29 | | 35 | Dna Repair | 1.10E-28 | | 36 | Autodegradation Of Cdh1 By Cdh1 Apc | 1.32E-27 | | 37 | Dna Strand Elongation | 9.22E-27 | | 38 | Global Genomic Ner | 9.64E-26 | | 39 | Vif Mediated Degradation Of Apobec3G | 2.55E-25 | | 40 | Cdt1 Association With The Cdc6 Orc Origin Complex | 1.24E-24 | | 41 | Regulation Of Ornithine Decarboxylase | 2.35E-23 | | 42 | P53 Independent Dna Damage Response | 1.71E-22 | | 43 | Activation Of The Pre Replicative Complex | 3.52E-22 | | 44 | Formation And Maturation Of mRNA Transcript | 9.90E-22 | | 45 | RNA Polymerase Ii Transcription | 1.56E-21 | | 46 | Elongation And Processing Of Capped Transcripts | 1.65E-20 | | 47 | Metabolism Of Carbohydrates | 5.86E-20 | | 48 | Membrane Trafficking | 2.92E-19 | | 49 | Metabolism Of Amino Acids | 4.16E-19 | | 50 | Nucleotide Excision Repair | 3.55E-18 | | 51 | Cyclin A1 Associated Events During G2/M Transition | 4.29E-18 | | 52 | Transcription Coupled Ner | 2.80E-17 | | 53 | Inactivation Of Apc Via Direct Inhibition Of The Apcomplex | 6.90E-17 | | 54 | Stabilization Of P53 | 1.67E-16 | | 55 | Transmembrane Transport Of Small Molecules | 3.76E-16 | | 56 | mRNA 3 End Processing | 4.47E-16 | | 57 | Dual Incision Reaction In Gg Ner | 6.33E-16 | | 58 | Metabolism Of mRNA | 5.97E-15 | | 59 | mRNA Splicing | 7.47E-15 | | 60 | Repair Synthesis Of Patch 27 30 Bases Long By Dna Polymerase | 8.21E-15 | | 61 | Lagging Strand Synthesis | 1.02E-14 | | 62 | E2F Mediated Regulation Of Dna Replication | 2.06E-14 | | 63 | Apoptosis | 9.41E-14 | | 64 | Unwinding Of Dna | 1.22E-13 | | 65 | Extension Of Telomeres | 4.56E-13 | | 66 | Phosphorylation Of The Apc | 9.80E-13 | | 67 | Regulation Of Pyruvate Dehydrogenase Complex | 2.89E-11 | | 68 | Metablism Of Nucleotides | 1.05E-10 | | 69 | E2F Enabled Inhibition Of Pre Replication Complex Formation | 1.26E-10 | | 70 | Pyruvate Metabolism | 3.15E-10 | | 71 | Apcdc20 Mediated Degradation Of Cyclin B | 3.47E-10 | | 72 | Double Strand Break Repair | 3.93E-10 | | 73 | Polymerase Switching | 5.96E-10 | | 74 | Pyruvate Metabolism And Tca Cycle | 6.49E-10 | | 75 | Clathrin Derived Vesicle Budding | 2.68E-09 | | 76 | Transcription Of The HIV Genome | 4.38E-09 | | 77 | mRNA Processing | 9.22E-09 | | 78 | Synthesis And Interconversion Of Nucleotide Di And Triphosphates | 1.48E-08 | | 79 | Deadenylation Of mRNA | 2.09E-08 | | 80 | Transcription | 2.39E-08 | | 81 | tRNA Aminoacylation | 2.48E-08 | | 82 | E2F Transcriptional Targets At G1/S | 3.71E-08 | | 83 | Conversion From Apc Cdc20 To Apc Cdh1 In Late Anaphase | 6.53E-08 | | 84 | Sphingolipid Metabolism | 7.05E-08 | | 85 | HIV1 Transcription Elongation | 7.72E-08 | | 86 | Gene Expression | 2.05E-07 | | 87 | Golgi Associated Vesicle Biogenesis | 2.29E-07 | | 88 | G2/M Transition | 3.12E-07 | | 89 | RNA Pol Ii Ctd Phosphorylation And Interaction With Ce | 3.28E-07 | | 90 | Pyrimidine Metabolism | 5.23E-07 | | 91 | RNA Polymerase I Promoter Escape | 6.11E-07 | | 92 | RNA Polymerase I Transcription Initiation | 6.73E-07 | | 93 | Cytosolic tRNA Aminoacylation | 1.13E-06 | | 94 | Homologous Recombination Repair | 1.87E-06 | | 95 | Formation Of The Early Elongation Complex | 2.99E-06 | | 96 | Citric Acid Cycle | 3.03E-06 | | 97 | RNA Polymerase I Transcription Termination | 3.46E-06 | | 98 | mRNA Splicing Minor Pathway | 4.80E-06 | | 99 | Betacatenin Phosphorylation Cascade | 5.56E-06 | | 100 | Removal Of The Flap Intermediate | 9.75E-06 | | 101 | HIV1 Transcription Initiation | 9.95E-06 | | 102 | Dual Incision Reaction In Tc Ner | 9.98E-06 | | 103 | RNA Polymerase I Chain Elongation | 1.09E-05 | | 104 | Copi Mediated Transport | 1.25E-05 | | 105 | Generic Transcription Pathway | 1.80E-05 | | 106 | mRNA Decay By 3 To 5 Exoribonuclease | 5.14E-05 | | 107 | Telomere Maintenance | 5.66E-05 | | 108 | Removal Of The Flap Intermediate From The C Strand | 7.42E-05 | | 109 | Energy Dependent Regulation Of Mtor By Lkb1 Ampk | 1.12E-04 | | 110 | Lysosome Vesicle Biogenesis | 1.17E-04 | | 111 | Association Of Tric Cct With Target Proteins During Biosynthesis | 1.45E-04 | | 112 | mRNA Decay By 5 To 3 Exoribonuclease | 2.60E-04 | | 113 | Peroxisomal Lipid Metabolism | 3.77E-04 | | 114 | Activated Ampk Stimulates Fatty Acid Oxidation In Muscle | 4.23E-04 | | 115 | Cholesterol Biosynthesis | 1.21E-03 | | 116 | MicroRNA Biogenesis | 1.49E-03 | | 117 | Cdc6 Association With The Orc:Origin Complex | 1.88E-03 | | 118 | Signaling By Tgf Beta | 2.15E-03 | | 119 | Activation Of Rac | 4.24E-03 | | 120 | Signaling By Bmp | 4.75E-03 | | 121 | Recruitment Of Numa To Mitotic Centrosomes | 4.92E-03 | | 122 | Mtor Signalling | 8.12E-03 | | 123 | Metabolism Of Lipids And Lipoproteins | 9.39E-03 | | 124 | Proteolytic Cleavage Of Snare Complex Proteins | 9.47E-03 | | 125 | Fanconi Anemia Pathway | 9.63E-03 | | 126 | Mitochondrial tRNA Aminoacylation | 9.83E-03 | | 127 | Branched Chain Amino Acid Catabolism | 1.17E-02 | | 128 | Regulation Of Ampk Activity Via Lkb1 | 1.60E-02 | | 129 | Sema3A Pak Dependent Axon Repulsion | 2.06E-02 | | 130 | Metal Ion Slc Transporters | 2.09E-02 | | 131 | Metabolism Of Vitamins And Cofactors | 2.26E-02 |  | 132 | G1 Phase | 2.30E-02 | | --- | --- | --- | | 133 | Base Excision Repair | 2.84E-02 |  |  |  |  | | | --- | --- | --- | --- | |  |  |  | | |  |  |  |  | |  |
| --- | --- | --- | --- | --- | --- | --- | --- | --- | --- | --- | --- | --- | --- | --- | --- | --- | --- | --- | --- | --- | --- | --- | --- | --- | --- | --- | --- | --- | --- | --- | --- | --- | --- | --- | --- | --- | --- | --- | --- | --- | --- | --- | --- | --- | --- | --- | --- | --- | --- | --- | --- | --- | --- | --- | --- | --- | --- | --- | --- | --- | --- | --- | --- | --- | --- | --- | --- | --- | --- | --- | --- | --- | --- | --- | --- | --- | --- | --- | --- | --- | --- | --- | --- | --- | --- | --- | --- | --- | --- | --- | --- | --- | --- | --- | --- | --- | --- | --- | --- | --- | --- | --- | --- | --- | --- | --- | --- | --- | --- | --- | --- | --- | --- | --- | --- | --- | --- | --- | --- | --- | --- | --- | --- | --- | --- | --- | --- | --- | --- | --- | --- | --- | --- | --- | --- | --- | --- | --- | --- | --- | --- | --- | --- | --- | --- | --- | --- | --- | --- | --- | --- | --- | --- | --- | --- | --- | --- | --- | --- | --- | --- | --- | --- | --- | --- | --- | --- | --- | --- | --- | --- | --- | --- | --- | --- | --- | --- | --- | --- | --- | --- | --- | --- | --- | --- | --- | --- | --- | --- | --- | --- | --- | --- | --- | --- | --- | --- | --- | --- | --- | --- | --- | --- | --- | --- | --- | --- | --- | --- | --- | --- | --- | --- | --- | --- | --- | --- | --- | --- | --- | --- | --- | --- | --- | --- | --- | --- | --- | --- | --- | --- | --- | --- | --- | --- | --- | --- | --- | --- | --- | --- | --- | --- | --- | --- | --- | --- | --- | --- | --- | --- | --- | --- | --- | --- | --- | --- | --- | --- | --- | --- | --- | --- | --- | --- | --- | --- | --- | --- | --- | --- | --- | --- | --- | --- | --- | --- | --- | --- | --- | --- | --- | --- | --- | --- | --- | --- | --- | --- | --- | --- | --- | --- | --- | --- | --- | --- | --- | --- | --- | --- | --- | --- | --- | --- | --- | --- | --- | --- | --- | --- | --- | --- | --- | --- | --- | --- | --- | --- | --- | --- | --- | --- | --- | --- | --- | --- | --- | --- | --- | --- | --- | --- | --- | --- | --- | --- | --- | --- | --- | --- | --- | --- | --- | --- | --- | --- | --- | --- | --- | --- | --- | --- | --- | --- | --- | --- | --- | --- | --- | --- | --- | --- | --- | --- | --- | --- | --- | --- | --- | --- | --- | --- | --- | --- | --- | --- | --- | --- | --- | --- | --- | --- | --- | --- | --- | --- | --- | --- | --- | --- | --- | --- | --- | --- | --- | --- | --- | --- | --- | --- | --- | --- | --- | --- | --- | --- | --- | --- | --- | --- | --- | --- | --- | --- |
|  | |
