# Supplementary material for: Genome-wide identification of Wig-1 mRNA targets by RIP-Seq analysis
Source: Oncotarget. 2015 Dec 11;7(2):1895–911. doi: 10.18632/oncotarget.6557 (PMC4811505; doi:10.18632/oncotarget.6557)
Supplement: Supplementary file 10 [file oncotarget-07-1895-s010.doc]

| Supplementary Table S10: List of the 286 RNAs included in the unbound group | | |
| --- | --- | --- |
| **Gene name** | **Ensembl Gene ID** | **Ref Seq** |
| LSM14A | ENSG00000257103 | NM_015578 |
| EFNA4 | ENSG00000243364 | NM_005227 |
| DHFR | ENSG00000228716 | NM_000791 |
| PGAM4 | ENSG00000226784 | NM_001029891 |
| CALM2P3 | ENSG00000215482 | NG_004858 |
| ATP5G1P6 | ENSG00000214318 | NG_028974 |
| REPIN1 | ENSG00000214022 | NM_013400 |
| RRM2P3 | ENSG00000214018 | NG_026678 |
| AP1G2 | ENSG00000213983 | NM_003917 |
| NHP2P2 | ENSG00000213786 | NG_005298 |
| GTF2H4 | ENSG00000213780 | NM_001517 |
| GAPDHP71 | ENSG00000213376 | NG_003027 |
| SNORA46 | ENSG00000207493 | NR_002978 |
| SNORA57 | ENSG00000206597 | NR_004390 |
| ZBTB48 | ENSG00000204859 | NM_005341 |
| HLA-DMA | ENSG00000204257 | NM_006120 |
| HIST2H2BF | ENSG00000203814 | NM_001024599 |
| SNORA73B | ENSG00000200087 | NR_004406 |
| C9orf114 | ENSG00000198917 | NM_016390 |
| MT1F | ENSG00000198417 | NM_005949 |
| MGEA5 | ENSG00000198408 | NM_012215 |
| ZNF544 | ENSG00000198131 | NM_014480 |
| MPZL1 | ENSG00000197965 | NM_003953 |
| HIST1H4K | ENSG00000197914 | NM_003541 |
| TEAD4 | ENSG00000197905 | NM_003213 |
| ENTPD6 | ENSG00000197586 | NM_001247 |
| WDR45 | ENSG00000196998 | NM_007075 |
| ANXA4 | ENSG00000196975 | NM_001153 |
| CASP4 | ENSG00000196954 | NM_001225 |
| PLXNB2 | ENSG00000196576 | NM_012401 |
| ANAPC7 | ENSG00000196510 | NM_016238 |
| RRP7A | ENSG00000189306 | NM_015703 |
| AC002398.9 | ENSG00000188223 | AC002398.9 |
| RTN4RL2 | ENSG00000186907 | NM_178570 |
| PRR5 | ENSG00000186654 | NM_015366 |
| UBE2H | ENSG00000186591 | NM_003344 |
| IFITM1 | ENSG00000185885 | NM_003641 |
| ZFP36L1 | ENSG00000185650 | NM_004926 |
| NELFA | ENSG00000185049 | NM_005663 |
| HIST2H2AC | ENSG00000184260 | NM_003517 |
| IRAK1 | ENSG00000184216 | NM_001569 |
| CHEK2 | ENSG00000183765 | NM_007194 |
| HIST2H2AA3 | ENSG00000183558 | NM_003516 |
| AP2A2 | ENSG00000183020 | NM_012305 |
| CNOT10 | ENSG00000182973 | NM_015442 |
| EWSR1 | ENSG00000182944 | NM_005243 |
| CMC4 | ENSG00000182712 | NM_001018024 |
| CLK3 | ENSG00000179335 | NM_003992 |
| MPI | ENSG00000178802 | NM_002435 |
| GTPBP6 | ENSG00000178605 | NM_012227 |
| FLII | ENSG00000177731 | NM_002018 |
| BNIP3 | ENSG00000176171 | NM_004052 |
| NR2F1 | ENSG00000175745 | NM_005654 |
| ATP2A2 | ENSG00000174437 | NM_001681 |
| PLK3 | ENSG00000173846 | NM_004073 |
| JUP | ENSG00000173801 | NM_002230 |
| C2orf70 | ENSG00000173557 | NM_001105519 |
| ESRRA | ENSG00000173153 | NM_004451 |
| RARG | ENSG00000172819 | NM_000966 |
| ZNF131 | ENSG00000172262 | NM_003432 |
| NME6 | ENSG00000172113 | NM_005793 |
| THOP1 | ENSG00000172009 | NM_003249 |
| CTPS1 | ENSG00000171793 | NM_001905 |
| RGS19 | ENSG00000171700 | NM_005873 |
| CXXC5 | ENSG00000171604 | NM_016463 |
| HARS | ENSG00000170445 | NM_002109 |
| MGMT | ENSG00000170430 | NM_002412 |
| RFNG | ENSG00000169733 | NM_002917 |
| EFNA1 | ENSG00000169242 | NM_004428 |
| MAP2K1 | ENSG00000169032 | NM_002755 |
| FEN1 | ENSG00000168496 | NM_004111 |
| ACOX2 | ENSG00000168306 | NM_003500 |
| HIST1H1E | ENSG00000168298 | NM_005321 |
| MAP4K2 | ENSG00000168067 | NM_004579 |
| KLK6 | ENSG00000167755 | NM_002774 |
| SERTAD3 | ENSG00000167565 | NM_013368 |
| TMEM41B | ENSG00000166471 | NM_015012 |
| NOLC1 | ENSG00000166197 | NM_004741 |
| TTC7B | ENSG00000165914 | NM_001010854 |
| KLHDC2 | ENSG00000165516 | NM_014315 |
| CLDN3 | ENSG00000165215 | NM_001306 |
| SPIDR | ENSG00000164808 | NM_001080394 |
| SAP30 | ENSG00000164105 | NM_003864 |
| POC1A | ENSG00000164087 | NM_015426 |
| PGRMC2 | ENSG00000164040 | NM_006320 |
| MEMO1 | ENSG00000162959 | NM_015955 |
| FUBP1 | ENSG00000162613 | NM_003902 |
| STX5 | ENSG00000162236 | NM_003164 |
| CPSF4 | ENSG00000160917 | NM_006693 |
| FAM189B | ENSG00000160767 | NM_006589 |
| CHTOP | ENSG00000160679 | NM_015607 |
| MED27 | ENSG00000160563 | NM_004269 |
| ST6GALNAC6 | ENSG00000160408 | NM_013443 |
| ATP6V0D1 | ENSG00000159720 | NM_004691 |
| TSC22D3 | ENSG00000157514 | NM_004089 |
| HNRNPU | ENSG00000153187 | NM_004501 |
| PDCD4 | ENSG00000150593 | NM_014456 |
| SERPINH1 | ENSG00000149257 | NM_001235 |
| ADM | ENSG00000148926 | NM_001124 |
| RSU1 | ENSG00000148484 | NM_012425 |
| EBAG9 | ENSG00000147654 | NM_004215 |
| POMZP3 | ENSG00000146707 | NM_012230 |
| NFKBIE | ENSG00000146232 | NM_004556 |
| ABT1 | ENSG00000146109 | NM_013375 |
| TNFRSF21 | ENSG00000146072 | NM_014452 |
| GTF2H2 | ENSG00000145736 | NM_001515 |
| ECE2 | ENSG00000145194 | NM_014693 |
| EIF2B5 | ENSG00000145191 | NM_003907 |
| DYNC1LI1 | ENSG00000144635 | NM_016141 |
| PARP1 | ENSG00000143799 | NM_001618 |
| UBAP2L | ENSG00000143569 | NM_014847 |
| ALDH9A1 | ENSG00000143149 | NM_000696 |
| PLK4 | ENSG00000142731 | NM_014264 |
| ANAPC11 | ENSG00000141552 | NM_016476 |
| CSNK1D | ENSG00000141551 | NM_001893 |
| EIF4A3 | ENSG00000141543 | NM_014740 |
| RAB40B | ENSG00000141542 | NM_006822 |
| SLC16A3 | ENSG00000141526 | NM_004207 |
| TMC6 | ENSG00000141524 | NM_007267 |
| ARHGDIA | ENSG00000141522 | NM_004309 |
| ARRB2 | ENSG00000141480 | NM_004313 |
| C18orf8 | ENSG00000141452 | NM_013326 |
| IMPA2 | ENSG00000141401 | NM_014214 |
| AFG3L2 | ENSG00000141385 | NM_006796 |
| SS18 | ENSG00000141380 | NM_005637 |
| PTRH2 | ENSG00000141378 | NM_016077 |
| CLTC | ENSG00000141367 | NM_004859 |
| NPEPPS | ENSG00000141279 | NM_006310 |
| TOB1 | ENSG00000141232 | NM_005749 |
| TOM1L1 | ENSG00000141198 | NM_005486 |
| DDX52 | ENSG00000141141 | NM_007010 |
| NOB1 | ENSG00000141101 | NM_014062 |
| TCF25 | ENSG00000141002 | NM_014972 |
| NDUFB10 | ENSG00000140990 | NM_004548 |
| RPS2 | ENSG00000140988 | NM_002952 |
| MBTPS1 | ENSG00000140943 | NM_003791 |
| NOL3 | ENSG00000140939 | NM_003946 |
| GCSH | ENSG00000140905 | NM_004483 |
| NUDT7 | ENSG00000140876 | NM_001105663 |
| KIFC3 | ENSG00000140859 | NM_005550 |
| DHX38 | ENSG00000140829 | NM_014003 |
| CDR2 | ENSG00000140743 | NM_001802 |
| UQCRC2 | ENSG00000140740 | NM_003366 |
| PARN | ENSG00000140694 | NM_002582 |
| SEC11A | ENSG00000140612 | NM_014300 |
| IQGAP1 | ENSG00000140575 | NM_003870 |
| FURIN | ENSG00000140564 | NM_002569 |
| MFGE8 | ENSG00000140545 | NM_005928 |
| POLG | ENSG00000140521 | NM_002693 |
| SCAMP2 | ENSG00000140497 | NM_005697 |
| ULK3 | ENSG00000140474 | NM_001099436 |
| PML | ENSG00000140464 | NM_002675 |
| USP3 | ENSG00000140455 | NM_006537 |
| WARS | ENSG00000140105 | NM_004184 |
| HADH | ENSG00000138796 | NM_005327 |
| CASP6 | ENSG00000138794 | NM_001226 |
| USO1 | ENSG00000138768 | NM_003715 |
| IDH1 | ENSG00000138413 | NM_005896 |
| CALHM2 | ENSG00000138172 | NM_015916 |
| NRM | ENSG00000137404 | NM_007243 |
| BPHL | ENSG00000137274 | NM_004332 |
| FPGS | ENSG00000136877 | NM_004957 |
| CDK9 | ENSG00000136807 | NM_001261 |
| DBNL | ENSG00000136279 | NM_014063 |
| DNAJB2 | ENSG00000135924 | NM_006736 |
| KRT7 | ENSG00000135480 | NM_005556 |
| PRPH | ENSG00000135406 | NM_006262 |
| ORC3 | ENSG00000135336 | NM_012381 |
| HILPDA | ENSG00000135245 | NM_013332 |
| OSTF1 | ENSG00000134996 | NM_012383 |
| ARF3 | ENSG00000134287 | NM_001659 |
| CSNK1G2 | ENSG00000133275 | NM_001319 |
| MPRIP | ENSG00000133030 | NM_015134 |
| MUTYH | ENSG00000132781 | NM_012222 |
| DPH2 | ENSG00000132768 | NM_001384 |
| MTL5 | ENSG00000132749 | NM_004923 |
| TIMM10B | ENSG00000132286 | NM_012192 |
| SLX1A | ENSG00000132207 | NM_001014999 |
| HSD17B7 | ENSG00000132196 | NM_016371 |
| PPARG | ENSG00000132170 | NM_005037 |
| DHX30 | ENSG00000132153 | NM_014966 |
| STARD3 | ENSG00000131748 | NM_006804 |
| ACLY | ENSG00000131473 | NM_001096 |
| DKC1 | ENSG00000130826 | NM_001363 |
| SLC6A8 | ENSG00000130821 | NM_005629 |
| EXOSC2 | ENSG00000130713 | NM_014285 |
| TUBGCP2 | ENSG00000130640 | NM_006659 |
| PRKCSH | ENSG00000130175 | NM_002743 |
| LDLR | ENSG00000130164 | NM_000527 |
| VGF | ENSG00000128564 | NM_003378 |
| POR | ENSG00000127948 | NM_000941 |
| KTN1 | ENSG00000126777 | NM_004986 |
| ELK1 | ENSG00000126767 | NM_005229 |
| UBA2 | ENSG00000126261 | NM_005499 |
| MAX | ENSG00000125952 | NM_002382 |
| AHNAK | ENSG00000124942 | NM_001620 |
| STAU1 | ENSG00000124214 | NM_004602 |
| NT5C3A | ENSG00000122643 | NM_016489 |
| RBBP6 | ENSG00000122257 | NM_006910 |
| ZNF639 | ENSG00000121864 | NM_016331 |
| SOCS2 | ENSG00000120833 | NM_003877 |
| FKBP1B | ENSG00000119782 | NM_004116 |
| RHOQ | ENSG00000119729 | NM_012249 |
| NEK6 | ENSG00000119408 | NM_014397 |
| RAP1A | ENSG00000116473 | NM_002884 |
| DHCR24 | ENSG00000116133 | NM_014762 |
| CEBPZ | ENSG00000115816 | NM_005760 |
| GORASP2 | ENSG00000115806 | NM_015530 |
| GTF3C2 | ENSG00000115207 | NM_001521 |
| TP53I3 | ENSG00000115129 | NM_004881 |
| EIF4G1 | ENSG00000114867 | NM_004953 |
| TFDP2 | ENSG00000114126 | NM_006286 |
| ERGIC1 | ENSG00000113719 | NM_001031711 |
| DROSHA | ENSG00000113360 | NM_013235 |
| SLC29A1 | ENSG00000112759 | NM_004955 |
| PRPF4B | ENSG00000112739 | NM_003913 |
| HBS1L | ENSG00000112339 | NM_006620 |
| MCM3 | ENSG00000112118 | NM_002388 |
| CHD4 | ENSG00000111642 | NM_001273 |
| DCPS | ENSG00000110063 | NM_014026 |
| NAT9 | ENSG00000109065 | NM_015654 |
| DHRS7B | ENSG00000109016 | NM_015510 |
| SEC23IP | ENSG00000107651 | NM_007190 |
| EIF3A | ENSG00000107581 | NM_003750 |
| PRPF31 | ENSG00000105618 | NM_015629 |
| SNRNP70 | ENSG00000104852 | NM_003089 |
| KCNN4 | ENSG00000104783 | NM_002250 |
| MAN2B1 | ENSG00000104774 | NM_000528 |
| EMC2 | ENSG00000104412 | NM_014673 |
| GABPB1 | ENSG00000104064 | NM_002041 |
| CTSH | ENSG00000103811 | NM_004390 |
| DNAJA3 | ENSG00000103423 | NM_005147 |
| HMOX2 | ENSG00000103415 | NM_002134 |
| NUBP1 | ENSG00000103274 | NM_002484 |
| COG4 | ENSG00000103051 | NM_015386 |
| E2F1 | ENSG00000101412 | NM_005225 |
| NTSR1 | ENSG00000101188 | NM_002531 |
| RAE1 | ENSG00000101146 | NM_003610 |
| RABGGTA | ENSG00000100949 | NM_004581 |
| PCK2 | ENSG00000100889 | NM_004563 |
| LGMN | ENSG00000100600 | NM_005606 |
| KIAA0930 | ENSG00000100364 | NM_015264 |
| SGSM3 | ENSG00000100359 | NM_015705 |
| MCM5 | ENSG00000100297 | NM_006739 |
| DNAL4 | ENSG00000100246 | NM_005740 |
| RTCB | ENSG00000100220 | NM_014306 |
| DDX17 | ENSG00000100201 | NM_006386 |
| PES1 | ENSG00000100029 | NM_014303 |
| GADD45B | ENSG00000099860 | NM_015675 |
| RASSF7 | ENSG00000099849 | NM_003475 |
| HNRNPM | ENSG00000099783 | NM_005968 |
| TSPAN15 | ENSG00000099282 | NM_012339 |
| AAAS | ENSG00000094914 | NM_015665 |
| TINF2 | ENSG00000092330 | NM_012461 |
| HECTD1 | ENSG00000092148 | NM_015382 |
| ORC6 | ENSG00000091651 | NM_014321 |
| MAEA | ENSG00000090316 | NM_005882 |
| PXN | ENSG00000089159 | NM_002859 |
| MAPKAPK5 | ENSG00000089022 | NM_003668 |
| ADD1 | ENSG00000087274 | NM_001119 |
| SEPHS1 | ENSG00000086475 | NM_012247 |
| RAD54L | ENSG00000085999 | NM_003579 |
| APLP2 | ENSG00000084234 | NM_001642 |
| ITGB5 | ENSG00000082781 | NM_002213 |
| SMARCD3 | ENSG00000082014 | NM_003078 |
| KIAA0020 | ENSG00000080608 | NM_014878 |
| ARAF | ENSG00000078061 | NM_001654 |
| NFKB2 | ENSG00000077150 | NM_002502 |
| MCM6 | ENSG00000076003 | NM_005915 |
| SMARCE1 | ENSG00000073584 | NM_003079 |
| TCF3 | ENSG00000071564 | NM_003200 |
| TRIP13 | ENSG00000071539 | NM_004237 |
| NUCB2 | ENSG00000070081 | NM_005013 |
| BCL3 | ENSG00000069399 | NM_005178 |
| ERLEC1 | ENSG00000068912 | NM_015701 |
| CYFIP1 | ENSG00000068793 | NM_014608 |
| CYFIP2 | ENSG00000055163 | NM_014376 |
| PRSS8 | ENSG00000052344 | NM_002773 |
| FUT8 | ENSG00000033170 | NM_004480 |
| TSSC1 | ENSG00000032389 | NM_003310 |
| NR1H3 | ENSG00000025434 | NM_005693 |
| MED24 | ENSG00000008838 | NM_014815 |
| ITGA3 | ENSG00000005884 | NM_002204 |
| GCFC2 | ENSG00000005436 | NM_003203 |
| HCCS | ENSG00000004961 | NM_005333 |
| HS3ST1 | ENSG00000002587 | NM_005114 |
