# Supplementary material for: Genome-wide identification of Wig-1 mRNA targets by RIP-Seq analysis
Source: Oncotarget. 2015 Dec 11;7(2):1895–911. doi: 10.18632/oncotarget.6557 (PMC4811505; doi:10.18632/oncotarget.6557)
Supplement: Supplementary file 11 [file oncotarget-07-1895-s011.doc]

Supplementary Table S11: List of the TaqMan probes used for qRT-PCR

| **Gene name** | **Description** | **RefSeq** | **TaqMan Probe** |
| --- | --- | --- | --- |
| MAD2L1 | Mad2 Mitotic Arrest Deficient-Like 1 (Yeast) | NM_002358 | Hs01554513_g1 |
| MTHFD2 | Methylenetetrahydrofolate Dehydrogenase (NAPD+ Dependent) 2 | NM_006636 | Hs01073390_g1 |
| CCNG1 | Cyclin G1 | NM_004060 | Hs00171112_m1 |
| EIF4E | Eukaryotic Translation Initiation Factor 4E | NM_001968 | Hs00913390_m1 |
| CHEK1 | Checkpoint Kinase 1 | NM_001274 | Hs00967506_m1 |
| RMI1 | RecQ Mediated Genome Instability 1 | NM_024945 | Hs00227878_m1 |
| HIF1A | Hypoxia Inducible Factor 1, Alpha Subunit | NM_181054 | Hs00153153_m1 |
| AMD1 | Adenosylmethionine Decarboxylase 1 | NM_001634 | Hs01017615_g1 |
| CAV1 | Caveolin 1 | NM_001753 | Hs00971716_m1 |
| TP53I3 | Tumor Protein p53 Inducible Protein 3 | NM_004881 | Hs00936520_m1 |
| WARS | Tryptophanyl-tRNA Synthetase | NM_004184 | Hs00188259_m1 |
| GAPDH | Glyceraldehyde-3-Phosphate Dehydrogenase | NM_002046 | Hs99999905_m1 |
| ZMAT3 | Zinc Finger, Matrin-Type 3 | NM_022470 | Hs00536976_m1 |
|  |  |  |  |
